# Nature Counts to Three: Universal Mg-Pinch Motif Polarizes the Cleaved Bond in NTP-Processing Enzymes

**DOI:** 10.1021/jacs.6c10146

**Published:** 2026-07-13

**Authors:** Balint Dudas, Dénes Berta, Pablo Jambrina, Pedro J. Buigues, Reynier Suardiaz, Silvia Gómez-Coca, Wenhao Deng, Bernard R. Brooks, Beáta G. Vértessy, Edina Rosta

**Affiliations:** † Department of Physics and Astronomy, 4919 University College London, London WC1E 6BT, U.K.; ‡ Laboratory of Computational Biology, National Heart, Lung, and Blood Institute, National Institutes of Health, Bethesda, Maryland 20892, United States; § HUN-REN-BME Quantum Chemistry Research Group, Budapest University of Technology and Economics, 1111 Budapest, Hungary; ∥ MTA-BME Lendület Quantum Chemistry Research Group, Budapest University of Technology and Economics, 1111 Budapest, Hungary; ⊥ Department of Physical Chemistry and Materials Science, Budapest University of Technology and Economics, 1111 Budapest, Hungary; # Department of Physical Chemistry, University of Salamanca, 37008 Salamanca, Spain; ∇ Italian Institute of Technology, 16163 Genova, Italy; ○ Department of Physical Chemistry, Complutense University of Madrid, 28040 Madrid, Spain; ◆ Department of Inorganic and Organic Chemistry and Institute of Theoretical and Computational Chemistry,University of Barcelona, 08028 Barcelona, Spain; ¶ Department of Applied Biotechnology and Food Science, Budapest University of Technology and Economics, 1111 Budapest, Hungary; †† Genome Metabolism Research Group, Research Centre for Natural Sciences, Hungarian Research Network, 1117 Budapest, Hungary; ‡‡ Institute of Chemistry, Eötvös Loránd University, 1053 Budapest, Hungary

## Abstract

Phosphates are essential for life in all organisms, playing key roles in nucleic acids, signaling, energy transfer, and biosynthesis. We conducted a quantitative analysis of nucleoside triphosphate (NTP) processing enzymes across all enzymatic reactions, revealing their dominance in phosphate reactivity with ATP as the most prevalent substrate. Two main reaction types occur predominantly: cleavage resulting in pyrophosphate release or phosphate release/transfer. The large majority of NTP processing enzymes require divalent Mg^2+^ ions in a mechanistically analogous manner. However, the catalytically competent metal-ion coordination remains unclear for many NTP-processing enzymes. By examining a vast data set of crystallographic structures, we identified a universal “Mg-pinch” motif, confirming our structural hypothesis for almost all NTP processing enzymes. We hypothesized and subsequently confirmed that the catalytic Mg^2+^ typically coordinates the two phosphate groups between which the P–O bond is cleaved. We present a comprehensive analysis of NTP processing superfamilies across all species, determining distinct enzyme active site structures. We highlight exceptional cases and propose challenging superfamilies that lack sufficient structural data to determine precise active site coordination. DFT-based QM/MM calculations with full electrostatic embedding support a mechanistic interpretation in which appropriately coordinated Mg^2+^ ions contribute to catalysis by electrostatic preorganization and polarization of the reacting phosphate groups. The Mg-pinch motif provides a mechanistic framework for understanding the catalytic role of metal ions in NTP processing. Our findings offer insights into enzyme evolution, provide a basis for rational enzyme engineering, and could inform the development of novel therapeutics targeting NTP processing enzymes.

## Graphical abstract

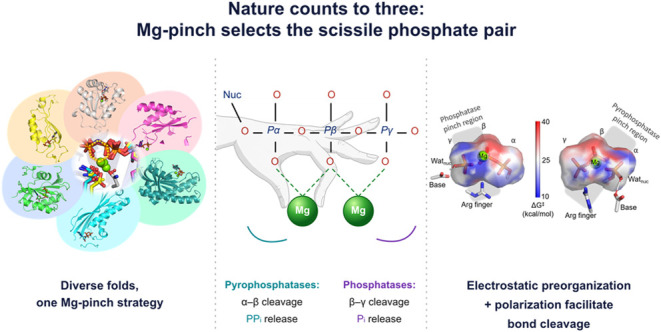

## 1 Introduction

Life, as we know it, depends on phosphate chemistry, which is essential for all major biological processes of living organisms, including the dynamic regulation and maintenance of genetic material; signaling and regulation; energy storage and transfer, and in activating and regulating biosynthetic pathways.^1,2^

The most basic building blocks involving phosphates are nucleotides. Although longer linear and cyclic phosphate anhydrides can form,^3^ nucleoside-5′-triphosphates (NTPs) represent the longest phosphate chains that dominate biological processes, central to enzymatic phosphate chemistry. Oligophosphates beyond NTPs only rarely occur.^4,5^

As essential cofactors for phosphate catalysis, divalent metal ions are required in the large majority of phosphate catalytic enzymes, not only in proteins, but also in RNA-based ribozymes.^6^ Mg^2+^ ions are the predominant natural cofactors;^7^ however, they can often be substituted by Mn^2+^.^8^ Ca^2+^ is also crucial in cell signaling, its concentration is heavily regulated, and, interestingly, Ca^2+^ often inhibits phosphate catalysis, consistent with its key role in apoptosis.^9,10^ The metal ion-aided phosphate catalytic mechanism is, therefore, considered a foundational element in the molecular basis of life.^11,12^ When in solution, early spectroscopic studies showed that Mg^2+^ interacts predominantly with the β-phosphate group of nucleoside triphosphates.^13^ Subsequent studies demonstrated that two dominant chelation geometries are accessible in solution: a bidentate (βγ-coordinated) and a tridentate (αβγ-coordinated) Mg^2+^–phosphate coordination mode separated by a high energy barrier.^14–16^

However, the structure–function relationship concerning the nucleoside—metal ion coordination in protein active sites differs from that in solution and is not well understood for phosphate catalysis. Bioinformatics tools can be used to predict NTP binding sites from as little as sequence information.^17,18^ These are, however, not always reliable in identifying the active sites. Even highly accurate structure-prediction algorithms based on machine learning, like AlphaFold 3,^19^ can be uncertain in lesser-known superfamilies. The coordination geometry, number of ions required varies among different types of enzymes, and there is a lack of systematic analysis. The catalytically competent active-site structure often remains elusive.^20–22^ Furthermore, quantitative subatomic resolution insights are experimentally lacking, which could provide comprehensive understanding of the catalytic role of these essential cofactors in modulating the kinetics of phosphate reactions in biology. No prior study has catalogued NTP-processing enzymes across all superfamilies at structural resolution.

Here we aim to systematically reveal the functional role of the divalent metal ions in NTP processing enzymes. We hypothesize that the catalytically active coordination of Mg^2+^ ions is determined by the chemistry performed by these enzymes: Mg^2+^ coordinates both phosphate groups involved in the cleaved P–O bond. We focused on the two most common reactions of NTP catalytic enzymes: hydrolysis or transfer (i) with phosphate and a nucleoside diphosphate product, or (ii) with pyrophosphate and nucleoside monophosphate products (Figure 1A,B). To challenge this hypothesis, we identified NTP-processing superfamilies (SFs) based on all available high-resolution crystallographic structures of NTP hydrolysis enzyme active sites deposited in the Protein Data Bank (PDB)^23^ to date. We found that almost all exhibit a universal metal ion coordination, which we termed the “Mg-pinch motif”, where an active site divalent metal ion coordinates both the attacked and the cleaved phosphate groups of the NTP simultaneously.

**Figure 1:**
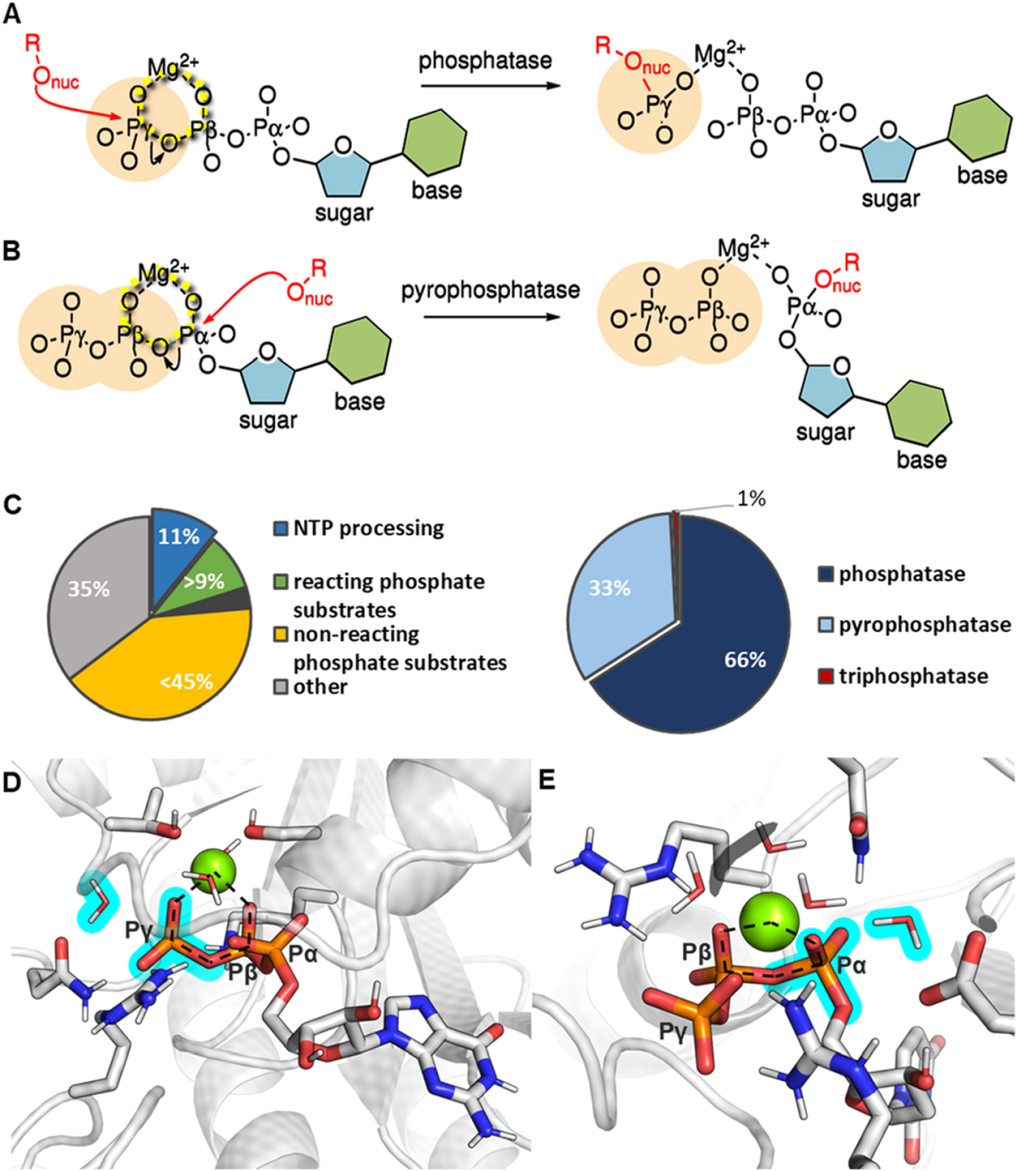
(A, B) Schematics of γ-phosphate (A, purple) and βγ-diphosphate (B, cyan) cleavages by an oxygen nucleophile (O_nuc_). “Mg-pinch” is highlighted by yellow hexagons. (C) Phosphate chemistry is key in enzymatic reactions, with the NTP-processing enzymes dominating biological processes. Statistical analysis was performed using Enzyme Commission Numbers. (D, E) Active site for phosphate hydrolysis in the KRas.p120GAP enzyme complex (D) and in dUTPase **(**E). Nucleophilic waters and the attached phosphates are highlighted in cyan.

To quantitatively evaluate geometrical, electrostatic, and polarization factors, we additionally performed quantum mechanical (QM) and quantum mechanical/molecular mechanical (QM/MM) calculations on three representative enzymes: a phosphatase, Ras (Figure 1D), a pyrophosphatase, dUTPase (Figure 1E), and a general two-metal ion catalytic phosphate cleaving enzyme, RNase H (SI Sections 1 and 9), revealing the key polarization effects essential to biocatalysis.

## 2 Results and Discussion

### 2.1 EC Analysis

To systematically identify examples of enzyme-catalyzed NTP processing reactions, we obtained reactivity data from the *Kyoto Encyclopedia of Genes and Genomes* (KEGG).^24^ We analyzed the substrates and products of the reactions categorized by the Enzyme Commission (EC) number assigned to enzymes. We evaluated (i) whether the reacting chemical species contain phosphates, and (ii) whether the reaction involves the phosphate groups directly, and finally (iii) whether the reaction uses NTP as a substrate.

Phosphates occur in at least one substrate in approximately 65% of EC numbers (4348 out of 6728), and more than 20% of all enzyme reaction types (1469) carry out phosphorus chemistry directly (Figure 1C), as measured by the prevalence of the EC-classified enzymatic reactions involving phosphate chemistry according to our KEGG analysis as described in the Methods (SI Section 1).

Demonstrating the importance of the triphosphate chain, about half of the phosphate processing enzymes use NTP (759). Of all NTP reacting EC reactions, about two-thirds of the enzymes (480) perform phosphatase activity, removing the γ-phosphate (Figure 1A), and about one-third are pyrophosphatases (272), cleaving between the α and β phosphates (Figure 1B). Six EC categories correspond to reactions cleaving all three phosphates of the nucleoside (2.5.1.6, 2.5.1.17, 2.5.1.154, 3.1.5.1, 4.1.2.50 and 4.2.3.12). EC 3.6.1.29 hydrolyses P^1^,P^3^-bisnucleoside-triphosphates, where the conventional numbering of the phosphates is ambiguous. Occasionally, more than three phosphates are attached to a nucleoside (e.g., in 3.6.1.17). Here we primarily focus on the two prominent NTP reactions, the phosphatase and pyrophosphatase activities, which are distinct and well-defined in all cases except for the above-mentioned exception.

### 2.2 Data Collection

To identify superfamilies (SFs) capable of NTP processing, we collected (i) PDB structures^23^ containing NTPs and bound divalent metal ions, as well as (ii) PDB structures and protein sequences that are associated with NTP-processing ECs in protein databases (KEGG,^25–27^ InterPro,^28^ and Expasy^29^) (Figure S1). All PDB entries meeting our geometric and resolution criteria were included regardless of mutations or deletions present in the active site, which were accounted for during representative structure selection. The identified protein chain sequences were then fetched to SUPERFAMILY 2.0 ^30,31^ to assign the corresponding SCOPe (Structural Classification of Proteins—extended) SFs^32,33^ by matching them against a collection of hidden Markov models that represent structural protein domains at the SCOPe SF level (a superfamily is a group of protein domains arisen from a common ancestor). For those cases where SUPERFAMILY did not identify any SCOPe SFs, we used the InterPro annotation.^28^ The identified SFs were then validated against the literature to ensure that the corresponding protein chains are indeed capable of NTP-processing. Finally, redundant SFs were merged if an InterPro entry matched an existing SCOPe SF based on the Evolutionary Classification of Protein Domains (ECOD)^34–36^ or on structural alignments with the distance matrix alignment (DALI) software.^37^ After aligning all structures within each SF, we examined crystallographic details—including active-site mutations, metal identity introduced during crystallization, and completeness of the coordination sphere—when selecting representative structures. Only structures exhibiting intact and unambiguous first-shell metal coordination were chosen. For each SF with sufficient structural information, a representative structure was identified based on the consensus metal-ion coordination pattern established through alignment of all members.

Strikingly, all identified NTP-processing SFs require at least one divalent metal ion, primarily Mg^2+^. We hypothesized that members of a given SF share a common active site arrangement with a corresponding consensus metal ion coordination, and that NTP-processing active sites exhibit a universal metal ion coordination strategy, which we termed the “Mg-pinch” motif. We hypothesized that the Mg-pinch, whereby both phosphates that are involved in the cleaved P–O bond are simultaneously coordinated, is universal in NTP-processing enzymes and is energetically advantageous for the catalytic reaction to polarize the reacting bonds. To validate our hypothesis, we analyzed the metal ions and their specific coordination geometries within the active sites for each SF. We introduced a reference plane defined by the three phosphorus atoms of the NTP and labeled the two half-spaces (+) and (−) such that, when viewed from the α-phosphorus, the (+) half-space corresponds to the side from which the α → β → γ sequence appears clockwise, while the (−) half-space corresponds to the counterclockwise orientation (see inset in Figure 2 top left corner and SI Figure S2). The main ion positions coordinated by two or three phosphates are color-coded (Figure 2) and labeled by which phosphates coordinate them with capital Latin letters (e.g., ABG for αβγ). We found that an Mg-pinch is formed in the vast majority of NTP-processing enzymes (96%, in 64 of 67 SFs with identified catalytic Mg^2+^ coordination), where the Mg^2+^ coordinates both phosphate groups between which the bond is cleaved during the catalytic reaction. This configuration results in the characteristic hexagonal arrangement, the Mg-pinch, depicted in Figure 2.

**Figure 2:**
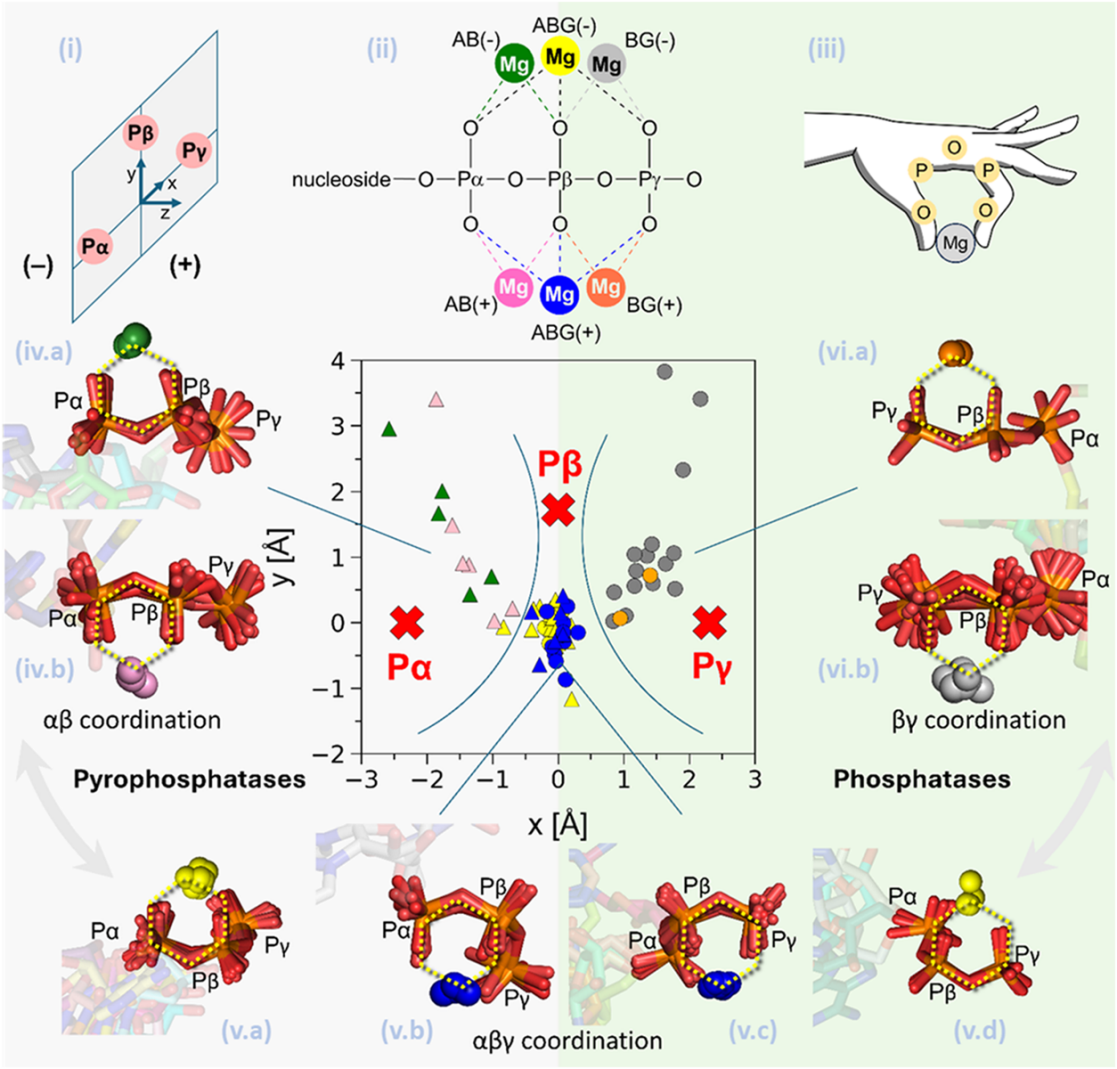
Mg-pinch configurations in pyrophosphatase (left, gray shading) and phosphatase (right, green shading) superfamilies (SFs). (i) Coordinate frame defining the (+)/(−) sides of the plane formed by the three phosphorus atoms. (ii) Schematic overview of the possible Mg-pinch positions relative to the triphosphate chain, highlighting the hexagonal arrangement formed by the bridging and nonbridging phosphate oxygens. (iii) Hand model illustrating the “pinching” geometry. Representative pyrophosphatase structures showing αβ (iv) and αβγ coordination modes (v.a, v.b). Representative phosphatase structures showing βγ **(vi)** and αβγ coordination modes (v.c, v.d) (yellow dashed hexagons). The faintly rendered nucleotide backbone is included only to illustrate the orientation of the triphosphate and to clarify the definition of the (+)/(−) sides of the triphosphate plane. The different Mg^2+^ ion clusters are color-coded: αβγ-coordinated ions (common to both phosphatases and pyrophosphatases) appear in blue on the (+) side (ABG­(+), (v.b and v.c)) and yellow on the (−) side (ABG(−), (v.a and v.d)); βγ-coordinated ions (typical for phosphatases) in orange (BG­(+), **vi.a**) and gray (BG(−), vi.b); and αβ-coordinated ions (characteristic of pyrophosphatases) in pink (AB­(+), **iv.b**) and dark green (AB(−), iv.a). **Central panel:** projection of Mg-pinch-forming metal-ion positions onto the plane of the three phosphorus atoms. Phosphatase ion positions are shown as circles and pyrophosphatase ion positions as triangles (using the color code above). The αβγ coordination is shared between both enzyme classes, whereas βγ coordination predominates among phosphatases and αβ coordination among pyrophosphatases. Alternative projections are shown in Figure S2.

### 2.3 Phosphatases: Pγ Leaving Group

We identified a total of 42 phosphatase enzyme SFs, of which 32 have consensus structures with clear Mg-pinch ion coordination (Table 1, SI Sections 2 and 10). These form four groups involving the β and the γ phosphate: ABG­(+), ABG(−), BG­(+), and BG(−) (Figure 2 right, Figure S3). The majority of phosphatases are either ABG­(+) (11 SFs) or BG(−) (15 SFs, Figure 3). An additional cluster of auxiliary ions are identified in the AG position (Figure S4). The exception of the *Metallo-dependent phosphatases* SF, with a well-defined representative structure, is discussed separately.

**Figure 3:**
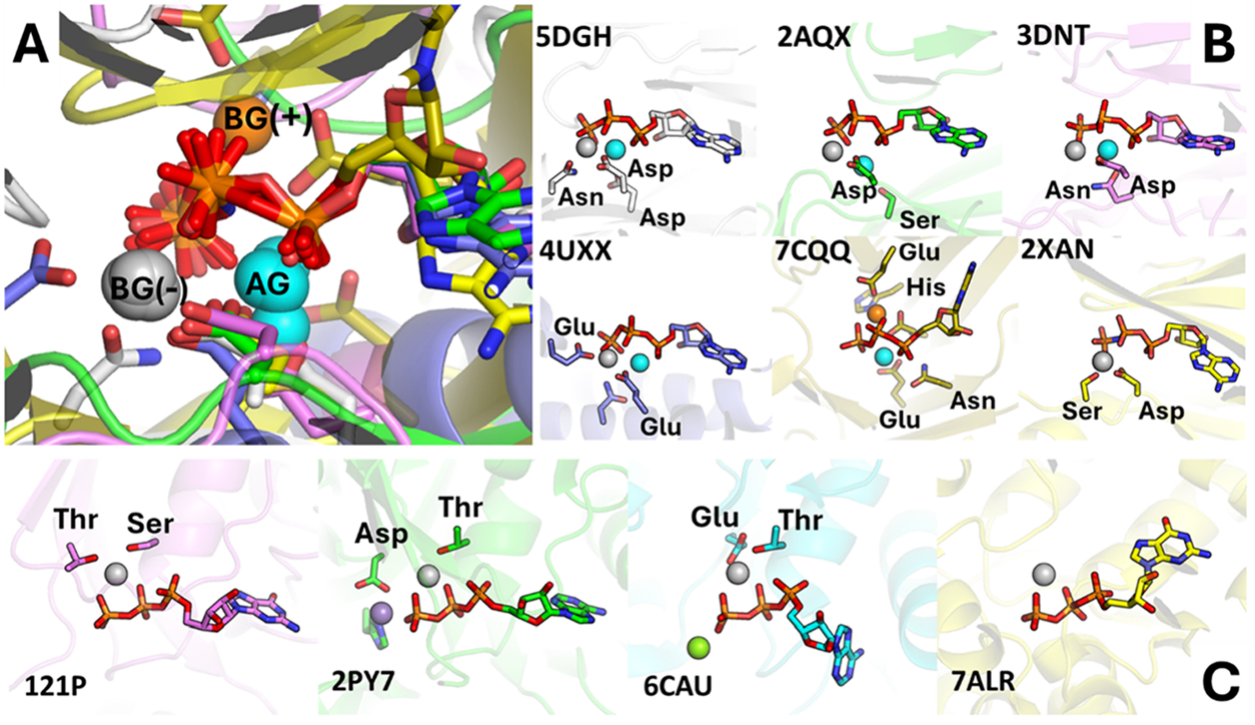
SFs with a BG(−) Mg-pinch metal ion. Proteins are shown as cartoons, nucleotides as sticks, and metal ions as spheres. (A) Structural overlay of six SF active sites, including ATP grasp enzymes, shown in (B), aligned on the bound triphosphate, illustrating the conserved location of the BG(−) pinching ion and the position of the auxiliary AG site (cyan). (B) Representative active sites from BG(−) SFs in which an additional divalent ion occupies the **AG** site (cyan): Glutathione synthetase ATP-binding domain-like (white, PDB 5DGH), SAICAR synthase-like (green, 2AQX), protein kinase-like (pink, 3DNT), and diacylglycerol kinase (DgkA)-like (blue, 4UXX). Glutamine synthetase/guanido kinase (olive, 7CQQ) is an exception, with the pinching ion located at **BG­(+)**. The structurally related inositol-pentakisphosphate 2-kinase (yellow, 2XAN) lacks the additional **AG** ion. (C) Similarities in BG(−) metal coordination across additional phosphatase SFs: P-loop NTP hydrolases (pink, 121P), PEP carboxykinase-like (green, 2PY7; second ion Mn^2+^ shown in purple), MurD-like peptide ligase catalytic domain (cyan, 6CAU; green cation, Mg^2+^), and tubulin nucleotide-binding domain-like (yellow, 7ALR).

**Table 1:**
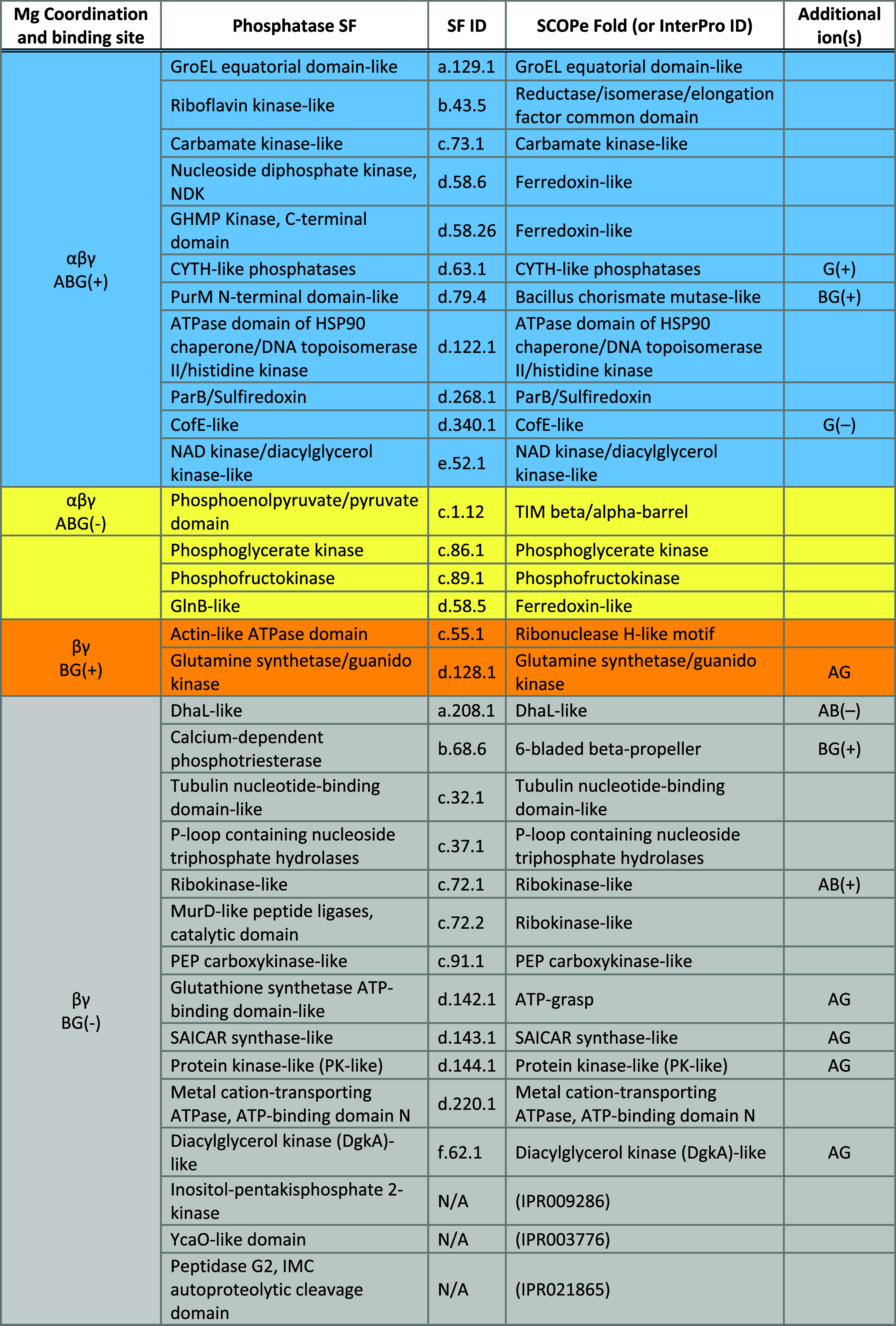
Phosphatase Superfamilies Grouped by Their Mg-Pinch-Forming Metal Ions (See Also SI Section 10 for Details)^a^

a The colors of the four groups match those of the corresponding ion positions in Figure 2.

The *P-loop containing nucleoside triphosphate hydrolase* SF, by far the largest in our data set with nearly 1000 corresponding high-resolution PDB structures, exhibits a highly conserved βγ phosphate coordination, besides a conserved serine and threonine residue in the first coordination sphere of the Mg^2+^ (Figure 3C). Both the *PEP carboxykinase-like* and the *MurD-like peptide ligase, catalytic domain* SFs exhibit nearly identical metal ion coordination configurations to the *P-loop containing NTP hydrolase* SF, where the serine is replaced by a threonine and an additional glutamate is involved in the Mg^2+^ coordination (Figure 3C). A very similar coordination is also present in the *Tubulin nucleotide-binding domain-like* SF, with the corresponding ∼250 structures showing highly consistent active-site coordination (Figure 3C). Four SFs—*Protein kinase-like*, *Glutathione synthetase ATP-binding domain-like*, *SAICAR synthase-like*, and *Diacylglycerol kinase (DgkA)-like*—share similar BG(−) coordination, accompanied by an additional αγ-coordinated metal ion (Figure S5A,B). In the case of the *Inositol-pentakisphosphate 2-kinase* group, no SCOPe SF could be assigned, and we termed it after EC 2.7.1.158 (InterPro SF, Inositol-pentakisphosphate 2-kinase, N-terminal lobe, IPR043001). This family was previously described to share some level of similarity to the *Inositol polyphosphate kinase* family belonging to the *SAICAR synthase-like* SF.^38^ A clear phosphate coordination with a Mg^2+^ at the BG(−) site can be identified for the corresponding structures. Even though the structural (DALI) alignment of the representative structure also revealed a good structural overlap with the *SAICAR synthase-like* SF where the nucleotides are positioned similarly, we introduced them as distinct categories due to some clear differences observed at the active site (Figure S6). Furthermore, the ECOD fold is also classified as *SAICAR synthase-like* SF using the ATP-grasp motif.^39^ The *Diacylglycerol kinase (DgkA)-like* SF has a similar ATP configuration and Mg^2+^ coordination to those discussed above, but the overall secondary structure is rather different (Figure S5B).

The *Actin-like ATPase domain* is the most abundant phosphatase superfamily having a metal ion on the (+) side—with ∼200 corresponding high-resolution PDB structures uncovered—and features a one metal-ion at its active site.

Some other SFs had less structural data available or lacked a SCOPe annotation. For the *DhaL-like* superfamily, only one structure with ATP and two metal ions was identified (PDB 1UN9),^40^ which was not part of the high-resolution data set. This SF was identified as the corresponding enzymes for three EC categories (SI Appendix, Note 1). Other ADP-containing structures suggest that it most probably exhibits an αβ and a βγ coordinated Mg^2+^. The *YcaO-like domain* SF—for which we identified one Mg^2+^ at the

BG­(−) site—is defined on the InterPro domain level (IPR003776) and has been shown to use ATP to activate amide backbones during peptide cyclodehydrations.^41^ However, there is no associated EC to this domain and its corresponding structures. ECOD classifies the fold as *Heterocyclase TruD C-terminal domain*. Similarly, no associated EC exists for the *Peptidase G2, IMC autoproteolytic cleavage domain* SF (defined based on the InterPro domain level, IPR021865) and the corresponding structures of the premature bacteriophage phi29 gene product 12 originate from a single publication.^42^ The authors suggest that autocleavage of the C-terminal domain is a post-trimerization event that is followed by a unique ATP-dependent cleavage and release.^42^ The corresponding ECOD fold is classified as *Head decoration protein D (gpD, major capsid protein D)*. Based on the structures we propose a BG(−) Mg^2+^ coordination.

### 2.4 Pyrophosphatases: PβPγ Leaving Group

Among the 90 SFs in our data set, 44 SFs have pyrophosphatase activity, 32 of which have available structures enabling us to clearly determine the corresponding Mg-pinch coordination (Table 2, SI Sections 3 and 11). Two exceptions (*Acetyl-CoA synthetase-like* and *Alkaline phosphatase-like* SFs) with well-defined representative structures are discussed separately. The Mg-pinch in pyrophosphatases has an αβ or an αβγ metal ion coordination, forming four groups: ABG­(+), ABG(−), AB­(+), and AB(−) (Figure 2, left, Figure S7).

**Table 2:**
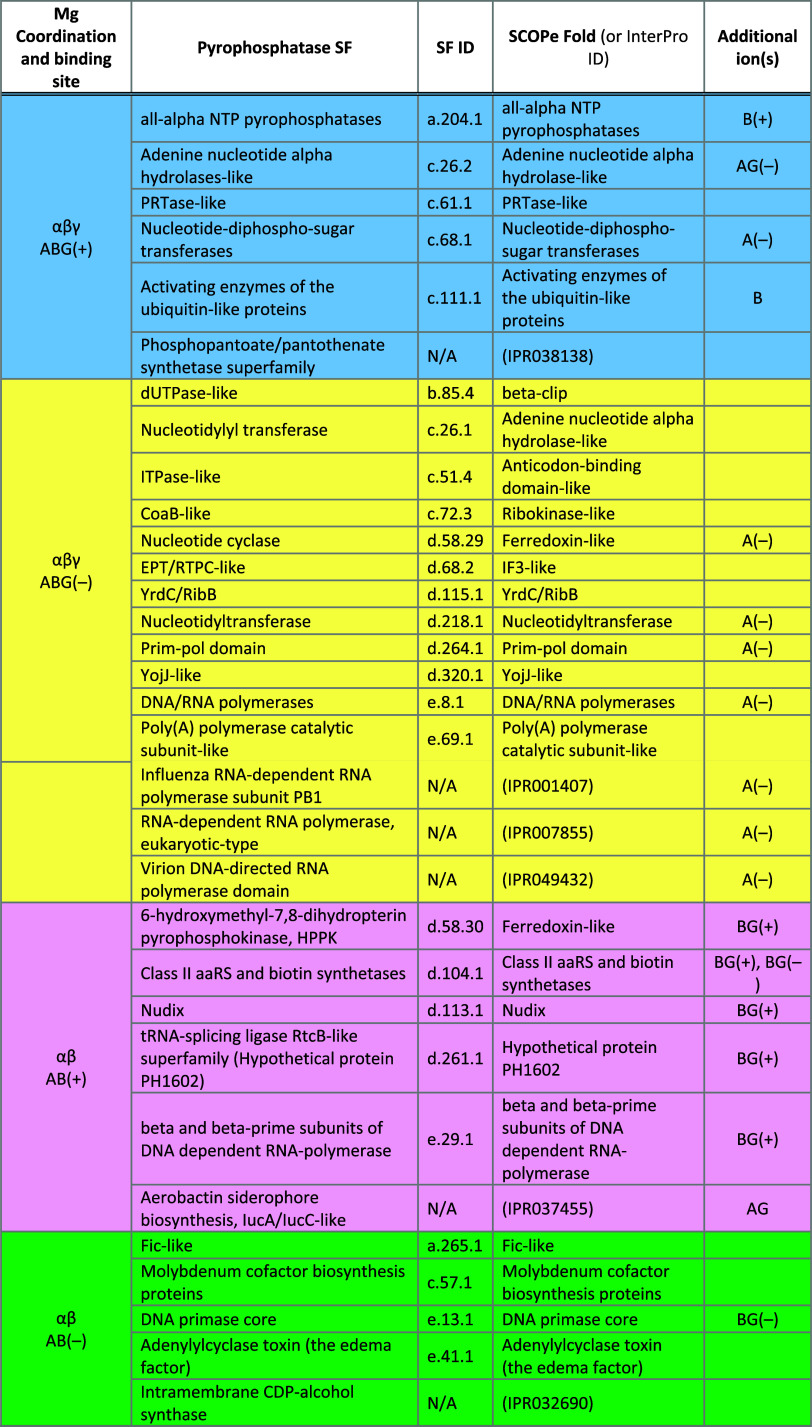
Pyrophosphatase Superfamilies Grouped by Their Mg-Pinch-Forming Metal Ions (See Also SI Section 11 for Details)^a^

a The colors of the four groups match those of the corresponding ion positions in Figure 2.

Twenty SFs possess a Mg-pinch-forming metal ion on the (−) side, the majority (15 SFs) showcasing αβγ-coordination. Among these, the *DNA/RNA polymerase* and the *Nucleotidyltransferase* SFs display a remarkably similar nucleoside configuration, orientation, and ion coordination. These are the two most populated pyrophosphatase (PPi) SFs in our high-resolution PDB data set, with ∼475 and ∼340 structures, respectively. Despite their structurally distinct folds, the two SFs exhibit nearly identical positioning and orientation of the nucleoside, catalytic residues, and coordinating metal ions. Both demonstrate a clear two-metal-ion catalytic coordination,^22^ with a third metal ion present in some structures, located not far from the γ phosphate, potentially playing a role in ligand release.^43^ Alongside the αβγ-coordinated Mg^2+^ that forms the Mg-pinch, the second Mg^2+^ is coordinated by the α phosphate and the attacking hydroxyl group of the ligand nucleoside (Figure 4).

**Figure 4:**
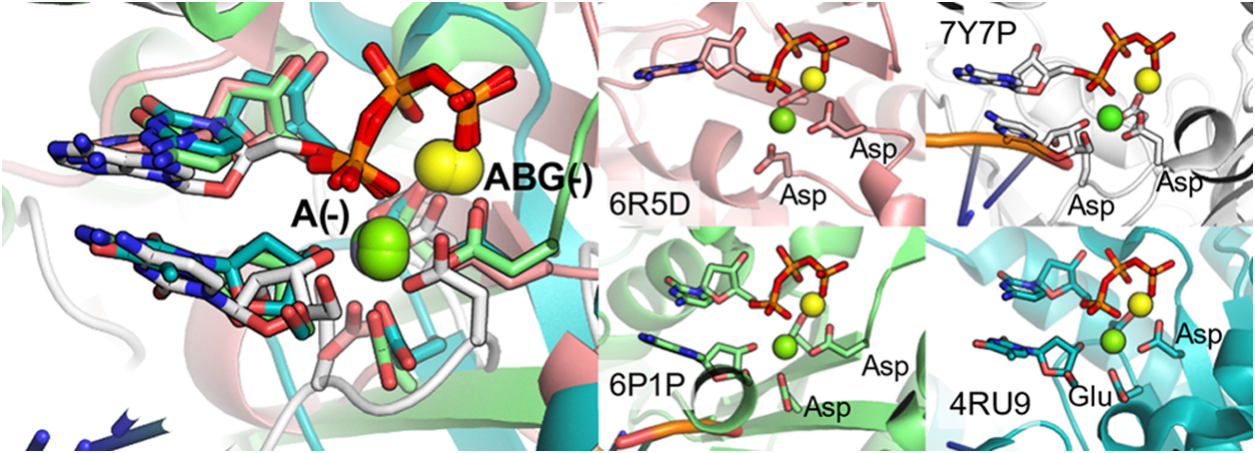
Pyrophosphatases with additional ion between the α phosphate and the nucleophile alongside the ABG(−) pinching ion, typical to polymerases: Prim-pol domain (salmon, 6R5D); RNA-dependent RNA polymerase, eukaryotic-type (white, 7Y7P); Nucleotidyltransferase (green, 6P1P); DNA/RNA polymerases (teal, 4RU9).

SCOPe classification using SUPFAM did not always give a result, or sometimes it was incorrect. We identified a group of PDB structures, exemplified by PDB 2IRX^44^ that were classified as *PLP-dependent transferases* (yet with a weak score) or were not classified at all by SUPFAM 2.0. By using the InterPro database^28^ we clearly identified this domain to be the *DNA ligase D, polymerase domain* (the *PLP-dependent transferase* was a misclassification). After comparing the corresponding PDB structures to our data set, we found that these belong to the *Prim-pol* superfamily, yet this domain remains unannotated in the SCOPe database.^32,33^

No SCOPe annotation is available for the structures of the *Influenza RNA-dependent RNA polymerase subunit PB1* (defined at the InterPro family level, IPR001407), the *Virion DNA-directed RNA polymerase domain* (defined on the InterPro domain level, IPR049432), and the *RNA-dependent RNA polymerase, eukaryotic-type* (defined at the InterPro family level, IPR007855) SFs. All three SFs exhibit a typical two-metal ion catalytic active site configuration highly alike polymerases, yet they result in a poor overall DALI alignment suggesting that they represent distinct SFs. Accordingly, ECOD identifies three distinct folds, *Adenylyl and guanylyl cyclase catalytic domain-like*, *helical bundle in Bacillus stearothermophilus*-*like DNA polymerase I*, and *RNAi polymerase N-terminal domain*.

*Intramembrane CDP-alcohol synthase* SF also does not have a SCOPe annotation. Additionally, it matches multiple InterPro families that are not connected with each other, such as phytol and farnesol kinases IPR039606 (see also SI Section 4, Phytol kinase sequence analysis), as well as Pfam Dolichol kinases (PF28421). SUPFAM also classified 5GUF reference structure as mitochondrial carrier SF with weak confidence, however this is incorrect and corresponds to a short domain match with high uncertainty.

Some of the (+) side SFs also do not have SCOPe annotation, we identified the *Phosphopantoate/pantothenate synthetase* and *Aerobactin siderophore biosynthesis, IucA/IucC-like* SFs that have a unique active site organization and are clearly distinct from other PPi SFs based on DALI alignments. The former SF has an ECOD classification as Rossmann-related, DHS-like NAD/FAD-binding domain. Intriguingly, *Aerobactin siderophore biosynthesis, IucA/IucC-like* SF is identified as protein kinase fold, and has a close similarity to both phosphatase (Pi) catalytic *PK-like* and *SAICAR-synthase-like* SFs. Similarly, the Protein adenylyltransferase SelO family defined in the InterPro database (IPR003846) is known to have a *PK-like* fold and it is termed as a “flipped pseudokinase”, which carries a PPi function.^45,46^ In these InterPro families (Figure S5C), the altered orientation of the triphosphate chain enables the formation of a canonical Mg-pinch between Pα and Pβ, and supports chemistry that does not involve acylphosphate or phosphoryl-transfer intermediates typical of ATP-grasp enzymes.

### 2.5 Exceptional Coordination and Superfamilies Challenging to Classify

While the *Acetyl-CoA synthetase-like* SF uses Mg^2+^ as cofactor, its BG­(+) coordination is exceptional considering its pyrophosphatase activity (Figure 5A–C). A potential reason for the deviation from our hypothesis is due to the negatively charged attacking group, whereby the nucleophilic attack can occur more readily and does not necessitate the metal coordination between Pα and Pβ to hydrolyze ATP to AMP and pyrophosphate. Unlike classical pyrophosphatases that attack by neutral molecules like water, acetyl-CoA synthetases generate an acyl-adenylate intermediate using a negatively charged nucleophile and release pyrophosphate as a byproduct of adenylation.^47^

**Figure 5:**
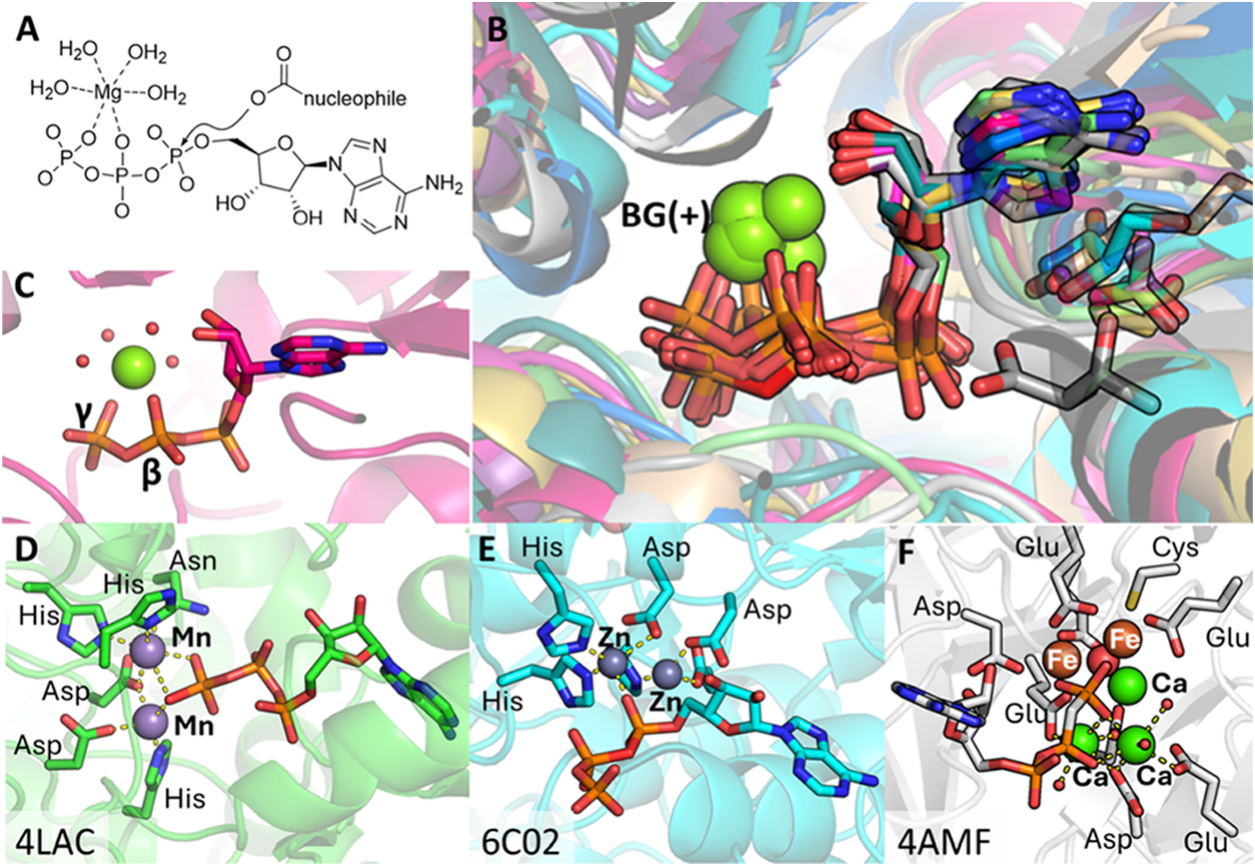
(A) Reaction scheme of carboxylate activation in peptide synthesis catalyzed by the Acetyl-CoA synthetase-like SF. (B) Multiple structural alignment agrees on metal coordination and secondary structure. (C) Metal ion coordination in the Acetyl-CoA synthetase-like SF (PDB 5BSM). Mg^2+^ ions are not colored based on their coordination. Exceptional metal ion coordination is also displayed in the (D) Metallo-dependent phosphatases SF (green, 4LAC) (E) Alkaline phosphatase-like SF (cyan, 6C02) and (F) Ca-dependent phosphotriesterase SF (white, 4AMF).

A decisive conclusion regarding the metal coordination and corresponding representative structures could not be drawn for further 9 phosphatase and 10 pyrophosphatase SFs due to the lack of available structures with NTP and metal ions in their active site (Table 3). Some of the SFs had only structures in the product state—e.g., the *Thiamin pyrophosphokinase, catalytic domain* SF. Using these structures with thiamine diphosphate and AMP, we hypothesize an αβγ coordination. NTP analogues can also distort the active site—e.g., the *RibA-like* SF, where we hypothesize an αβ coordination. In some cases high resolution structures do not represent catalytically active complexes—e.g., the DNA or RNA is missing in the structures of the *DNA ligase/mRNA capping enzyme, catalytic domain* SF for which a two metal-ion mechanism was proposed,^48–50^ yet it cannot be validated based on the available structures. There are further SFs, for which no substrate-bound active site structures are available to date.

**Table 3:** Superfamilies the Coordination of Which Could Not Be Determined with Certainty Due to the Lack of Structures with High-Resolution Active Sites, in the Presence of NTP and Metal Ions

Superfamily	Reaction	SF ID	SCOPe Fold (or InterPro ID)
Transglutaminase, two C-terminal domains	*Pi*	b.1.5	Immunoglobulin-like β-sandwich
Apyrase	*Pi*	b.67.3	5-bladed β-propeller
FomD-like	*Pi*	b.175.1	FomD barrel-like
Nicotinate/Quinolinate PRTase C-terminal domain-like	*Pi*	c.1.17	TIM β/α-barrel
NagB/RpiA/CoA transferase-like	*Pi*	c.124.1	NagB/RpiA/CoA transferase-like
Glycerate kinase I	*Pi*	c.141.1	Glycerate kinase I
YgbK-like	*Pi*	c.146.1	YgbK-like
HIT-like	*Pi*	d.13.1	HIT-like
Phospholipase D/nuclease	*Pi*	d.136.1	Phospholipase D/nuclease
S-adenosyl-l-methionine-dependent methyltransferases	*PPi*	c.66.1	S-adenosyl-l-methionine-dependent methyltransferases
Thiamin pyrophosphokinase, catalytic domain	*PPi*	c.100.1	Thiamin pyrophosphokinase, catalytic domain
RibA-like	*PPi*	c.144.1	RibA-like
Molybdenum cofactor biosynthesis protein C, MoaC	*PPi*	d.58.21	Ferredoxin-like
RPB5-like RNA polymerase subunit	*PPi*	d.78.1	RPB5-like RNA polymerase subunit
DNA ligase/mRNA capping enzyme, catalytic domain	*PPi*	d.142.2	ATP-grasp
tRNA(Ile2) 2-agmatinylcytidine synthetase TiaS	*PPi*	N/A	(IPR024913)
Phosphatidate cytidylyltransferase, mitochondrial	*PPi*	N/A	(IPR015222)
BioW-like	*PPi*	N/A	(IPR005499)
GTP cyclohydrolase MptA	*PPi*	N/A	(IPR022840)

While the majority of SFs are ubiquitous in all kingdoms of life, lack of structural information sometimes also corresponds to the restricted prevalence of the enzymes in various species, when primarily prokaryotic or microorganisms use the corresponding enzymes. For example, the *tRNA­(Ile2) 2-agmatinylcytidine synthetase TiaS* SF was found to be present in extremophile archaea species only, or the *Molybdopterin cofactor biosynthesis C, (MoaC)* SF is identified in bacteria, archaea, and plants only. The former has an ECOD classification as *Adenylyl and guanylyl cyclase catalytic domain-like*, and, accordingly, structural similarity with the *Influenza RNA-dependent RNA polymerase subunit PB1* SF, however the substrate binding seems distinct.

In three linked membrane-bound kinase categories (2.7.1.174, 2.7.1.182, and 2.7.1.216) there were no available experimental structures. We therefore assembled all available UniProt sequences for each EC number and used UniProt AlphaFold predictions to obtain structural representatives (see also SI Section 4, Phytol kinase sequence analysis). Structural similarity was quantified using DALI, yielding Z-scores, RMSDs, and alignment lengths for each query–reference comparison. The EC 2.7.1.182 family (1,570 AlphaFold models) was analyzed in detail by systematic DALI structural comparison against a curated reference panel, including two internal anchors: A0A1F8NHP0 (A1) and A0A8H3B4Z4 (B1), which do not align well to each other. Clustering on the full-matrix representation of standardized DALI Z-scores and RMSDs (missing alignments set to *Z* = 0 and RMSD = 100) resolved two dominant structural regimes and a smaller heterogeneous subset (Table S2; Figures S8–S11). One class consistently aligned most strongly with the AlphaFold reference structure A1, while a second class aligned preferentially with B1. The A1-like cluster corresponds to the Intramembrane CDP-alcohol synthase SF and is structurally consistent with the same fold observed in phosphatidate cytidylyltransferases (A1-like; median *Z* to A1 ≈ 32). All structures from ECs 2.7.1.174 and 2.7.1.216 also matched this cluster. A second, B1-like major cluster exhibited lower but still significant structural similarity (median *Z* to B1 ≈ 6), suggesting a more divergent fold that does not align convincingly to any established SF in our reference set (typical intertemplate *Z* around ∼3 or less). AlphaFold confidence also differed by cluster: the dominant structural regimes showed higher and more concentrated mean pLDDT distributions than the heterogeneous subset, consistent with the latter reflecting mixed-quality models and/or atypical architectures (Figures S9–S11). A phylogeny inferred from 70% identity–reduced representatives further confirmed this organization, with strong local clustering of tips by structural label and reduced within-cluster versus between-cluster patristic distances (Figures S12–S13). Collectively, these computational analyses awaiting experimental structural data nominate two principal structural subtypes within EC 2.7.1.182, providing a framework for more detailed mechanistic and evolutionary analysis. It is uncertain whether B1-like proteins would present a distinct active site geometry or they would require a binding partner for catalytic activity, we therefore opted not to include B1-like sequences as a distinct SF awaiting further biochemical and structural data.

There is one other SF for which literature verifiable NTP processing takes place, but no structural information exists, it is the *GTP cyclohydrolase MptA* SF.^51^ Based on our structure predictions using AlphaFold 3, this is likely a novel PPi NTP processing SF, structurally matching type GCYH-IB GTP cyclohydrolases. The highly homologous experimental structures (5K95 and 3D2O) have a classification as *Tetrahydrobiopterin biosynthesis enzymes-like* (a PPPi SF) by ECOD. In some cases, AlphaFold 3 could not accurately predict ligand binding. For Apyrase (using template 1S1D), Phosphatidate cytidylyltransferase, mitochondrial (using template 6IG2), and Molybdenum cofactor biosynthesis protein C, MoaC (using template 3JQM) SFs the AlphaFold 3 prediction results in an incorrect orientation of the nucleotide (see SI Section 12).

Similarly, the BioW-like SF (IPR005499, EC 6.2.1.14) lacks any SCOPe/SUPFAM fold assignment, yet experimental structures of *Aquifex aeolicus* and *Bacillus subtilis* BioW reveal a distinct fold that does not match any other adenylate-forming enzyme; based on the postcatalytic complex with pimeloyl-AMP, pyrophosphate, and two Mg^2+^ (PDB 5FLL), we tentatively classify the catalytic ion as ABG­(+) given the coordination by two conserved aspartate residues (Asp195, Asp196) on the protein face of Pα. The water-coordinated second Mg^2+^ in this structure most plausibly represents a postcatalytic spectator, but whether two metal ions are catalytically required and whether the assignment is AB­(+) rather than ABG­(+) cannot be resolved from the available structural and mechanistic data.

We also encountered some historically inaccurate EC assignments. For example, recent Cryo-EM structures of FAST kinases were not found to possess any kinase activity,^52^ despite the original proposition^53^ and EC annotation. This EC entry (2.7.11.8) is therefore omitted from our SF assignments. We further identified 71 NTP-processing ECs that are not directly associated with any representative structure in the PDB. By systematic literature, KEGG and UniProt sequence analysis supplemented by AlphaFold modeling and TM-align structural comparison against representative SF members, we could confidently assign 54 of these to existing SFs (52 verified by structural data or unambiguous sequence orthology to characterized SF members; 2 likely on the basis of family relationships). The remaining 17 ECs (SI Appendix, Note 2) lack any sequence or structural information beyond decades-old enzymological characterization, or appear to correspond to side-activities of already-classified enzymes.

Finally, while a well-defined consensus structure can be identified for all distinct SFs that carry out a well-defined reaction, this might not always be the case in some very rare examples. MutT1 reportedly carries out phosphatase activity,^54^ although being a member of the *Nudix* SF, confirmed both by sequences and alignment using AlphaFold 3 predicted structures, as experimental structures were not available. *Nudix* members, however, generally hydrolyze pyrophosphates. Two distinct groups are present in the *Adenine nucleotide α hydrolases-like* SF: ATP hydrolysis of the stress proteins within this SF that bind a single Mg^2+^ coordinated by the αβγ phosphates is unlikely,^55,56^ whereas the remaining enzymes of this SF have a second Mg^2+^ at the AG(−) site and possess pyrophosphatase activity.

OxsA is a member of the *HD-domain/PDEase-like* SF of enzymes, which are typically triphosphatases. Interestingly, OxsA processes oxetanocin A triphosphate compounds, acting as a phosphatase despite its HD/PDEase domain. However, the substrate coordination is altered compared with that of other triphosphatases, and the Mg^2+^ coordination shows an ABG(−) coordination, fulfilling the Mg-pinch hypothesis (see more details in SI, Section 13, HD-domain/PDEase-like SF).^57^

### 2.6 Triphosphatases: P_α_P_β_P_γ_ Leaving Group

Only five EC categories correspond to reactions cleaving all three phosphates of the nucleoside (2.5.1.6, 2.5.1.17, 2.5.1.154, 3.1.5.1, 4.1.2.50) and one (4.2.3.12) that similarly processes 7,8-Dihydroneopterin 3′-triphosphate. We identified four SFs that catalyze these reactions (Table 4), a minority compared with 86 phosphatase and pyrophosphatase SFs. Their coordination is very variable (Figure S14). The *S-adenosylmethionine synthetase* SF has a Mg^2+^ in the ABG(−) position and another in the AG­(+), the *Cobalamin adenosyltransferase-like* SF one at ABG­(+) and another at A(−), the *HD-domain/PDEase-like* SF one at BG­(+) and two additional ions coordinated by the α phosphates. The coordination could not be determined for the 7,8-Dihydroneopterin 3′-triphosphate processing *Tetrahydrobiopterin biosynthesis enzymes-like* SF, due to the lack of structural information. The phosphate chemistry of triphosphatases differs from those of the phosphatases and pyrophosphatases, as for triphosphatases the bond is cleaved between C5′-O5′, as opposed to the P–O bond cleavage.

**Table 4:** Triphosphatase Superfamilies and Their Metal Ion Coordination

Triphosphatase SF	SF ID	Fold	Mg^2+^ coordination	Additional ion(s)
Cobalamin adenosyltransferase-like	a.25.2	Ferritin-like	ABG(+)	A(−)
HD-domain/PDEase-like	a.211.1	HD-domain/PDEase-like	BG(+)	A, A
Tetrahydrobiopterin biosynthesis enzymes-like	d.96.1	T-fold	N/A	N/A
S-adenosylmethionine synthetase	d.130.1	S-adenosylmethionine synthetase	ABG(−)	AG(+)

### 2.7 Non-Mg^2+^ Cofactors

The vast majority of NTP-processing enzymes rely on Mg^2+^ as the divalent catalytic metal ion cofactor, with the coordination of six 2-electron donors in an octahedral arrangement. Some enzymes may utilize Mn^2+^ as their preferred metal ion cofactors. Manganese was identified to be optimal for the guanylylation reaction of the RtcB enzyme belonging to the *tRNA-splicing ligase RtcB-like* SF.^58^ Likewise, PrimPol of the *Prim-pol domain* SF is Mn^2+^ dependent that shows significantly improved primase and polymerase activities when binding Mn^2+^ rather than Mg^2+^ as cofactors.^44,59^ In addition, a rare case of Zn^2+^-mediated NTP catalysis is reported in some *Diacylglycerol kinase (DgkA)-like* bacterial enzymes, where ATP is coordinated by Zn^2+^ rather than Mg^2+^ (PDB ID: 4UXX).^60^

Intriguingly, although Ca^2+^ inhibits NTP-processing enzymes in almost all cases, the *soluble* adenylyl cyclase (sAC) from *S. platensis* is an exception in the *Nucleotide cyclase* SF. In the active site, Ca^2+^ plays the pinching metal ion role at the ABG(−) position, while an α-coordinated Mg^2+^ coordinates the attacking hydroxyl group of the ATP, orienting it for the cyclase reaction.^61^ The unusual Ca^2+^ activation is suggested to increase ATP binding and lead to more effective overall kinetics.^62^ Most enzymes of this SF, however, use Mg^2+^ in both ion positions.

Very few enzymes do not work with Mg^2+^. The *Metallo-dependent phosphatases* SF has an atypical coordination with two metal ions at its active site, usually manganese, iron, or zinc (Figure 5D).^63^ It is, however, more promiscuous and can hydrolyze phosphate chains of various lengths in addition to NTPs, whereas typical NTP catalytic enzymes have a clear specificity for triphosphates. Similarly, the nucleotide-degrading members of the *Alkaline phosphatase-like* SF contain two Zn^2+^ ions instead of Mg^2+^ at their catalytic center (Figure 5E) for their pyrophosphatase activity.

The only family belonging to the *Calcium-dependent phosphotriesterase* SF (b.68.6) that processes NTP as substrate, is the *Alkaline phosphatase PhoX* family (InterPro entry IPR008557), containing proteins predominantly found in bacteria. Its complex active-site comprised of two antiferromagnetically coupled ferric iron ions (Fe^3+^), three calcium ions (Ca^2+^), and an oxo group bridging three of the metal ions.^64^ Interestingly, two of the Ca^2+^ ions have pinching positions in this group, in BG­(+) and BG(−) coordination (Figure 5F). The DALI alignment of the SF representatives revealed a surprisingly close resemblance to the *Apyrase* SF (b.67.3) for which the coordination could not be deduced based on the available structures.

We also assessed whether the protein or domain lengths correlate with the reaction type (Pi or PPi release) or the number of ions used (see SI Section 6 for details, Figures S15–S21). We focused on 61 SFs for which we had both (i) a reliable fold/superfamily assignment via SUPFAM and (ii) a consensus annotation of the active-site metal content. The number of ions in the active site was determined from the representative structures, irrespective of whether these coordinated the triphosphate chain or some other substrate/protein groups. We analyzed (i) curated domain boundaries from the CATH database and obtained >44,000 domain instances mapping to 60 CATH superfamilies as well as (ii) retrieved >16 million full-length UniProt sequences for 61 SCOPe SFs. In both the domain-level data set and the full-length sequence data set we did not observe a systematic increase or decrease in length as a function of (a) Pi vs PPi-releasing chemistry or (b) the number of active-site metal ions. We note that PPi-releasing enzymes are most often implemented with two-metal catalytic arrangements, whereas enzymes that release Pi tend to use a single metal ion more frequently. That difference in mechanism, however, is not accompanied by a detectable shift in overall chain or domain length. Unlike protein NTP-processing enzymes, this kind of length–metal dependence is much more pronounced in catalytic RNAs, where divalent cations such as Mg^2+^ are needed not only for chemistry but to neutralize the RNA backbone and stabilize the tertiary fold;^65–67^ large ribozymes only reach their active conformations at sufficient Mg^2+^ concentrations, and often position more than one Mg^2+^ in the core.^68^

Across the 71 representative structures with known metal coordination, first-shell coordination of catalytic metal ions is dominated by carboxylate residues (Asp or Glu, together accounting for over half of all observed ligands), with other oxygen donors and coordinated waters contributing more variably (SI Table S3). While most Mg^2+^-dependent SFs rely exclusively on O-based ligands, several transition-metal-using SFs employ histidines, and rare cases such as the RtcB-like SF include cysteine ligation. Notably, histidine coordination also occurs in a few Mg^2+^-dependent SFs (SI Figure S22). Full residue distributions and structural examples are provided in the Supporting Information.

### 2.8 Analysis on the Protein Fold Level

For those SFs that are defined in the SCOPe database, we collected their corresponding fold information. The fundamental unit in the SCOPe database is the domain found in experimentally determined protein structures, which are organized in several levels of hierarchy. While SFs bridge together protein families with common functional and structural features inferred to be from a common evolutionary ancestor, a fold, which corresponds to the level above SFs, groups structurally similar SFs solely relying on structural features; hence sharing a common fold does not imply sequence homology.^32^

NTP hydrolysis enzymes cover a wide range of protein folds defined in the SCOPe database, and even the same fold does not imply similar metal ion coordination geometries. In our data set, 77 SFs originate from the SCOPe database, and cover a total of 67 folds. We identified a maximum of six NTP-processing SFs within the same *Ferredoxin-like* fold. Three SFs belong to the *Ribokinase-like* fold, and two SFs belong to the *TIM β/α-barrel*, the *ATP-grasp*, and the *Adenine nucleotide α hydrolase-like* folds each. The remaining 62 SFs all belong to distinct folds.

Moreover, SFs in the same fold may even catalyze distinct reaction classes. In the *Ferredoxin-like* fold, three SFs are phosphatases and three are pyrophosphatases, with very diverse coordination geometries (Figure S23A). Similarly, both the *Glutathione synthetase ATP-binding domain-like* and the *DNA ligase/mRNA capping enzyme, catalytic domain* SFs belong to the *ATP-grasp* fold, yet the former is a phosphatase, and the latter is a pyrophosphatase. Their nucleotide binding is clearly distinct (Figure S5B). Even if the NTP-processing is similar, as in the *Ribokinase-like* fold, where both the *Ribokinase-like* and the *MurD-like peptide ligases, catalytic domain* SFs are phosphatases and they both have a Mg^2+^ at the BG(−) position, when overlapped, their active sites are located distantly from each other (Figure S23B).

On the other hand, very different secondary structural arrangements may create similar active sites to tackle similar reaction mechanisms (Figure S24). This is observed for four polymerases (*Prim-pol domain*; *Nucleotidyltransferase*; *DNA/RNA polymerases*; and *RNA-dependent RNA polymerase, eukaryotic-type*) that belong to different folds (d.264, d.218, e.8) or are not defined in SCOPe, yet they share high similarities in their active sites. They all exhibit a Mg^2+^ binding site at ABG(−) and another at A(−). In all cases, both ions are coordinated by two conserved aspartates. The A(−) ion is additionally coordinated by a side chain carboxyl group, as well as by the 3′ hydroxyl of the priming nucleoside. The three coordinating side chains can reside on different structural elements, yet their relative positions and orientations are strikingly similar among the different SFs that catalyze the polymerase reaction (Figure S24A,B). A similar arrangement is also likely present in the *Poly­(A) polymerase catalytic subunit–like* SF, although its available structures contain Ca^2+^, precluding direct observation of the native Mg^2+^ coordination and in the *RPB5-like RNA polymerase subunit* SF (Figure S24F).

These enzymes (including those in Figure S24D,E) use the classical two-metal ion catalytic Mg-binding motif.^22,69^ One metal ion (often termed metal A) activates the nucleophile by lowering its p*k*_a_ and stabilizing the developing negative charge during the onset of attack, while the second metal ion (metal B) stabilizes the leaving group. In reactions involving poor leaving groups, such as the ribose 3′-oxygen in RNase H and related enzymes, the second metal is essential for lowering the barrier associated with bond cleavage. Polymerases follow a similar logic: metal A facilitates deprotonation and alignment of the 3′–OH of the primer terminus, whereas metal B stabilizes the pyrophosphate leaving group generated during nucleotide incorporation. Our systematic analysis shows that the Mg-pinch–forming ion maps onto the role of metal B, weakening the scissile P–O bond via polarization of the triphosphate chain. This pinching motif represents a unifying structural signature by which diverse folds achieve analogous charge stabilization during catalysis, even though the surrounding protein residues differ substantially.

Beyond the classical two-metal catalysis, several polymerases and nucleases have been proposed to transiently recruit a third divalent metal ion, either during catalysis or product release. While such ions have been observed crystallographically (Figure S24C), their catalytic necessity remains debated.^70,71^ Our previous work showed that, although a third metal can modulate electrostatics along the reaction coordinate, it is not required for achieving the chemical step of phosphoryl transfer.^43^ Instead, its role appears auxiliary—promoting product departure or reshaping the electrostatic environment following bond cleavage. Consistent with this view, the catalytic role of a third metal ion remains unresolved,^72^ and crystallographic and kinetic studies indicate that it is not obligatorily present in higher-fidelity polymerases.^73^ Moreover, time-resolved and kinetic analyses have shown that metal-ion binding can be highly dynamic and sequential, suggesting that transient metal occupancy—rather than static three-metal configurations—may influence catalysis and product release.^73,74^ The present structural survey reinforces this interpretation: three-metal configurations occur in various positions, typically not coinciding with the conserved Mg-pinch arrangements.

Identical metal coordination (at the BG(−), Figure 3) site and an additional αγ-coordinated ion (Figure S4) is also presented by SFs that include the ATP-grasp motif SFs (Figure S5). All belong to different folds (d.142–144 and f.62), nevertheless the metal ion coordination shares structural similarities. However, the *Diacylglycerol kinase (DgkA)-like* SF is structurally different from the others, yet its NTP coordination strikingly resembles the rest of the group (Figure S5B). Interestingly, there is a carboxylate residue that coordinates both Mg^2+^ ions in a conserved position with respect to the NTP for all four SFs, which either resides on a β-sheet (*Glutathione synthetase ATP-binding domain-like*), on an α-helix (*Diacylglycerol kinase (DgkA)-like*) or on a loop (*Protein kinase-like (PK-like)* and *SAICAR synthase-like*). This same coordination mode is also present in the *Inositol-pentakisphosphate 2-kinase* representative structure. ATP-grasp enzymes are known to use a dual-metal strategy in which one Mg^2+^ gates substrate positioning while the second stabilizes the acylphosphate intermediate during phosphoryl transfer.^75^ This mechanistic requirement explains why these folds consistently recruit additional catalytic metal ions.^39^ Consequently, the Mg^2+^ coordination geometries seem to be determined by the reaction mechanism rather than the fold itself.

### 2.9 Analysis of the EC Category Distributions

We analyzed the distribution of the identified 759 NTP processing EC categories (Figure 1C) in terms of the structural motifs found. ECs are unevenly covered by the identified 90 SFs, the most common eight SFs perform more than 65% (∼496/759) of the NTP-processing EC reactions, with the *P-loop NTPase*, *Acetyl-CoA synthetase-like*, *PK-like*, and *Glutathione synthetase ATP-binding domain-like* SFs alone accounting for ∼43% of EC categories (Figure S25). When these EC and SF totals are broken down by Mg-pinch coordination type (Figure S26), the picture becomes more skewed still: a single coordination geometry βγ(−) is the most common amongst NTP-processing ECs (∼390 of 928 EC–SF associations), again driven primarily by the *P-loop NTPase* SF. We note that assignment of function in terms of EC categories in several databases may be ambiguous and in cases incorrectly linked to SFs, resulting in SFs seemingly performing both phosphatase and pyrophosphatase activities. For example, the phosphatase *PK-like* SF is verifiably associated with adenylyltransferase activity through flipped pseudokinases by Protein adenylyltransferase SelO,^45,46^ although our alignments showed a closer similarity of SelO to the *Aerobactin siderophore biosynthesis, IucA/IucC-like* SF.

The top SFs are often used to fuel molecular machines *via* inorganic phosphate release (*P-loop containing nucleoside triphosphate hydrolases* or *Actin-like ATPase domain*), including AAA+ ATPases.^76,77^ While ATP is by far the most common NTP substrate (Figure S27), some SFs are specific to other nucleosides, e.g., *dUTPase-like* SF for dUTP. The *P-loop containing nucleoside triphosphate hydrolases* SF is identified most frequently, in nearly 150 EC categories (Figure S25), ubiquitous in all forms of life. While ATP is its most common substrate, *P-loop NTPases* SF also includes GTP processing enzymes, e.g., signaling GTPases.

*Acetyl CoA synthetase-like* and *Glutathione synthetase ATP-binding domain-like* SF enzymes are involved in biosynthetic pathways and specifically use ATP. *Acetyl CoA synthetase-like* is abundant among carbon–sulfur ligases (6.2.-.- EC subclasses), typically with a carboxylate nucleophile being adenylated. *Glutathione synthetase ATP-binding domain-like* SF enzymes are phosphatases on the other hand, mostly found among peptide ligases (6.3.-.- EC subclasses).

Kinases of the *Protein kinase-like* and *Actin-like ATPase domain* SFs also cover many EC categories, as they are often associated with distinct ECs corresponding to a range of nucleophiles being phosphorylated.

For a given function, the same structure with a single SF is typically used, and the distribution of the number of SFs present in the same ECs is far less ambiguous. Only nine EC categories are linked to four or more SFs (SI Table S4). The most extreme case is the generic EC 2.7.11.1 (nonspecific serine/threonine protein kinase) which is propagated to 11 different SFs in our data set—a number that reflects database promiscuity in assigning a catch-all kinase identifier across structurally distinct kinase folds rather than genuinely diverse mechanisms. Among the polymerase-associated ECs, 2.7.7.48 (RNA-directed RNA polymerase) is the most structurally diverse, spanning six SFs. While the *DNA/RNA polymerases* SF, with its many members, covers all organisms from viruses to mammals, other SFs were identified with the same functionality but specific for e.g., negative-sense RNA viruses (*Virion DNA-directed RNA polymerase domain* or *Influenza RNA-dependent RNA polymerase subunit PB1*). The DNA-directed DNA polymerase EC (2.7.7.7) is associated with three SFs.

### 2.10 The Effect of Mg^2+^ Analyzed through QM/MM and QM Calculations

We analyze the catalytic contribution of Mg^2+^ using three prototype phosphate-cleaving enzymes: Ras, dUTPase, and the ribonuclease H (RNase H).^78–80^ Ras is a GTPase, which belongs to the largest identified superfamily, the *P-loop containing NTP hydrolases*, and serves as a representative model for phosphatase enzymes. A representative of the *dUTPase-like* fold, dUTPase is our prototype for a pyrophosphatase enzyme. To provide an additional comparison outside canonical NTP processing, we also included RNase H, a phosphate-cleaving enzyme that uses classical two-metal-ion catalysis to cleave RNA. The RNase H active site shares similarities with DNA/RNA polymerases,^8,80^ hosting two Mg^2+^ ions by conserved carboxylate motifs. While polymerases incorporate new bases from NTPs into the nucleic acid chain, ribonucleases are cleaving such chains to produce 3′–OH and 5′-P-terminated products (Figure S28).

For these systems, the essentiality of Mg^2+^ is well established experimentally. In dUTPase, Mg^2+^ enhances dUTP binding 100-fold and dramatically increases *k*_cat_, activity is abolished by EDTA chelation; neither Na^+^ nor K^+^ can substitute.^81^ In Ras, Mg^2+^ is required for GTP hydrolysis: Mg^2+^ chelation by EDTA abolishes intrinsic hydrolysis,^82^ and mutations that disrupt the Mg^2+^ coordination site (e.g., Thr35Ala) abolish GTPase activity.^83^ In RNase H, Ca^2+^ can occupy the active site but does not support catalysis, consistent with QM/MM calculations showing a ∼3–4 kcal·mol^–1^ higher barrier for Ca^2+^ relative to Mg^2+^.^10^

To quantify the catalytic contribution of Mg^2+^ computationally, we repeated the previous pathway calculations using the same optimized geometries but with the probed Mg^2+^ site replaced by Na^+^ or K^+^ preserving coordination, or removed. These calculations were designed to probe the electronic consequences of altering the catalytic ion within fixed reaction pathways and they are therefore most useful for identifying plausible electrostatic and polarization contributions, rather than for providing fully relaxed alternative catalytic mechanisms. Within this fixed-path framework, changes in the computed barriers primarily report on the electronic consequences of changing or removing the catalytic ion, while Na^+^ and K^+^ substitutions partly preserve the local coordination environment. The treatment of Mg^2+^ ions and their coordination shell is crucial for accurate reaction mechanisms, as well as for structural roles.^67,84,85^ For this reason, Mg^2+^ and its first coordination shell were included in all three pathways together with key H-bonding networks, explicitly including also water molecules (SI Section 1, Table S1).

In the fixed-geometry QM/MM models, perturbing or removing the catalytic Mg^2+^ site substantially increases the computed activation barriers in all three systems (Figure 6A). For Ras, removal of Mg^2+^ increases the barrier for the rate-limiting step from 21.4 to 36.4 kcal mol^–1^. For dUTPase, removal of the Mg-pinch-forming αβγ-coordinated Mg^2+^ increases the barrier from 17.9 to 39.0 kcal mol^–1^. For RNase H, removal of the Mg^2+^ ion associated with nucleophile activation increases the barrier from 23.8 to 46.7 kcal mol^–1^. Because these calculations use fixed reaction paths without full reoptimization after ion substitution or removal, the absolute magnitude of these changes should not be interpreted as a direct experimental prediction. Instead, the consistent barrier increase highlights the large electronic cost of removing an appropriately coordinated divalent ion from the catalytic geometry.

**Figure 6:**
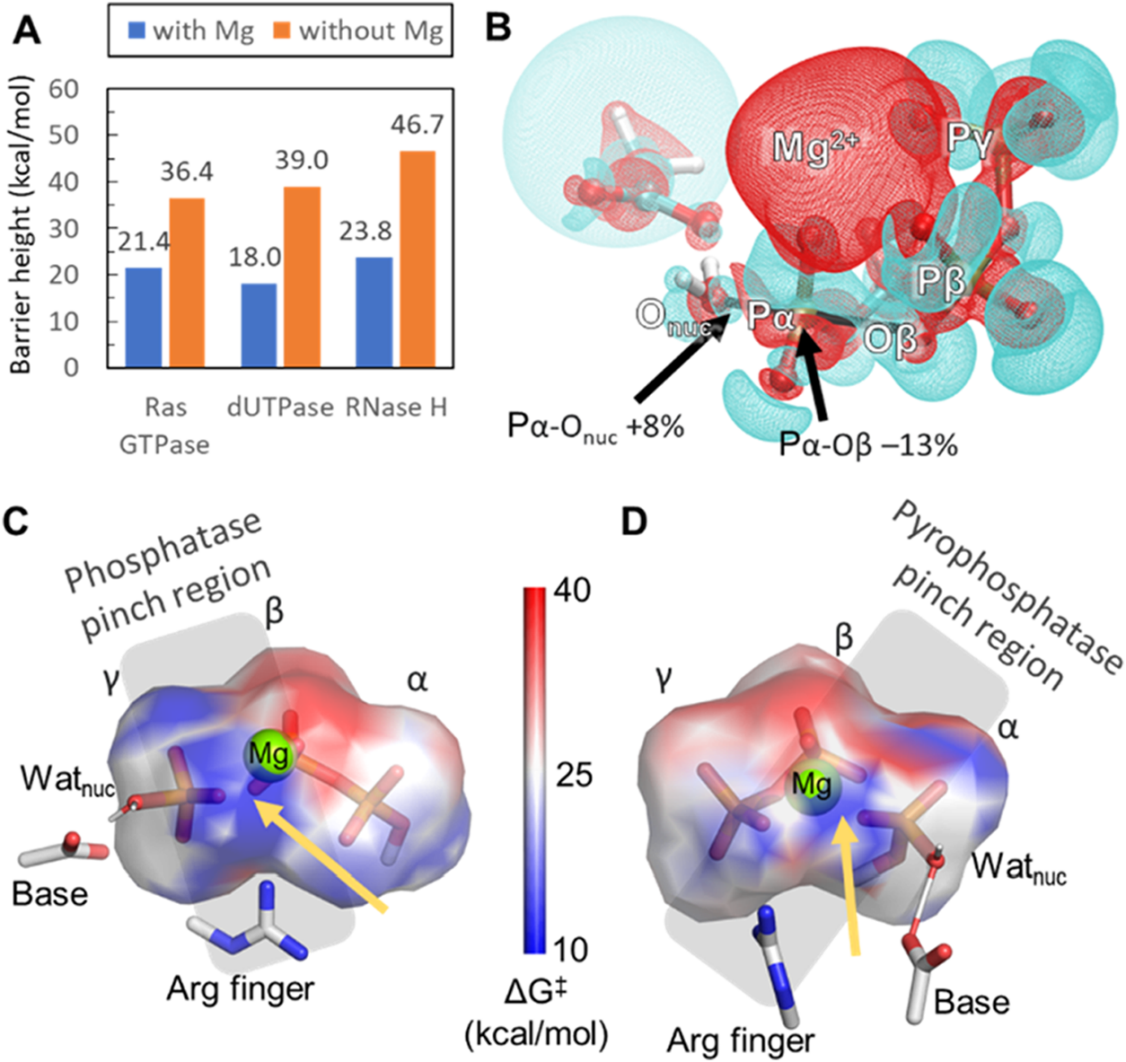
(A) Activation energies in the presence and absence of the catalytic Mg^2+^ ion in QM/MM simulation of three phosphate catalytic enzymes: Ras, dUTPase, and RNase H. (B) Isosurface of the electron density change upon the introduction of a 2+ point charge at the Mg^2+^ position for the dUTPase model system. Increased density is depicted in red, decreased in cyan. The changes in the forming and breaking P–O bonds (black lines) are highlighted by arrows. Map of the 2+ charge positions around the triphosphate colored by the activation energy to carry out phosphate (C), pyrophosphate (D) hydrolysis. The approximate region where the cation can form a Mg-pinch is highlighted in gray, optimal position indicated by yellow arrows. The original Mg^2+^ (green spheres) and arginine fingers (white sticks) are depicted for orientation only.

Additional QM calculations were also carried out on minimal phosphate catalytic QM models, in which the catalytic effect of Mg^2+^ was approximated by placing a +2 point charge at the metal position (Figure S29). This setup allows isolation of the purely electrostatic and polarization contributions of the ion while keeping the geometry fixed. Systematic variation of the point charge value shows that increasing positive charge lowers the activation barrier in both the phosphatase (Pi) and pyrophosphatase (PPi) models (Figure S29), consistent with the trends observed in the full QM/MM enzyme systems (Tables S6–S8). To examine the electronic origin of this effect, we analyzed density-difference maps (Figure 6B; Figures S30–S31), Wiberg bond indices (Figures S30–S31), and an electrostatic/polarization decomposition of the model barriers (Table S5; Figure S32). Introduction of the positive charge induces redistribution of electron density within the triphosphate group, including changes along the forming and breaking P–O bonds. However, the associated Wiberg bond-index changes are modest and do not scale uniformly with the barrier reduction, indicating that bond-order descriptors alone do not capture the full catalytic effect. The electrostatic/polarization decomposition provides a clearer interpretation: polarization contributes substantially to transition-state stabilization, although the balance between frozen electrostatics and polarization differs between the Pi and PPi model systems (Table S5; Figure S32). Thus, these model calculations support the view that an appropriately positioned divalent positive charge can stabilize phosphate cleavage through a combination of electrostatic preorganization and electronic polarization.

We further evaluated the dependence of the catalytic effect on the position of the positive charge by scanning its location around the triphosphate (Figure 6C–D). The resulting qualitative activation-energy maps identify a region where positive charge is especially stabilizing. This region is centered around the cleaved P–O bond and overlaps with the experimentally observed Mg-pinch positions. It also overlaps with the positions of arginine fingers, suggesting that metal ions and positively charged residues can exploit a similar electrostatic geometry to stabilize the reacting phosphate group.

To further dissect the origin of the catalytic effect on the full QM/MM systems, we also quantified how ion substitution with Na^+^ or K^+^ alters the electronic structure along the reaction coordinate using ESP-based charge analysis, dipole metrics, and bond-order descriptors (SI Tables S9–S22). We also verified that higher level treatment of the QM region with the dispersion-corrected range-separated hybrid functional (ωB97M-V/def2-TZVP), gives the same trends and reliance on the Mg^2+^ ion for reactivity (SI Tables S6–S8, Figures S33–S34).

We explored multiple descriptors to understand the changes in catalytic effects. Atomic charges and fragment charge-separation metrics such as *Q*_diff_(P–L) = *Q*_phos_ – *Q*_lg_, where *Q*_phos_ is the summed ESP charge of the reacting phosphate fragment and *Q*_lg_ is the summed ESP charge of the leaving-group fragment, report the charge imbalance between the phosphate side and the departing group. *Q*_diff_(P–L) exhibits system-dependent behavior (Tables S9–S11; Figure S35), with Ras notably showing near-zero Δ*Q*_diff_ for Mg^2+^ but larger values for monovalent ions and the ion-free case, despite Mg^2+^ providing one of the lowest barriers. We also note that Ras with K^+^ in the active site is the only exception where the barrier is lower than with Mg^2+^, and we treat this as an anomaly.

Analysis of dipole properties (Tables S12–S15; Figure S36) shows that while the total dipole magnitude of the QM region is strongly affected by ion identity (Table S13), the projected dipole change along the reaction coordinate is nearly invariant within each system (Tables S13–S14), and the magnitude of the dipole-change vector varies only weakly across ions (Table S15). While individual Wiberg bond orders alone are not descriptive, changes in Wiberg bond orders along the reaction coordinate are informative (Table S16; Figure S37): the increase in the forming P–O_nuc_ bond order and the decrease in the breaking P–O_LG_ bond order both track the barrier trends well, especially for dUTPase and RNase H.

We further analyzed metal-centered and local-field descriptors as well as the electrostatic potential at the reacting phosphorus, Φ­(P), represented via atom-centered Coulomb potential from the ESP fitted partial charges of the nonreacting QM atoms (Tables S17–S20, Figures S38–S39). The electrostatic potential at the reacting phosphorus, Φ­(P), already distinguishes the ion states in the reactant complex and tracks the QM/MM barriers across all three enzymes (SI Figure S39), showing that Mg^2+^ preorganizes the phosphate electronically before bond reorganization begins. Consistent with this picture, the NBO LP­(O) → M and LP­(O) → P* interactions are generally strongest for Mg^2+^ and weaker for Na^+^ and K^+^ (SI Tables S17–S19; Figure S38), in line with its greater Lewis acidity. Additional leaving-group-side NBO channels show that this stabilization is enzyme-specific: dUTPase and Ras use strong local N–H donor networks, whereas RNase H is dominated by direct LP­(O3′) → LP*­(Mg) coordination at the leaving-group-side metal (Table S20). Together, these analyses suggest that Mg^2+^ does not act simply as a structural cofactor or a generic positive charge, but instead lowers the barrier by creating the most activating electrostatic environment at phosphorus and by enhancing donor–acceptor delocalization from the reacting oxygen ligands into metal- and phosphorus-centered acceptor orbitals.

Descriptors that explicitly capture the proton-transfer component of the reaction also show strong and systematic relationships with the activation barriers (Tables S21–S22). Decomposition of the nucleophilic water into the transferred proton and the remaining OH fragment shows that the ESP charge of the OH fragment, q­(OH) at the transition state correlates strongly with the QM/MM barriers, with Pearson correlation coefficients of *r* = −0.92 for dUTPase, *r* = −1.00 for RNase H, and *r* = −0.85 for Ras. Thus, higher barriers are associated with a more negative residual OH fragment at the TS, consistent with less effective stabilization of the developing proton-transfer. Importantly, this OH-fragment polarization is not displayed at the RS: q­(OH) at the RS shows weak correlation with barrier height across the three enzyme systems (Figure S40), even though the formal ion charge differs by up to two units. This rules out a simple charge-induced static effect on the nucleophile. Only at the TS does q­(OH) track the barrier, establishing that this polarization is dynamical in nature—driven by the direct electronic influence of the ion on the charge redistribution occurring during bond formation and proton transfer. Complementary descriptors based on charge redistribution between the transferred proton and the acceptor group (Tables S17–S19) support the same conclusion: metal substitution primarily perturbs the coupled proton-transfer/phosphate-cleavage polarization, rather than simply changing phosphate bond cleavage alone.

Taken together, these results support a two-component mechanistic model for Mg^2+^ catalysis. First, the ion preorganizes the electrostatic environment at phosphorus already in the reactant state, lowering the intrinsic electrophilicity barrier for nucleophilic attack (Figure S39). Second, at the transition state, Mg^2+^ dynamically polarizes the nucleophilic OH fragment, driving charge redistribution into the forming P–O bond during the coupled proton transfer (Figure S40). Both components are absent or diminished for monovalent substitutes. This is also consistent with extensive experimental observations that NTP-processing enzymes typically require physiological Mg^2+^ concentrations for activity, supporting the conclusion that this two-component electrostatic preorganization and dynamical polarization mechanism is a relevant feature in the representative systems studied here.^9,81,83,86–90^

## 3 Conclusions

Phosphate chemistry is at the center of biological processes. Triphosphates have a unique dominating role in all forms of biological processes, as NTPs are used most frequently as reactants of phosphate processing enzyme reactions. We classify two major categories of NTP reactivity: phosphatase and pyrophosphatase activities. Both reactivities require metal cations for activation, predominantly Mg^2+^ functions as the catalytic divalent ion.

We hypothesized that the metal ion coordination has a specific structural requirement for its functional role in the catalytic mechanism. In particular, the Mg^2+^ ion should be coordinated by the phosphates that are involved in the cleaved P–O bond, ie. for phosphatases at least one Mg^2+^ must show a BG (or ABG) coordination while for pyrophosphatases AB (or ABG) coordination is expected. To test our hypothesis, as well as to enable analysis of future structure–function relationships, we built a comprehensive structural database of distinct NTP processing enzyme superfamilies. We associated SCOPe superfamilies for each NTP processing enzyme when this was possible, and identified InterPro IDs otherwise. We compared the active site geometries within each superfamily using currently available structural data, and highlighted challenging cases where this is currently missing.

We analyzed the experimental structures of enzyme-NTP-cation complexes by their Mg—triphosphate chain interaction and position. We established that the members of a superfamily exhibit a consensus binding mode including conserved motifs of the ion coordination of protein residues. Furthermore, the NTP–Mg^2+^ coordination reveals a dominant Mg-pinch motif across structurally characterized superfamilies, in which the divalent ion is positioned to coordinate the phosphate groups flanking the cleaved P–O bond in phosphatases and pyrophosphatases.

In total, we identified 90 NTP-processing SFs, comprising 42 phosphatases (Pi-releasing), 44 pyrophosphatases (PPi-releasing), and 4 triphosphatases. Among the phosphatases, 32 SFs exhibit a well-defined Mg-pinch coordination, while one promiscuous SF does not rely on Mg^2+^ for catalysis. The remaining phosphatase SFs lack sufficient structural information to unambiguously define the catalytic metal-ion geometry. Within the pyrophosphatase group, 32 SFs also display a clear Mg-pinch motif, with one promiscuous SF that does not employ Mg^2+^ and a single genuine exception—the Acetyl-CoA synthetase-like SF—which uses Mg^2+^ but deviates from the canonical pinch configuration. Across both Pi- and PPi-releasing enzymes, a further 19 SFs (9 phosphatases and 10 pyrophosphatases) could not be conclusively classified due to limited or incomplete structural data. Of these, three phosphatase SFs are currently defined only at the InterPro family level without an associated SCOPe SF, and ten pyrophosphatase SFs lack SCOPe classification, although six of them nonetheless show well-defined metal coordination in available structures. Together, these results show that the Mg-pinch motif is a dominant and conserved structural feature of currently characterized NTP-processing enzymes, with rare, mechanistically informative exceptions.

Our analysis of NTP binding sites provides templates for less studied enzymes, when only their reactivity (registered as an EC number) or their sequence information (through assigned superfamilies) is known, as well as if the available structures are incomplete (e.g., missing substrates or ions). Indeed, several examples we identified correspond to cases where nucleoside binding prediction is inaccurate even using e.g., AlphaFold 3, despite the experimentally available data. The established structural templates and insights into the Mg-pinch motif can be used in simulations to study enzyme reactivity and guide the development of new inhibitors or activators.

The catalytic role of Mg^2+^ is also supported by our QM and QM/MM calculations. The model charge-probe analysis shows that barrier lowering is favored when positive charge is placed near the pinching positions around the cleaved P–O bond. In the enzyme QM/MM systems, removal or substitution of Mg^2+^ increases the barriers in most cases, even when the coordination environment is partly preserved by monovalent ions. These observations suggest that coordination geometry alone is insufficient and that the electronic properties of an appropriately coordinated Mg^2+^ ion contributes to catalysis. Our results indicate that the key contribution of the Mg-pinch motif is electronic: Mg^2+^ preorganizes the reacting phosphate through a strongly activating electrostatic field and, at the transition state, drives the dynamical polarization and charge redistribution required for the catalytic process. Monovalent ions such as Na^+^ and K^+^ do not consistently reproduce these effects, despite largely preserving the coordination geometry, consistent with their lower charge density and weaker ability to polarize coordinating ligands. Thus, the Mg-pinch motif can contribute to polarization-driven catalytic elements that combine reactant-state electrostatic preorganization with transition-state-specific dynamical polarization to enable efficient catalysis.^91^

Collectively, these observations show that neither uncatalyzed aqueous NTP hydrolysis nor known RNA-based catalysts adequately mimic the highly organized NTP coordination and hydrolysis achieved by protein enzymes. Protein enzymes create specific binding sites that position the nucleotide, organize one or more metal ions, and supply appropriately placed acid–base groups to achieve efficient and selective phosphoanhydride cleavage. By contrast, ribozymes that rely on multiple Mg^2+^ ions typically act on RNA backbones (e.g., self-cleavage or ligation) and do not employ NTP substrates as broadly as protein enzymes do in metabolism, signaling, and energy transduction. Thus, the combination that is characteristic of protein systems—tight NTP coordination, Mg-pinch-assisted catalysis, and pervasive functional use of NTPs—is a unique characteristic of protein catalysts.

Our comprehensive database matches reactivity with structural alignment, advancing our understanding of these ubiquitous biological processes. The insights into phosphate chemistry and NTP processing can contribute to a deeper understanding of fundamental biological mechanisms. The structural database and analysis of NTP processing enzymes can also inform the design of new drugs targeting these enzymes, which are crucial for many diseases, as well as help identify off-target toxicity for NTP-analogue inhibitors and inform protein engineering efforts to enhance or modify the activity of NTP processing enzymes.

## Supplementary Material



## Notes

Methods and data collection; superfamily assignments and representative structures; additional structural alignments and ion-cluster analyses; EC-category and domain-length analyses; QM/MM and QM model construction and computational details; supplementary figures and tables; model geometries and coordinates (PDF)

## References

[1] Westheimer F. H. (1987). Why nature
chose phosphates. Science.

[2] Kamerlin S. C. L., Sharma P. K., Prasad R. B., Warshel A. (2013). Why nature really chose
phosphate. Q. Rev. Biophys..

[3] Shepard S. M., Jessen H. J., Cummins C. C. (2022). Beyond
Triphosphates: Reagents and
Methods for Chemical Oligophosphorylation. J.
Am. Chem. Soc..

[4] Jakubowski H. (1986). Sporulation
of the yeast Saccharomyces cerevisiae is accompanied by synthesis
of adenosine 5′-tetraphosphate and adenosine 5′-pentaphosphate. Proc. Natl. Acad. Sci. U.S.A..

[5] Azevedo C., Livermore T., Saiardi A. (2015). Protein polyphosphorylation of lysine
residues by inorganic polyphosphate. Mol. Cell.

[6] Ekesan Ş., McCarthy E., Case D. A., York D. M. (2022). RNA Electrostatics:
How Ribozymes Engineer Active Sites to Enable Catalysis. J. Phys. Chem. B.

[7] Cowan J. A. (2002). Structural
and catalytic chemistry of magnesium-dependent enzymes. BioMetals.

[8] Yang W., Lee J. Y., Nowotny M. (2006). Making and breaking nucleic acids:
two-Mg^2+^-ion catalysis and substrate specificity. Mol. Cell.

[9] Knape M. J., Ahuja L. G., Bertinetti D., Burghardt N. C., Zimmermann B., Taylor S. S., Herberg F. W. (2015). Divalent
Metal Ions
Mg(2)­(+) and Ca(2)­(+) Have Distinct Effects on Protein Kinase A Activity
and Regulation. ACS Chem. Biol..

[10] Rosta E., Yang W., Hummer G. (2014). Calcium inhibition of ribonuclease
H1 two-metal ion catalysis. J. Am. Chem. Soc..

[11] Maguire M.
E., Cowan J. A. (2002). Magnesium
chemistry and biochemistry. BioMetals.

[12] Krzywoszyńska K., Witkowska D., Swiatek-Kozlowska J., Szebesczyk A., Kozlowski H. (2020). General Aspects of Metal Ions as Signaling Agents in
Health and Disease. Biomolecules.

[13] Son T.-D., Roux M., Ellenberger M. (1975). Interaction of Mg^2+^ ions
with nucleoside triphosphates by phosphorus magnetic resonance spectroscopy. Nucleic Acids Res..

[14] Huang S. L., Tsai M. D. (1982). Does the magnesium­(II)
ion interact with the alpha-phosphate
of adenosine triphosphate? An investigation by oxygen-17 nuclear magnetic
resonance. Biochemistry.

[15] Takeuchi H., Murata H., Harada I. (1988). Interaction
of adenosine 5′-triphosphate
with Mg^2+^: vibrational study of coordination sites by use
of 18O-labeled triphosphates. J. Am. Chem. Soc..

[16] Mudryk K., Lee C., Tomaník L., Malerz S., Trinter F., Hergenhahn U., Neumark D. M., Slavíček P., Bradforth S., Winter B. (2024). How Does Mg^2+^(aq) Interact
with ATP­(aq)? Biomolecular Structure through the Lens of Liquid-Jet
Photoemission Spectroscopy. J. Am. Chem. Soc..

[17] Chen K., Mizianty M. J., Kurgan L. (2012). Prediction and analysis of nucleotide-binding
residues using sequence and sequence-derived structural descriptors. Bioinformatics.

[18] Hu J., Li Y., Zhang Y., Yu D. J. (2018). ATPbind: Accurate Protein-ATP Binding
Site Prediction by Combining Sequence-Profiling and Structure-Based
Comparisons. J. Chem. Inf Model.

[19] Abramson J., Adler J., Dunger J., Evans R., Green T., Pritzel A., Ronneberger O., Willmore L., Ballard A. J., Bambrick J., Bodenstein S. W., Evans D. A., Hung C. C., O′Neill M., Reiman D., Tunyasuvunakool K., Wu Z., Zemgulyte A., Arvaniti E., Beattie C., Bertolli O., Bridgland A., Cherepanov A., Congreve M., Cowen-Rivers A. I., Cowie A., Figurnov M., Fuchs F. B., Gladman H., Jain R., Khan Y. A., Low C. M. R., Perlin K., Potapenko A., Savy P., Singh S., Stecula A., Thillaisundaram A., Tong C., Yakneen S., Zhong E. D., Zielinski M., Zidek A., Bapst V., Kohli P., Jaderberg M., Hassabis D., Jumper J. M. (2024). Accurate structure
prediction of biomolecular interactions with AlphaFold 3. Nature.

[20] Gao Y., Yang W. (2016). Capture of a third Mg(2)­(+) is essential for catalyzing
DNA synthesis. Science.

[21] Yang W. (2008). An equivalent
metal ion in one- and two-metal-ion catalysis. Nat. Struct Mol. Biol..

[22] Steitz T. A., Steitz J. A. (1993). A general two-metal-ion
mechanism for catalytic RNA. Proc. Natl. Acad.
Sci. U.S.A..

[23] Berman H. M., Westbrook J., Feng Z., Gilliland G., Bhat T. N., Weissig H., Shindyalov I. N., Bourne P. E. (2000). The Protein Data Bank. Nucleic
Acids Res..

[24] Kanehisa, M. Enzyme Annotation and Metabolic Reconstruction Using KEGG. In Methods in Molecular Biology; Springer, 2017; Vol. 1611, pp 135–145. 28451977 10.1007/978-1-4939-7015-5_11

[25] Kanehisa M., Goto S. (2000). KEGG: kyoto encyclopedia of genes and genomes. Nucleic Acids Res..

[26] Kanehisa M. (2019). Toward understanding
the origin and evolution of cellular organisms. Protein Sci..

[27] Kanehisa M., Furumichi M., Sato Y., Kawashima M., Ishiguro-Watanabe M. (2023). KEGG for taxonomy-based analysis of pathways and genomes. Nucleic Acids Res..

[28] Paysan-Lafosse T., Blum M., Chuguransky S., Grego T., Pinto B. L., Salazar G. A., Bileschi M. L., Bork P., Bridge A., Colwell L., Gough J., Haft D. H., Letunic I., Marchler-Bauer A., Mi H., Natale D. A., Orengo C. A., Pandurangan A. P., Rivoire C., Sigrist C. J. A., Sillitoe I., Thanki N., Thomas P. D., Tosatto S. C. E., Wu C. H., Bateman A. (2023). InterPro in
2022. Nucleic Acids
Res..

[29] Gasteiger E., Gattiker A., Hoogland C., Ivanyi I., Appel R. D., Bairoch A. (2003). ExPASy: The proteomics server for in-depth protein
knowledge and analysis. Nucleic Acids Res..

[30] Gough J., Karplus K., Hughey R., Chothia C. (2001). Assignment of homology
to genome sequences using a library of hidden Markov models that represent
all proteins of known structure. J. Mol. Biol..

[31] Pandurangan A. P., Stahlhacke J., Oates M. E., Smithers B., Gough J. (2019). The SUPERFAMILY
2.0 database: a significant proteome update and a new webserver. Nucleic Acids Res..

[32] Fox N. K., Brenner S. E., Chandonia J. M. (2014). SCOPe:
Structural Classification
of Proteins--extended, integrating SCOP and ASTRAL data and classification
of new structures. Nucleic Acids Res..

[33] Chandonia J. M., Guan L., Lin S., Yu C., Fox N. K., Brenner S. E. (2022). SCOPe: improvements to the structural classification
of proteins - extended database to facilitate variant interpretation
and machine learning. Nucleic Acids Res..

[34] Schaeffer R. D., Medvedev K. E., Andreeva A., Chuguransky S. R., Pinto B. L., Zhang J., Cong Q., Bateman A., Grishin N. V. (2025). ECOD: integrating classifications of protein domains
from experimental and predicted structures. Nucleic Acids Res..

[35] Schaeffer R. D., Liao Y., Cheng H., Grishin N. V. (2017). ECOD: new developments
in the evolutionary classification of domains. Nucleic Acids Res..

[36] Cheng H., Schaeffer R. D., Liao Y., Kinch L. N., Pei J., Shi S., Kim B. H., Grishin N. V. (2014). ECOD: an evolutionary classification
of protein domains. PLoS Comput. Biol..

[37] Holm L., Laiho A., Toronen P., Salgado M. (2023). DALI shines
a light
on remote homologs: One hundred discoveries. Protein Sci..

[38] González B., Banos-Sanz J. I., Villate M., Brearley C. A., Sanz-Aparicio J. (2010). Inositol 1,3,4,5,6-pentakisphosphate
2-kinase is a distant IPK member with a singular inositide binding
site for axial 2-OH recognition. Proc. Natl.
Acad. Sci. U.S.A..

[39] Fawaz M. V., Topper M. E., Firestine S. M. (2011). The ATP-grasp
enzymes. Bioorg. Chem..

[40] Siebold C., Arnold I., Garcia-Alles L. F., Baumann U., Erni B. (2003). Crystal structure
of the Citrobacter freundii dihydroxyacetone kinase reveals an eight-stranded
alpha-helical barrel ATP-binding domain. J.
Biol. Chem..

[41] Dunbar K. L., Melby J. O., Mitchell D. A. (2012). YcaO domains use ATP to activate
amide backbones during peptide cyclodehydrations. Nat. Chem. Biol..

[42] Xiang Y., Leiman P. G., Li L., Grimes S., Anderson D. L., Rossmann M. G. (2009). Crystallographic Insights into the Autocatalytic Assembly
Mechanism of a Bacteriophage Tail Spike. Mol.
Cell.

[43] Berta D., Buigues P. J., Badaoui M., Rosta E. (2020). Cations in
motion:
QM/MM studies of the dynamic and electrostatic roles of H­(+) and Mg­(2+)
ions in enzyme reactions. Curr. Opin. Struct.
Biol..

[44] Pitcher R. S., Brissett N. C., Picher A. J., Andrade P., Juarez R., Thompson D., Fox G. C., Blanco L., Doherty A. J. (2007). Structure
and function of a mycobacterial NHEJ. DNA repair polymerase. J. Mol. Biol..

[45] Bardwell L. (2019). Pseudokinases:
Flipping the ATP for AMPylation. Curr. Biol..

[46] Sreelatha A., Yee S. S., Lopez V. A., Park B. C., Kinch L. N., Pilch S., Servage K. A., Zhang J., Jiou J., Karasiewicz-Urbanska M., Lobocka M., Grishin N. V., Orth K., Kucharczyk R., Pawlowski K., Tomchick D. R., Tagliabracci V. S. (2018). Protein
AMPylation by an Evolutionarily Conserved Pseudokinase. Cell.

[47] Gulick A. M., Starai V. J., Horswill A. R., Homick K. M., Escalante-Semerena J. C. (2003). The 1.75
A crystal structure of acetyl-CoA synthetase bound to adenosine-5′-propylphosphate
and coenzyme A. Biochemistry.

[48] Deng J., Schnaufer A., Salavati R., Stuart K. D., Hol W. G. J. (2004). High
Resolution Crystal Structure of a Key Editosome Enzyme from Trypanosoma
brucei: RNA Editing Ligase 1. J. Mol. Biol..

[49] Cherepanov A. V., de Vries S. (2002). Kinetic Mechanism of the Mg2+-dependent Nucleotidyl
Transfer Catalyzed by T4 DNA and RNA Ligases. J. Biol. Chem..

[50] Shuman S., Goldgur Y., Unciuleac M.-C. (2020). Caveat
mutator: alanine substitutions
for conserved amino acids in RNA ligase elicit unexpected rearrangements
of the active site for lysine adenylylation. Nucleic Acids Res..

[51] Grochowski L. L., Xu H., Leung K., White R. H. (2007). Characterization of an Fe2+-Dependent
Archaeal-Specific GTP Cyclohydrolase, MptA, from Methanocaldococcus
jannaschii. Biochemistry.

[52] Liu S., Wang H., Li X., Zhang F., Lee J. K. J., Li Z., Yu C., Hu J. J., Zhao X., Suematsu T., Alvarez-Cabrera A. L., Liu Q., Zhang L., Huang L., Aphasizheva I., Aphasizhev R., Zhou Z. H. (2023). Structural basis of gRNA stabilization
and mRNA recognition
in trypanosomal RNA editing. Science.

[53] Jourdain A. A., Popow J., de la Fuente M. A., Martinou J. C., Anderson P., Simarro M. (2017). The FASTK family of
proteins: emerging regulators of
mitochondrial RNA biology. Nucleic Acids Res..

[54] Patil A. G.
G., Sang P. B., Govindan A., Varshney U. (2013). Mycobacterium tuberculosis
MutT1 (Rv2985) and ADPRase (Rv1700) proteins constitute a two-stage
mechanism of 8-oxo-dGTP and 8-oxo-GTP detoxification and adenosine
to cytidine mutation avoidance. J. Biol. Chem..

[55] Schweikhard E. S., Kuhlmann S. I., Kunte H. J., Grammann K., Ziegler C. M. (2010). Structure
and function of the universal stress protein TeaD and its role in
regulating the ectoine transporter TeaABC of Halomonas elongata DSM
2581­(T). Biochemistry.

[56] Bangera M., Panigrahi R., Sagurthi S. R., Savithri H. S., Murthy M. R. (2015). Structural
and functional analysis of two universal stress proteins YdaA and
YnaF from Salmonella typhimurium: possible roles in microbial stress
tolerance. J. Struct Biol..

[57] Deng W. H., Lewin H., Liao R. Z., Rosta E. (2025). Reaction Mechanism
and Metal Selectivity of Human SAMHD1 Elucidated by QM/MM Calculations. ACS Catal..

[58] Tanaka N., Chakravarty A. K., Maughan B., Shuman S. (2011). Novel mechanism of
RNA repair by RtcB via sequential 2′,3′-cyclic phosphodiesterase
and 3′-Phosphate/5′-hydroxyl ligation reactions. J. Biol. Chem..

[59] Tokarsky E. J., Wallenmeyer P. C., Phi K. K., Suo Z. (2017). Significant impact
of divalent metal ions on the fidelity, sugar selectivity, and drug
incorporation efficiency of human PrimPol. DNA
Repair.

[60] Li D., Stansfeld P. J., Sansom M. S. P., Keogh A., Vogeley L., Howe N., Lyons J. A., Aragao D., Fromme P., Fromme R., Basu S., Grotjohann I., Kupitz C., Rendek K., Weierstall U., Zatsepin N. A., Cherezov V., Liu W., Bandaru S., English N. J., Gati C., Barty A., Yefanov O., Chapman H. N., Diederichs K., Messerschmidt M., Boutet S., Williams G. J., Marvin
Seibert M., Caffrey M. (2015). Ternary structure reveals mechanism
of a membrane diacylglycerol
kinase. Nat. Commun..

[61] Steegborn C., Litvin T. N., Levin L. R., Buck J., Wu H. (2005). Bicarbonate
activation of adenylyl cyclase via promotion of catalytic active site
closure and metal recruitment. Nat. Struct.
Mol. Biol..

[62] Litvin T. N., Kamenetsky M., Zarifyan A., Buck J., Levin L. R. (2003). Kinetic
properties of ″soluble″ adenylyl cyclase. J. Biol. Chem..

[63] Keppetipola N., Shuman S. (2008). A phosphate-binding
histidine of binuclear metallophosphodiesterase
enzymes is a determinant of 2′,3′-cyclic nucleotide
phosphodiesterase activity. J. Biol. Chem..

[64] Yong S. C., Roversi P., Lillington J., Rodriguez F., Krehenbrink M., Zeldin O. B., Garman E. F., Lea S. M., Berks B. C. (2014). A complex iron-calcium cofactor catalyzing phosphotransfer
chemistry. Science.

[65] Bowman J. C., Lenz T. K., Hud N. V., Williams L. D. (2012). Cations in charge:
magnesium ions in RNA folding and catalysis. Curr. Opin. Struct. Biol..

[66] DeRose V. J. (2003). Metal ion
binding to catalytic RNA molecules. Curr. Opin.
Struct. Biol..

[67] Panteva M. T., Giambasu G. M., York D. M. (2015). Force Field for
Mg­(2+), Mn­(2+), Zn­(2+),
and Cd­(2+) Ions That Have Balanced Interactions with Nucleic Acids. J. Phys. Chem. B.

[68] Klein D. J., Moore P. B., Steitz T. A. (2004). The contribution
of metal ions to
the structural stability of the large ribosomal subunit. RNA.

[69] Steitz T. A. (1998). A mechanism
for all polymerases. Nature.

[70] Nakamura T., Zhao Y., Yamagata Y., Hua Y. J., Yang W. (2012). Watching DNA
polymerase eta make a phosphodiester bond. Nature.

[71] Yang W., Weng P. J., Gao Y. (2016). A new paradigm of DNA synthesis:
three-metal-ion catalysis. Cell Biosci..

[72] Tsai M. D. (2019). Catalytic
mechanism of DNA polymerases-Two metal ions or three?. Protein Sci..

[73] Gong S., Kirmizialtin S., Chang A., Mayfield J. E., Zhang Y. J., Johnson K. A. (2021). Kinetic and thermodynamic analysis defines roles for
two metal ions in DNA polymerase specificity and catalysis. J. Biol. Chem..

[74] Ummat A., Silverstein T. D., Jain R., Buku A., Johnson R. E., Prakash L., Prakash S., Aggarwal A. K. (2012). Human DNA
polymerase
eta is pre-aligned for dNTP binding and catalysis. J. Mol. Biol..

[75] Pederick J. L., Thompson A. P., Bell S. G., Bruning J. B. (2020). d-Alanine-d-alanine
ligase as a model for the activation of ATP-grasp enzymes by monovalent
cations. J. Biol. Chem..

[76] Snider J., Thibault G., Houry W. A. (2008). The AAA+
superfamily of functionally
diverse proteins. Genome Biol..

[77] Jessop M., Felix J., Gutsche I. (2021). AAA+ ATPases:
structural insertions
under the magnifying glass. Curr. Opin. Struct.
Biol..

[78] Berta D., Gehrke S., Nyiri K., Vertessy B. G., Rosta E. (2023). Mechanism-Based
Redesign of GAP to Activate Oncogenic Ras. J.
Am. Chem. Soc..

[79] Lopata A., Jambrina P. G., Sharma P. K., Brooks B. R., Toth J., Vertessy B. G., Rosta E. (2015). Mutations
Decouple Proton Transfer
from Phosphate Cleavage in the dUTPase Catalytic Reaction. ACS Catal..

[80] Dürr S. L., Bohuszewicz O., Berta D., Suardiaz R., Jambrina P. G., Peter C., Shao Y., Rosta E. (2021). The Role of Conserved
Residues in the DEDDh Motif: the Proton-Transfer Mechanism of HIV-1
RNase H. ACS Catal..

[81] Mustafi D., Bekesi A., Vertessy B. G., Makinen M. W. (2003). Catalytic and structural
role of the metal ion in dUTP pyrophosphatase. Proc. Natl. Acad. Sci. U.S.A..

[82] Vish K. J., Paul M. E., Rollins A. P., Boggon T. J. (2025). Optimized conditions
for GTP loading of Ras. J. Biol. Chem..

[83] Farnsworth C. L., Feig L. A. (1991). Dominant inhibitory mutations in the Mg­(2+)-binding
site of RasH prevent its activation by GTP. Mol. Cell. Biol..

[84] Duarte F., Bauer P., Barrozo A., Amrein B. A., Purg M., Aqvist J., Kamerlin S. C. (2014). Force field independent metal parameters
using a nonbonded dummy model. J. Phys. Chem.
B.

[85] Ȧqvist J. (1990). Ion-water
interaction potentials derived from free energy perturbation simulations. J. Phys. Chem. A.

[86] Elnatan D., Betegon M., Liu Y., Ramelot T., Kennedy M. A., Agard D. A. (2017). Symmetry broken
and rebroken during the ATP hydrolysis
cycle of the mitochondrial Hsp90 TRAP1. eLife.

[87] Keum Y. S., Jeong Y. J. (2012). Development of chemical inhibitors of the SARS coronavirus:
viral helicase as a potential target. Biochem.
Pharmacol..

[88] Apell H. J., Hitzler T., Schreiber G. (2017). Modulation
of the Na,K-ATPase by
Magnesium Ions. Biochemistry.

[89] Taylor M. R., Conrad J. A., Wahl D., O′Brien P. J. (2011). Kinetic
mechanism of human DNA ligase I reveals magnesium-dependent changes
in the rate-limiting step that compromise ligation efficiency. J. Biol. Chem..

[90] Frieden C. (1983). Polymerization
of actin: mechanism of the Mg2+-induced process at pH 8 and 20 degrees
C. Proc. Natl. Acad. Sci. U.S.A..

[91] Rosta, E. QM/MM Free Energy Calculations for Enzyme Catalytic Reactions. In Hungarian Quantum Chemistry: Part B - Contemporary Research: Part B - Contemporary Research; Surjan, P. ; Szabados, A. , Eds.; Academic Press, 2026; Vol. 94, pp 177–195.

