# Supplementary material for: Nature Counts to Three: Universal Mg-Pinch Motif Polarizes the Cleaved Bond in NTP-Processing Enzymes
Source: J Am Chem Soc. 2026 Jul 13;148(28):30494–511. doi: 10.1021/jacs.6c10146 (PMC13397572; doi:10.1021/jacs.6c10146)
Supplement: Supplementary file 1 [file ja6c10146_si_001.pdf]

Supporting Information for

Nature counts to three: Universal Mg-pinch motif polarizes the cleaved bond in NTP-processing enzymes

Balint Dudas[a,b]†, Dénes Berta[a,c,d,e]†, Pablo Jambrina[f], Pedro J. Buigues[g], Reynier Suardiaz[h], Silvia Gómez-Coca[i], Wenhao Deng[a], Bernard R. Brooks[b], Beáta G. Vértessy[j,k], Edina Rosta[a,l,]*

† equal contribution

a Department of Physics and Astronomy, University College London, London, UK

b Laboratory of Computational Biology, National Heart, Lung and Blood Institute, National Institutes of Health, Bethesda, Maryland, USA

c HUN-REN-BME Quantum Chemistry Research Group, Budapest University of Technology and Economics, Budapest, Hungary

d MTA-BME Lendület Quantum Chemistry Research Group, Budapest University of Technology and Economics, Budapest, Hungary

e Department of Physical Chemistry and Materials Science, Budapest University of Technology and Economics, Budapest, Hungary

f Department of Physical Chemistry, University of Salamanca, Salamanca, Spain

g Italian Institute of Technology, Genova, Italy

h Department of Physical Chemistry, Complutense University of Madrid, Madrid, Spain

i Department of Inorganic and Organic Chemistry and Institute of Theoretical and Computational Chemistry, University of Barcelona, Barcelona, Spain

j Department of Applied Biotechnology and Food Science, Budapest University of Technology and Economics, Budapest, Hungary

k Genome Metabolism Research Group, Research Centre for Natural Sciences, Hungarian Research Network, Budapest, Hungary

l Institute of Chemistry, Eötvös Loránd University, Budapest, Hungary

* e.rosta@ucl.ac.uk

Contents

1. Methods and data collection 3
Analysis of Enzymatic Reactions 3
PDB accession and analysis of the Mg^{2+} coordination 3
Consensus structures representing each superfamily 4
QM/MM and QM calculations....5
Alternative projections of the ion clusters 7
2. Phosphatases: Pγ leaving group 9
3. Pyrophosphatases: PβPγ leaving group 12
4. Exceptional coordination and superfamilies challenging to classify 13
Phytol kinase sequence analysis 13
5. Triphosphatases: $P_\alpha P_\beta P_\gamma$ leaving group 20
6. Non-Mg^{2+} cofactors 20
Domain length distributions 20
Metal ion coordinating residues 26
7. Analysis on the protein fold level 28
8. Analysis of the EC category distributions 31
9. The effect of Mg^{2+} analyzed through QM/MM and QM calculations 34
Model system results 34
QM/MM system results....39
10. Phosphatase superfamilies 60
Phosphatases with αβγ coordination on the (+) side 60
Phosphatases with βγ coordination from the (+) side 71
Phosphatases with αβγ coordination on the (-) side....73
Phosphatases with βγ coordination on the (-) side....77
11. Pyrophosphatase superfamilies 93
Pyrophosphatases with αβγ coordination on the (+) side 93
Pyrophosphatases with αβ coordination on the (+) side....101
Pyrophosphatases with αβγ coordination on the (-) side 108
Pyrophosphatases with αβ coordination on the (-) side....122
Additional pyrophosphatases....128
12. Other superfamilies without sufficient structural information:....134
13. Triphosphatase superfamilies....146
Appendix 150

QM Geometries for the model enzymatic transition states and reactants 150
Note 1: ECs associated to superfamily members 164
Note 2: NTP processing ECs with no associated superfamily 167
References 167

1. Methods and data collection

Analysis of Enzymatic Reactions

The KEGG database was analyzed as of 20th of December 2023. There are 6728 distinct EC numbers, of which 6197 carry out 10571 KEGG registered chemical reactions. The reactions were primarily obtained from the KEGG reaction database, substrates and products of the enzymatic reactions were parsed for ECs where no KEGG reaction entry was assigned. Each reaction corresponds to one or more EC numbers. The details of these can be found in https://github.com/Rosta-Research-Group/Nature-counts-to-three, KEGG_info document. The reactions were categorized as follows:

- Whether any substrate contained a phosphate group. 2012 phosphate compounds (KEGG_info A1) were identified from the KEGG compound database.
- If the enzymatic activity involved phosphate chemistry. This was determined using KEGG reaction classes (KEGG_info A2). Because reaction-class assignments are not exhaustive, the actual number of phosphate-processing reactions is expected to be higher.
- If one of the substrates was a nucleoside triphosphate (or modified NTP), it was subcategorized into one of the following (KEGG_info A3):
  - Phosphatase, if it produced inorganic phosphate and/or NDP
  - Pyrophosphatase, if it produced inorganic pyrophosphate or NMP
  - Triphosphatase, if it produced a nucleoside or inorganic triphosphate.

Thirty-six reactions could not be categorized by these criteria and were therefore classified manually (KEGG_info A4).

The identified NTP-processing ECs were then used to retrieve PDB structures and protein sequences. Protein chains were matched to the SUPERFAMILY 2.0[1-2] database, which contains hidden Markov model profiles representing SCOPe SF-level structural domains, to assign each sequence to a SCOPe SF.[3-4] For cases where SUPERFAMILY 2.0 did not yield a match, the corresponding InterPro[5] SF (or, if absent, the InterPro Family) was used. Redundant SFs—InterPro entries structurally similar to existing SCOPe SFs based on DALI alignments—were merged.

PDB accession and analysis of the Mg^{2+} coordination

We performed the Mg^{2+} coordination analysis on experimental PDB structures[6] that meet the following four criteria: i) a resolution of at least 2.7 Å to ensure reliable metal-ion coordination; ii) the presence of a residue containing three phosphorus atoms; iii) at least one relevant divalent metal ion (Mg^{2+}, Ca^{2+}, or Mn^{2+}—excluding Zn^{2+} due to its distinct biochemical role) within 5 Å of the triphosphate moiety; and iv) a coordination number of at least four around the metal ion, defined as atoms within 3 Å of the cation, to ensure adequate structural quality of the active site.

From the PDB, we retrieved 8641 structures satisfying criteria (i) and (ii). Of these, 4553 contained at least one divalent metal ion (criterion iii). After excluding structures that failed any of the four criteria or showed

ambiguity in the active site (occupancy < 1.00 for either the metal ion or its coordinating atoms), a total of 3118 PDB entries fulfilled all structural and geometric requirements and were retained for further analysis. Using the SUPERFAMILY 2.0 webserver,[1-2] we classified the active sites — defined as the protein region binding the three-phosphorus–containing residue together with the metal ion(s) — into superfamilies (SFs) based on amino acid sequence. Residues were filtered to include only ligands containing at least three phosphate groups in a chain, based on geometric criteria for consecutive bonding, and each active site was assigned to the protein chain contributing the most atoms around the NTP (or analog) ligand.

We then carried out a systematic assessment of all active sites to determine: i) the number of coordinating metal ions (one, two, or more), and ii) the coordination configuration of each metal ion, specifically whether it interacts with the nonbridging oxygens of the α, the β, and/or the γ phosphate groups.

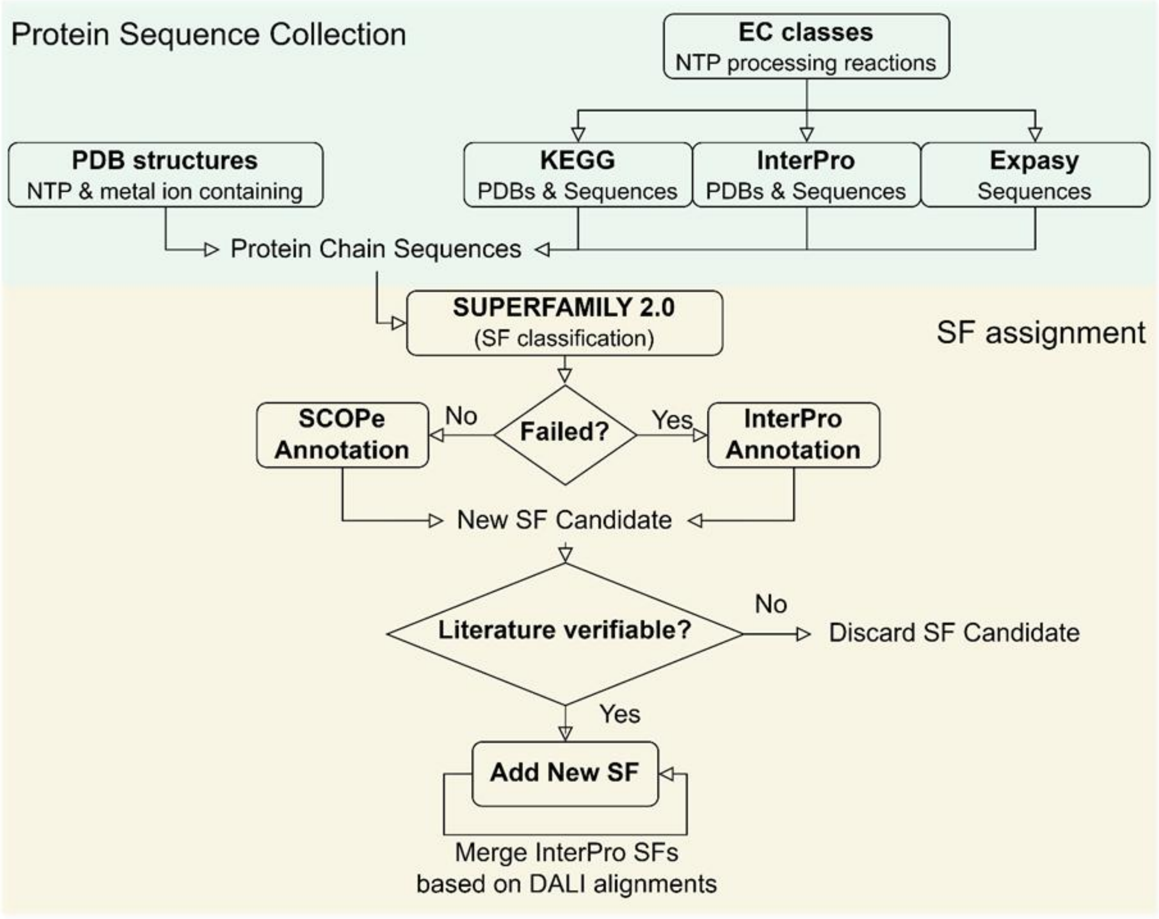

Figure S1: *Flowchart of the data-collection and classification workflow used to identify all NTP-processing superfamilies (SFs) with currently available structural data.*

Consensus structures representing each superfamily

A representative structure was selected for each SF to reflect the consensus metal-ion coordination observed after aligning all available NTP-bound active sites within that superfamily. Although this selection is not fully automated, our goal was to illustrate the characteristic coordination pattern of each SF. We

therefore manually chose structures in which all relevant metal ions are present (when more than one is used) and exhibit full octahedral coordination, including crystallographic waters when available. Based on the identity of the leaving group in the corresponding catalytic reaction, the SFs were classified as phosphatases (P_γ monophosphate leaving group), pyrophosphatases ($P_\beta P_\gamma$ pyrophosphate leaving group), or triphosphatases ($P_\alpha P_\beta P_\gamma$ triphosphate leaving group).
Finally, we examined and compared the positions of metal ions relative to the triphosphate chain across all representative structures from both phosphatase and pyrophosphatase SFs. This systematic analysis allowed us to comprehensively describe all currently identified catalytically relevant Mg-coordination modes in NTP-processing enzymes.

QM/MM and QM calculations

RNase H simulation setup. Minimized QM/MM structures for HIV-1 RNase H were taken from the model of Dürr *et al.*,[7] built using the 3.2 Å HIV-1 RT/DNA–RNA complex of Huang *et al.* (PDB **1RTD**) and refined using the higher-resolution D192N *Bacillus halodurans* RNase H catalytic domain (PDB **1ZBL**). The structure was equilibrated followed by 20 ns classical MD simulations in explicit solvent. For QM/MM the system was trimmed to a 20 Å sphere around the scissile phosphate, atoms beyond 15 Å were frozen, and CHARMM27 was electrostatically embedded into Q-Chem 4.4 (B3LYP/6-31+G(d)). The QM region (109 atoms) comprised both catalytic Mg^{2+}, the RNA backbone of the reacting nucleotide and the 3′-neighbor, the side chains of Q475, D443, E478, D498, D549 and H539, and 6 active-site waters; MM contained 4164 atoms. The geometry optimization was done using a reaction coordinate that included the proton transfer (Q_p) and electron transfer (Q_e) defined as:

$$Q_{ep} = Q_e + Q_p$$

where

$$Q_e = d(\text{WAT\_nuc:O H}_2\text{, RNA:P}) - d(\text{RNA:P, RNA:O3'})$$

and

$$Q_p = d(\text{H1126:NE2, WAT\_nuc:H}) - d(\text{WAT\_nuc:H, WAT\_nuc:O}) + d(\text{RNA:O3', RNA:H2'}) - d(\text{RNA:H2', E1065:OE1})$$

dUTPase simulation setup. The QM/MM optimized reaction pathway was obtained in Ref [8], [9] and [10]. The models of *M. tuberculosis* dUTPase were originally built in Ref [8] from the 1.49 Å resolution PDB structure 2PY4 (WT enzyme with α,β-imido-dUTP and Mg^{2+}),[11] converting the imido group to an O to obtain dUTP. After initial MD equilibration, the protein–substrate complex was truncated to a 21 Å sphere around Pα of dUTP; atoms beyond 15 Å were frozen. QM/MM calculations were done with CHARMM27 electrostatically embedded in Q-Chem/Gaussian at the B3LYP/6-31+G(d,p) level, link hydrogens at the cut bonds, no cutoff for QM/MM electrostatics. The QM region (132 atoms) contained dUTP, the backbone of T81 and I82, the side chains of D83, S65, H145, S147, S148, the Q113 amide, the R140 guanidinium, the Mg^{2+} ion with its full coordination shell and nearby crystallographic water molecules, in total 10 water molecules. The reaction coordinate for the QM/MM minimization scans was defined as

$$Q = Q_{ET} + 0.5\, Q_{PT}$$

with

$$Q_{ET} = d(\mathrm{P}_\alpha - \mathrm{O}_{3A}) - d(\mathrm{P}_\alpha - \mathrm{O_w}),$$

representing the bond breaking/formation in the phosphate cleavage, and

$$Q_{PT} = d_3 - d_4 = d(\mathrm{O_w} - \mathrm{H_w}) - d(\mathrm{O}_{D83} - \mathrm{H_w}),$$

representing the proton transfer from the nucleophilic water to the catalytic Asp83.

Ras simulation setup. The QM/MM optimized reaction pathways were obtained in Ref. [10]. For Ras·p120GAP complex, starting coordinates were taken from the Ras·p120GAP transition-state-analogue complex 1WQ1 and rebuilt to a reactive Ras·GTP·Mg^{2+} complex. Classical MD was performed using CHARMM36 in NAMD: 10 000 steps minimization, 10 ns restrained equilibration, followed by three independent 200 ns production trajectories. QM/MM snapshots were extracted from equilibrated frames containing a properly oriented catalytic water molecule. The system was truncated to a 25 Å sphere around the Mg^{2+} ion of the catalytic pocket, and the positions of atoms farther than 20 Å were kept fixed. The QM region comprised the GTP triphosphate (α–β–γ) including the bridging O, Mg^{2+} ion and its two first-shell waters, the nucleophilic water, the full side chain of Ras Gln61, the guanidinium group of the GAP arginine finger (Arg789), the side chain of Ras Lys16, the side chains of Ser17 and Thr35 that directly coordinate Mg^{2+}, and two water molecules that H-bond between Gln61 and the phosphate, accounting for 101 atoms in total. QM/MM calculations were performed in CHARMM electrostatically embedded into a DFT code (B3LYP/6-31+G(d)). The reaction coordinate ξ was defined as:

$$\xi = d(\mathrm{P}_\gamma - \mathrm{O}_{3\beta}) - d(\mathrm{P}_\gamma - \mathrm{Wat_{nuc}{:}O}) + 0.5\, d(\mathrm{Wat_{nuc}{:}O} - \mathrm{Wat_{nuc}{:}H}) - 0.5\, d(\mathrm{Wat_{nuc}{:}H} - \mathrm{O}_{3\gamma})$$

— coupling the phosphate-cleavage (first two terms) and proton-transfer (last two terms) processes.

Table S1. *Details of the QM/MM model building and simulation parameters.*

Protein	Human HRas GTPase	M. Tb. dUTPase	HIV-1 Ribonuclease H
Starting PDB code	1WQ1	2PY4	1RTD/1ZBL
Reaction	NTP -> NDP + Pi	NTP -> NMP + PPi	RNA chain breakup
Number of Mg^{2+} ions	1	1	2
Number of QM atoms	101	132	109
Formal charge of the QM region	0	-2	-1
MM force field	CHARMM36m	CHARMM27	CHARMM27

The QM region with electrostatic embedding were originally optimized with the B3LYP hybrid functional at the 6-31+G(d) split-valence basis set. The energy barrier evaluations with and without the Mg^{2+} ion were done at the ωB97M-V range-separated hybrid functional with VV10 dispersion correction and the

def2-TZVP basis set. QM/MM calculations were done with the interface between QChem 5.2[12] and CHARMM 47b1.[13]

QM calculations

Minimal QM models were derived from the QM/MM structures. The NTP substrate was truncated at the C5′ atom to a methyl-triphosphate (MeTP), retaining the Mg^{2+} ion, the nucleophilic water, and the catalytic base. For consistency, the catalytic base was an acetate for the calculations in Fig. 6C and 6D. In the pyrophosphatase model, the carboxylate base was modeled as an acetate, in the Ras-based phosphatase model, an acetamide. The xyz coordinates of the reactant and transition-state structures of these models are provided in the Appendix.

Electronic properties and population analysis were performed at the B3LYP/6-31+G(d) level of theory with the software packages: QChem 5.2, Gaussian 09 Rev. E and NBO 3.0.[12, 14-15]

For the point-charge screening calculations, the reactant and transition-state structures were aligned, and a Cartesian grid with 0.35 Å spacing was generated to sample points located 2.0–2.1 Å from the phosphate oxygens.
Electron densities from the DFT SCF calculations were processed with Gaussian 09 and analyzed on a 200^3 grid. Density-difference isosurfaces are shown at ±0.001 au.

Full QM/MM geometries and details of the density and point charge analyses are available at https://github.com/Rosta-Research-Group/Nature-counts-to-three.

Alternative projections of the ion clusters

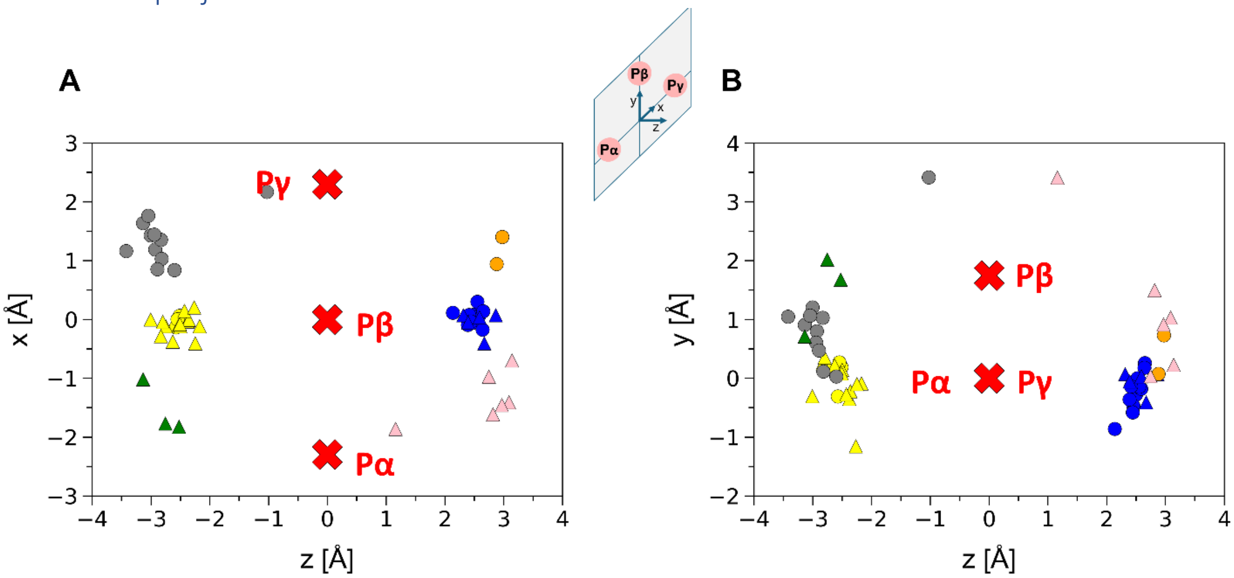

Figure S2: *Spatial distribution of Mg-pinch–forming metal ions relative to the phosphate groups, shown from two orthogonal views (**A** and **B**). The red labels indicate the positions of the Pα, Pβ, and Pγ atoms in the reference coordinate frame. Ion positions from phosphatase SFs are shown as circles, whereas those*

from pyrophosphatase SFs are shown as triangles. Colors correspond to the metal ion coordination modes introduced in Figure 2.

2. Phosphatases: Pγ leaving group

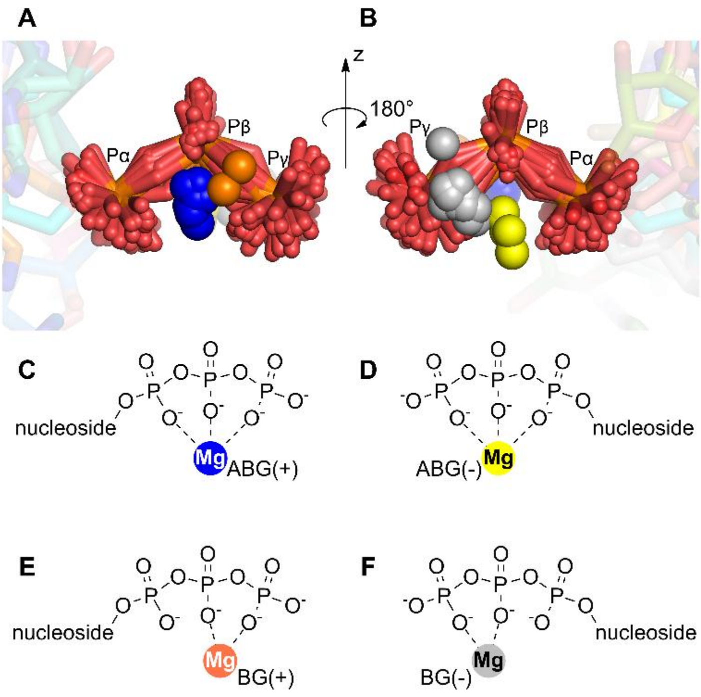

Figure S3. *Clusters of catalytic divalent metal ions observed in phosphatases. The Mg-pinch–forming ions from the representative phosphatase structures are shown from two opposite views relative to the plane defined by the three phosphorus atoms (Pα, Pβ, Pγ).*

A) *Ions located on the "A" side of this plane (left side in the front view), with αβγ-coordinated ions shown in blue and βγ-coordinated ions shown in orange.*

B) *Ions located on the "B" side of the plane (right side in the front view), with αβγ-coordinated ions shown in yellow and βγ-coordinated ions shown in gray.*

In both panels, the NTP ligands from the representative structures were superimposed based on their three phosphorus atoms and are shown as sticks.

Besides the pinching Mg^{2+} ion, we also analyzed the positions of any additional metal ions present at the active site that coordinate the triphosphate group. We identified a second, well-defined cluster formed by an additional metal ion in five SFs (Figure S4). These second metal ions occupy the AG site and are positioned close to the plane defined by the three phosphorus atoms. Interestingly, this AG-site cluster is observed only in SFs where the pinching ion adopts the BG coordination mode. Additional metal ions were also found in other superfamilies at the pinching position (BG, 2 SFs), at the AB position (2 SFs), or interacting solely with the leaving phosphate group (G, 2 SFs; see Table 1).

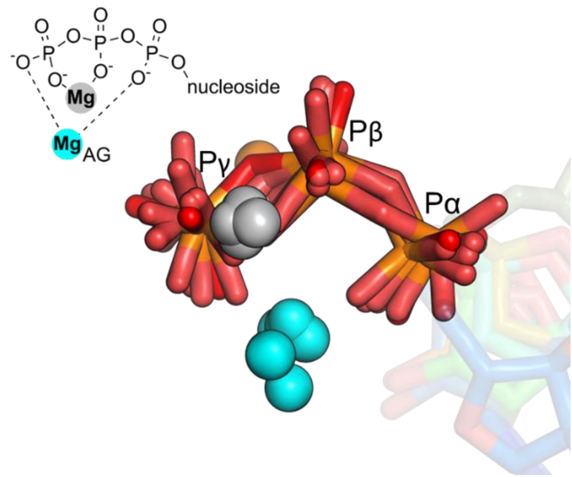

Figure S4. *Cluster of divalent second metal ions (cyan) identified in five phosphatase SFs, in addition to their Mg-pinch–forming ion (shown in gray or orange). These second ions occupy the αγ-coordination site (AG site) and lie closer to the plane defined by the three phosphorus atoms than the Mg-pinch–forming ions. The following SFs belong to this group: Protein kinase–like, Glutathione synthetase ATP-binding domain–like, SAICAR synthase–like, and Diacylglycerol kinase (DgkA)–like, all of which have their Mg-pinch–forming ions at the BG(–) site (gray), and Glutamine synthetase/guanido kinase, which symmetrically positions its Mg-pinch–forming ion at the BG(+) site (orange).*

We identified metal ions with βγ and αβγ coordination on both sides of the plane defined by the three phosphorus atoms in phosphatases. Notably, most metal ions located on the (+) side are coordinated simultaneously by all three phosphate groups (ABG(+), Figure 2 and Table 1). Among the phosphatases with the Mg-pinch–forming ion at the ABG(+) site, eight SFs contain a single metal ion, whereas three contain additional metal ions at their active sites. Of the phosphatases with ions positioned on the (+) side, only two SFs belong to the BG(+) group (shown in orange in Figure 2).

Conversely, the *Glutamine synthetase/guanido kinase* SF, for which only a few high-resolution structures are available, features a multi-ion active site. Notably, this SF is unique in having a second metal ion at the AG site (shown in cyan in Figure S4) while simultaneously positioning its Mg-pinch–forming ion on the (+) side, as well as a third ion located near the nucleophile.

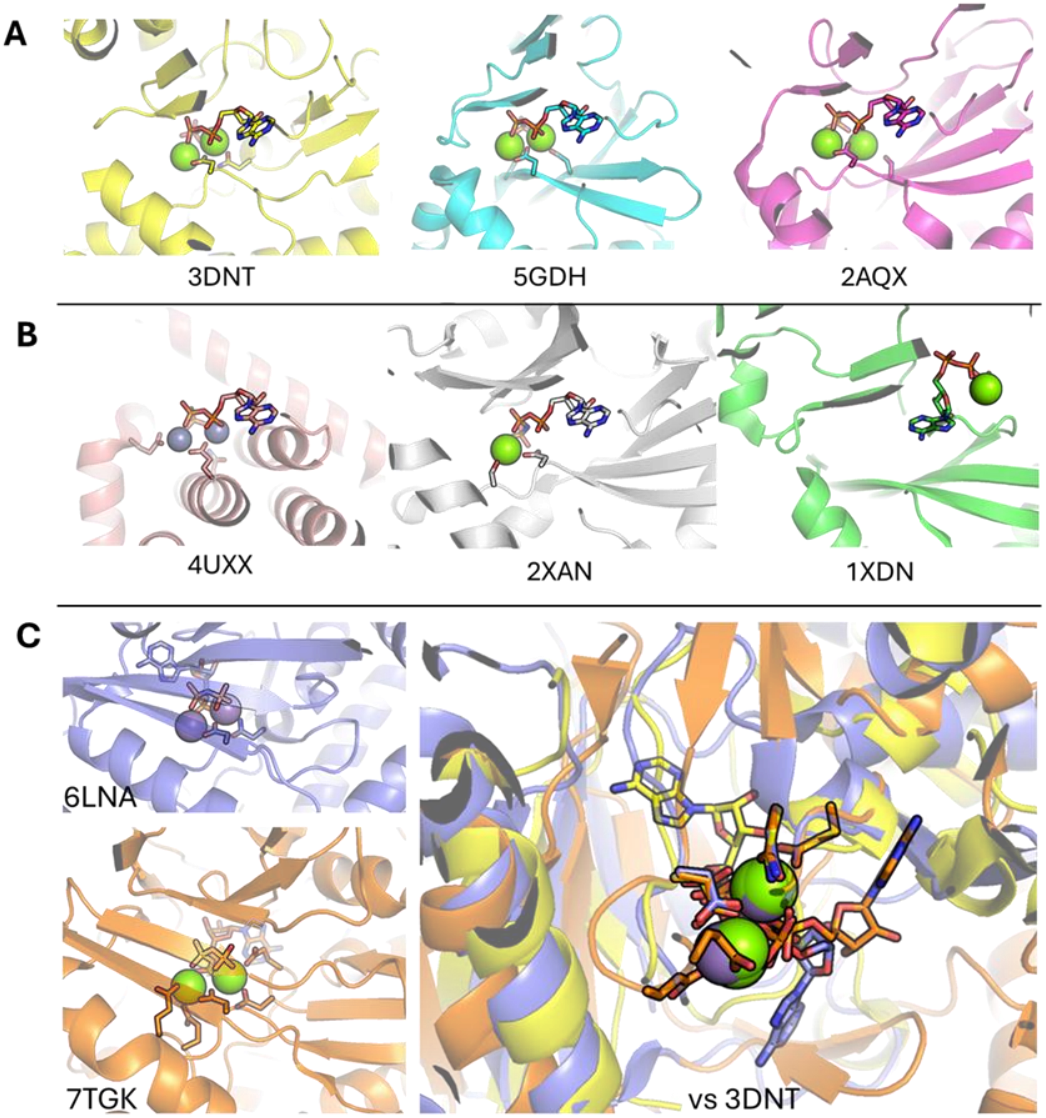

Figure S5. A: *Aligned individual structures showing the same active site orientation for the ATP grasp motif: Protein kinase–like (3DNT, yellow), Glutathione synthetase ATP-binding domain–like (5GDH, cyan), and SAICAR synthase–like (2AQX, pink) SF representatives in the top row.* ***B:*** *Aligned active sites for the representatives of Diacylglycerol kinase (DgkA)–like (4UXX), Inositol-pentakisphosphate 2-kinase (2XAN), and DNA ligase/mRNA capping enzyme, catalytic domain (1XDN) SFs. While the 4UXX structure is a clear outlier in terms of its fold, 1XDN differs significantly in its nucleotide binding.* ***C:*** *Left: Active site representatives of Protein adenylyltransferase SelO family (6LNA, blue), and Aerobactin siderophore biosynthesis, IucA/IucC-like SF (7TGK, orange). Alignment with Protein kinase–like active site (3DNT, yellow) is also shown (right).*

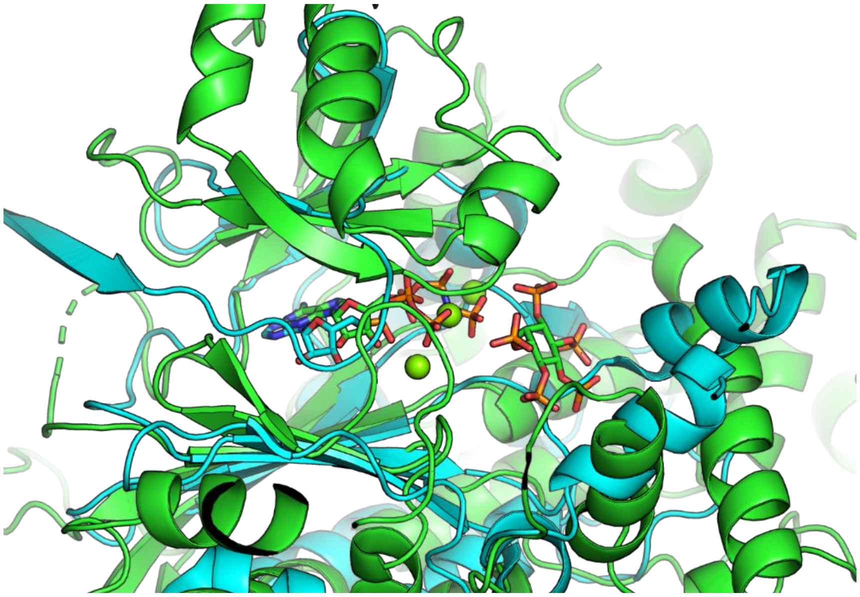

Figure S6: *Aligned structures of the Inositol-pentakisphosphate 2-kinase SF (green, 2XAN) and the SAICAR synthase-like SF (cyan, 2AQX).*

3. Pyrophosphatases: PβPγ leaving group

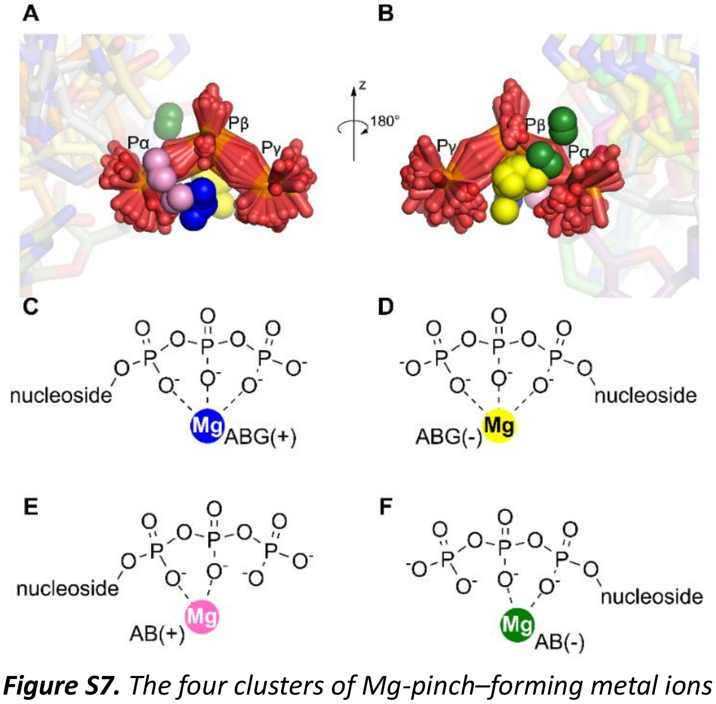

Figure S7. *The four clusters of Mg-pinch–forming metal ions observed in representative pyrophosphatase structures.*

A) *Ions located on the (+) side of the plane defined by the three phosphorus atoms, with αβγ-coordinated ions shown in blue (ABG(+)) and αβ-coordinated ions shown in pink (AB(+)).*

B) *Ions located on the (–) side of the same plane, with αβγ-coordinated ions shown in yellow (ABG(–)) and αβ-coordinated ions shown in green (AB(–)).*

In both panels, the NTP ligands from representative superfamily structures are superimposed using their three phosphorus atoms and are shown as sticks.

4. Exceptional coordination and superfamilies challenging to classify

Phytol kinase sequence analysis

Sequence collection and structural dataset assembly

UniProt was queried to retrieve all protein sequences annotated with EC 2.7.1.174, 2.7.1.182 and 2.7.1.216. For structural analysis, UniProt AlphaFold-predicted structures (v6 only) were obtained for each group where available. The resulting structure collections comprised 47 models for EC 2.7.1.174, 5 models for EC 2.7.1.216, and 1,570 models for EC 2.7.1.182.

All AlphaFold structures were compared using DALI against a reference panel of 85 structural templates spanning all identified SF PDB structures, adding also additional chains, a phosphatidate cytidylyltransferase PDB: 4Q2GA (note, different from *Phosphatidate cytidylyltransferase, mitochondrial* SF), and three internal references: A0A1F8NHP0 (A1), A0A8H3B4Z4 (B1) and A0A0H2ST96 (B2) AlphaFold models (see also Figure S8, left). This ensured that the reference panel could capture divergent conformations/folds present within the AlphaFold dataset rather than forcing a single-fold interpretation.

DALI alignment results

DALI (DaliLite v.5)[16] comparisons for the 5 sequences in the 2.7.1.216 EC set resulted in a mean Z=33.6 (median 32.4, SD 1.0), median RMSD=1.6 Å (n=5) for the internal template A1. There were also close matches for all 5 sequences with the experimental templates 5GUF: mean Z=9.2 (median 9.3, SD 0.2), mean RMSD=2.6 Å , and 4Q2G: mean Z=9.1 (median 8.9, SD 0.5), mean RMSD=12.4 Å.

DALI comparisons for the 47 structures in the 2.7.1.174 EC set to the reference templates similarly show strong structural agreement with the main fold:

A1: mean Z=15.3 (median 15.9, SD 1.9), mean RMSD=3.1 Å (n=45)

5GUF: mean Z=6.0 (median 6.1, SD 1.2), mean RMSD=3.1 Å (n=46)

4Q2G: mean Z=7.9 (median 8.3, SD 1.8), mean RMSD=5.0 Å (n=46).

Only 3 structures have a Z score for the internal template A1 below 6.0 (A0AAF0J0M5 has a slightly lower Z=4.8 score to A1 with RMSD of 4.7 Å, A0AAN7DM55 is annotated as a diacylglycerol kinase, it matches 7TGK, an ATP grasp family enzyme, with Z score of 6.2 and RMSD of 4.0 Å, and A0AAF0E4K9 has a poor structural prediction).

This is consistent with the observation that most models align well to the dominant A1-like group from ECs 2.7.1.174 and 2.7.1.216, matching the *Intramembrane CDP-alcohol synthase* pyrophosphatase SF.

Because EC 2.7.1.182 was more diverse with 1570 sequences, we performed structural analysis and subsequent clustering was performed on this largest group. For each of R = 85 experimentally determined structures and representative models, we retained the full profile of DALI outputs (Z-scores and RMSD values) across the entire panel. Missing query–reference alignments were encoded as non-matches (Z = 0; RMSD = 100 Å) to obtain a complete, fixed-length feature representation per query, q:

$$\mathbf{x}_q = (Z_{q,1}, \dots, Z_{q,R}, \mathrm{RMSD}_{q,1}, \dots, \mathrm{RMSD}_{q,R}).$$

We then standardized each column (each “Z to reference q” and each “RMSD to reference q”) across all queries:

$$x'_{q,j} = \frac{x_{q,j} - \mu_j}{\sigma_j},$$

where μ_j and σ_j are the mean and standard deviation of feature j across all queries. These feature vectors were then used for unsupervised k-means clustering, and cluster robustness was evaluated across multiple

random initializations to confirm stability of the dominant solutions. We then performed principal component analysis (PCA) on the standardized feature matrix and projected queries into the first two principal components for visualization only (Figure S8, right). Of the 1,570 models, 1,567 were assigned to clusters; 3 models had no DALI hits and were left unassigned.

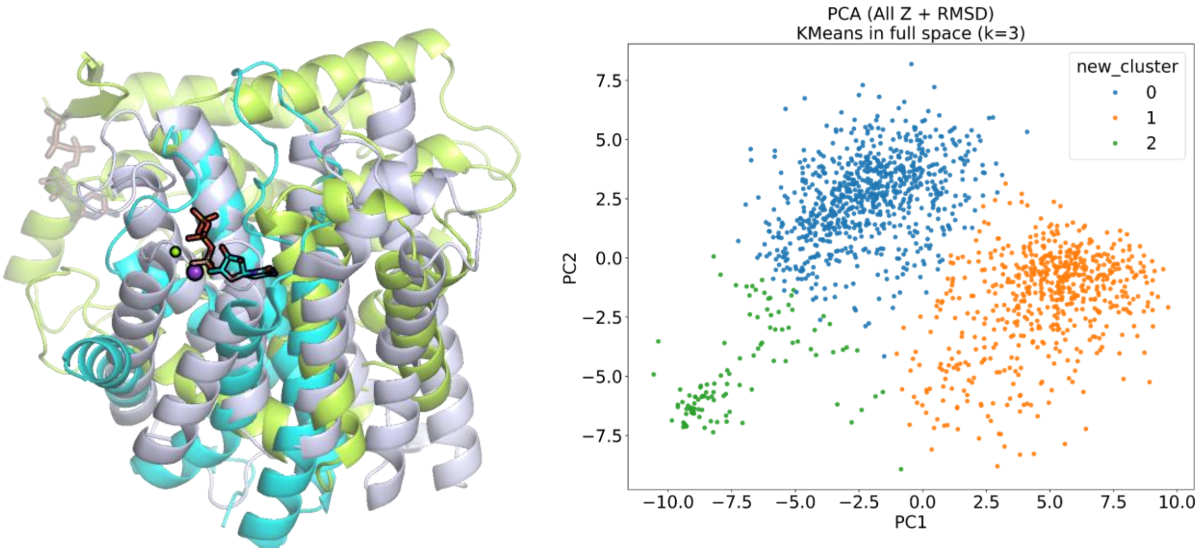

Figure S8. (Left) *Structural alignment for predicted structures of A1 (grey cartoon), B1 (olive cartoon and CTP using sticks), and the representative of the Intramembrane CDP-alcohol synthase* SF: *5GUF (cyan cartoon and substrate with sticks).* ***(Right)*** *PCA projection of the full DALI feature matrix (Z-scores and RMSDs to 85 references) for EC 2.7.1.182 AlphaFold models, with points colored by k-means assignment in the feature space for k=3 clusters. The 2D projection is shown for visualization using the leading PCA eigenvectors (PC1 and PC2).*

This analysis resolved two dominant structural regimes and a smaller heterogeneous subset (Table S2). One cluster showed consistently strong structural agreement with the A1-like, *Intramembrane CDP-alcohol synthase* pyrophosphatase SF references, with high DALI similarity to A1-like representatives (median Z to A1 ≈ 32) and comparatively low dispersion, consistent with membership in the established A1-like fold class. In contrast, the remaining major clusters were characterized by low similarity to A1-like references (median Z to A1 ≈ 2–3) while retaining moderate similarity to B1-like templates (median Z to B1 ≈ 6), suggesting a structurally distinct regime relative to the A1-like fold despite local similarities to other kinase-like templates (Figure S8, left). In fact, the ligand binding region of 5GUF is not present in the common aligned regions of A1 and B1, potentially suggesting that an auxiliary enzyme is required for catalysis (Figure S8, left). A minority subset exhibited mixed behavior and broader dispersion, including partial/low-quality matches that are consistent with domain boundary effects and/or structural heterogeneity rather than a clean third fold class.

Table S2. Cluster summary for EC 2.7.1.182 AlphaFold models (n=1,567) clustered on the full DALI Z+RMSD matrix (85 references). For each cluster, the cluster size, the reference with the maximum DALI Z-score (top-1 ref), median best-hit Z-score and RMSD across the full reference panel, median Z-scores to the two key anchors (A1 and B1) and the percentage of models exceeding Z ≥ 10 to each anchor. AlphaFold confidence is summarized as the median model-level mLDDT, and the median fraction of residues above pLDDT thresholds of 70 and 90 are given. Phylogenetic coherence metrics were computed on CD-HIT representatives and FastTree, the fraction of labeled tips whose nearest labeled neighbor in patristic distance belongs to the same structural cluster is reported.

**Metric**	**Cluster 0**	**Cluster 1**	**Cluster 2**
n (cluster size)	788	614	165
top-1 ref	B1-like	A1-like	B1-like (weak)
Best Z (median)	6.8	31.9	5.9
RMSD (median, Å)	4.4	1.6	5.3
Z to A1 (median)	2.5	31.9	2.9
% Z(A1) ≥ 10	2.5	96.1	3.6
Z to B1 (median)	6.5	3.1	6.0
% Z(B1) ≥ 10	1.5	0.0	0.0
mLDDT (median)	68.5	76.5	67.5
% res ≥ 70 (median)	55.9	68.9	50.5
% res ≥ 90 (median)	13.0	44.1	17.6
nearest-neighbor same-cluster (%)	87.9	90.9	39.8

AlphaFold confidence analysis

To assess whether structural outcomes were driven by AlphaFold confidence, we extracted per-residue pLDDT values from AlphaFold PDB files (pLDDT stored in the B-factor column) and computed per-model summary metrics (Table S2, Figures S9-11), including mean pLDDT (mLDDT) and the fraction of residues above confidence thresholds (pLDDT ≥ 70 and ≥ 90). Across clusters, confidence was not uniformly low (Figure S9): the A1-like cluster exhibited the highest confidence overall (median mLDDT ≈ 76.5; median fraction pLDDT ≥ 90 ≈ 0.44), whereas the non-A1-like clusters showed moderate confidence (median mLDDT ≈ 68–69; median fraction pLDDT ≥ 90 ≈ 0.12–0.18). Importantly, when DALI similarity and confidence were analyzed jointly, many low-Z outcomes persisted at moderate-to-high pLDDT, indicating that lack of A1-like similarity is not explained solely by low-confidence AlphaFold predictions; rather, it is consistent with genuine structural divergence and/or domain organization differences relative to the reference panel. These relationships are summarized by cluster-wise Z-score distributions and scatter plots comparing best DALI Z against mLDDT.

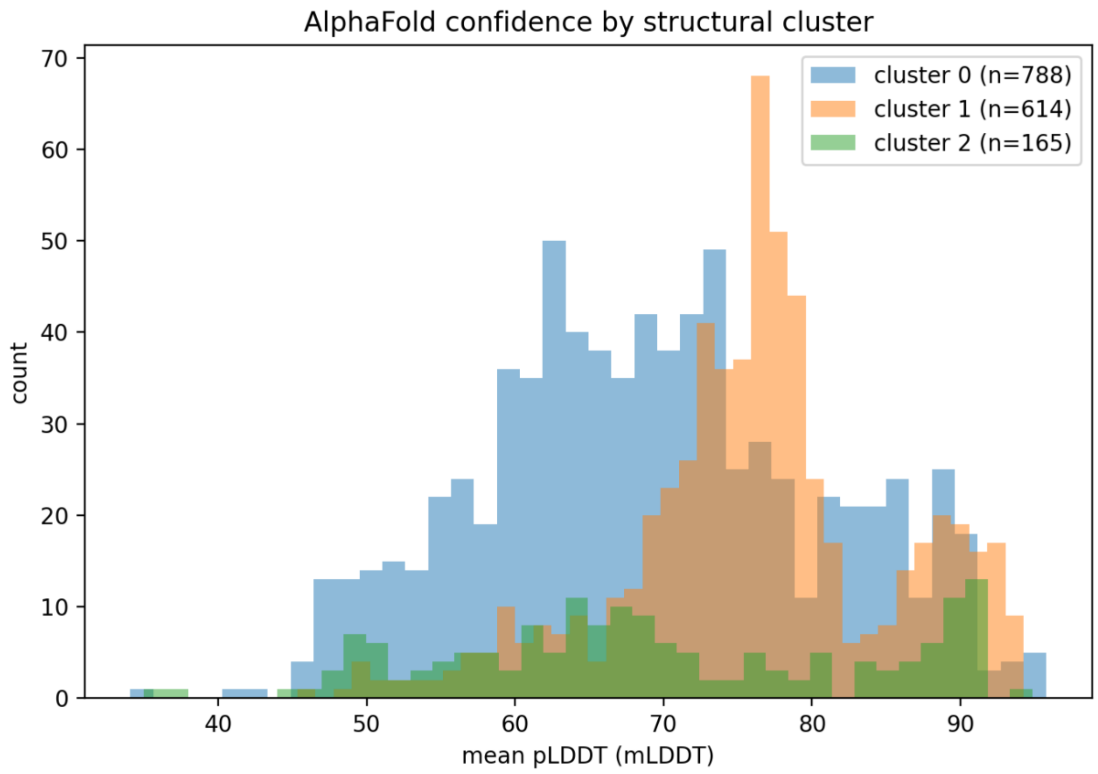

Figure S9. *Distribution of mLDDT across AlphaFold models, stratified by structural cluster.*

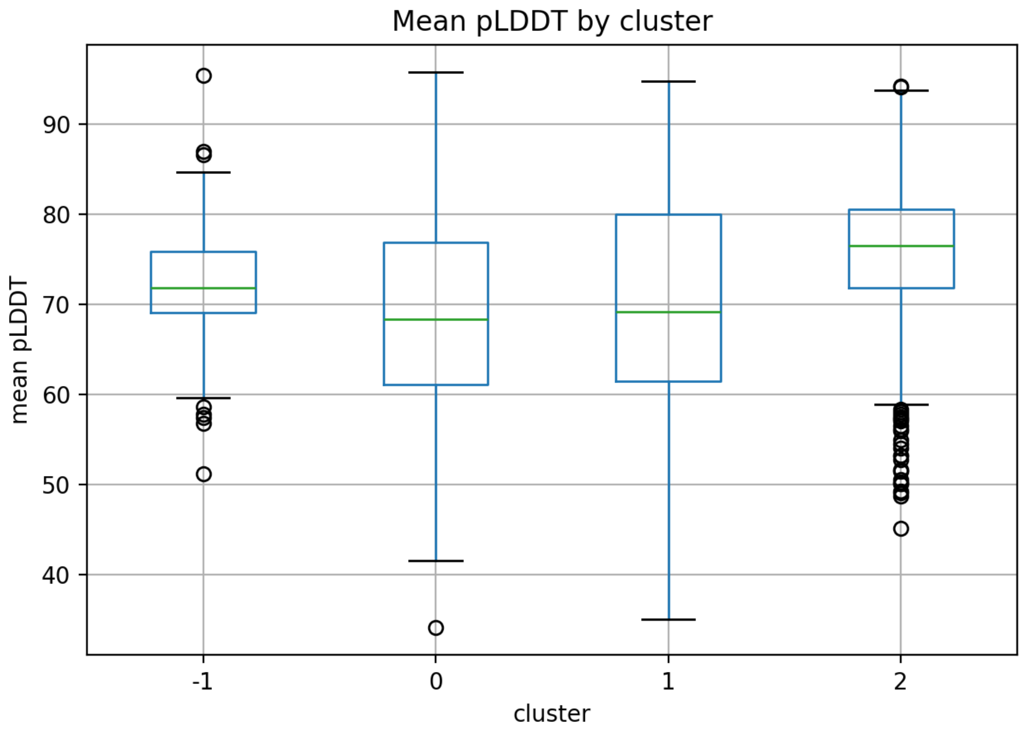

Figure S10. *Model-level mean pLDDT (mLDDT) summarized by structural clustering. Cluster −1 denotes 3 unassigned models without any DALI hits.*

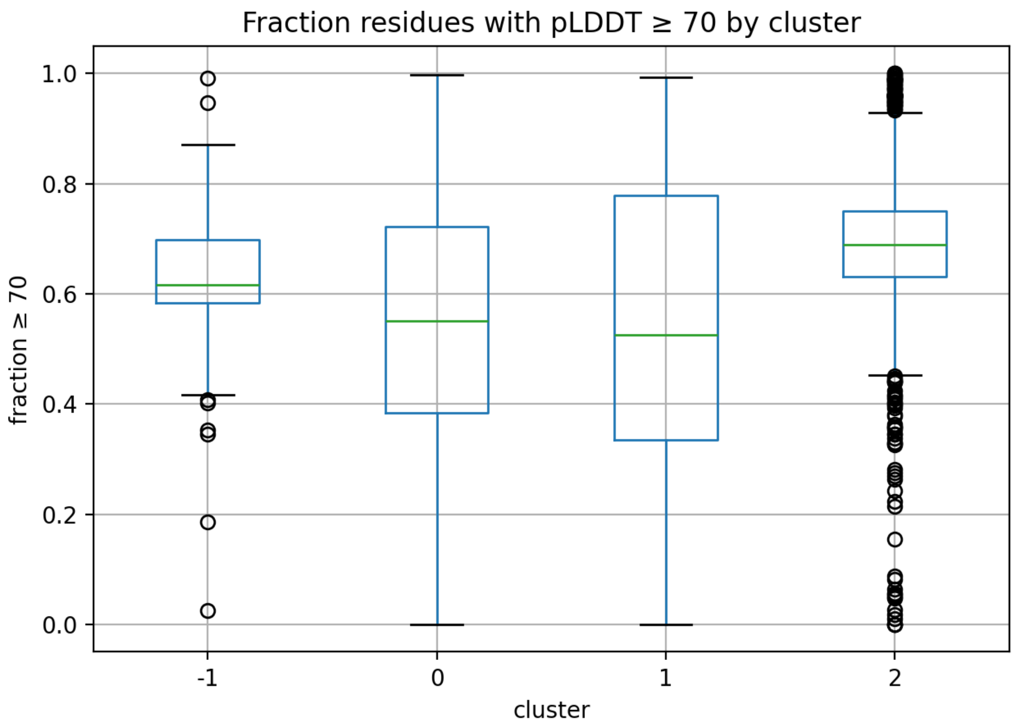

Figure S11. *Fraction of residues with pLDDT ≥70 per model, summarized by structural clustering. Cluster −1 denotes 3 unassigned models without any DALI hits.*

Phylogenetic tree analysis

Finally, we tested whether the structural clusters track evolutionary separation using a representative phylogenetic analysis. Sequences were first redundancy-reduced at 70% identity using CD-HIT,[17] representatives were aligned using MAFFT,[18] and an approximately maximum-likelihood–style tree was inferred with FastTree.[19] Tips were colored by the structural cluster assignment to visualize concordance (Figure S12). Although clusters were not strictly monophyletic at the whole-tree level (consistent with broad taxonomic sampling and possible domain architecture variation), structural labels exhibited strong local phylogenetic coherence: the nearest labeled neighbor shared the same structural cluster for ~83% of labeled tips, and within-cluster patristic distances were substantially smaller than between-cluster distances (median within ≈ 4.6 vs median between ≈ 6.8, Figure S13). Together, these results support that the dominant structural regimes reflect coherent evolutionary groupings rather than stochastic variation in model quality.

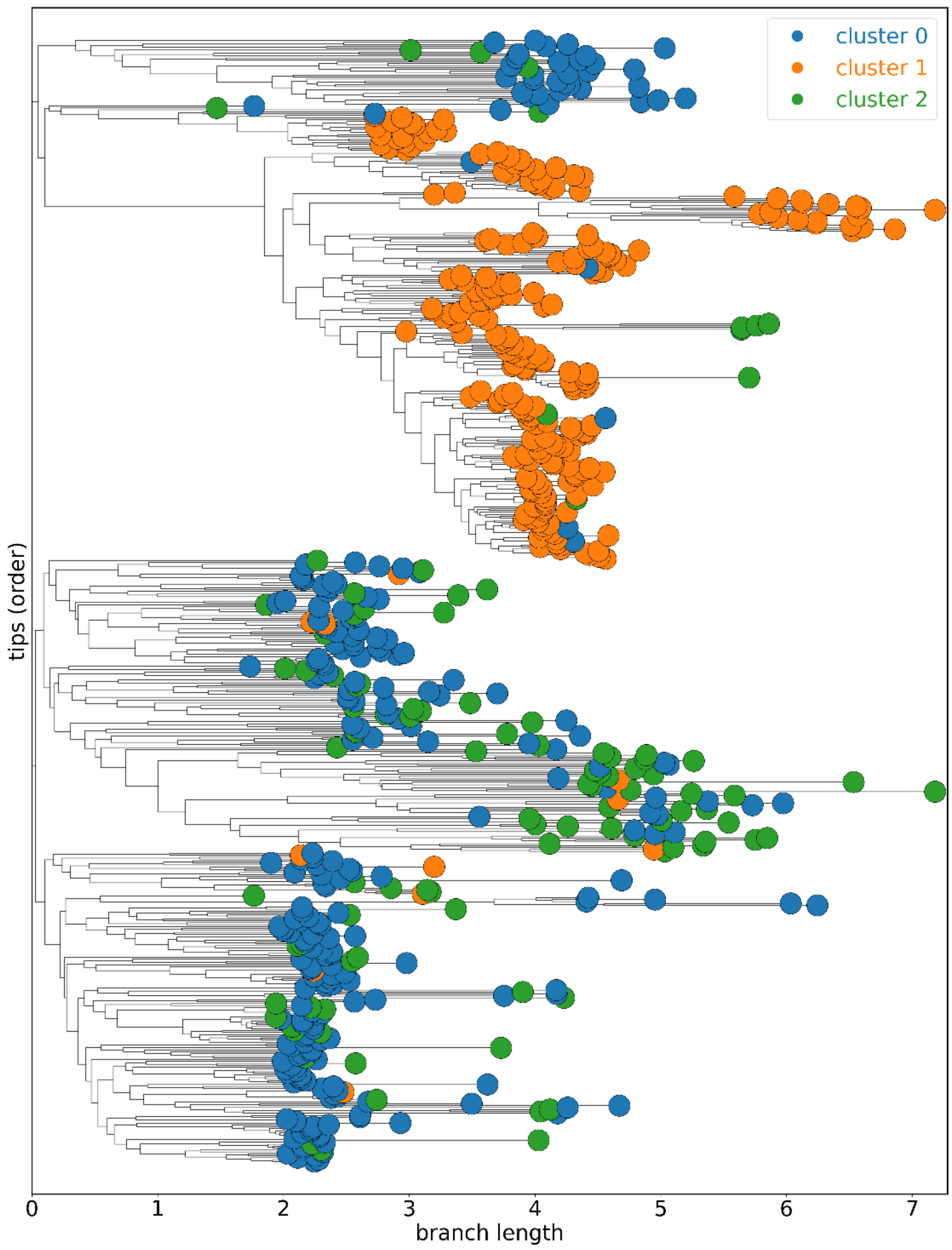
cluster 0
cluster 1
cluster 2
tips (order)
0
1
2
3
4
5
6
7
branch length

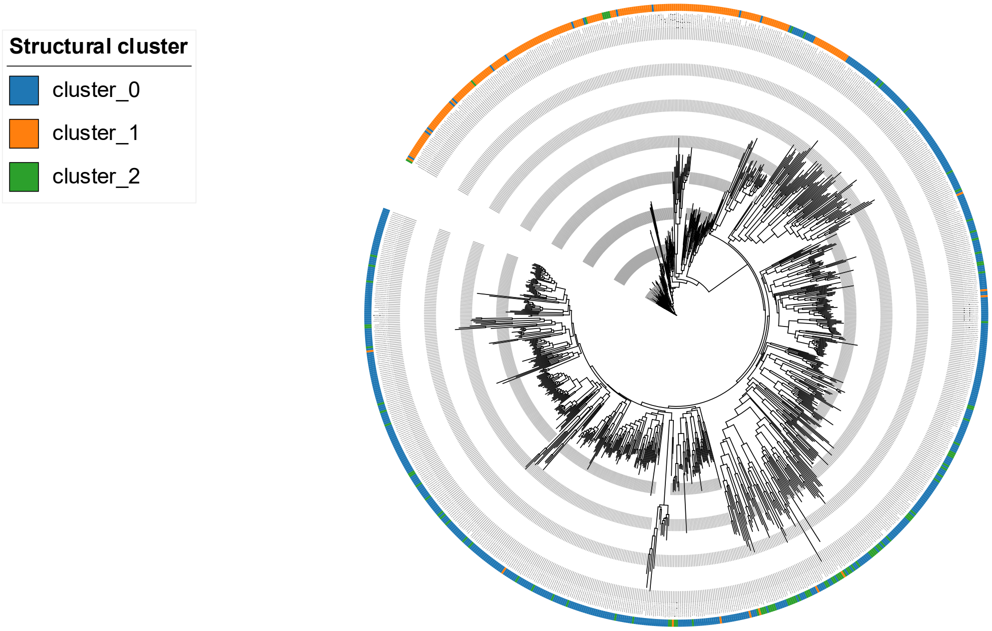

Figure S12. *FastTree phylograms of representative sequences (pruned for readability), with tips colored by structural cluster. The tree is midpoint-rooted for visualization.*

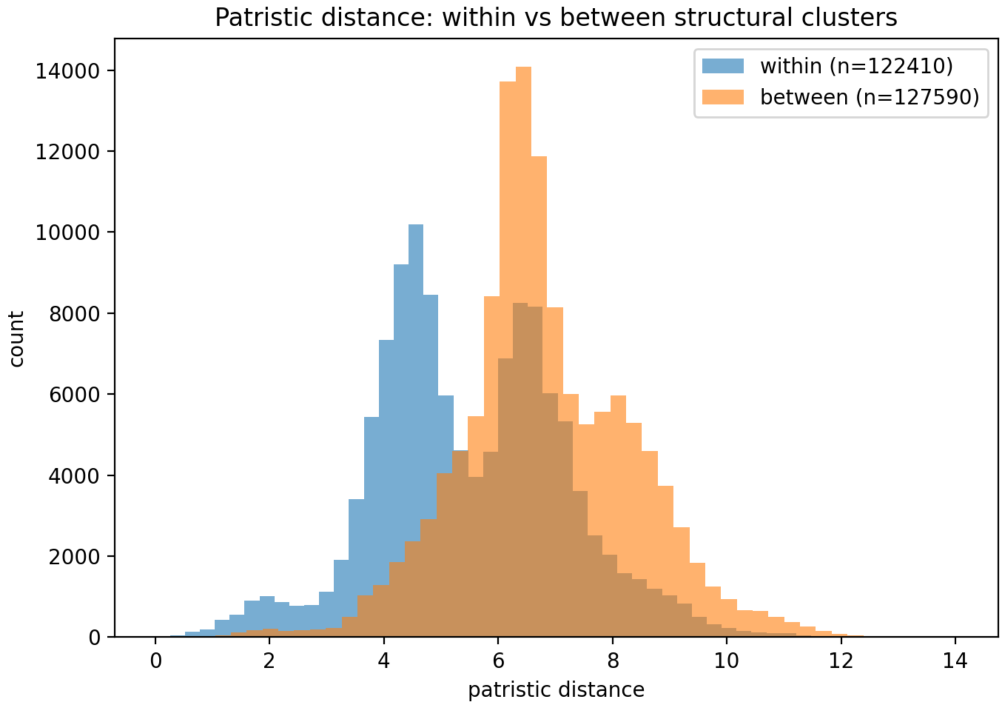

Figure S13. *Distribution of patristic distances for labeled tip pairs within the same structural cluster versus between clusters. A right-shift of the between-cluster distribution relative to within-cluster distances supports evolutionary separation between the dominant structural regimes.*

5. Triphosphatases: $P_\alpha P_\beta P_\gamma$ leaving group

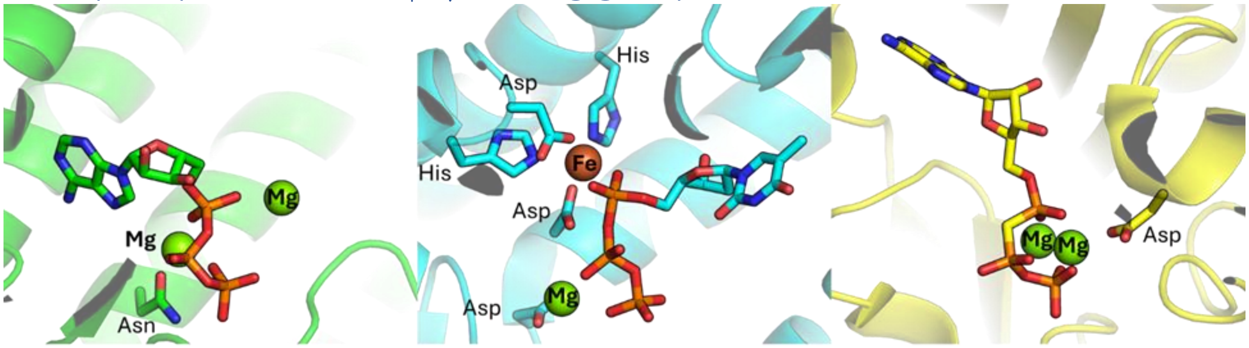

Figure S14: *Examples of versatile ion-coordination modes observed in triphosphatases. Shown are the Cobalamin adenosyltransferase–like (green; PDB: 6D5K), HD-domain/PDEase-like (cyan; PDB: 6TXE), and S-adenosylmethionine synthetase (yellow; PDB: 6VD0) SFs. Ion coloring used elsewhere to denote coordination modes is not applied here; instead, ions are colored according to their element to highlight chemical identity.*

6. Non-Mg^{2+} cofactors

Domain length distributions

Python codes used for analysis and corresponding metadata are available at https://github.com/Rosta-Research-Group/Nature-counts-to-three.

Relating CATH Domain Lengths to Reaction Class and Number of Active-Site Metals

We quantified domain lengths from the CATH database (release v4.4.0; file date 15-Jan-2025).[20-21] The following bulk files were used:

- cath-superfamily-list.txt — superfamily IDs and names.
- cath-domain-list.txt (CLF format 2.0) — per-domain class/architecture/topology/superfamily (C,A,T,H) numeric codes.
- cath-domain-boundaries-seqreschopping.txt — domain boundary segments defined on the PDB **SEQRES** sequence coordinates.

We used **SEQRES** boundaries because domain identifiers in our CLF file (e.g., 1jqoA03) matched the SEQRES naming scheme (with two-digit domain suffixes). ATOM-boundary files from some releases use legacy domain IDs and do not always intersect with the CLF IDs.

Mapping curated superfamilies to CATH. We started from our curated SF list with their reaction class and the number of active-site metals identified containing 61 superfamilies which have SUPFAM fold identification (not only through InterPro) and metal ion counts in the active site (all that have a clearly identified representative with well-defined coordination, and also *RibA-like* and *DNA ligase/mRNA capping enzyme, catalytic domain* SFs that have 2 metal ions in the active site yet with unclear coordination).

Enumerating domains per superfamily. From cath-domain-list.txt (CLF), we reconstructed each domain's full CATH code as class.arch.topology.superfamily using the first five columns (domain ID, then C, A, T, H). For a given superfamily ID (e.g., 1.20.1440.90), we selected all domains whose reconstructed code started with that ID. For each domain, we parsed the boundary string and summed the lengths of all segments. The parser accepts both chain-labelled segments (A:12-98,B:120-210) and unlabelled ranges (313-449, 10-50,60-90). Domain length was defined as

$$L = \sum_{\text{segments}} (\text{end} - \text{start} + 1).$$

If a domain had multiple discontinuous segments, our length is the sum across segments (the conventional CATH definition for domain extent).

Discontinuous domains were therefore counted as the total extent of all CATH-assigned fragments. The resulting per-domain lengths represent the distribution of actual CATH-curated PDB-derived structural instances belonging to that superfamily.

Result: domain length is not associated with reaction class or metal count

Across all superfamilies in our curated set, we did **not** observe a systematic dependence of domain length on either (i) reaction class (Pi vs PPi) or (ii) the number of active-site metal ions. The median domain lengths for Pi and PPi were comparable, with overlapping interquartile ranges (Figure S15). The number of active site metal ions (1, 2, or 3) did not shift median domain length; distributions overlapped substantially (Figure S16). Within each reaction class, the between-superfamily variability dominated over any trend with the number of ions (Figure S17).

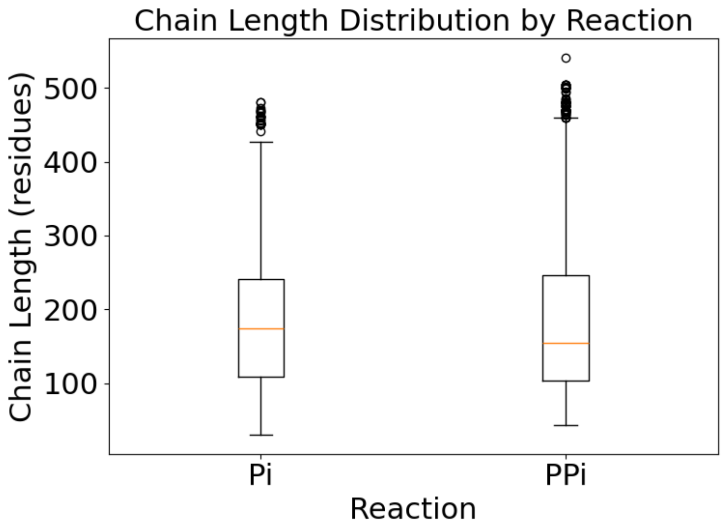

Figure S15: *Distribution of domain lengths for Pi and PPi enzyme SFs.*

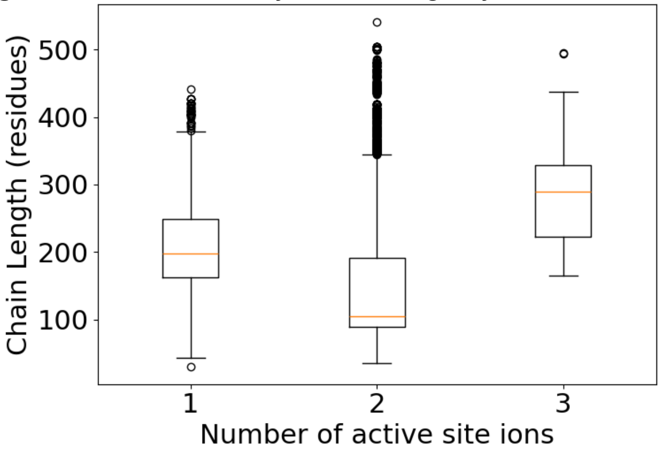

Figure S16: *Chain lengths distribution of enzyme SFs using 1, 2 or 3 active site metal ions.*

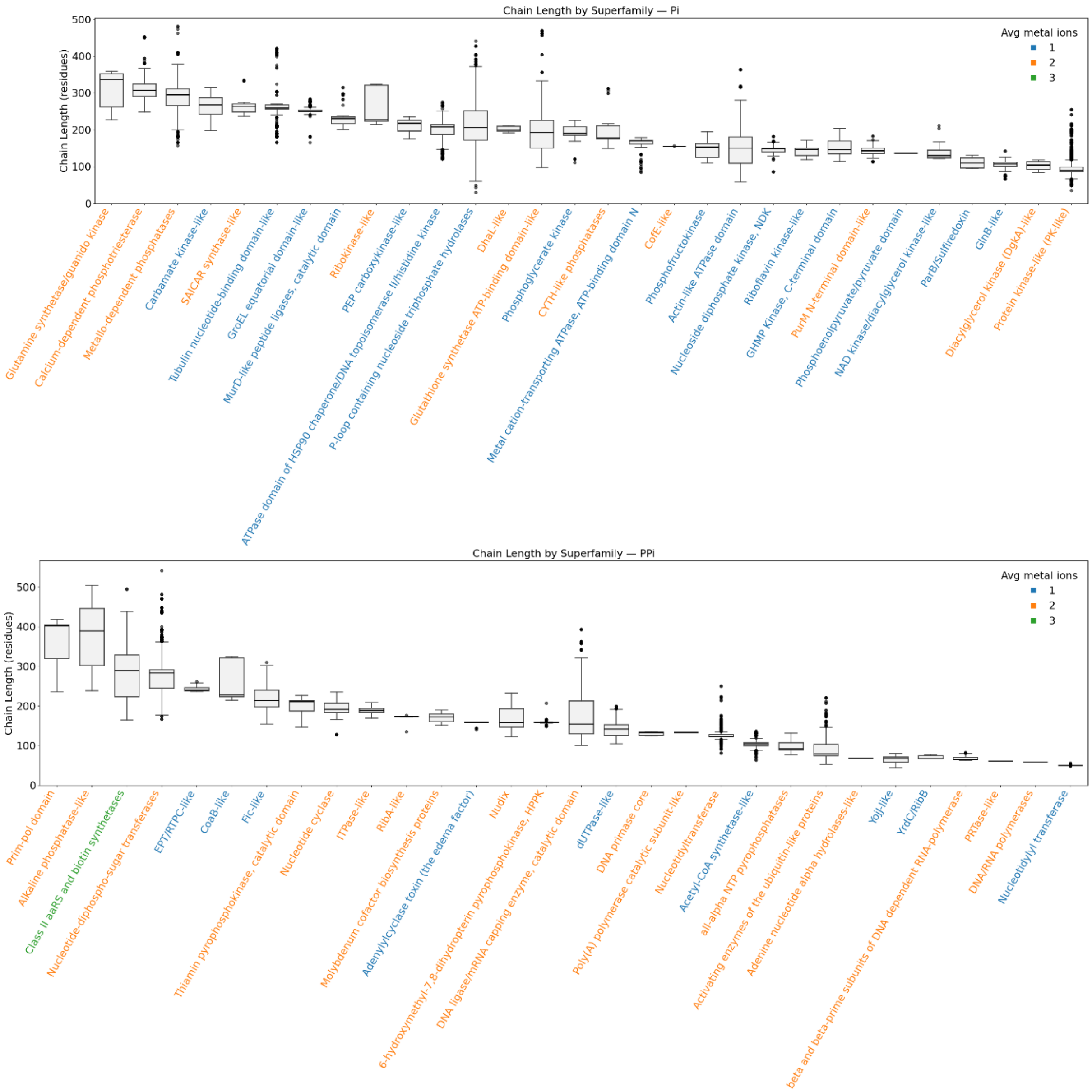

Figure S17: *Chain length distribution for NTP processing SFs with Pi (top) and PPi (bottom) leaving groups. Number of active-site metal ions used is colour coded (1: blue, 2: orange, 3: green).*

These observations indicate that, within this CATH-based analysis of domain extents, there is no evidence that binding multiple metals requires systematically longer domains. The active site architecture tends to be a local feature of the domain, therefore the analysis is likely valid across the full length of the proteins. Nevertheless, our results do not necessarily preclude longer linkers, multi-domain architectures, or RNA cases; here we measure protein domain lengths as defined by CATH. CATH boundaries capture domain extents, not full-length polypeptides or multi-domain assemblies. Some enzymes that bind multiple metals may recruit charge from neighbouring domains or oligomer interfaces, which is not reflected in a single-domain length. Superfamily name → CATH ID mapping was done via manual curation and it is not a complete equivalence.

Relating SCOPe Superfamily Full Protein Lengths to Reaction Class and Number of Active-Site Metals

To complement the domain-length analysis performed using the CATH database, we conducted a parallel study using SCOPe Superfamily (SSF) identifiers linked to UniProt[22-23] full-length protein sequences. This approach allowed a broader comparison between catalytic domain organization (CATH) and full protein architectures (SSF–UniProt). For each curated SSF we separately also queried the InterPro mapping and retrieved (i) all and (ii) up to the first 1000 UniProt protein accessions to analyze the overall distributions from (i), and the long protein sequences from (ii). Each accession was then annotated with (a) reaction class (Pi vs PPi) and (b) number of active-site metal ions by joining to the same manually curated Reaction/SF table used for the structural analysis. Sequence lengths were taken directly from the downloaded UniProt records, so the values reported here correspond to the full polypeptide length computed from the local snapshot of UniProt, not to a PDB-truncated construct.

SSF-based analysis details

The SSF-based analysis was implemented as an automated pipeline. UniProt accessions associated with each SSF were retrieved via the InterPro REST API. Subsequently, to obtain sequence lengths, the corresponding protein entries were searched directly from the downloaded UniProt records for the full dataset (i), and queried through the UniProt REST API for dataset (ii). Each UniProt sequence was annotated with its reaction class (Pi or PPi) and the number of active-site metal ions based on curated mappings from the primary dataset as previously done.

SSF-based analysis results

The SSF–UniProt analysis confirmed and extended the main observations derived from domain-level (CATH) data. Across all 61 SFs, we analyzed a total of 25,656,986 protein sequence entries. The bulk of full-length sequences falls in the same interval as the CATH domain-based view: most proteins are in the ~400–600 aa range, with broad overlap between Pi-releasing and PPi-releasing enzymes. Changing the number of active-site metals from 1 to 2 or 3 did not shift the median length in a systematic way; within each reaction class, the variation between superfamilies was larger than the variation between ion counts (Figures S18–S21). This mirrors the domain-level results: metal-binding chemistry is predominantly a local feature.

When we ranked all locally retrieved sequences by length (dataset (i)) and inspected the longest 100 entries, a small number of extremely long sequences (≥ 35–40 k aa) appeared. These are much longer than biologically validated giant proteins such as human titin (~34 k aa),[24] which strongly suggests that they arise from automatic gene-prediction or annotation artefacts (e.g. fused ORFs, untrimmed low-complexity regions, or incomplete curation of TrEMBL-only entries).[22-23, 25-26]

The biologically relevant long outliers (>10 000 aa), as observed in the top 100 proteins in (ii) dataset, derive predominantly from bacterial and eukaryotic megasynthases, such as non-ribosomal peptide or polyketide synthetases, and from multidomain adenylate-forming enzymes involved in toxin or secondary-metabolite biosynthesis.[27-28] Consistent with this, ~80% of the longest proteins mapped to only two superfamilies, ***Acetyl-CoA synthetase-like*** and ***Adenylylcyclase toxin (edema factor)***, indicating that the extreme sequence sizes mainly reflect modular, assembly-line enzymatic architectures.

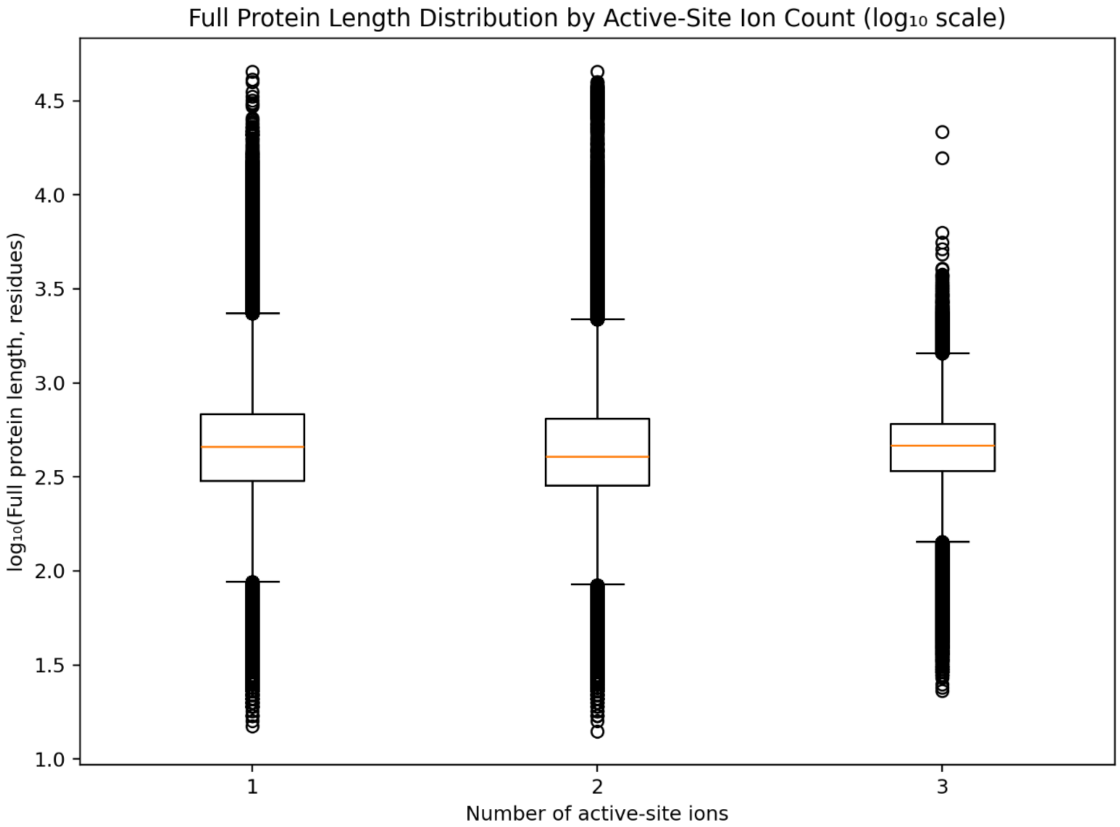

Figure S18: *Full protein length distributions by active-site ion count (SSF–UniProt).*

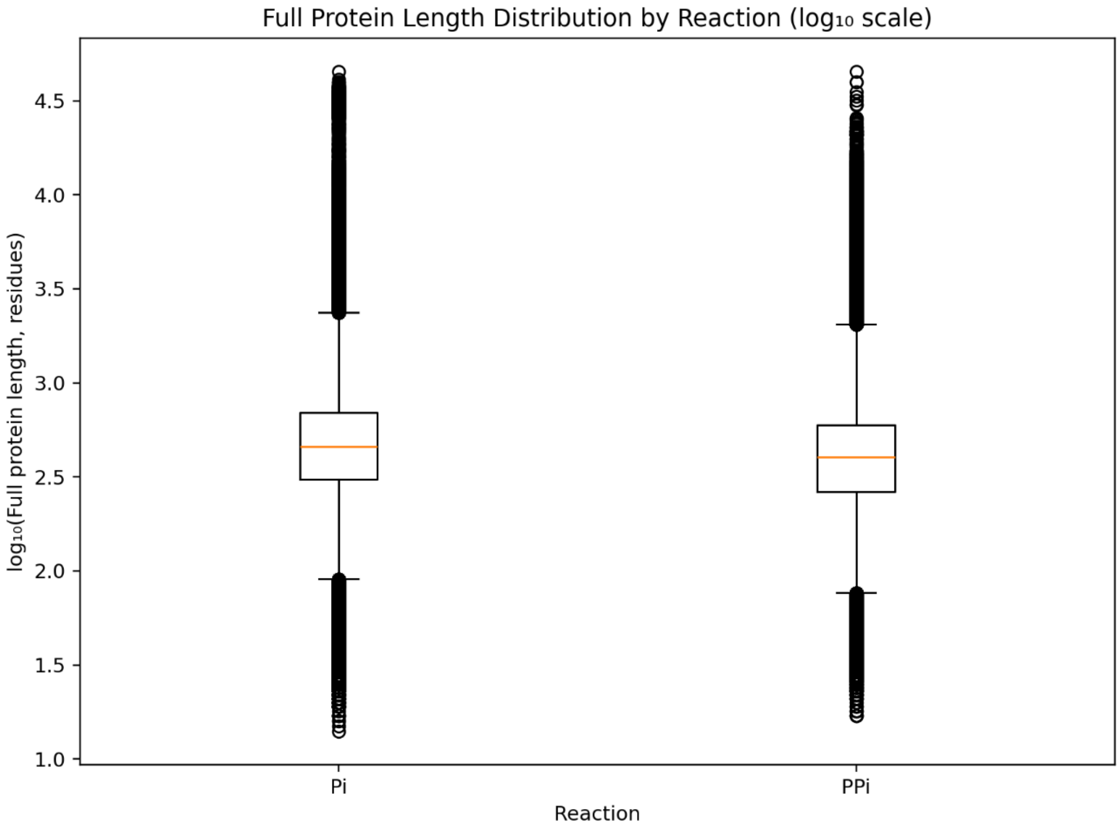

Figure S19: *Full protein length distributions by reaction type (Pi vs PPi).*

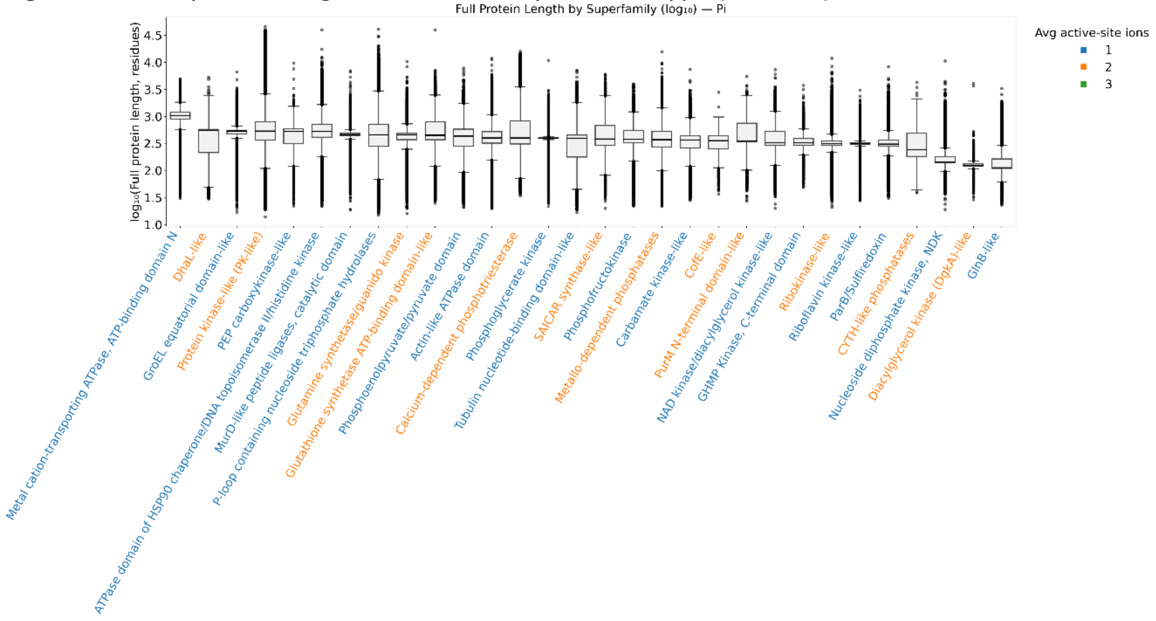

Figure S20: *Protein length distributions by superfamily for Pi-releasing enzymes.*

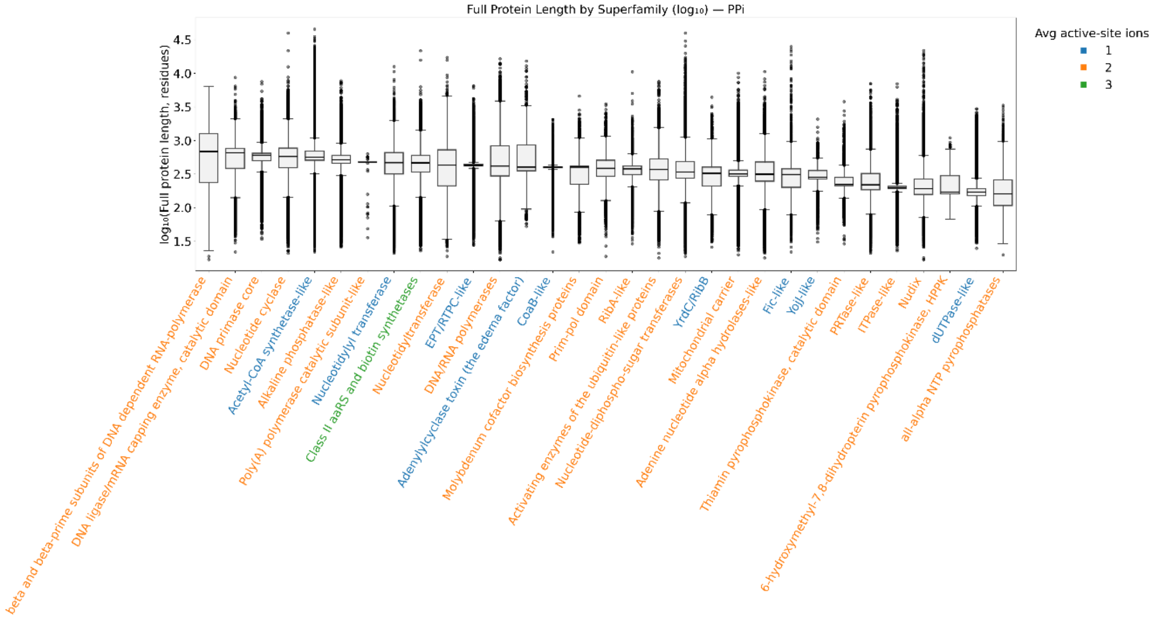

Figure S21: *Protein length distributions by superfamily for PPi-releasing enzymes.*

Comparison with Domain-Level Analysis

The CATH- and SSF-based analyses provide complementary perspectives. The CATH approach isolates catalytic domains and emphasizes the conserved structural core, while the SSF–UniProt framework contextualizes those domains within their full-length biological assemblies. Despite these differences, the two datasets reveal congruent trends: there is no significant difference in the enzyme or domain length based on the number of active site ions. It is also notable that PPi-releasing enzymes tend to use two-metal ion catalytic mechanisms more often.

Metal ion coordinating residues

Additionally, we analyzed the coordinating protein residues around the catalytic metal ions among the 71 identified representative structures (Table S3). When selecting representative structures, we prioritized cases that exhibited complete first-shell coordination wherever possible. SFs that do not rely on Mg^{2+} but instead use transition-metal catalytic cofactors such as zinc, iron, or cobalt often feature atypical coordinating residues. Coordinating histidine residues were identified in the previously discussed *Metallo-dependent phosphatases*, *Alkaline phosphatase–like*, and *Ca-dependent phosphotriesterase* SFs. In the rare case of the *tRNA-splicing ligase RtcB-like* SF, a cysteine residue coordinates both Mn^{2+} ions. Interestingly, histidine coordination also occurs in several Mg^{2+}-dependent SFs: *Glutamine synthetase/guanido kinase* among the phosphatases, and *PRTase-like* as well as *Adenylylcyclase toxin (the edema factor)* among the pyrophosphatases (Figure S22).

Table S3. *Protein residues involved in the coordination of the Mg-pinch forming ion.*

	Asp	Glu	Ser	Thr	Asn	backbone CO	His
Pi	16	12	4	4	4	1	3
PPi	40	8	2	0	0	5	6
total	56	20	6	4	4	6	9
ratio	53%	19%	6%	4%	4%	6%	9%

Carboxylate residues (aspartate and glutamate) are the most prevalent protein ligands for both phosphatases and pyrophosphatases (Table S3). Other oxygen donors also contribute to Mg^{2+} coordination: serine and threonine sidechains appear four times each in phosphatases, whereas only two serine residues are found in pyrophosphatases. Occasional carbonyl coordination is observed via asparagine (four instances in phosphatases) or backbone carbonyl oxygens (six instances, primarily in pyrophosphatases). Finally, water molecules frequently complete the octahedral coordination sphere of Mg^{2+} (63 water molecules in 33 phosphatases; 59 in 38 pyrophosphatases).

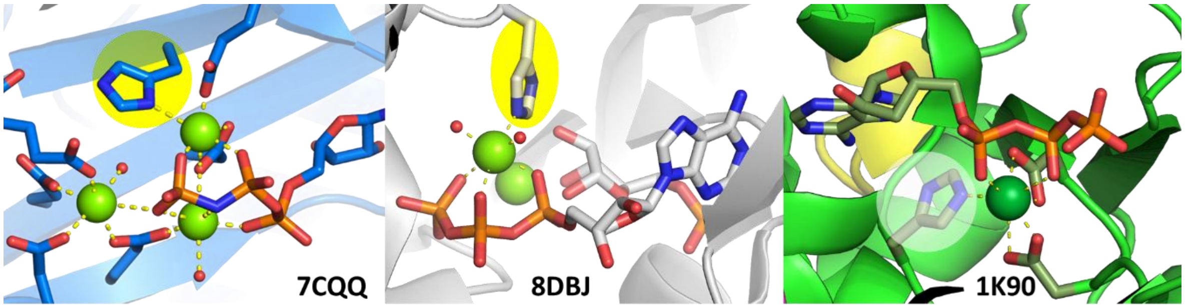

Figure S22: *Mg^{2+}-coordinating histidine residues (highlighted) in selected SFs: Glutamine synthetase/guanido kinase (blue; PDB: 7CQQ), PRTase-like (white; PDB: 8DBJ), and Adenylylcyclase toxin (the edema factor) (green; PDB: 1K90).*

7. Analysis on the protein fold level

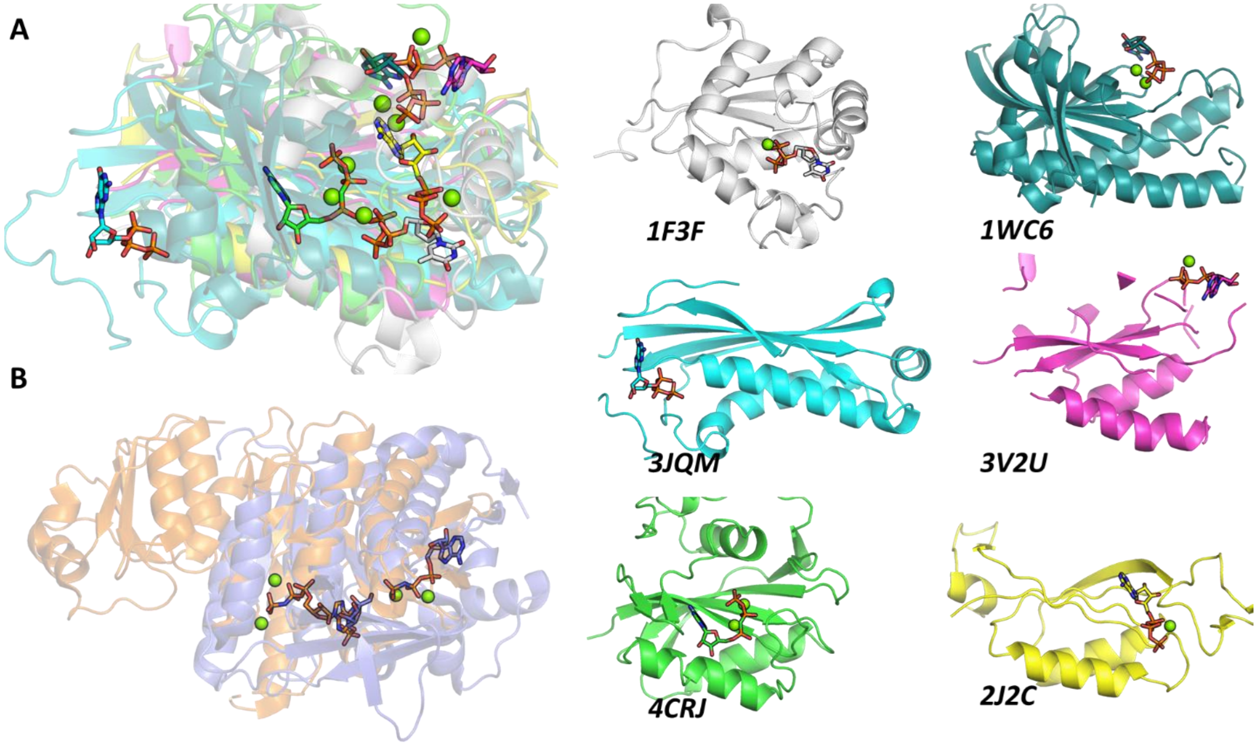

Figure S23: *Examples of distinct NTP-binding sites found in superfamilies (SFs) belonging to the same structural fold.*
***A**. Six SFs from the Ferredoxin-like fold: Nucleoside diphosphate kinase, NDK (white; PDB: 1F3F); Nucleotide cyclase (teal; PDB: 1WC6); Molybdenum cofactor biosynthesis protein C, MoaC (cyan; PDB: 3JQM; no available metal-bound structure); GHMP kinase, C-terminal domain (magenta; PDB: 3V2U); 6-hydroxymethyl-7,8-dihydropterin pyrophosphokinase, HPPK (green; PDB: 4CRJ); and GlnB-like proteins (yellow; PDB: 2J2C).*
B. *Two SFs from the Ribokinase-like fold—Ribokinase-like (blue; PDB: 2JG1) and MurD-like peptide ligases, catalytic domain (orange; PDB: 6CAU)—both of which function as phosphatases with a Mg^{2+} ion at the BG(–) position, yet possess active sites located in different regions of the fold.*

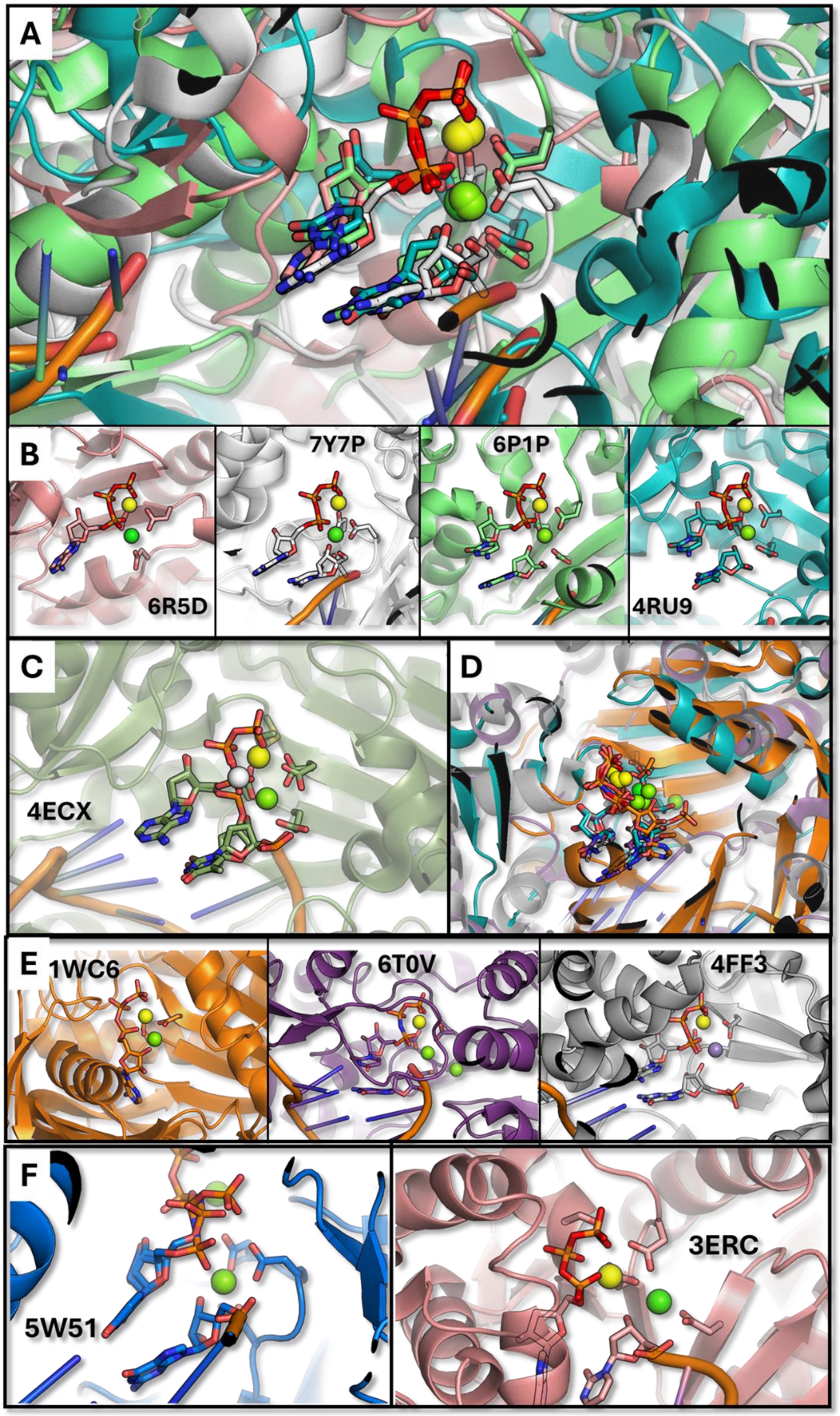
A
B
7Y7P
6P1P
6R5D
4RU9
C
4ECX
D
E
1WC6
6T0V
4FF3
F
5W51
3ERC

Figure S24. *Two-metal ion catalytic enzymes, pinching ion is shown in yellow spheres.* ***A–B:*** *Examples of polymerases from distinct structural folds—Prim-pol domain (6R5D, salmon), RNA-dependent RNA polymerase, eukaryotic type (7Y7P, white), nucleotidyltransferase (6P1P, green), and DNA/RNA polymerase (4RU9, cyan). Despite their divergent overall architectures, these enzymes exhibit strikingly similar active-site metal-ion coordination geometries, even though the coordinating amino-acid side chains originate from different structural elements.*
C: *Example of a transient third metal-ion position (white sphere) observed in a DNA/RNA polymerase (4ECX, teal). Time-resolved crystallographic studies have shown that, in addition to the canonical two catalytic metal ions, a third divalent metal ion may transiently associate with the active site during catalysis or immediately thereafter, stabilizing highly charged intermediates or products.*[29-30]
D-E: *Additional polymerase structures displaying analogous active-site geometries and similar structural motifs/folds to DNA/RNA polymerase (4RU9, cyan) include nucleotide cyclase (1WC6, orange), influenza RNA-dependent RNA polymerase subunit PB1 (6T0V, purple), and virion DNA-directed RNA polymerase domain (4FF3, grey).*
F: *Active site structures of the additional representative enzymes predicted to harbor two-metal catalytic arrangement: RPB5-like RNA polymerase subunit (5W51, blue) and Poly(A) polymerase catalytic subunit-like (3ERC, salmon) SFs.*

8. Analysis of the EC category distributions

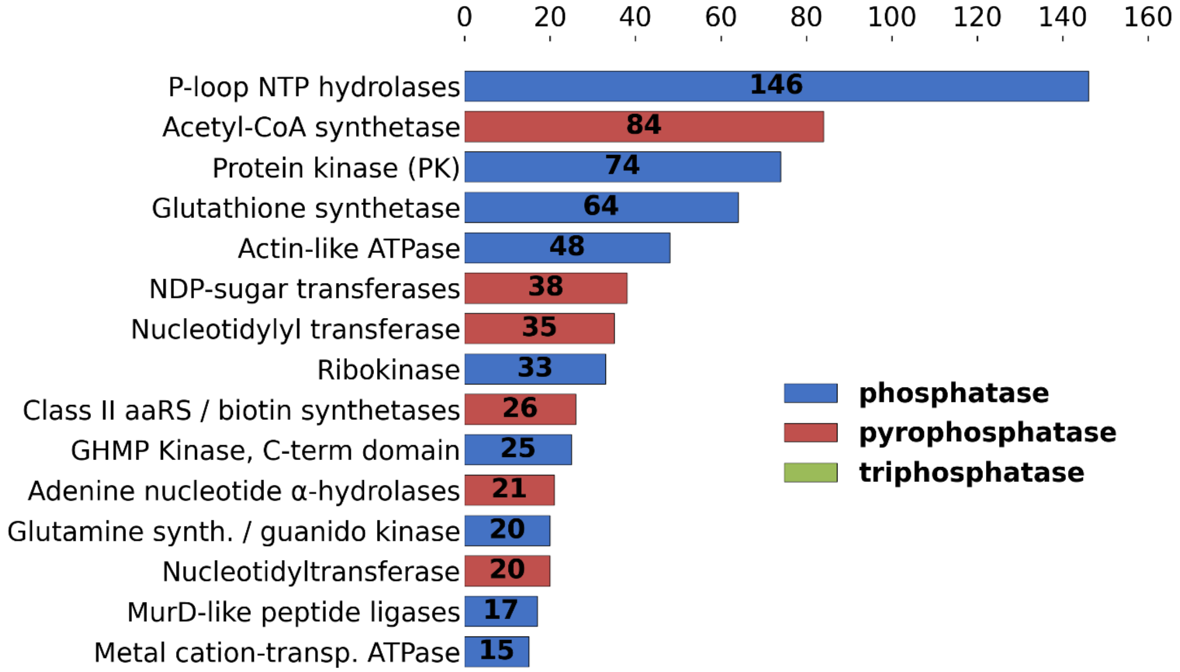

Figure S25: *Number of EC categories assigned to structures and sequences of superfamilies, grouped by reaction type: phosphatases (blue), pyrophosphatases (red), and triphosphatases (green, none in the top 15). The full lists are provided in Note 1.*

To provide an approximate functional assignment, we used the EC categories associated with the sequences collected from the sources used in Figure S1 — predominantly InterPro,[5] which propagates EC annotations across multi-domain sequences and across protein-family members. We observed that such annotations often correspond to reactions catalyzed by different domains, or even different chains within the same protein complex. Thus, when multiple domains are present, EC annotations may be propagated to all domains within a chain, even if only one domain actually carries out the annotated reaction. We ranked the most prevalent SFs according to their assigned EC functions (Figure S25).

Misannotations were frequently encountered. For instance, EC 2.7.7.6 (polymerase activity) is sometimes incorrectly associated with the *P-loop NTPase* domain because replication complexes include P-loop NTPase helicase chains.[31] Upon manual inspection, all such pyrophosphatase assignments for the *P-loop NTPase* SF proved incorrect and have been removed. Similarly, phosphatase EC assignments previously linked to the *Nucleotidylyl transferase* SF often correspond to separate kinase domains encoded in the same protein—for example, in a reported prokaryotic FAD synthetase where the N-terminal domain is a kinase and the C-terminal domain is a *Nucleotidylyl transferase*.[32] Among *CYTH-like phosphatases*, both phosphatase and pyrophosphatase activities are identified in the literature, due to the adenylyl cyclase class IV pyrophosphatases that possess a CYTH domain.[33-34] Interestingly, the *PK-like* SF is also verifiably associated with adenylyltransferase activity through flipped pseudokinases in the literature.[35-36] After applying these corrections, each SF in our dataset is associated with a single dominant phosphate-leaving-group category (Figure S25). The Mg-pinch coordination subtype distributions are summarized in Figures S26A and S26B by SF and by EC, respectively. Figure S26B shows that βγ(−) is the most common coordination geometry among NTP-processing EC–SF associations (387 of 928), driven primarily by the *P-loop NTPase* superfamily.

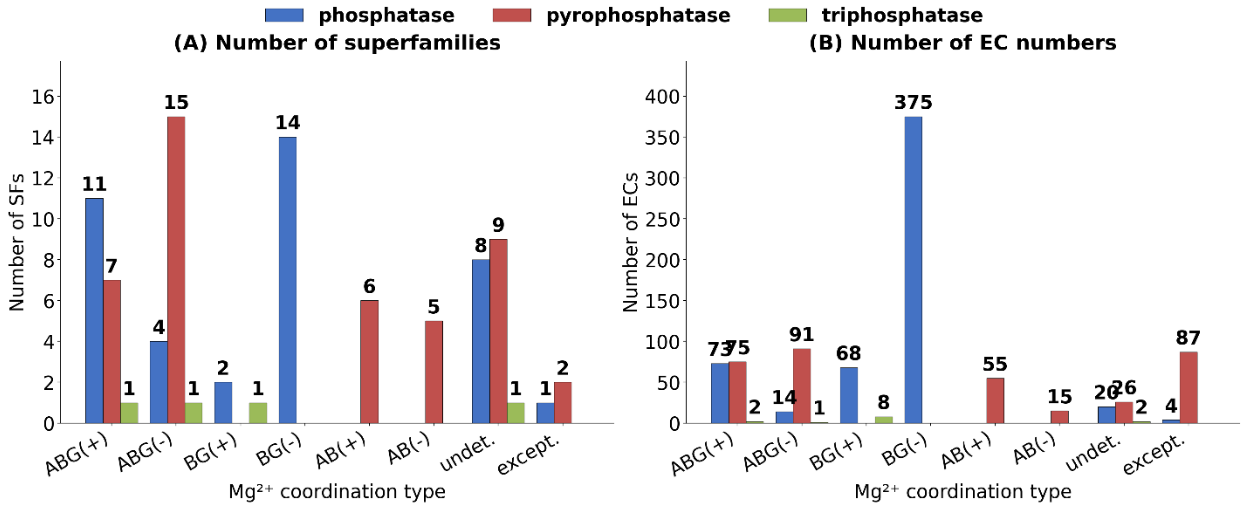

Figure S26: *Distribution of NTP-processing superfamilies (**A**) and their associated EC numbers (**B**) across the seven Mg^{2+}-pinch coordination categories: ABG(+), ABG(−), BG(+), BG(−), AB(+), AB(−), undetermined ("undet.", SFs without an unambiguous coordination assignment, Table 3), and the few exceptional SFs ("except.", e.g., Acetyl-CoA synthetase-like, Alkaline phosphatase-like, Metallo-dependent phosphatases). Bars are colour-coded by leaving-group category: phosphatase (blue), pyrophosphatase (red), triphosphatase (green). Some EC numbers are associated with more than one SF and are counted once per SF assignment; the total EC counts in panel B therefore exceeds the unique EC numbers in our dataset. αβ-type coordinations are exclusive to pyrophosphatases; βγ-type coordinations are nearly exclusive to phosphatases (with one triphosphatase exception, HD-domain/PDEase-like); αβγ coordinations are shared between both classes. By SF count, ABG(+) and ABG(−) are the most populous coordination types, whereas by EC count, BG(−) dominates owing to the very large P-loop NTPase superfamily.*

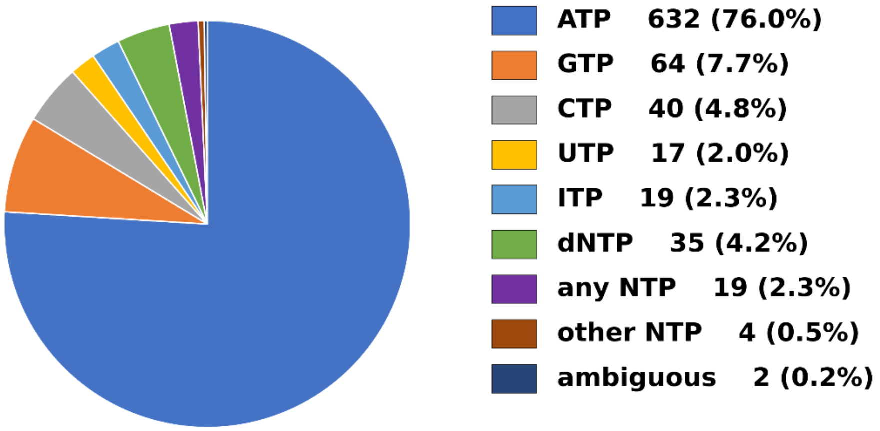

Figure S27. *Substrate distribution among NTP processing enzymes based on the EC reactions. Note that one EC may correspond to multiple substrates.*

Increased structural diversity — reflected by the number of associated SFs — is also observed among certain non-specific kinases, including ECs 2.7.11.1, 2.7.10.2, and 2.7.13.3, which are associated with 5, 4, and 4 SFs, respectively. Some EC numbers correspond to general reactivity categories and appear in both phosphatase and pyrophosphatase SFs, such as EC 3.6.1.15 (nucleoside-triphosphate phosphatase).

Table S4. *Enzyme Commission categories with members assigned to four or more superfamilies. Polymerase-associated ECs are highlighted in purple, and kinase-associated ECs in pink. Enzyme Commission categories with members assigned to four or more superfamilies. Polymerase-associated ECs are highlighted in purple, and kinase-associated ECs in pink. The non-specific kinase ECs (2.7.11.1, 2.7.13.3, 2.7.10.2) reflect cases where databases propagate a generic kinase identifier across many structurally distinct folds rather than truly different chemistries; the polymerase-associated ECs reflect genuine convergent evolution of nucleotidyltransfer chemistry across multiple folds (e.g., DNA-directed RNA polymerase 2.7.7.6 spans both the β/β′ subunits of DNA-dependent RNA polymerase fold and the DNA/RNA polymerases fold).*

EC	EC name	No. of SFs	Category
2.7.11.1	non-specific serine/threonine protein kinase	11	kinase
2.7.13.3	histidine kinase	7	kinase
3.6.1.15	nucleoside-triphosphate phosphatase	7	Other (Pi)
4.6.1.1	adenylate cyclase	7	Other (PPi)
2.7.7.48	RNA-directed RNA polymerase	6	polymerase
2.7.10.2	non-specific protein-tyrosine kinase	5	kinase
2.7.4.1	ATP-polyphosphate phosphotransferase	4	Other (Pi)
2.7.7.19	polynucleotide adenylyltransferase	4	polymerase
6.4.1.8	acetophenone carboxylase	4	Other (Pi)

9. The effect of Mg^{2+} analyzed through QM/MM and QM calculations

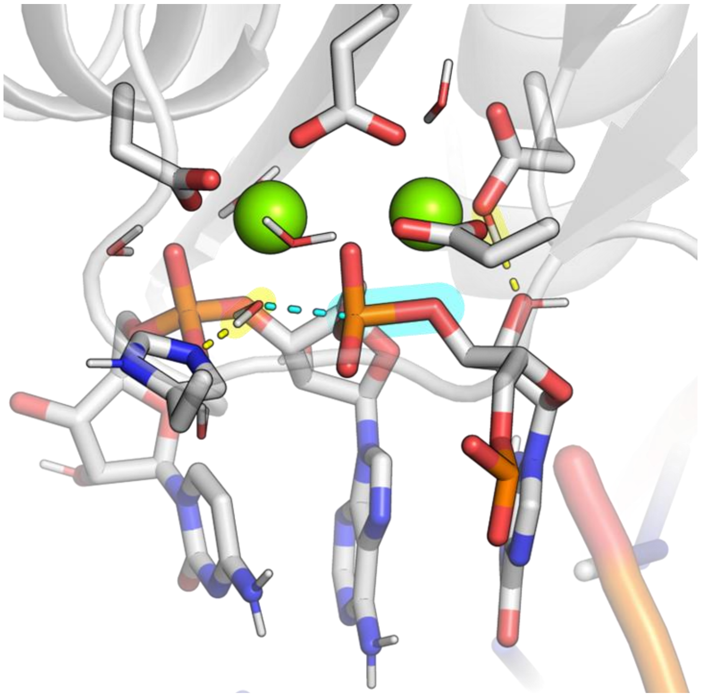

Figure S28: *Active site of the HIV-1 ribonuclease H (based on Ref [7]). The cleaved phosphodiester bond is highlighted in cyan, associated proton transfers are highlighted in yellow.*

Model system results

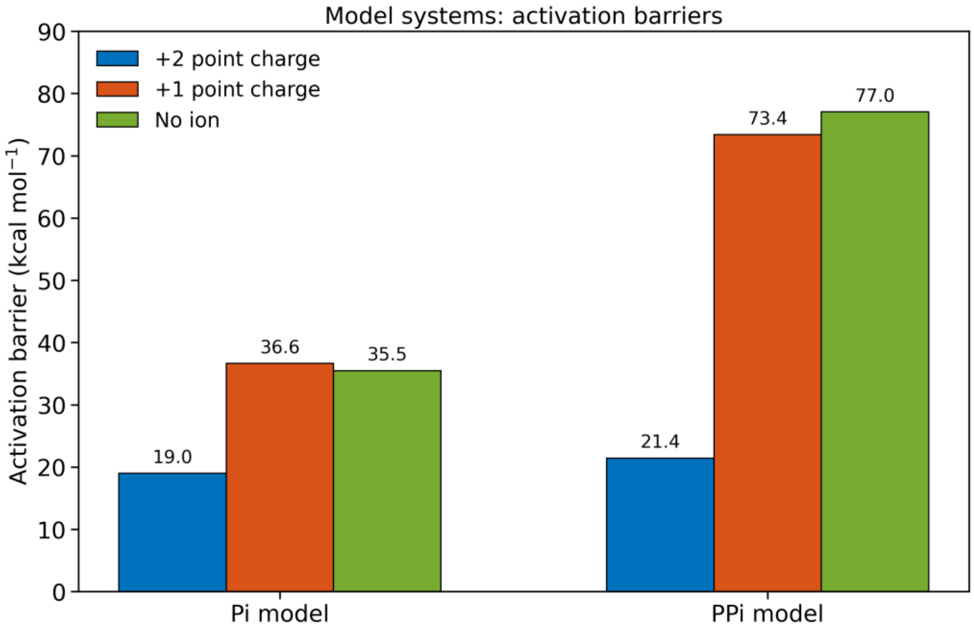

Figure S29: *Activation barriers for the Pi and PPi minimal model systems with a +2 point charge, a +1 point charge, or no ion.*

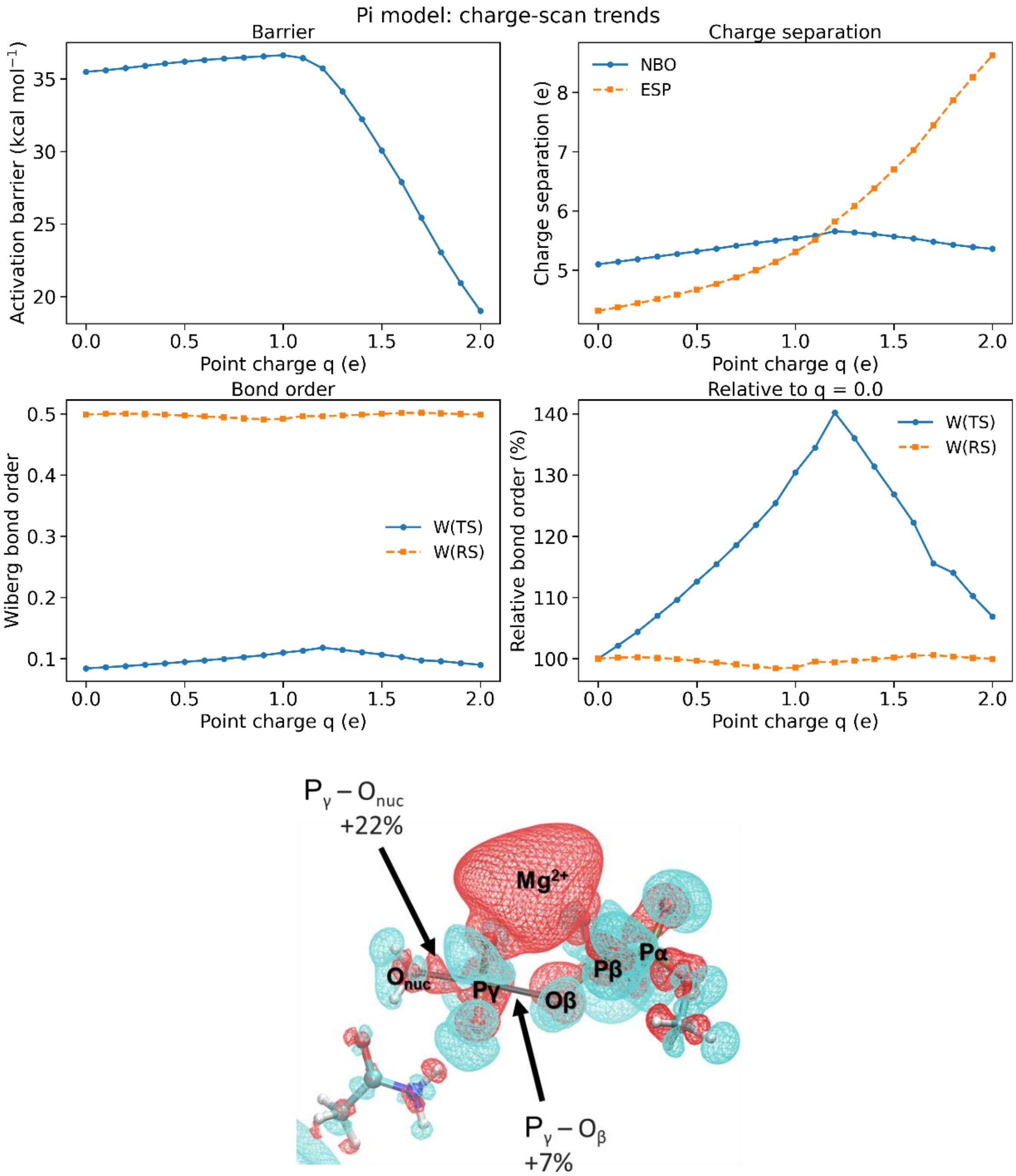

Figure S30: ***top:*** *Charge-scan trends for the Pi model system: activation barrier, charge separation using NBO and ESP atomic charges, Wiberg bond order of the breaking bond and relative bond order as a function of point charge at the RS (orange) and TS (blue).* ***bottom****:Electron density difference for Pi model system between q=+2 and q=0 point charges at the* Mg^{2+} *position.*

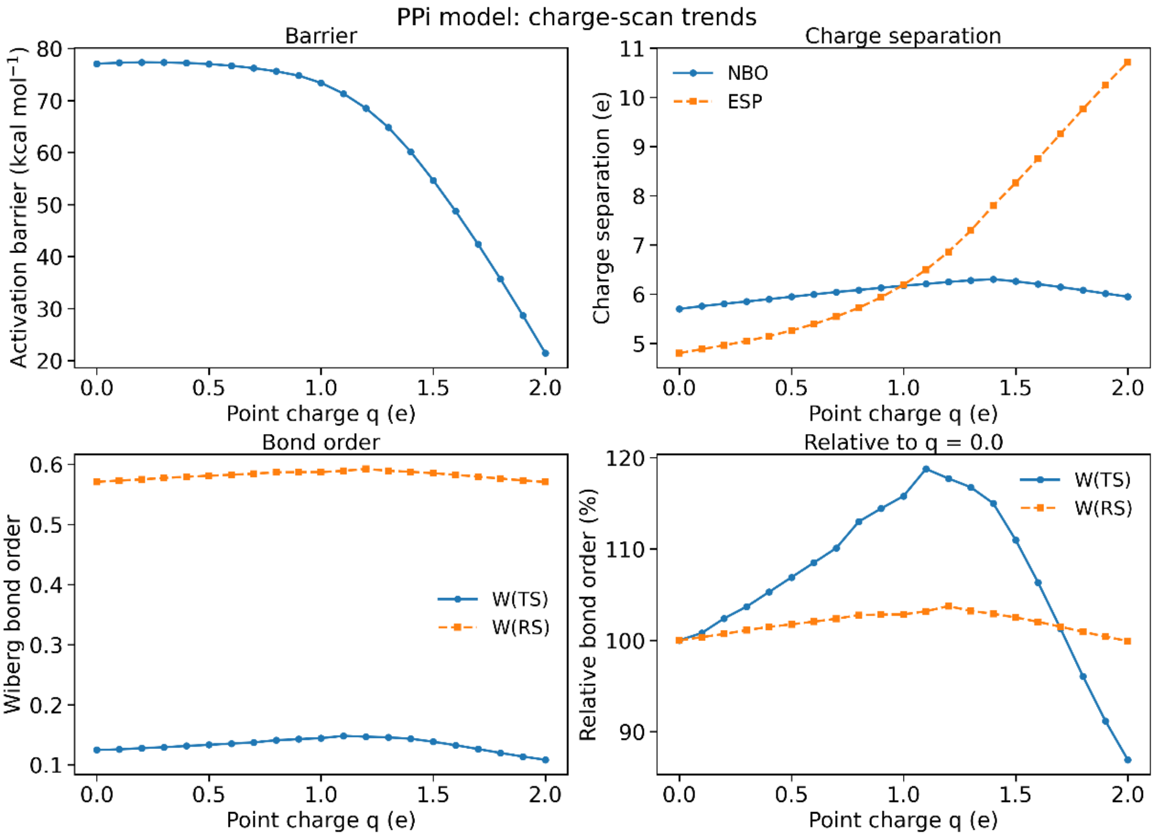

Figure S31: *Charge-scan trends for the PPi model system: activation barrier, charge separation using NBO (blue) and ESP (orange) atomic charges, Wiberg bond order of the breaking bond and relative bond order as a function of point charge at the RS (orange) and TS (blue).*

Electrostatic Stabilization and Polarization Contributions to Catalysis in the Model Systems

To quantify the electrostatic contribution of the metal ion to transition-state stabilization, we evaluate the interaction between the electronic density and an external probe charge placed at the metal position.

We consider two fixed geometries: $s \in (\mathrm{RS}, \mathrm{TS})$, and two charge states: $q = 0$ (no probe charge) and $q = +2$ (point charge mimicking Mg^{2+}). For each state s and charge q, the self-consistent electronic density is denoted $\rho_s^{(q)}$. The electrostatic potential generated by a given density and the nuclei at the probe position R_q is:

$$\Phi[\rho; R_q] = \sum_A \frac{Z_A}{|R_A - R_q|} - \int \frac{\rho(r)}{|r - R_q|}\, dr$$

The electrostatic interaction energy between this density and a probe charge q is $E_{\mathrm{el}}[\rho; q] = q\,\Phi[\rho; R_q]$. For each charge state, the electronic energy is:

$$E_s^{(q)} = F_s[\rho_s^{(q)}] + q\,\Phi[\rho_s^{(q)}; R_q]$$

The activation barrier is $\Delta E^{\ddagger}(q) = E_{\mathrm{TS}}^{(q)} - E_{\mathrm{RS}}^{(q)}$ and the barrier change induced by the charge is $\Delta\Delta E^{\ddagger} = \Delta E^{\ddagger}(2) - \Delta E^{\ddagger}(0)$.

For the model systems, we obtain $\Delta\Delta E^{\ddagger}$ values of -16.5 and -55.6 kcal/mol for Pi and PPi systems, respectively. To decompose these terms with respect to the polarization and electrostatic components, we define two cases.

(a) Frozen-density electrostatic contribution

This corresponds to applying the $+2$ charge to the unperturbed ($q = 0$) densities, measuring pure static-field effects:

$$\Delta E_{\mathrm{frozen}}^{\ddagger} = 2\left[\Phi[\rho_{\mathrm{TS}}^{(0)}; R_q] - \Phi[\rho_{\mathrm{RS}}^{(0)}; R_q]\right].$$

(b) Polarized electrostatic contribution

This uses the **self-consistent densities in the presence of the charge**:

$$\Delta E_{\mathrm{pol}}^{\ddagger} = 2\left[\Phi[\rho_{\mathrm{TS}}^{(2)}; R_q] - \Phi[\rho_{\mathrm{RS}}^{(2)}; R_q]\right],$$

representing the full electrostatic stabilization after electronic relaxation.

The gain due to polarization is $\Delta E_{\mathrm{pol}}^{\ddagger} - \Delta E_{\mathrm{frozen}}^{\ddagger}$, this isolates the effect of wavefunction relaxation. Next, we define the electronic reorganization penalty as:

$$\Delta E_{\mathrm{reorg}}^{\ddagger} = \Delta\Delta E^{\ddagger} - \Delta E_{\mathrm{pol}}^{\ddagger}$$

This term captures the internal energetic cost of polarization. The numerical results are summarized in Table S5, the $\Delta E_{\mathrm{pol}}^{\ddagger}, \Delta E_{\mathrm{frozen}}^{\ddagger}$ values for varied point charges are shown in Fig. S32.

Table S5. Decomposition of electrostatic and polarization contributions to the activation barrier (kcal mol^{-1}).

Model	$\Delta E_{\mathrm{frozen}}^{\ddagger}$	$\Delta E_{\mathrm{pol}}^{\ddagger}$	$\Delta E_{\mathrm{pol}}^{\ddagger} - \Delta E_{\mathrm{frozen}}^{\ddagger}$	$\Delta\Delta E^{\ddagger}$	$\Delta E_{\mathrm{reorg}}^{\ddagger}$
**PPi**	−2.23	−153.99	−151.76	−55.6	+98.4
**Pi**	−33.92	−76.89	−42.97	−16.5	+60.4

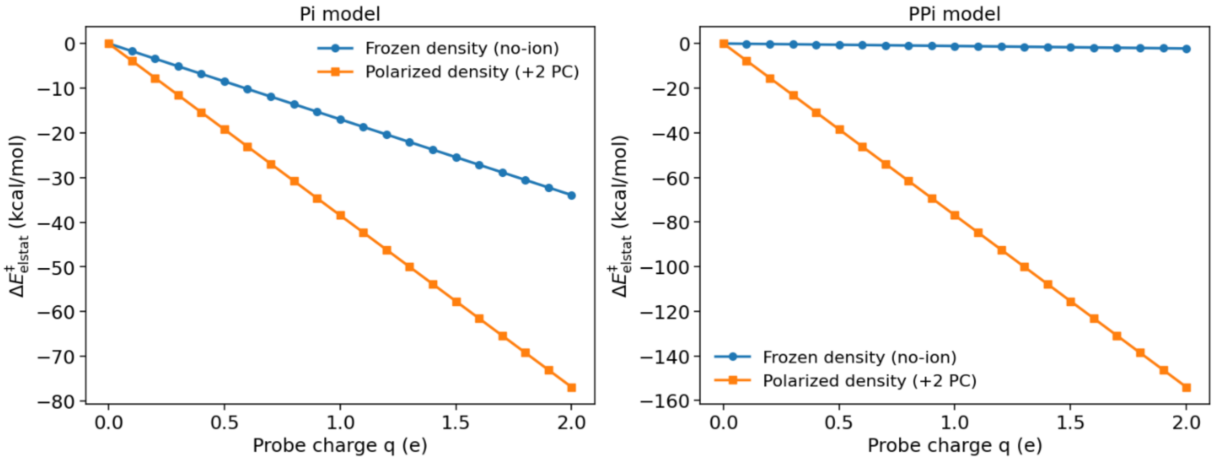

Figure S32: *Electrostatic stabilization in the enzyme model: frozen versus polarized density for the Pi (left) and PPi (right) models. The electrostatic contribution to TS stabilization is plotted as a function of probe charge, comparing the frozen no-ion density (blue) with the density polarized in the presence of a +2 point charge (orange). Polarization leads to a dramatic increase in electrostatic stabilization for PPi. The Pi model reproduces the same qualitative trend, with substantially stronger transition-state stabilization for the polarized density.*

Both the Pi and PPi small model systems demonstrate the key roles of the point charge at the Mg^{2+}-ion position, +1 charge or no ion leads to significantly increased barrier heights, despite the identical geometries used (Figures S29–S32; Table S5). The NBO-derived charge separation (Figures S30 and S31) does not reproduce the monotonic trend observed with ESP charges or obtained from the full electronic density (Figure S32), indicating a lack of robustness in capturing the electrostatic response to increasing point charge. Similarly, the Wiberg bond orders do not exhibit consistent or physically interpretable trends across the 0 to +2 charge range, limiting their reliability as descriptors of the evolving electronic structure (Table S5).

For the PPi system, frozen electrostatic contributions are negligible, while the polarized interactions are very large, thus the catalytic effect is dominated by electronic polarization. On the other hand, for the Pi system, the static field already contributes significantly, but polarization further enhances stabilization. In this case, both static electrostatics and polarization contribute (Table S5; Figure S32).

QM/MM system results

QM/MM activation barriers (Figures S33 and S34) were computed for dUTPase, RNase H, and Ras using consistent reactant-state and transition-state structures across ion substitutions (Mg^{2+}, Na^{+}, K^{+}, and no ion). For Ras, this corresponds to the rate-limiting step. Barriers were evaluated both from QM/MM total energies and from QM-only single-point calculations on the QM region. In all three systems, replacing Mg^{2+} by Na^{+}, K^{+}, or removing the ion altogether produces substantial changes in the computed barriers, with the strongest effects observed in dUTPase and RNase H and a pronounced effect for Ras in the absence of the ion. The only exception is Ras in the presence of K^{+}, which presents the only anomaly in the energy trends. We note here that at the higher-level using ωB97M-V/def2-TZVP range-separated hybrid meta-GGA, the anomalous Ras/K^{+} behaviour is reduced but not removed: the K^{+}-substituted system gives barriers of 20.40 kcal/mol for TS1 and 16.66 kcal/mol for TS2, so the overall barrier remains only about 1 kcal/mol below the corresponding Mg^{2+} value (20.92–21.38 kcal/mol), and is therefore still considered anomalous relative to the otherwise consistent catalytic trend.

Table S6. *QM/MM total energies (kcal mol⁻¹) for the reactant state (RS) and transition state (TS) of dUTPase, RNase H, and Ras with Mg^{2+} or without divalent cation (no Mg^{2+}). Energies are QM/MM total energies from B3LYP/6-31+G* and ωB97M-V/def2-TZVP single-point calculations. Activation barriers correspond to TS–RS differences.*

System	Cation	State	B3LYP/6-31+G*	ωB97M-V/def2-TZVP
dUTPase	Mg^{2+}	RS	-3098717.3399	-3098882.6165
dUTPase	Mg^{2+}	TS	-3098699.4664	-3098863.0378
dUTPase	no Mg^{2+}	RS	-2972924.0999	-2973082.9379
dUTPase	no Mg^{2+}	TS	-2972885.0911	-2973041.1724
RNase H	Mg^{2+}	RS	-2634234.3289	-2634351.5013
RNase H	Mg^{2+}	TS	-2634210.5313	-2634324.9055
RNase H	no Mg^{2+}	RS	-2508663.1030	-2508780.2753
RNase H	no Mg^{2+}	TS	-2508616.3856	-2508730.6171
Ras	Mg^{2+}	RS	-2222718.3914	-2222814.0607
Ras	Mg^{2+}	TS	-2222696.9826	-2222795.3697
Ras	no Mg^{2+}	RS	-2096947.1767	-2097042.0741
Ras	no Mg^{2+}	TS	-2096910.7726	-2097007.9688

Table S7. Activation barriers (kcal mol^{-1}) from QM/MM and QM calculations.

System	Ion	QM/MM total barrier	Q-Chem QM barrier	Gaussian QM barrier
dUTPase	Mg	17.873	22.279	22.797
dUTPase	Na	29.739	34.145	34.707
dUTPase	K	31.996	36.401	40.760
dUTPase	no ion	39.009	43.414	42.244
RNase H	Mg	23.798	23.921	23.935
RNase H	Na	34.334	34.457	34.550
RNase H	K	33.027	33.150	31.549
RNase H	no ion	46.717	46.841	46.849
Ras	Mg	21.409	24.098	24.216
Ras	Na	26.922	29.612	29.582
Ras	K	16.773	19.463	18.928
Ras	no ion	36.404	39.093	39.054

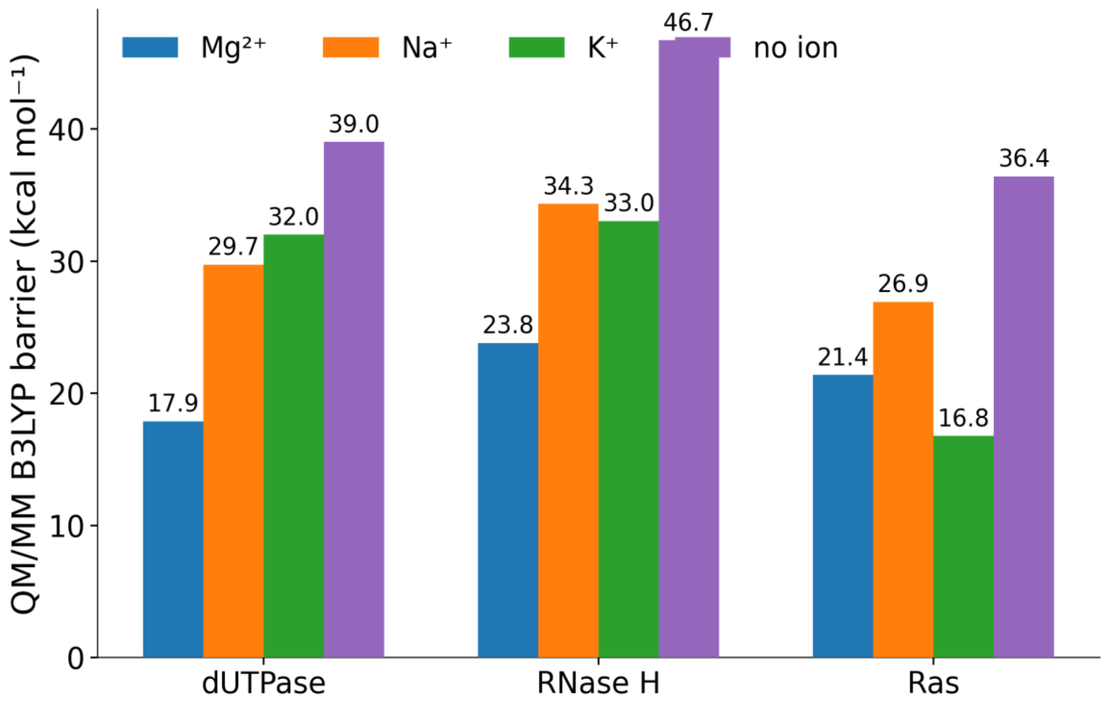

Figure S33: *QM/MM activation barriers for dUTPase, RNase H, and Ras across ion substitutions (Mg^{2+}, Na^{+}, K^{+}, no ion). Bars show absolute barrier heights (kcal mol^{-1}) from QM/MM total energies.*

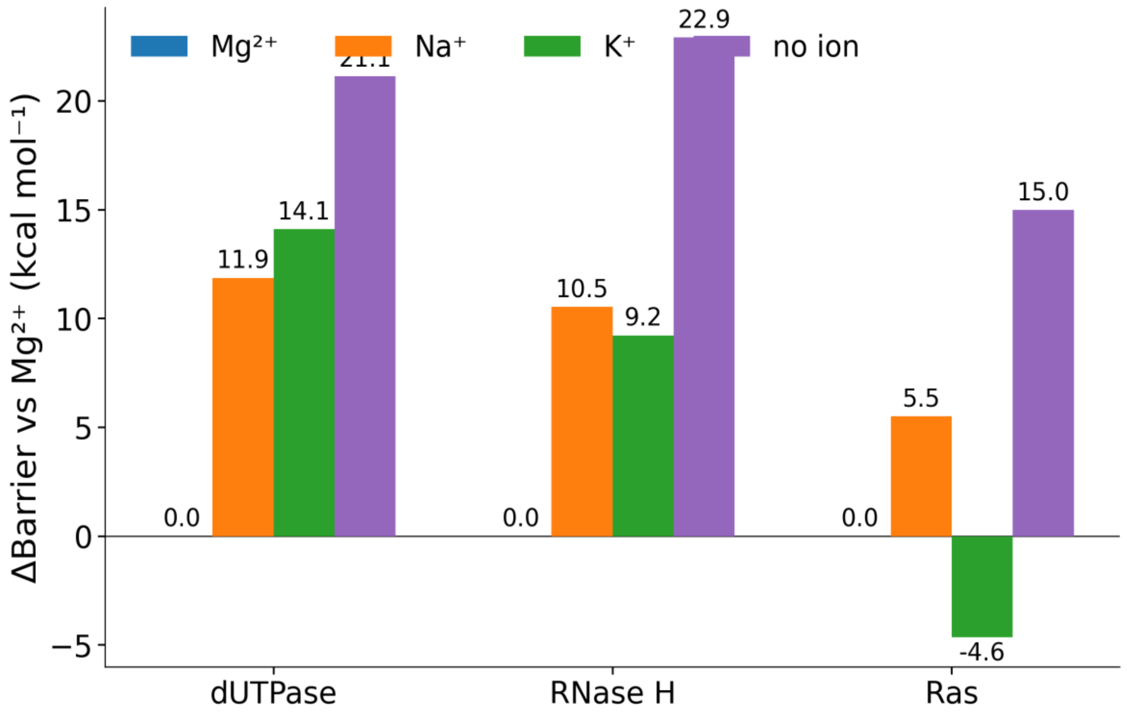

Figure S34: *QM/MM activation barriers relative to Mg^{2+} for each enzyme system. Positive values indicate higher barriers relative to the Mg^{2+} reference.*

Table S8. QM/MM activation barriers (kcal mol^{-1}) for the three phosphate-catalytic enzymes, computed with and without the catalytic Mg^{2+} ion. Barriers are shown for two QM levels of theory: B3LYP/6-31+G* and ωB97M-V/def2-TZVP.

System	Ion	QM/MM barrier B3LYP/6-31+G*	QM/MM barrier ωB97M-V/def2-TZVP
**Ras**	Mg	21.4	18.7
**Ras**	no Mg	36.4	34.1
**dUTPase**	Mg	17.9	19.6
**dUTPase**	no Mg	39.0	41.8
**RNase H**	Mg	23.8	26.6
**RNase H**	no Mg	46.7	49.7

To quantify how ion substitution changes the electronic structure along the reaction coordinate, ESP-based atomic charges were used to compute fragment charges and derived descriptors. For each system, the phosphorus charge (qP) at the reaction center, leaving-group oxygen charge (qOlg), nucleophile charge (qNuc), total phosphate fragment charge (Qphos), and the leaving-group fragment charge (Qlg) were evaluated for both RS and TS. The local fragment charge difference was also defined as Qdiff(P−L) = Qphos − Qlg, where Qphos is the summed ESP charge of the reacting phosphate fragment and Qlg is the summed ESP charge of the leaving-group fragment. Thus, Qdiff(P−L) reports the charge imbalance between the phosphate side and the departing group (Tables S9–S11; Figure S35). As shown in Figure S35, the ion dependence of Qdiff(P−L) is system-specific. In dUTPase and RNase H, Qdiff(P−L) generally increases from RS to TS, consistent with growing charge separation as the phosphoryl-transfer coordinate develops. In contrast, Ras shows a different pattern: Mg^{2+} already supports substantial charge separation at the reactant state and exhibits little additional increase at the TS, whereas Na^{+}, K^{+}, and especially the ion-free state require progressively larger RS→TS changes. This suggests that in Ras, Mg^{2+} acts largely through stronger reactant-state electrostatic preorganization, while in dUTPase and RNase H a larger fraction of the polarization develops along the reaction coordinate itself.

Detailed definitions for these quantities:

Descriptor	Meaning	Units
qP	ESP charge on the reacting phosphorus atom	e
qOlg	ESP charge on the specific breaking-bond / leaving-group oxygen atom	e
qNuc	ESP charge on the nucleophilic/attacking water oxygen atom only	e
q(H)	ESP charge on the proton transferred from the nucleophilic water to the acceptor base	e

Descriptor	Meaning	Units
q(OH)	Sum of ESP charges on the remaining OH fragment (O_{nu}^{c} + H_{stays}) after proton transfer	e
q(Acc)	Sum of ESP charges on the proton-acceptor fragment (His imidazole for RNase H; equivalent base for dUTPase and Ras)	e
Qphos	Sum of ESP charges over the reacting phosphate fragment	e
Qlg	Sum of ESP charges over the leaving-group fragment	e
Qdiff (P−L)	Qphos − Qlg	e
Dipole (fragment)	Effective fragment dipole, approximately abs(Qlg) × rSep × 4.8	D
Total dipole magnitude	Gaussian QM-region dipole magnitude, separate from the fragment dipole above	D

Atom Definitions

These are the atom indices for the Mg/Na/K calculations. For no ion calculations, we apply a −1 index shift because one QM atom was removed.

Enzyme	P	Olg	Phosphate	Q_{lg}	nucleophilic H_2O	O_{nuc}	Transfer H	OH fragment	Acceptor
dUTPase	22	25	21 22 23 24	25 26 27 28 29 30 31 32 33	66 67 68	66	67	66 68	14 15 16
RNase H	63	62	63 64 65 66	62	91 92 93	91	93	91 92	33 34 35 36 37 38 39 40
Ras	88	87	88 89 90 91	76 77 78 79 80 81 82 83 84 85 86 87 92	99 100 101	99	100	99 101	48 49 51 52 53

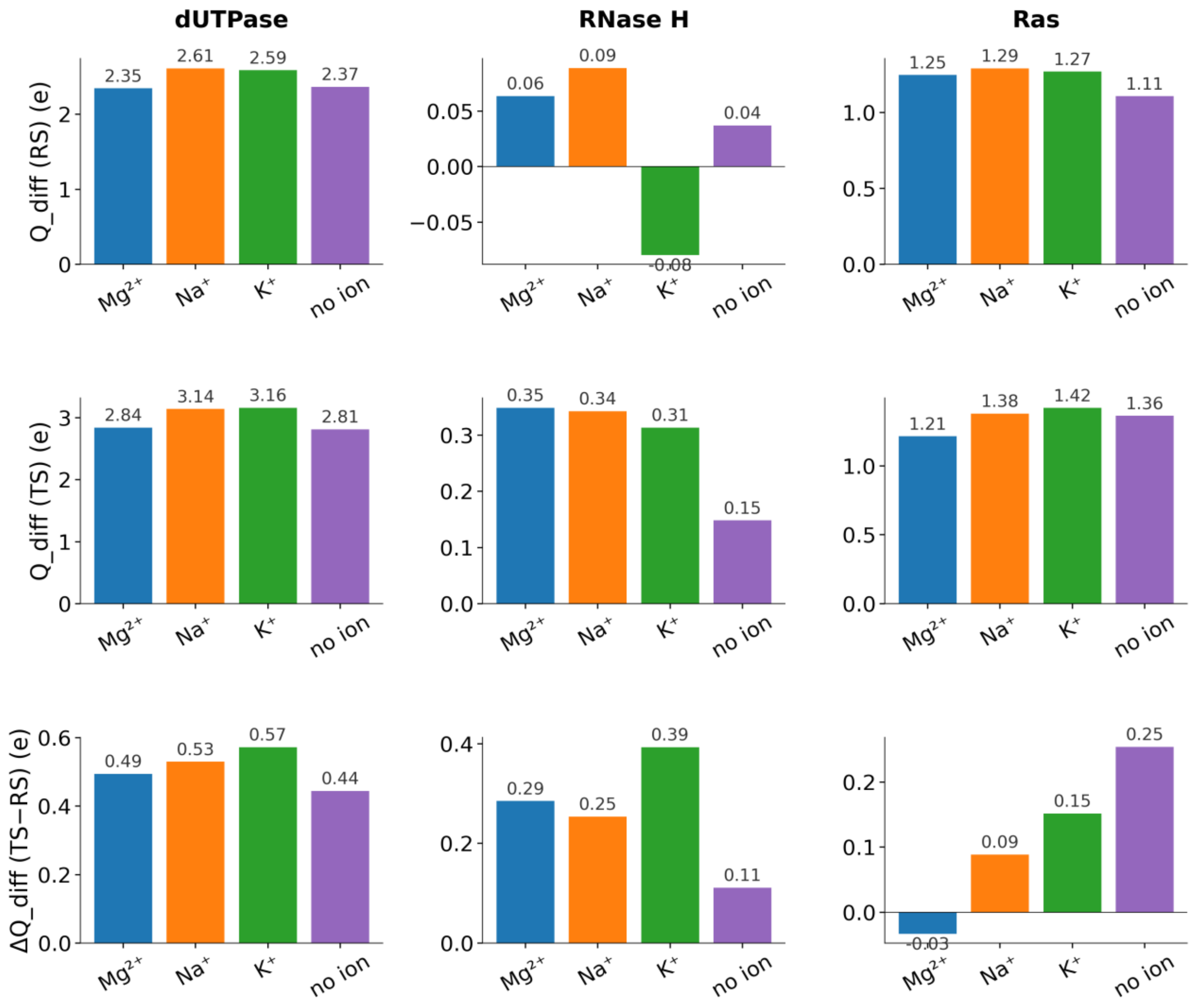

Figure S35: *ESP fragment charge-imbalance descriptor, Qdiff(P−L) = Qphos − Qlg, for dUTPase, RNase H, and Ras. Top row: reactant state (RS); middle row: transition state (TS); bottom row: ΔQdiff = Qdiff(TS) − Qdiff(RS). Qphos is the summed ESP charge of the reacting phosphate group (gamma-P + three non-bridging oxygens) and Qlg is the summed ESP charge of the leaving-group fragment. In dUTPase and RNase H, ΔQdiff is positive for all ions, indicating that the charge imbalance between the phosphate and leaving group increases at the TS. For Ras, Mg^{2+} uniquely shows near-zero ΔQdiff (−0.03 e), while monovalent ions and the ion-free case exhibit larger increases (Na: +0.09, K: +0.15, no ion: +0.25 e), suggesting that Mg^{2+} specifically stabilises the developing charge on the leaving group relative to the transferred phosphate.*

Table S9. ESP-based charges and properties for dUTPase.

Ion / State	qP	qOlg	qNuc	Qphos	Qlg	Qdiff (P–L)	Dipole (D)
Mg (RS)	1.4012	-0.5768	-0.7873	-0.7399	-3.0869	2.3470	39.6057
Mg (TS)	1.4839	-0.7470	-0.9381	-0.5413	-3.3828	2.8415	50.8073

Ion / State	qP	qOlg	qNuc	Qphos	Qlg	Qdiff (P–L)	Dipole (D)
Δ(TS–RS)	0.0826	-0.1703	-0.1508	0.1986	-0.2959	0.4945	11.2016
Na (RS)	1.7056	-0.6934	-0.8320	-0.9409	-3.5531	2.6122	43.4145
Na (TS)	1.7292	-0.8352	-1.0229	-0.6811	-3.8231	3.1420	54.4059
Δ(TS–RS)	0.0236	-0.1418	-0.1909	0.2599	-0.2700	0.5298	10.9914
K (RS)	1.6033	-0.6496	-0.8183	-1.0153	-3.6035	2.5881	43.1638
K (TS)	1.6973	-0.7975	-1.0242	-0.7402	-3.9006	3.1604	54.1025
Δ(TS–RS)	0.0940	-0.1479	-0.2058	0.2751	-0.2971	0.5722	10.9387
No ion (RS)	1.5564	-0.5969	-0.6548	-0.3560	-2.7237	2.3676	51.9502
No ion (TS)	1.8451	-0.7398	-1.0172	-0.2997	-3.1118	2.8121	61.9940
Δ(TS–RS)	0.2887	-0.1429	-0.3624	0.0563	-0.3881	0.4444	10.0438

Table S10. ESP-based charges and properties for RNase H.

Ion / State	qP	qOlg	qNuc	Qphos	Qlg	Qdiff (P–L)	Dipole (D)
Mg (RS)	1.9785	-0.8403	-0.4073	-0.7767	-0.8403	0.0636	30.5411
Mg (TS)	1.8962	-1.0614	-0.3694	-0.7128	-1.0614	0.3486	26.4387
Δ (TS-RS)	-0.0823	-0.2211	0.0379	0.0639	-0.2211	0.2851	-4.1024
Na (RS)	2.3277	-0.9392	-0.5425	-0.8505	-0.9392	0.0888	35.7125
Na (TS)	2.1581	-1.1162	-0.6073	-0.7735	-1.1162	0.3427	32.8194
Δ (TS-RS)	-0.1696	-0.1770	-0.0648	0.0770	-0.1770	0.2539	-2.8931
K (RS)	2.1328	-0.8815	-0.1852	-0.9610	-0.8815	-0.0795	35.4293
K (TS)	2.1043	-1.1325	-0.5885	-0.8189	-1.1325	0.3136	32.5559

Ion / State	qP	qOlg	qNuc	Qphos	Qlg	Qdiff (P–L)	Dipole (D)
Δ (TS-RS)	-0.0285	-0.2510	-0.4034	0.1420	-0.2510	0.3931	-2.8734
no ion (RS)	2.2602	-0.6940	-0.4788	-0.6569	-0.6940	0.0371	43.1322
no ion (TS)	2.1496	-0.7015	-0.9237	-0.5533	-0.7015	0.1482	41.0185
Δ (TS-RS)	-0.1106	-0.0075	-0.4449	0.1036	-0.0075	0.1111	-2.1137

Table S11. ESP-based charges and properties for Ras.

Ion / State	qP	qOlg	qNuc	Qphos	Qlg	Qdiff (P–L)	Dipole (D)
Mg (RS)	1.3428	-0.3866	-1.1393	-0.7494	-1.9962	1.2468	16.1675
Mg (TS)	1.3359	-0.4450	-0.7333	-0.9926	-2.2065	1.2139	26.1189
Δ(TS–RS)	-0.0069	-0.0584	0.4060	-0.2432	-0.2103	-0.0329	9.9514
Na (RS)	1.3802	-0.4234	-1.1906	-0.9150	-2.2049	1.2899	15.9386
Na (TS)	1.3718	-0.5241	-0.7826	-1.0740	-2.4525	1.3785	27.4703
Δ(TS–RS)	-0.0084	-0.1006	0.4079	-0.1589	-0.2475	0.0886	11.5317
K (RS)	1.2966	-0.3836	-1.1921	-0.9752	-2.2442	1.2691	15.9314
K (TS)	1.3577	-0.5380	-0.7897	-1.0828	-2.5035	1.4207	27.3409
Δ(TS–RS)	0.0610	-0.1544	0.4024	-0.1076	-0.2593	0.1517	11.4095
No ion (RS)	1.0247	-0.1768	-1.2157	-0.9047	-2.0131	1.1083	20.2594

Ion / State	qP	qOlg	qNuc	Qphos	Qlg	Qdiff (P–L)	Dipole (D)
No ion (TS)	1.3636	-0.3864	-0.8334	-0.9906	-2.3532	1.3626	31.1211
Δ(TS–RS)	0.3390	-0.2097	0.3823	-0.0858	-0.3401	0.2543	10.8617

The geometric evolution of the electronic polarization was also quantified using dipole-orientation metrics (Table S12). The Angle value for each state is the angle between the total QM dipole vector and the P → O_{lg} bond axis; Rotation is the net change in this angle from RS to TS. In dUTPase, the dipole is consistently anti-aligned with the P → O_{lg} axis (angles 134–157° across all ions), indicating polarization directed away from the leaving group. Rotation is small and uniform (5–7°), showing that the direction of polarization changes little as the reaction proceeds. In RNase H, the dipole is oriented far closer to perpendicular (angles 58–95°), reflecting the different dual-metal coordination geometry. Rotation is larger (11–15°) and relatively consistent across metal types, suggesting dipole reorientation is controlled primarily by the active-site scaffold. In Ras, the dipole is broadly anti-aligned (138–166°), similar to dUTPase. Notably, Mg^{2+} produces the largest rotation (22°), while Na^+ and K^+ show very small reorientation (5°); no ion gives intermediate rotation (20°), the only system where metal identity substantially changes dipole reorganization direction, consistent with Mg^{2+}-s unique catalytic competence in Ras. Overall, dUTPase and Ras share a broadly anti-aligned polarization pattern, whereas RNase H exhibits a qualitatively different orientation reflecting its distinct active-site geometry.

Table S12. Dipole-orientation changes along the reaction coordinate. For each system, the listed Angle values are the angles between the total dipole vector and the P → O_{lg} bond vector in the reactant state and transition state, respectively, and Rotation is the angle between the RS and TS dipole vectors.

Metal state	dUTPase Angle	dUTPase Rotation	RNase H Angle	RNase H Rotation	Ras Angle	Ras Rotation
Mg^{2+}	153.1 → 157.8	5.1	58.0 → 73.2	15.3	151.9 → 166.6	22.0
Na^+	145.1 → 150.8	5.7	72.3 → 85.9	13.6	165.2 → 155.9	5.3
K^+	145.9 → 151.3	5.5	71.8 → 85.4	13.6	165.5 → 156.0	5.2
no ion	134.0 → 140.8	6.8	83.4 → 94.8	11.4	138.4 → 146.8	19.8

The total dipole magnitudes and projected dipole components were also monitored along the reaction coordinate (Tables S13-S14). The absolute dipole magnitudes are strongly ion-dependent, especially in dUTPase and RNase H, but this partly reflects the identity and formal charge of the ion-containing QM model rather than a unique catalytic descriptor. For that reason, total dipoles are treated mainly as polarization diagnostics and consistency checks. The projected dipole change along the leaving-group axis is much less sensitive to ion identity within a given system, indicating that ion substitution perturbs the overall polarization of the QM region more strongly than it changes the direction of charge flow along the reaction coordinate.

Table S13. Total dipole magnitudes, |μ| and projected dipole components, μ_{proj} (in Debye, D).

System	Metal state	|μ| RS → TS	Δ|μ|	μ_{proj} RS → TS	$\Delta\mu_{proj}$
dUTPase	Mg	39.6 -> 50.8	11.2	-35.3 -> -47.1	-11.7
dUTPase	Na	43.4 -> 54.4	11.0	-35.6 -> -47.5	-11.9
dUTPase	K	43.2 -> 54.1	10.9	-35.7 -> -47.5	-11.7
dUTPase	no ion	52.0 -> 62.0	10.0	-36.1 -> -48.0	-11.9
RNase H	Mg	30.5 -> 26.4	-4.1	16.2 -> 7.6	-8.6
RNase H	Na	35.7 -> 32.8	-2.9	10.9 -> 2.4	-8.5
RNase H	K	35.4 -> 32.6	-2.9	11.1 -> 2.6	-8.5
RNase H	no ion	43.1 -> 41.0	-2.1	4.9 -> -3.4	-8.4
Ras	Mg	16.2 -> 26.1	10.0	-14.3 -> -25.0	-10.8
Ras	Na	15.9 -> 27.5	11.5	-15.6 -> -26.0	-10.4
Ras	K	15.9 -> 27.3	11.4	-15.6 -> -25.9	-10.3
Ras	no ion	20.3 -> 31.1	10.9	-16.8 -> -26.9	-10.0

For direct comparison with the dipole-projection analysis, the RS and TS projected dipoles along the leaving-group axis are listed separately below (Table S14). In this analysis, the Gaussian QM-region dipole vector was projected onto the P → O_{LG} unit vector, corresponding to the bond-breaking direction. The projected value therefore measures the component of the dipole aligned with the leaving-group coordinate. While the total dipole magnitude varies substantially with ion identity, the TS - RS change in this projected component is comparatively conserved within each system.

Table S14. Projected dipole values, μ_{proj} from the reaction-axis analysis (in Debye, D).

System	Ion	μ_{proj} (RS)	μ_{proj} (TS)	$\Delta\mu_{proj}$
dUTPase	Mg	-35.3269	-47.0554	-11.7286
dUTPase	Na	-35.6243	-47.4815	-11.8572
dUTPase	K	-35.7398	-47.4582	-11.7184
dUTPase	no ion	-36.1100	-48.0444	-11.9344
RNase H	Mg	16.1954	7.6309	-8.5645

System	Ion	μ_{proj} (RS)	μ_{proj} (TS)	$\Delta\mu_{proj}$
RNase H	Na	10.8507	2.3503	-8.5004
RNase H	K	11.0665	2.5934	-8.4731
RNase H	no ion	4.9473	-3.4095	-8.3568
Ras	Mg	-14.2827	-25.0405	-10.7578
Ras	Na	-15.5620	-25.9620	-10.4000
Ras	K	-15.5852	-25.8843	-10.2991
Ras	no ion	-16.8446	-26.8509	-10.0063

We also evaluated the full dipole-change vector magnitudes (Table S15), which can be used as a consistency check that the projected change remains smaller than the full vector change. These values are relatively ion-invariant within each system (Ras spans approximately 11.1-12.5 D across ions) and therefore did not emerge as useful discriminators of barrier height. Any apparent correlation involving the absolute total dipole should therefore be interpreted cautiously, because it can largely report metal identity and the net ion-dependent polarization of the QM region.

Table S15. Magnitude of the dipole-change vector, |Δμ| (D).

System	Mg	Na	K	No ion
dUTPase TS−RS	11.8973	12.0128	11.8856	12.0816
RNase H TS−RS	8.5891	8.6028	8.5608	8.6408
Ras TS−RS	12.5044	11.8371	11.7528	11.1165

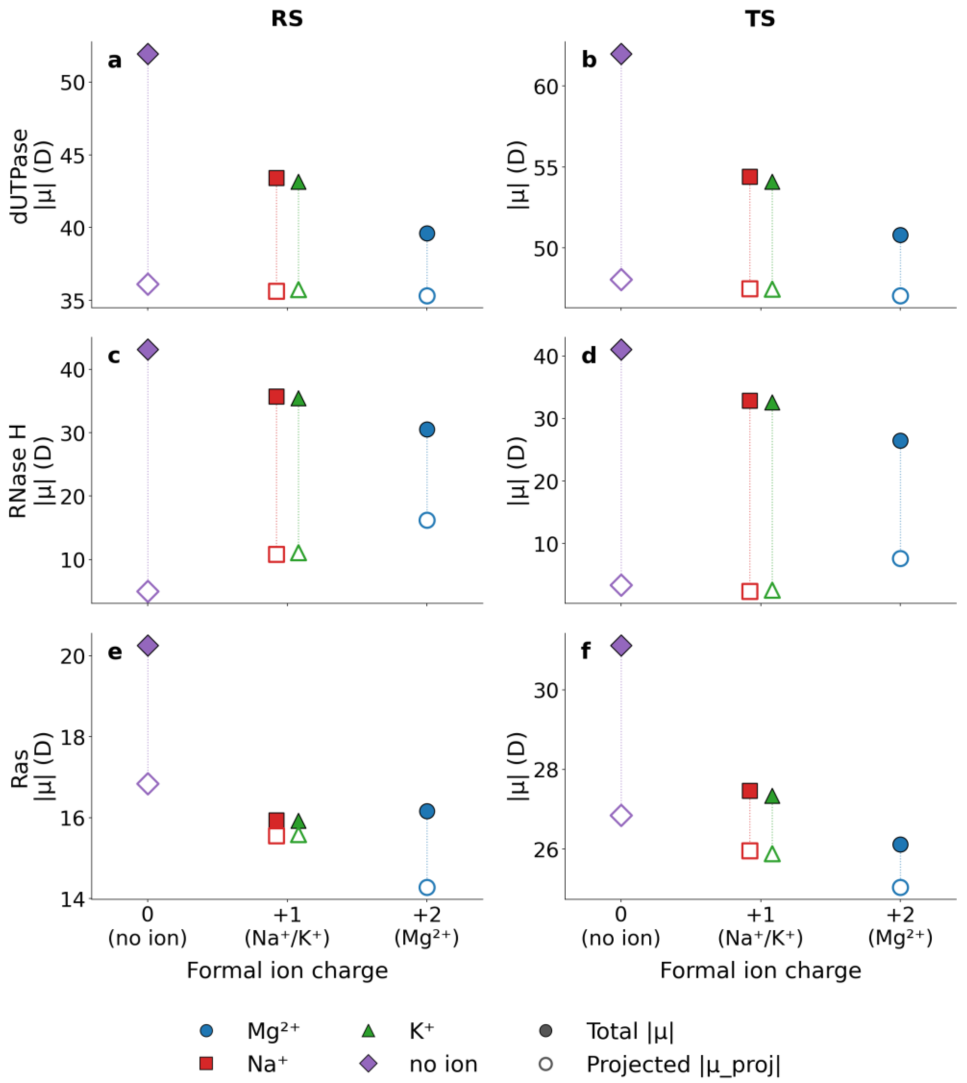

Figure S36: *Total QM-region dipole magnitude |μ| (filled markers) and projected dipole component $|\mu_{xaj}|$ along the P→O_{lg} axis (open markers) as a function of formal ion charge for dUTPase (a, b), RNase H (c, d), and Ras (e, f), at the reactant state (RS; left column) and transition state (TS; right column). In dUTPase and Ras, the projected component is nearly invariant across all ions at both the RS and TS despite large changes in total |μ|, indicating that the directional charge flow along the reaction coordinate does not simply track ion charge. In RNase H, the projected component decreases with increasing charge, reflecting the distinct dual-metal coordination geometry. The anomalous total |μ| ordering in Ras RS (no ion > Mg^{2+} ≈ Na^+/K^+) arises because Mg^{2+} organizes the QM-region polarization along a single axis, reducing the net vector magnitude relative to the more diffuse distributions found with monovalent or absent ions.*

The contrast between total and projected dipole magnitudes is illustrated in Figure S36, which plots both quantities as a function of formal ion charge at the RS and TS. In dUTPase, the total |μ| decreases markedly with increasing charge (no ion > Na^+/K^+ > Mg^{2+}), yet the projected component $|\mu_{xaj}|$ remains nearly constant across all ions at both the RS and TS, demonstrating that the directional charge flow along the reaction coordinate is insensitive to metal identity. In RNase H, an opposite trend is observed for the projected component: $|\mu_{xaj}|$ decreases as charge increases, reflecting the qualitatively different dual-metal active-site geometry in which the metal ion lies on the opposite side of the phosphate relative to

dUTPase and Ras. Notably, the Ras RS total dipole does not follow the expected charge ordering; the no-ion case shows the largest total |μ|, while Mg^{2+} gives a value only marginally above Na^{+}/K^{+}. This reflects the tight, approximately octahedral coordination of Mg^{2+} in Ras, which aligns the QM-region polarization predominantly along a single axis and thereby reduces the net vector magnitude compared with the more diffuse charge distributions observed without an ion.

Wiberg bond orders for both the breaking P–O_{LG} bond and the forming P–O_{nuc} bond were monitored to assess how closely progression along the phosphoryl-transfer coordinate tracks the catalytic trends (Table S16, Figure S37). These bond-order changes capture the ion dependence well. The increase in the forming bond order, ΔW(P–O_{nuc}) = W(TS) − W(RS), correlates strongly and inversely with the QM/MM barrier in dUTPase and RNase H (r = -0.99 and -0.98, respectively), and remains clearly informative in Ras (r = -0.68). Conversely, the decrease in the breaking bond order, ΔW(P–O_{LG}), shows strong positive correlation with the barrier (r = +0.95, +0.97, and +0.76 for dUTPase, RNase H, and Ras, respectively). In all three systems, Mg^{2+} gives the largest increase in P–O_{nuc} bond order and the largest decrease in P–O_{LG} bond order, whereas the ion-free state gives the smallest changes, with Na^{+} and K^{+} lying in between. These results show that Wiberg bond orders do reflect the catalytic ordering well when considering the change from RS to TS, particularly for dUTPase and RNase H, and indicate that Mg^{2+} promotes a more advanced phosphoryl-transfer coordinate at the transition state. The weaker correlation in Ras likely reflects, at least in part, the anomalously low K^{+} barrier, which behaves as an outlier relative to the otherwise monotonic ion dependence.

Table S16. Wiberg bond orders for the breaking P–O_{LG} bond and the forming P–O_{nuc} bond.

System	State	Mg^{2+}		Na^{+}		K^{+}		no ion	
		breaking	forming	breaking	forming	breaking	forming	breaking	forming
dUTPase	RS	0.5569	0.0069	0.5466	0.0064	0.5479	0.0061	0.5355	0.0060
	TS	0.1053	0.3861	0.1006	0.3603	0.1005	0.3567	0.0949	0.3355
RNase H	RS	0.5459	0.0681	0.5603	0.0571	0.5571	0.0565	0.5783	0.0482
	TS	0.2556	0.3462	0.2817	0.3174	0.2771	0.3142	0.3062	0.2919
Ras	RS	0.4798	0.0014	0.4701	0.0012	0.4708	0.0012	0.4602	0.0012
	TS	0.0028	0.6357	0.0027	0.6105	0.0027	0.6087	0.0028	0.5868

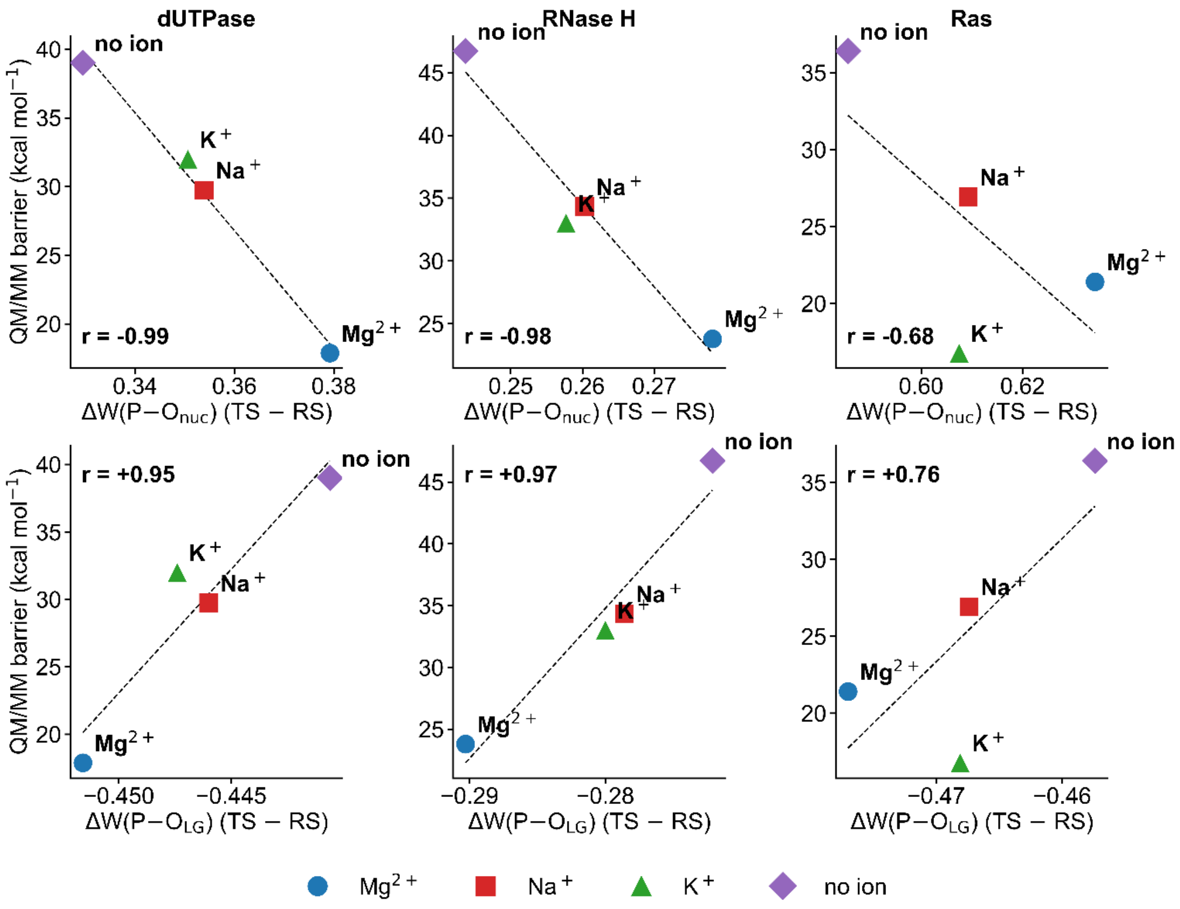

***Figure S37**: Changes in Wiberg bond orders along the reaction coordinate plotted against QM/MM activation barriers for dUTPase (left), RNase H (centre), and Ras (right). Top row: change in the forming bond order, ΔW(P–O_{nuc}) = W(TS) – W(RS). Bottom row: change in the breaking bond order, ΔW(P–O_{LG}) = W(TS) – W(RS). Symbols denote Mg^{2+} (blue circles), Na^{+} (red squares), K^{+} (green triangles), and the ion-free state (purple diamonds). Dashed lines show linear fits, and r values are Pearson correlation coefficients.*

We then further added candidate metal-centered and local-field descriptors (Figure S38; Tables S17–S20). The LP(O)→M E2 energy quantifies NBO donation from coordinating oxygen lone pairs to metal acceptor orbitals at the TS, calculated by summing the Gaussian/NBO second-order perturbation stabilization energies, \`E(2)\`, for all oxygen lone-pair donor interactions into lone-pair antibonding acceptor orbitals, \`LP*\`, on the catalytic metal ion. For dUTPase, this ranges from 148 kcal mol^{-1} for Mg^{2+} down to 92 for Na^{+} and 67 kcal mol^{-1} for K^{+} (Table S17), directly reflecting the greater Lewis acidity of Mg^{2+} and its ability to polarize the phosphate oxygen lone pairs. Using the site-specific definition plotted in Figure S38, this descriptor correlates strongly and negatively with the barrier for dUTPase and RNase H (r = -0.98 in both systems) and remains negative, although weaker, for Ras (r = -0.65), where the anomalously low K+ barrier weakens the four-point correlation (Table S22). The ordering cannot be explained by the formal 2:1 charge ratio between Mg^{2+} and the monovalent ions alone: the Mg^{2+}/Na^{+} ratio is approximately 1.6–1.7 across the three systems, while Na^{+} and K^{+} differ by approximately 1.3–1.4 despite having the same formal charge (Tables S17–S19). Thus, the LP(O)→M descriptor reflects not only charge density but also ion size, coordination geometry, and site-specific donor–acceptor stabilization. This is consistent with the finding that the projected component of the QM dipole change along the reaction coordinate is nearly invariant across all metal ions (Figure S36; Tables S13–S14), which likewise argues against a simple charge-scaling

interpretation of the metal's electronic influence. The LP(O)→P* E2 energy measures nucleophilic oxygen donation into a hypervalent acceptor orbital on phosphorus at the TS and is a particularly powerful predictor in dUTPase (r = −0.97) and RNase H (r = −0.99); it is absent for Ras, where a fully hypervalent P intermediate does not form at the second TS. The leaving-group oxygen is additionally stabilised by complementary NBO donor–acceptor channels whose dominant contributors differ across the three enzymes (Table S20). In dUTPase, two backbone NHs of the γ-phosphate pocket together donate ~43 kcal mol^{-1} at the Mg^{2+} TS. In RNase H, the dominant channel is direct LP(O 3′)→LP*(site-B Mg) coordination (~21 kcal mol^{-1}), with no backbone-amide donor partners. In Ras, where the LP(O)→P* channel is unavailable, the leaving group side is dominated by the Gly60 backbone NH (41.1 kcal mol^{-1}) with further contributions from the Gln61 side-chain NH_2 and the Walker-A Lys16 NH_3^+; this network is large in all three metal-bound states (~104–109 kcal mol^{-1}) but collapses in the no-ion case.

Table S17. ESP charges and NBO interaction energies for the dUTPase QM/MM models. ESP charges (e) of the attacking-water transferring proton (H), OH fragment (OH), and acceptor oxygen fragment (Acc) at the reactant state (RS) and transition state (TS) geometries. q(M, ESP) RS is the ESP charge of the catalytic metal ion from the RS geometry. E2[LP(O)→ion] is the total NBO second-order perturbation energy (kcal/mol) from coordinating oxygen lone pairs to metal ion acceptor orbitals at the TS. E2[LP(O)→P*] is the maximum NBO E2 for lone-pair donation from the nucleophilic oxygen to the phosphorus LP* at the TS. Φ(P) is the ESP electrostatic potential at phosphorus (au) at RS and TS. n/a: no metal ion present or absent above threshold.

Ion	ESP Charges (e)						q(M, ESP) RS	NBO E2 (kcal/mol)			Φ(P) ESP (au)	
Ion	q(H) RS	q(H) TS	q(OH) RS	q(OH) TS	q(Acc) RS	q(Acc) TS		LP(O)→ion	LP(O)→P*	n(Onuc)→σ*(P–Olg)	RS	TS
Mg	0.451	0.509	-0.524	-0.367	-0.513	-0.476	1.957	148.0	132.05	n.a.	0.0750	0.0426
Na	0.437	0.537	-0.483	-0.433	-0.462	-0.446	2.130	92.2	126.55	n.a.	0.0202	-0.0111
K	0.437	0.536	-0.485	-0.427	-0.470	-0.436	2.494	66.5	125.98	n.a.	0.0363	-0.0023
no ion	0.310	0.423	-0.605	-0.437	-0.340	-0.332	n/a	0.0	118.86	n.a.	-0.1916	-0.2119

Table S18. ESP charges and NBO interaction energies for the RNase H QM/MM models. Column definitions as in Table S17. RNase H without divalent cation: LP*(P) does not form at the TS, so the nucleophile oxygen donates into σ*(P–Olg) instead (E2 = 47.39 kcal mol^{-1}). This indicates a more S_N2-like geometry in which pentacoordinate phosphorus does not reach a fully hypervalent intermediate without Mg^{2+}. In all other cases, LP*(P) forms and n(Onuc)→σ*(P–Olg) is absent above threshold (n.a.). For LP(O)→ion, RNase H reports donation into the substituted/site-B metal only; the second Mg^{2+} retained in the QM region is not included in this descriptor.

Ion	ESP Charges (e)						q(M, ESP) RS	NBO E2 (kcal/mol)			Φ(P) ESP (au)	
Ion	q(H) RS	q(H) TS	q(OH) RS	q(OH) TS	q(Acc) RS	q(Acc) TS		LP(O)→ion	LP(O)→P*	n(Onuc)→σ*(P–Olg)	RS	TS
Mg	-0.253	-0.235	-0.061	0.018	0.354	0.540	1.594	117.5	109.84	n.a.	0.2604	0.2688
Na	-0.203	-0.172	-0.168	-0.125	0.331	0.540	1.286	69.4	101.79	n.a.	0.1904	0.1903
K	-0.337	-0.180	0.050	-0.095	0.332	0.508	1.580	51.8	101.58	n.a.	0.1883	0.2035
no ion	-0.232	0.003	-0.120	-0.291	0.326	0.500	n/a	0.0	0.0	47.39†	-0.0258	-0.0613

Table S19. ESP charges and NBO interaction energies for the Ras QM/MM models. Column definitions as in Table S17. The n(Olg)→σ*(P–Onuc) values at the TS are small (0.13–0.22 kcal mol^{-1} across all ions), consistent with a product-like TS in which the Olg–P bond is substantially broken and the residual Olg–P interaction is weak. LP*(P) does not form for Ras (n.a. in LP(O)→P* column), reflecting that Ras TS is further along the reaction coordinate than dUTPase or RNase H.

Ion	ESP Charges (e)						q(M, ESP) RS	NBO E2 (kcal/mol)			Φ(P) ESP (au)	
Ion	q(H) RS	q(H) TS	q(OH) RS	q(OH) TS	q(Acc) RS	q(Acc) TS		LP(O)→ion	LP(O)→P*	n(Olg)→σ*(P–Onuc)	RS	TS
Mg	0.636	0.517	-0.664	-0.345	-0.168	-0.197	1.249	142.6	n/a	0.13	0.2206	0.2501
Na	0.674	0.540	-0.721	-0.382	-0.170	-0.223	1.301	88.2	n/a	0.17	0.1546	0.1716
K	0.675	0.541	-0.722	-0.372	-0.165	-0.215	1.597	62.7	n/a	0.17	0.1653	0.1773
no ion	0.680	0.591	-0.745	-0.431	-0.197	-0.231	n/a	0.0	n/a	0.22	-0.0022	0.0173

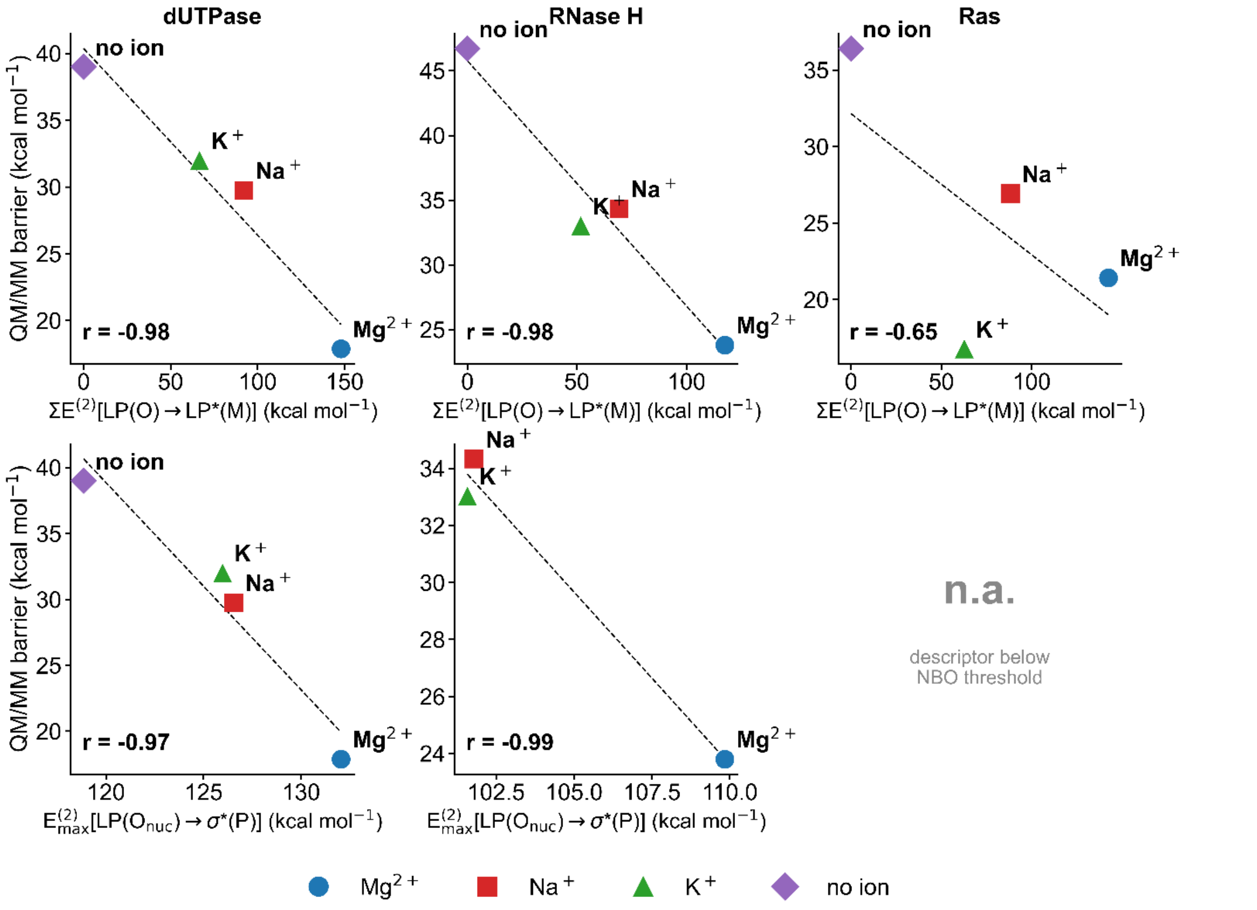

Figure S38: *NBO second-order perturbation theory E(2) descriptors plotted against QM/MM B3LYP activation barriers for dUTPase (left column), RNase H (middle column), and Ras (right column).* ***Top row:*** *total lone-pair donation into the metal acceptor, LP(O)→LP*(M) E2 donation energy at the TS (sum over all coordinating oxygens to the metal).* ***Bottom row:*** *maximum donation from the nucleophile-oxygen lone pair into a phosphorus antibonding orbital, LP(O_{nuc})→P* E2 at the TS; not shown for Ras as it is below printing threshold. Pearson's r against the activation barrier is annotated in each panel; dashed lines are linear regressions.*

Table S20. Leaving group-side NBO E^2 stabilisation channels at the TSs for the three enzymes. Each column lists one enzyme system; rows give, in order: the LG-side E^2 descriptor formula; the descriptor value at the Mg^{2+}, Na^+, K^+, and no-ion transition states (kcal mol^{-1}, summed over the three lone pairs of the donor oxygen); the Pearson correlation coefficient between the descriptor and the QM/MM activation barrier across the four ion conditions ($n = 4$); and the dominant donor → acceptor pairs at the Mg^{2+} TS. The descriptor differs by enzyme (Σ LP(O)→σ*(N–H) for dUTPase and Ras; LP(O)→LP*(metal) for RNase H), so absolute magnitudes are not directly comparable across rows. Top contributors are listed for the Mg^{2+} TS only, with E^2 summed over the three donor lone pairs. For RNase H, substrate-internal sugar σ*(C–C, C–H) hyperconjugation contributes a further ~28 kcal/mol at the Mg^{2+} TS but is intrinsic to the LG fragment and is not included in this column.

**Property**	**dUTPase**	**Ras TS2**	**RNase H**
LG-side E^2 descriptor	Σ LP(O_LG-side)<br>→ σ*(N–H)	Σ LP(O_LG-side)<br>→ σ*(N–H)	LP(O_LG)<br>→ LP*(site-B metal)
Mg^{2+} (kcal mol^{-1})	43.16	103.73	20.58
Na^+ (kcal mol^{-1})	45.95	108.57	13.25
K^+ (kcal mol^{-1})	45.29	108.67	10.59
no ion (kcal mol^{-1})	46.95	27.53	0.00
Pearson *r* vs barrier	+0.96	−0.87	−0.98
Top contributors at Mg^{2+} TS	O 33 → N 105–H 106 (22.82)<br>O 32 → N 108–H 109 (20.27)<br>both backbone NHs of γ-phosphate pocket	O 89 → N 51–H 52 Gly60 backbone NH (41.10)<br>O 91 → N 60–H 61 Gln61 NH_2_a (35.57)<br>O 82 → N 69–H 70 Lys16 NH_3^+ (19.71)	LP(O 62) → LP*(Mg-B)<br>substrate-internal sugar σ*(C–C, C–H) hyperconjugation, intrinsic to the LG fragment (~28 kcal/mol, not included here)

We also calculated the ESP electrostatic potential at phosphorus, Φ(P), evaluated as a Coulomb sum over the QM region using ESP-fitted atomic charges, with the reacting phosphate and the nucleophilic water excluded. Φ(P) provides a direct measure of how strongly the metal ion electrostatically activates the reaction center for leaving-group departure. Across all three enzyme systems, Φ(P) shows a strong

negative correlation with the QM/MM activation barrier (r ≈ -0.82 to -0.97; Tables S17–S19, S22), and this correlation is already present at the reactant state and remains essentially unchanged at the transition state (Figure S39). This behavior is the hallmark of electrostatic preorganization: through its pinching coordination geometry, the metal ion withdraws electron density from the phosphate and renders phosphorus more electrophilic before nucleophilic attack begins. Mg^{2+}, with its short metal–oxygen distances and high charge density, systematically produces the most activating Φ(P) and the lowest barriers, whereas Na^{+} and K^{+} are substantially less effective and the ion-free case gives the least activating potential and the highest barriers in every system. The ESP charge on the metal, q(M,ESP), was also monitored and is informative within each system, although it is not a universal predictor because the metal retains only a partial charge owing to covalent coordination. Together, these descriptors show that Mg^{2+} consistently occupies the low-barrier extreme of the electrostatic trends, confirming that its catalytic advantage is fundamentally electronic and reflects stronger preorganization of the phosphate charge distribution.

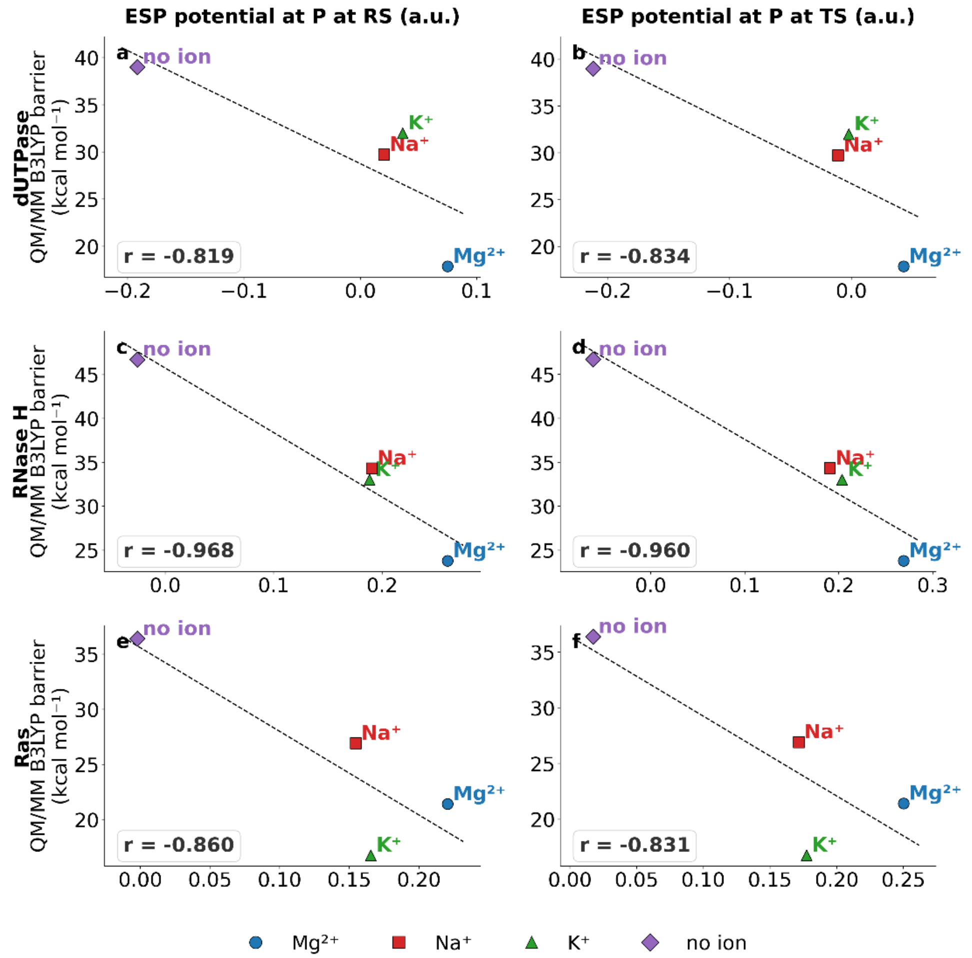

__Figure S39:__ Electrostatic potential at the phosphorus centre evaluated at the reactant state (RS, left column) and transition state (TS, right column), plotted against the QM/MM activation barrier for dUTPase, RNase H, and Ras. The potential was computed from ESP-fitted atomic charges, excluding the reacting atoms, and reflects how strongly the metal ion makes P electrophilic. Pearson correlation coefficients (r) are shown in each panel.

Finally, we examined the proton transfer from the nucleophilic water (Figure S40; Tables S17–S19). We decomposed the water into the transferred proton and the remaining OH fragment. This provides a more chemically direct proton-transfer descriptor. We found that the acceptor charge change Δq(Acc) that measures electron density gained by the base at the TS, and the Δq(H) − Δq(Acc) that reports the net charge asymmetry of the proton-transfer step, both correlate well with the barrier within individual systems (Table S22). The transferred-proton/acceptor ESP contrast is not a strict universal law, but it is the simplest raw charge-based quantity that remains consistently informative across the three enzyme systems. We observed the largest and most systematic electronic changes associated with proton-transfer-related charge redistribution, supporting a mechanistic description in which Mg^{2+} stabilizes a coupled proton-transfer/phosphoryl-transfer process rather than simply promoting phosphate cleavage.

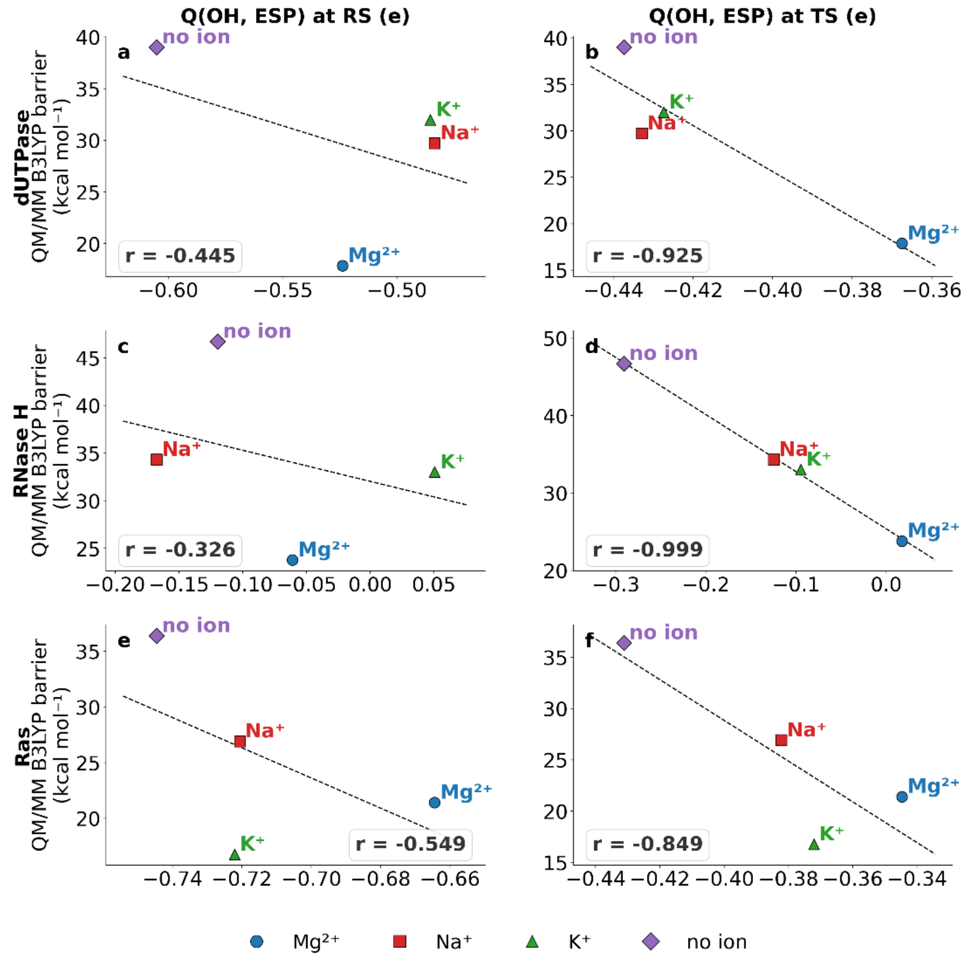

Figure S40: *Correlation of the OH fragment ESP charge at the reactant state (RS, left column) and transition state (TS, right column) with the QM/MM activation barrier for dUTPase, RNase H, and Ras. Each point represents one ion condition. The x-axis is the summed ESP-fitted charge of the attacking-water OH fragment. Pearson correlation coefficients (r) are shown in each panel.*

The contrasting behaviour of q(OH) at the reactant state versus the transition state is shown in Figure S40. At the RS, q(OH) shows no significant correlation with the barrier in any of the three enzyme systems, despite the formal charge of the metal ion differing by up to two units. This directly rules out static charge-induced polarization of the nucleophilic water as the determining factor: if the metal simply preorganized the nucleophile through its electrostatic field, the RS charge would already reflect the catalytic ranking.

The strong correlation observed at the TS (mean |r| = 0.92; Figure S40) instead points to a dynamical polarization effect. Crucially, all calculations within each enzyme system use the same protein structure — the MM region, active-site scaffold, and surrounding electrostatic environment are identical across the Mg^{2+}, Na^{+}, K^{+}, and no-ion series. The only variable is the identity of the metal ion. Therefore, the systematic differences in q(OH) at the TS cannot arise from structural differences in the protein environment; they must reflect the direct electronic influence of the ion on the charge redistribution as the O–P bond forms and the proton is transferred. The metal ion dynamically polarizes the reacting system at the transition state, modulating how electron density flows from the nucleophilic OH toward phosphorus during bond formation. This is a qualitatively different mechanism from static preorganization, and it explains why Mg^{2+} — with its greater Lewis acidity and tighter coordination — is uniquely effective across all three enzyme systems.

Together with the dynamical polarization evidence from q(OH) (Figure S40), these results support a two-component model for Mg^{2+} catalysis. First, the ion preorganizes the phosphate for P–O_{LG} cleavage through its electrostatic field, an effect that is already present at the reactant state and persists to the transition state. Second, at the TS the same ion dynamically polarizes the nucleophilic OH fragment, redistributing electron density from the attacking water into the forming P–O bond as the proton is simultaneously transferred to the acceptor. Na^{+} and K^{+} fail on both counts; in the Ras system, where only Mg^{2+} is catalytically competent, the gap in both ϕ(P) and q(OH) at the TS between Mg^{2+} and the monovalent ions is particularly pronounced.

Table S21. Pearson correlation coefficients (r) between selected descriptors and QM/MM B3LYP barrier.

Descriptor	r (dUTPase)	r (RNase H)	r (Ras)
n(Olg)→σ*(P-Onuc) [TS]	n.a.	n.a.	+0.76
LP(O)→M [TS]	-0.98	-0.98	-0.65
qP(ESP) [TS]	+0.98	+0.80	+0.45
Φ(P,ESP) [RS]	-0.82	-0.97	-0.86
ΔWiberg P-Olg	+0.95	+0.97	+0.76
LP(O)→P* [TS]	-0.97	-0.99	n.a.

Table S22: Pearson correlation coefficients (r) between the top 25 electronic descriptors (ranked by mean |r| averaged across the three enzyme systems) and the QM/MM B3LYP activation barrier, computed across all 12 ion/system combinations (All, n = 12) and separately for each enzyme (n = 4).

Descriptor	mean |r|	r (dUTPase)	r (RNase H)	r (Ras)
LP(O)→P* [TS]	+0.98	−0.97	−0.99	n.a.
LP(O)->P* [TS] (alternative extraction)	+0.94	−0.97	−0.90	n.a.
μ total [RS]	+0.92	+0.90	+1.00	+0.87
q(OH,ESP) [TS]	+0.92	−0.92	−1.00	−0.85
μ total [TS]	+0.92	+0.90	+1.00	+0.85
μ proj [TS]	+0.90	−0.95	−1.00	−0.75
Δμ proj	+0.90	−0.95	−1.00	−0.75
μ proj [RS]	+0.90	−0.97	−1.00	−0.72
ΔWiberg P–Olg	+0.89	+0.95	+0.97	+0.76
Φ(P,ESP) [RS]	+0.88	−0.82	−0.97	−0.86
Δq(H)−Δq(Acc) ESP	+0.88	+0.95	+0.79	+0.90
Φ(P,ESP) [TS]	+0.87	−0.83	−0.96	−0.83

Descriptor	mean |r|	r (dUTPase)	r (RNase H)	r (Ras)
LP(O)→M [TS]	+0.87	−0.98	−0.98	−0.65
q(H,ESP) [RS]	+0.78	−0.52	+0.99	+0.82
q(Acc,ESP) [TS]	+0.77	+0.87	−0.75	−0.70
n(Olg)→σ*(P-O_{nuc}) [TS]	+0.76	n.a.	n.a.	+0.76
qP(ESP) [TS]	+0.74	+0.98	+0.80	+0.45
q(M,ESP) [RS]	+0.74	+0.84	−0.63	−0.76
|Δμ|	+0.70	+0.69	+0.72	−0.69
Δq(Acc) ESP	+0.46	−0.79	−0.29	+0.30
q(OH,ESP) [RS]	+0.44	−0.45	−0.33	−0.55
Q_{lg}−qP ESP [TS]	+0.22	−0.26	+0.30	+0.11
Qdiff(P-L, ESP) [RS]	+0.22	−0.26	+0.30	+0.11

Overall, we interpreted the final correlations in Table S22 as a descriptor screen rather than as a set of independent mechanistic laws. Descriptors involving the absolute total dipole moment show sizable correlations, but these are partly expected because the total wave function dipole is strongly affected by the identity and formal charge of the substituted ion. Thus, total dipoles are useful as diagnostics of global polarization, but they are less chemically specific than the local charge-transfer and NBO descriptors. No single local descriptor is equally predictive for all three enzymes, consistent with the fact that dUTPase, RNase H, and Ras use different active-site geometries and different proton-transfer arrangements. Nevertheless, the descriptors that remain chemically informative point in the same direction: lower barriers are associated with stronger metal-assisted polarization of coordinating oxygens, larger nucleophile-to-phosphorus donor interactions where an LP*(P) acceptor is formed, and ESP charge redistribution along the proton-transfer coordinate. The no-ion cases often sit at the edge of these trends, as expected for high-energy structures with poorer electrostatic stabilization. Taken together, these correlations support a two-component mechanistic model for Mg^{2+} catalysis: (i) electrostatic preorganization of the phosphate for P–Olg cleavage through the ion's pinching coordination geometry, an effect already present at the reactant state and captured by the strongly correlated ESP potential at P (Figure S39); and (ii) dynamical polarization of the nucleophilic OH fragment at the transition state, evidenced by the TS-only correlation of q(OH) (Figure S40).

10. Phosphatase superfamilies

Phosphatases with αβγ coordination on the (+) side

ATPase domain of HSP90 chaperone/DNA topoisomerase II/histidine kinase

The αβγ coordination is unambiguous in this family and all active sites have one metal ion. Biochemical studies of the mitochondrial Hsp90 paralogue TRAP1 demonstrate that Mg^{2+} is essential specifically for catalytic hydrolysis: omitting Mg^{2+} from the reaction results in accumulation of the ATP-bound closed conformation without hydrolysis, confirming that Mg^{2+} is dispensable for substrate binding but critical for the chemical step.[37]

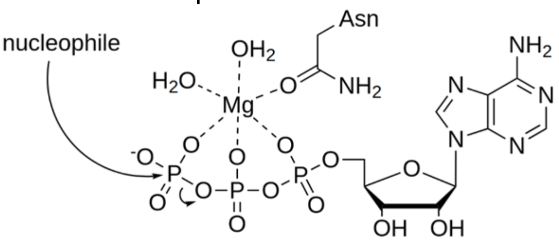

Typical coordination and the reaction facilitated by the ATPase domain of HSP90 chaperone/DNA topoisomerase II/histidine kinase superfamily.

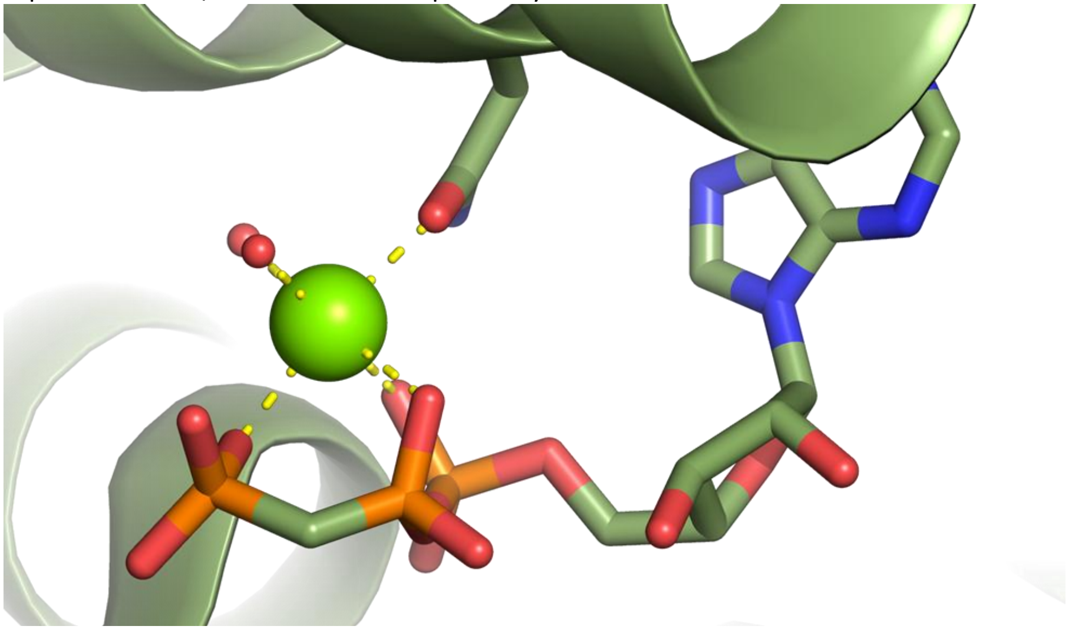

ATP binding represented by PDB structure 1I58-A.

Carbamate kinase-like

Some enzymes are known to have kinase function on small molecules; however, many molybdenum storage proteins share this fold. Their ATPase activity is connected to molybdenite release.[38] These structures are not necessarily in their active forms and exhibit only αβ phosphate coordination. In the kinases, the magnesium ion coordinates all three phosphates. Classic kinetic analysis of crystalline carbamate kinase established that Mg^{2+} (or Mn^{2+}) is an essential cofactor for both forward and reverse reactions, with the Mg-ATP complex serving as the true substrate.[39]

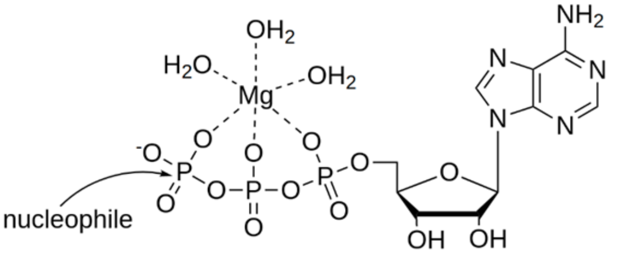

Typical coordination and the reaction catalyzed by the Carbamate kinase-like superfamily.

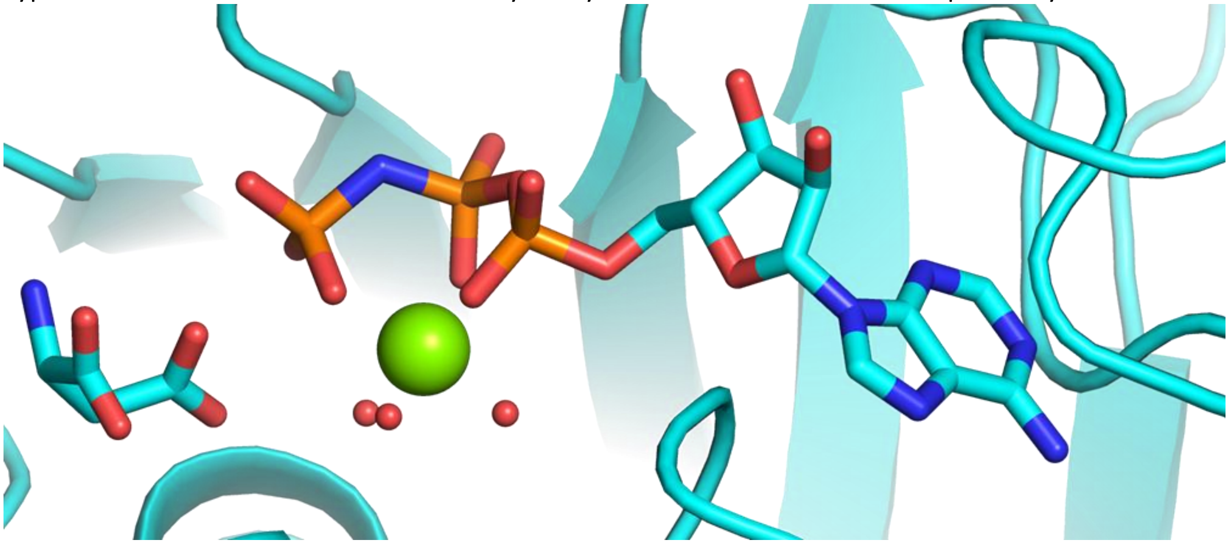
ATP and metal ion coordination in the representative structure 3C1M.

GHMP Kinase, C-terminal domain (Ribosomal protein S5 domain 2-like)

The collected structures agree in the active site architecture, binding the ATP at the end of a helix, although in two of them the magnesium ion appears misplaced and is not coordinated by the conserved serine. Interestingly, this superfamily is commonly identified in the same chain as more common ones as 'ATPase domain of HSP90 chaperone/DNA topoisomerase II/histidine kinase' and 'P-loop containing nucleoside triphosphate hydrolases'. Biochemical characterization of glucuronokinase (AtGlcAK), a member of the GHMP kinase superfamily, demonstrates that divalent metal ions are strictly required for activity: Mg^{2+} supports maximal turnover, Mn^{2+} and Co^{2+} can partially substitute, trivalent cations give only marginal activity, and critically, no activity is detected with monovalent cations.[40]

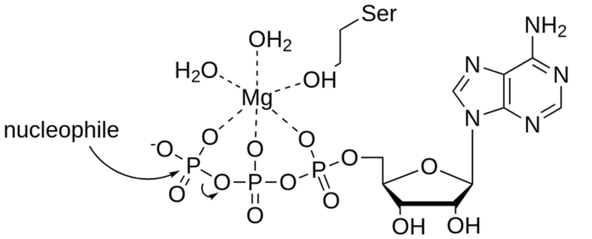

Typical coordination and the reaction facilitated by the GHMP Kinase, C-terminal domain superfamily.

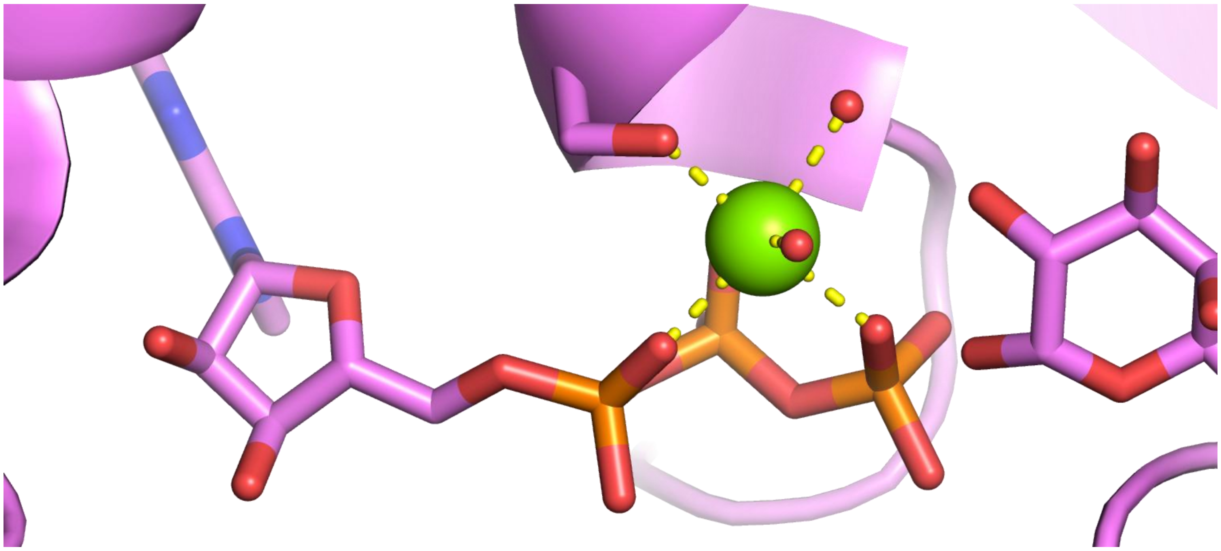

ATP binding represented by PDB structure 3V2U.

Nucleoside diphosphate kinase, NDK

Based on the EC association, this superfamily uses ATP to phosphorylate an NDP or L-histidine.
Nucleoside diphosphate kinase (NDPK) operates via a Mg^{2+}-dependent ping-pong mechanism in which the metal ion coordinates the γ-phosphate of ATP to form the active Mg–NTP complex; both autophosphorylation of the catalytic histidine residue and subsequent phosphoryl transfer to the NDP acceptor are abolished upon chelation of Mg^{2+} with EDTA, confirming that the true substrate is the Mg–ATP complex.[41]

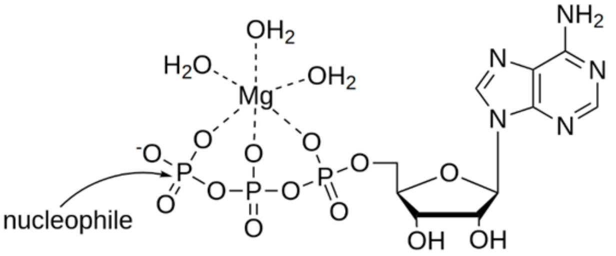

Typical coordination and the reaction facilitated by the Nucleoside diphosphate kinase, NDK superfamily.

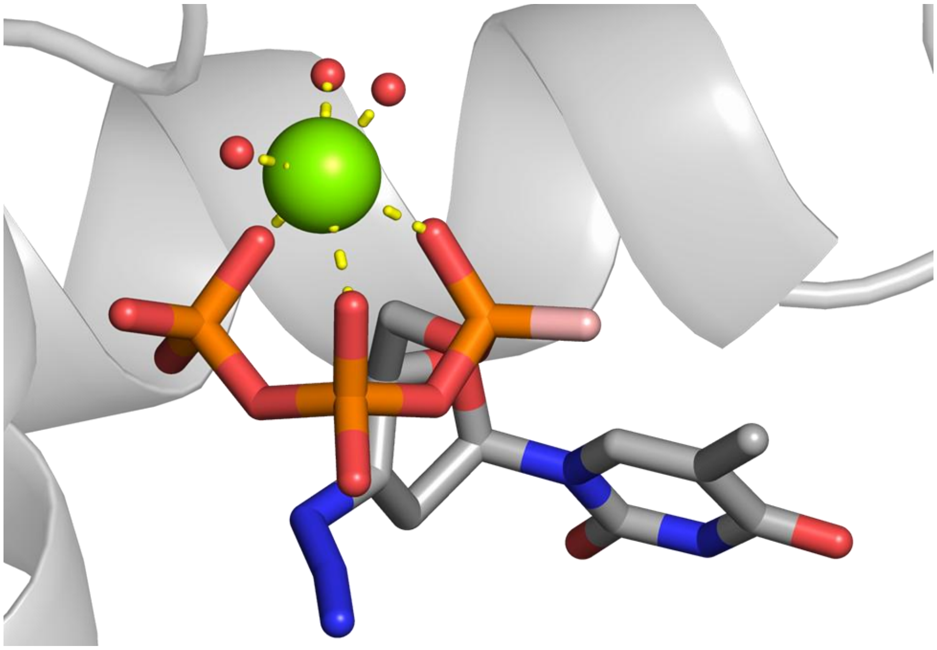

Nucleotide binding represented by PDB structure 1F3F.

GroEL equatorial domain-like

This superfamily also presents αβγ coordination to facilitate ATPase activity.

Mg^{2+} binding to the equatorial domain of GroEL is required for ATP-driven conformational cycling; in the absence of Mg^{2+}, the chaperonin ring remains predominantly in the apo $[GroEL]_7$ state even after prolonged incubation, demonstrating that Mg^{2+} is essential for productive ATP binding and the allosteric transitions that drive GroES association and substrate release.[42]

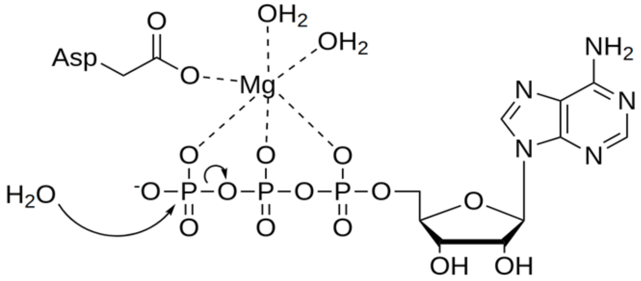

Typical coordination and the reaction facilitated by the GroEL equatorial domain-like superfamily.

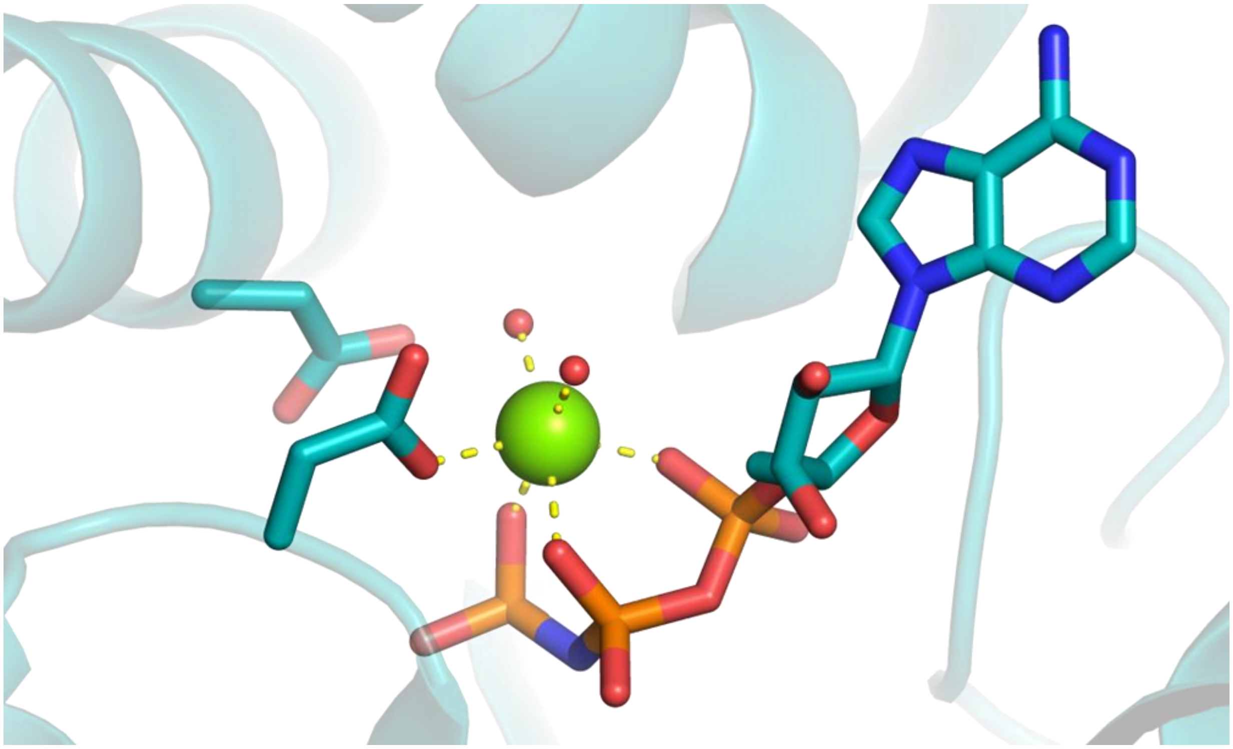

ATP binding represented by PDB structure 3RUV.

ParB/Sulfiredoxin

Similarly to the NDK, the Sulfiredoxin superfamily contains a few specialized structures, all with clear αβγ phosphate coordination.

Sulfiredoxin requires Mg^{2+} (or Mn^{2+}) as an essential divalent cofactor for ATP-dependent reduction of hyperoxidised peroxiredoxin; neither Ca^{2+}, Zn^{2+}, Fe^{2+}, nor Cu^{2+} can substitute, and chelation with EDTA abolishes activity that is fully restored by readdition of Mg^{2+} or Mn^{2+}.[43]

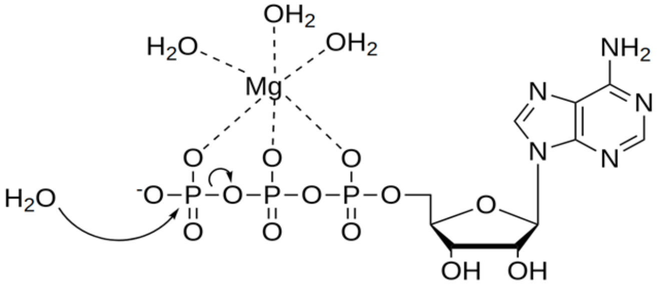

Typical coordination and the reaction facilitated by the Sulfiredoxin superfamily.

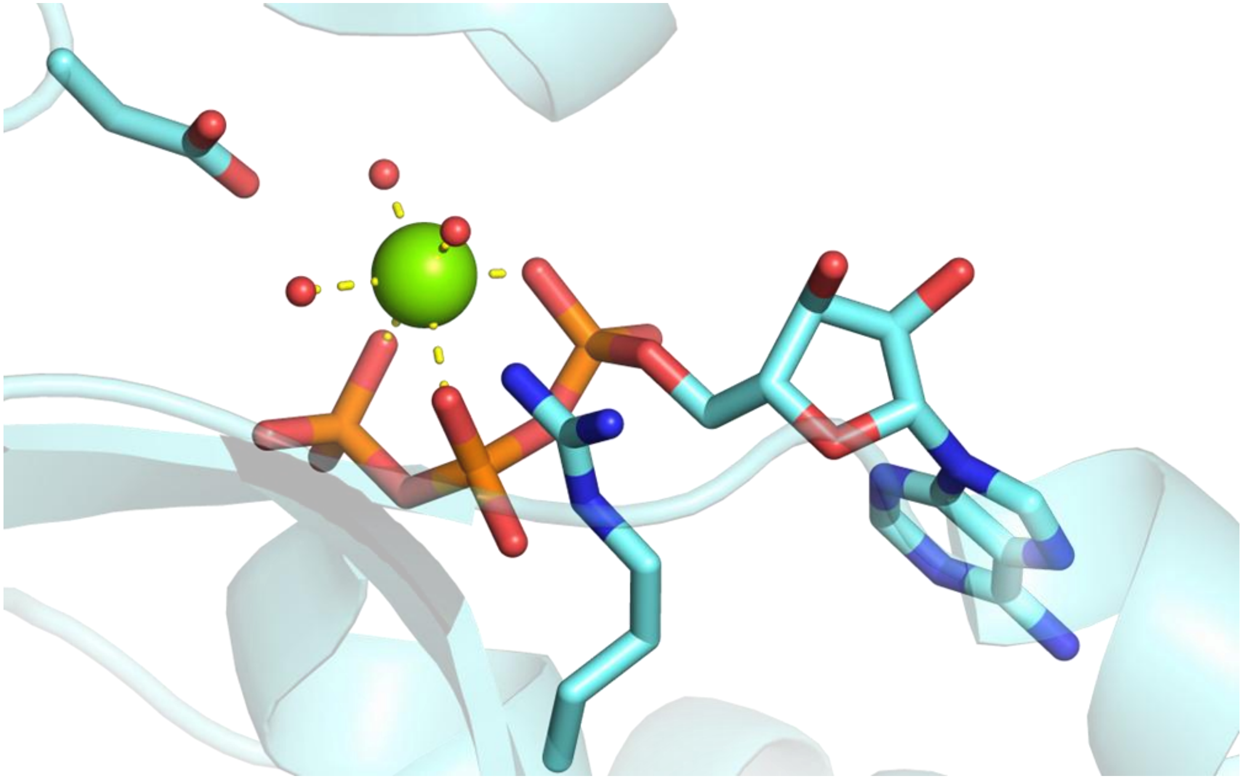

ATP binding represented by PDB structure 3HY2.

NAD kinase/diacylglycerol kinase-like

Uses an αβγ phosphate coordination like the superfamilies above. There are structures with bound NAD^+ and $NADP^+$, but the binding pose overlaps with the ATP binding, therefore it is unclear how the catalytic conformation looks like. Based on EC assignments, it is also active on other substrates.

NAD^+ kinase utilises Mg–ATP as the active co-substrate, with Mg^{2+} coordinating the phosphate groups of ATP to enable phosphoryl transfer to NAD^+ and produce $NADP^+$; the enzyme is reversible, and the apparent irreversibility of the human isoform arises from NAD^+ product inhibition rather than thermodynamic constraints.[44]

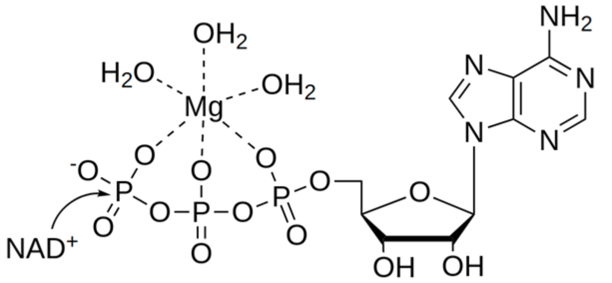

Coordination and reaction facilitated by the NAD^+ kinase superfamily, based on the example 1Z0Z.

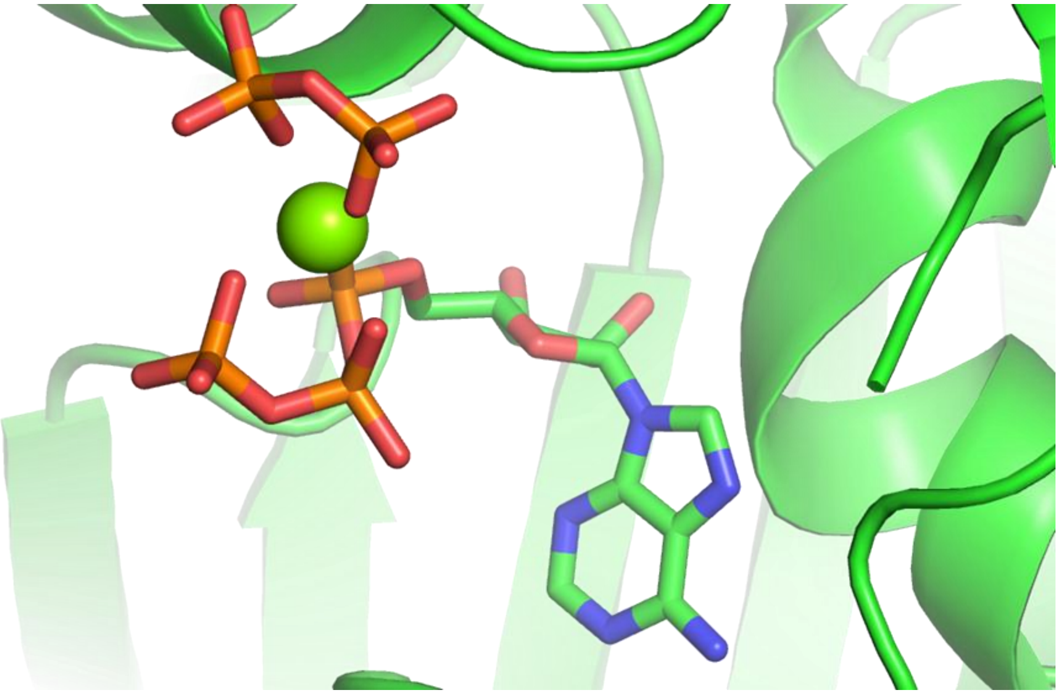

ATP binding represented by PDB structure 1Z0Z.

Riboflavin kinase-like

The fold often occurs alongside the *Adenine nucleotide alpha hydrolases-like*, but it is associated with phosphatase activity. 2.7.1.26 consumes ATP, 2.7.1.161 is CTP dependent, they align reasonably well. Limited number of NTP bound structures lack ions, 5TRD contains a sodium from which we derive the likely αβγ coordination.

Rat liver flavokinase (riboflavin kinase; EC 2.7.1.26) requires a divalent metal–ATP complex as the phosphoryl donor for riboflavin phosphorylation; both Zn^{2+} and Mg^{2+} support catalysis, whereas Ca^{2+} and Co^{2+} do not, establishing that divalent metal coordination to ATP is a strict prerequisite for flavokinase activity.[45]

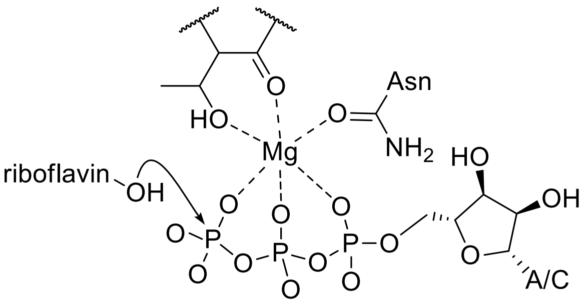

Coordination and reaction facilitated by the Riboflavin kinase-like superfamily, based on the example 5TRD.

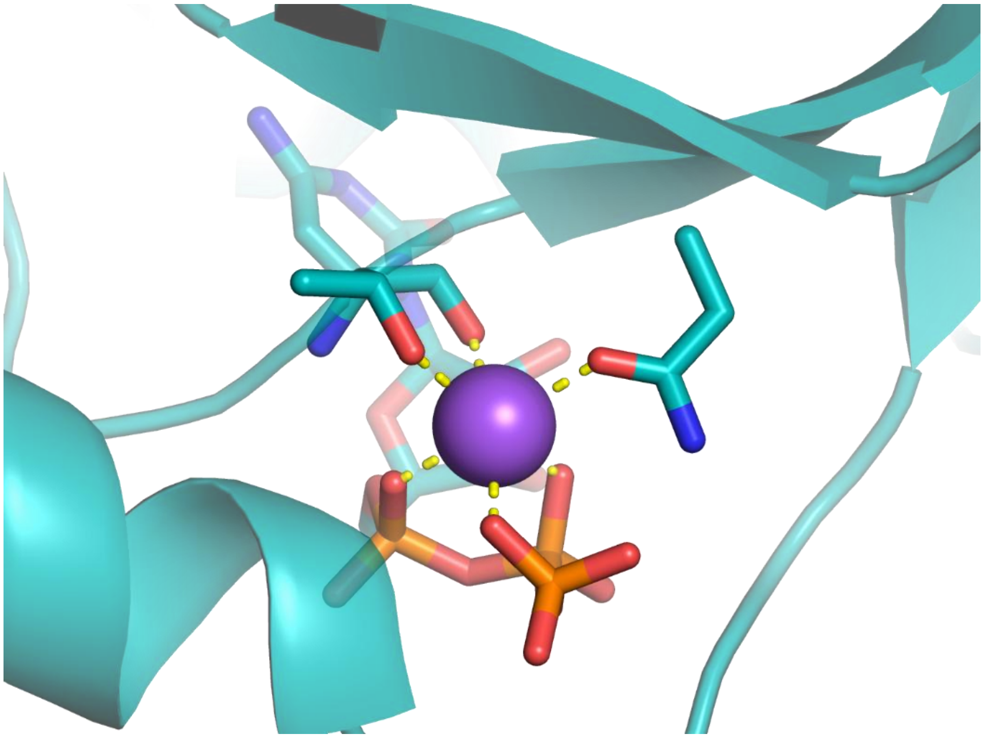

NTP binding and ion coordination represented by PDB structure 5TRD.

PurM N-terminal domain-like

Besides the αβγ coordinating metal ion, which is always present, members of this fold contain other metal ions as well. One additional βγ-coordinating ion is resolved in most structures, although in some cases three or four metal ions are present in the active site.

Biochemical characterisation of selenophosphate synthetase (SelD), a member of the PurM-like superfamily, demonstrates strict and dual cation dependence for its ATP-dependent reaction: both Mg^{2+} and a monovalent cation (K^+, NH_4^+, or Rb^+) are required for catalytic activity; omission of either abolishes synthesis of monoselenophosphate from ATP and selenide, establishing Mg^{2+} as an essential cofactor in this superfamily.[46]

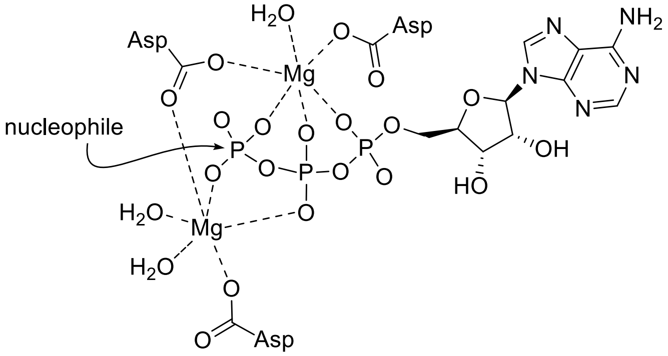

Metal coordination of the two main metal sites and reaction facilitated by the PurM N-terminal domain-like superfamily. Additional ions are present in some structures, coordination is represented by 5DD7.

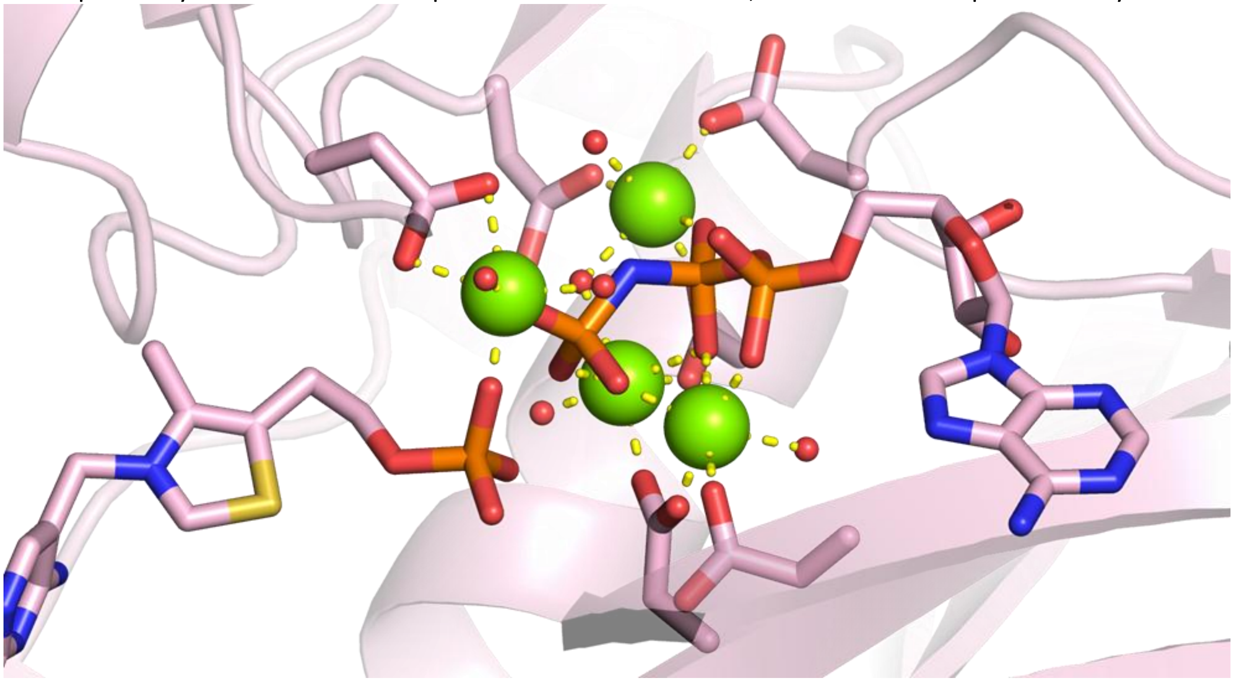

ATP and substrate binding represented by PDB structure 5DD7.

CYTH-like phosphatases

The main reaction carried out by proteins in the superfamily is the hydrolysis of inorganic triphosphates. One crystal structure had a bound ATP, hence the similarity of phosphatase enzymes could be identified. Interestingly, class IV adenylyl cyclases are also members of this superfamily,[47] despite the cyclase activity cleaving a pyrophosphate.

All characterized members of the CYTH superfamily act on triphosphorylated substrates and require Mg^{2+} for catalysis; the mammalian 25-kDa thiamine triphosphatase exemplifies this dependence, as full activity requires both Mg^{2+} coordinated to the substrate and millimolar free Mg^{2+} at a distinct activator site on the enzyme, a requirement proposed to be a general feature of the CYTH family.[48]

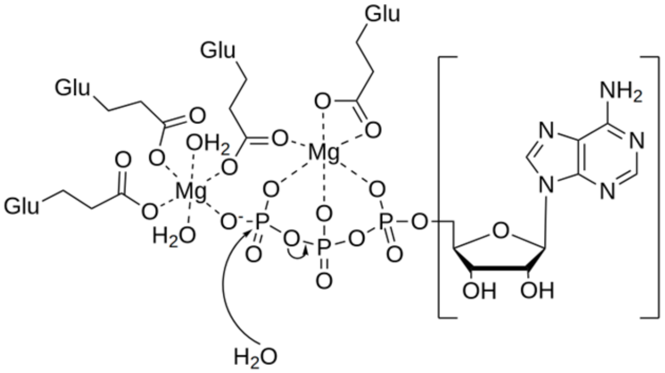

Coordination and phosphatase reaction facilitated by the CYTH-like phosphatases superfamily.

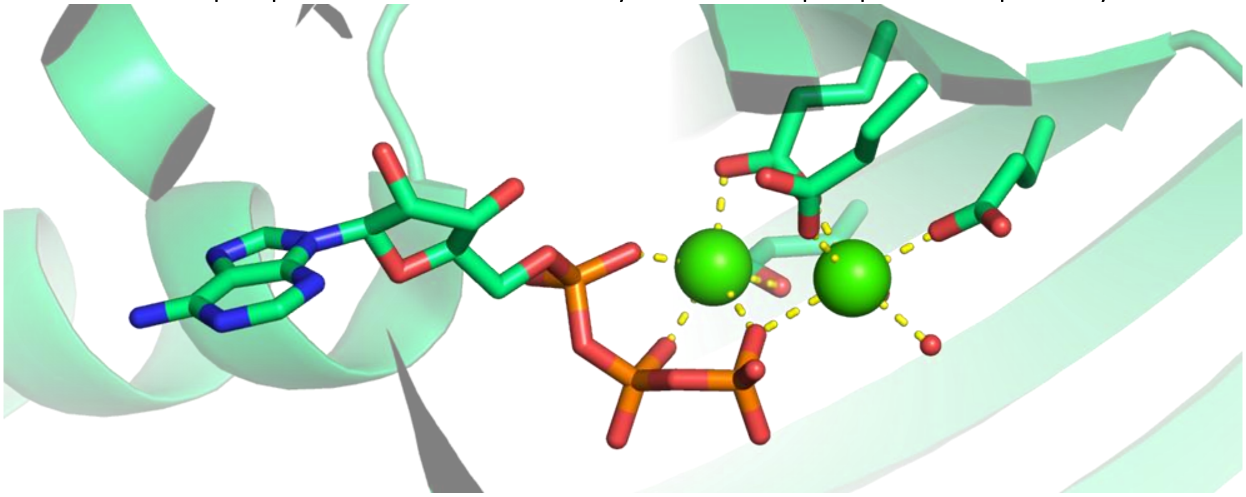

Substrate binding represented by PDB structure 7NSD.

CofE-like

The study corresponding to the only two deposited structures for this superfamily had not yet been published at the time of our analysis. They have two metal ions; one Mn^{2+} ion is coordinated by the nonesterified oxygens of the αβγ phosphates, a glutamate and a threonine residue, and a water molecule. The other Mn^{2+} ion is coordinated by an oxygen of the γ phosphate, two aspartate residues, and three water molecules.

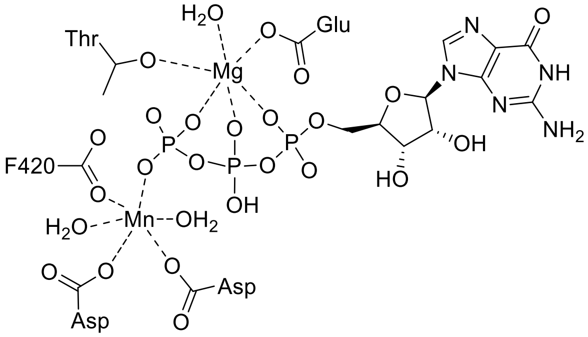

Based on EC annotation, the catalyzed reaction is a two-step amide bond formation, of which the first is a kinase-like reaction by the carboxyl group of coenzyme F420 variants. The αβγ coordinating ion is thought to be a magnesium, while a manganese coordinates the nucleophile as well, not resolved in the experimental structure.[49]

CofE catalyzes the ATP-dependent addition of two glutamate residues to F420-0 to form F420-2 in F420 coenzyme biosynthesis; the reaction has an absolute requirement for divalent metal ions — both Mn^{2+} and Mg^{2+} support activity, while EDTA abolishes it, establishing that a divalent metal–ATP complex is essential for each amide-bond-forming step.[49]

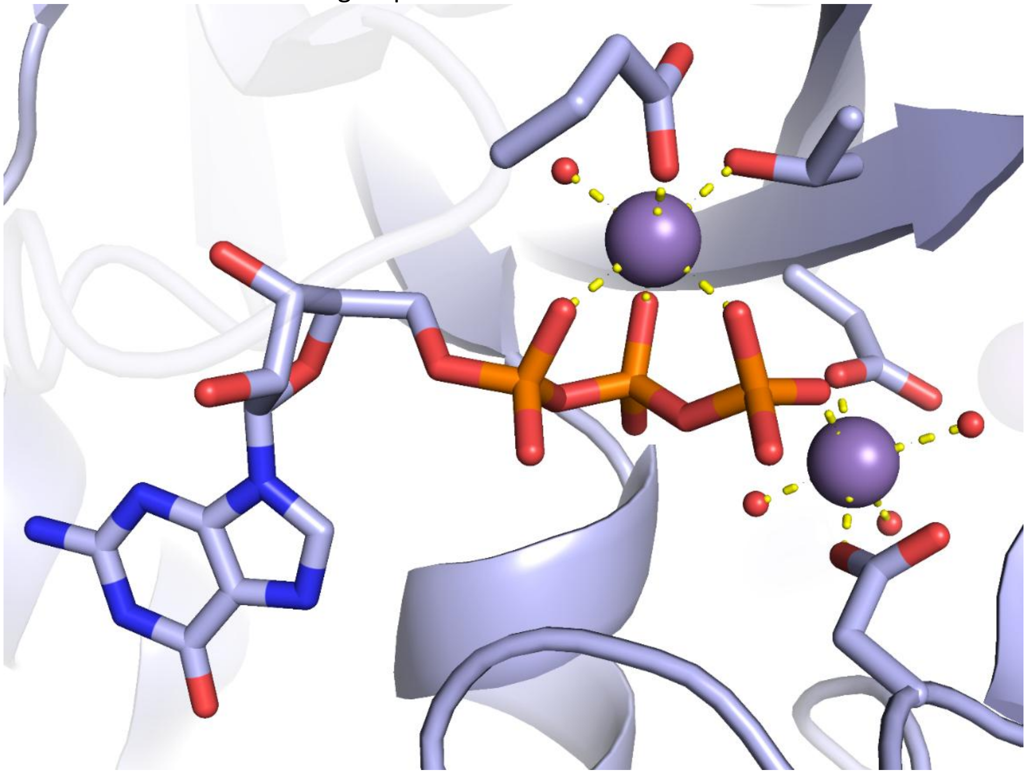

NTP binding and ion coordination represented by PDB structure 7ULD.

Phosphatases with βγ coordination from the (+) side

Actin-like ATPase domain

With well over a hundred structures, the Actin-like ATPase domain superfamily having a well-established ATP active site with a single magnesium ion coordinating the βγ phosphates.[50]

Members of the Actin-like ATPase superfamily depend critically on Mg^{2+} for activity; Mg^{2+} binds to a regulatory site on monomeric actin (G-actin) and induces the conformational change that enables polymerisation, with the Mg–ATP form (not Ca–ATP) being the polymerisation-competent species, and Mg–ADP is retained in the assembled filament after ATP hydrolysis.[51]

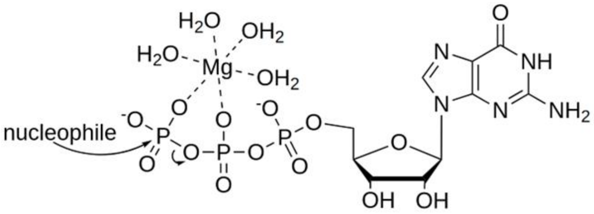

Typical coordination and the reaction facilitated by the Actin-like ATPase domain superfamily.

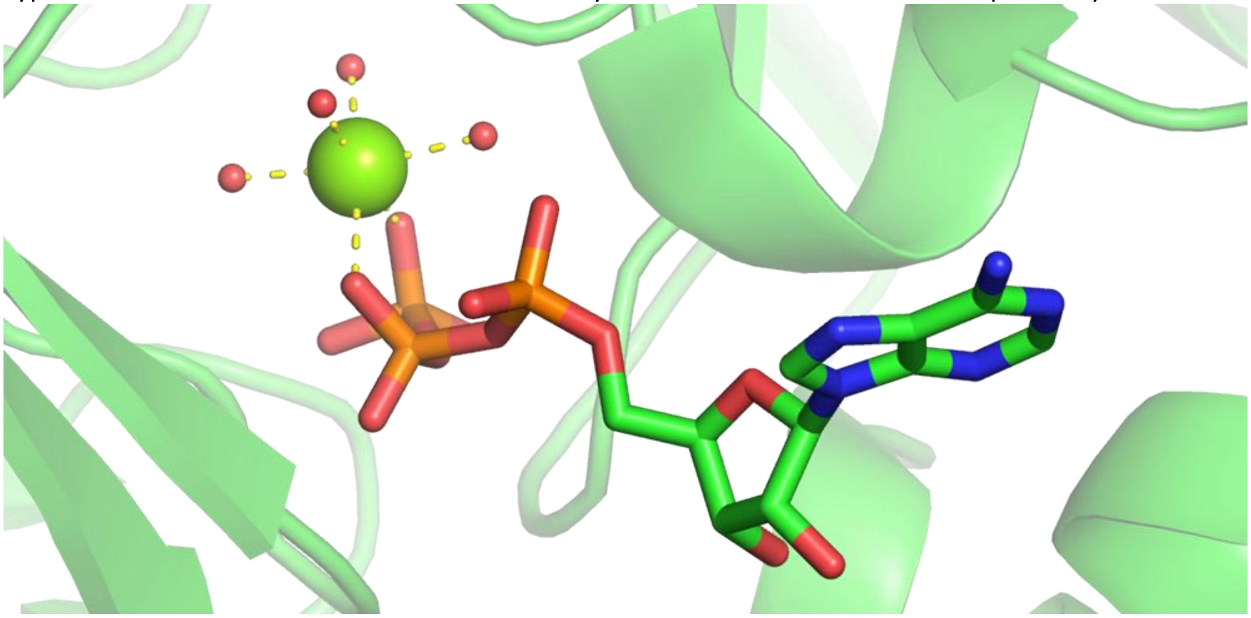

Substrate binding represented by PDB structure 1D4X.

Glutamine synthetase/guanido kinase

This is the only superfamily which features ions on the (+) side of the triphosphate and AG ion in its plane at the same time, the first only pinching the β and γ phosphates. A third ion is present near the nucleophile. This coordinative arrangement is very similar to the mirror image of the one of Glutathione synthetase ATP-binding domain-like superfamily.

Glutamine synthetase requires two divalent metal ions per active site for catalytic activity; only Co^{2+}, Mg^{2+}, and Mn^{2+} support the γ-glutamyl transfer reaction, with Mg^{2+} providing the highest activity, whereas Ca^{2+}, Zn^{2+}, and Cu^{2+} are inactive, confirming that the divalent cation requirement is both specific and essential for catalysis.[52]

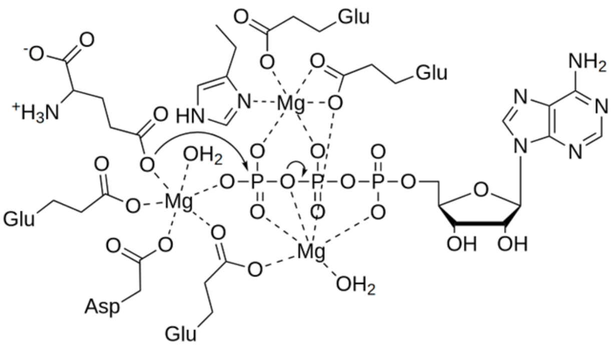

Coordination network and reaction facilitated by the Glutamine synthetase/guanido kinase superfamily.

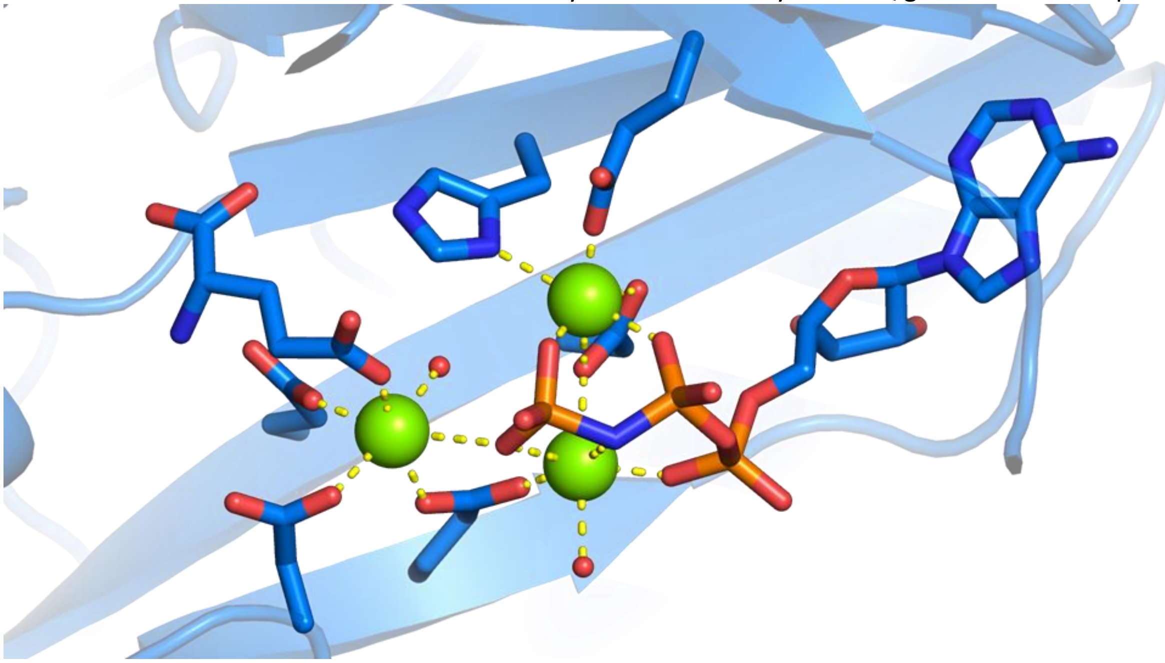

Metal ion coordination represented by the crystal structure 7CQQ.

Phosphatases with αβγ coordination on the (-) side

GlnB-like

Although the position of the metal ion is quite well-defined, it is unclear from the structures whether any protein residue participates in metal coordination, and in many cases organic substrates chelate the metal ion, hence we displayed waters in coordination as in the representative structure 2J9C.

The GlnB-like (PII) signalling protein binds Mg–ATP as its key effector; the Mg^{2+}/ATP-loaded form of GlnB interacts with the biotin carboxyl carrier protein BCCP to control acetyl-CoA availability in cyanobacteria, and both Mg^{2+} and ATP are strictly required for this regulatory protein–protein interaction.[53]

GlnD-catalyzed modification of GlnB-like PII signal transduction proteins requires divalent metal ions; deuridylylation is strictly Mn^{2+}-dependent, and no deuridylylation activity is detected in the absence of Mn^{2+} even when Mg^{2+} is present.[54]

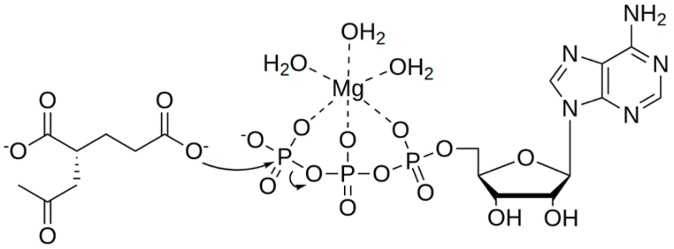

Typical coordination and the reaction catalyzed by the GlnB-like superfamily.

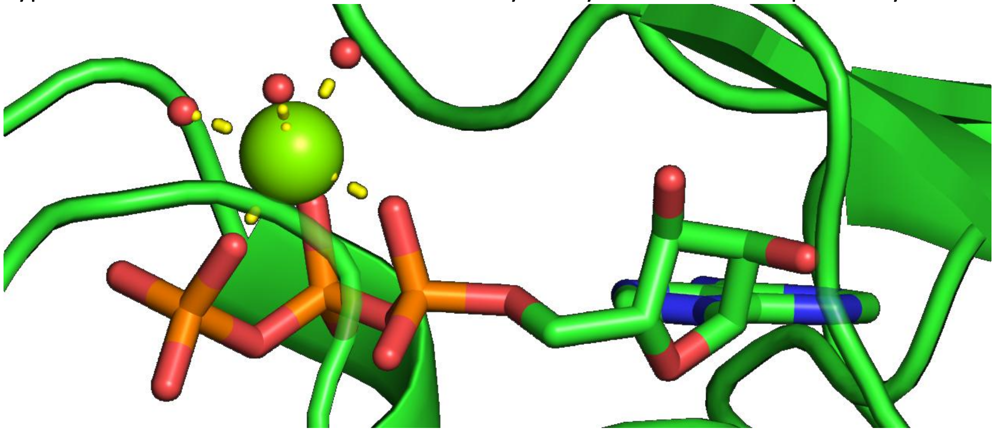

Metal ion and ATP coordination represented by the crystal structure 2J9C.

Phosphoglycerate kinase

This superfamily contains structures only for a specific EC, exhibiting a clear αβγ phosphate coordination. Phosphoglycerate kinase requires Mg^{2+} to form the Mg–ATP co-substrate and stabilise the phosphoryl-group transition state; the reaction is also supported by $MnATP^{2-}$ with approximately 90% efficiency, and a $^{25}Mg^{2+}$-dependent magnetic isotope effect on reaction rate implicates Mg^{2+} directly in the phosphoryl-transfer transition state.[55]

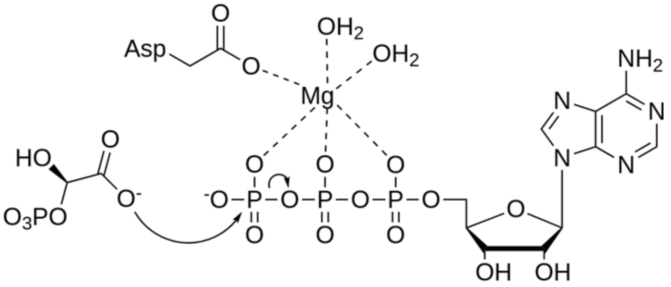

Typical coordination and the reaction catalyzed by the member of the phosphoglycerate kinase superfamily.

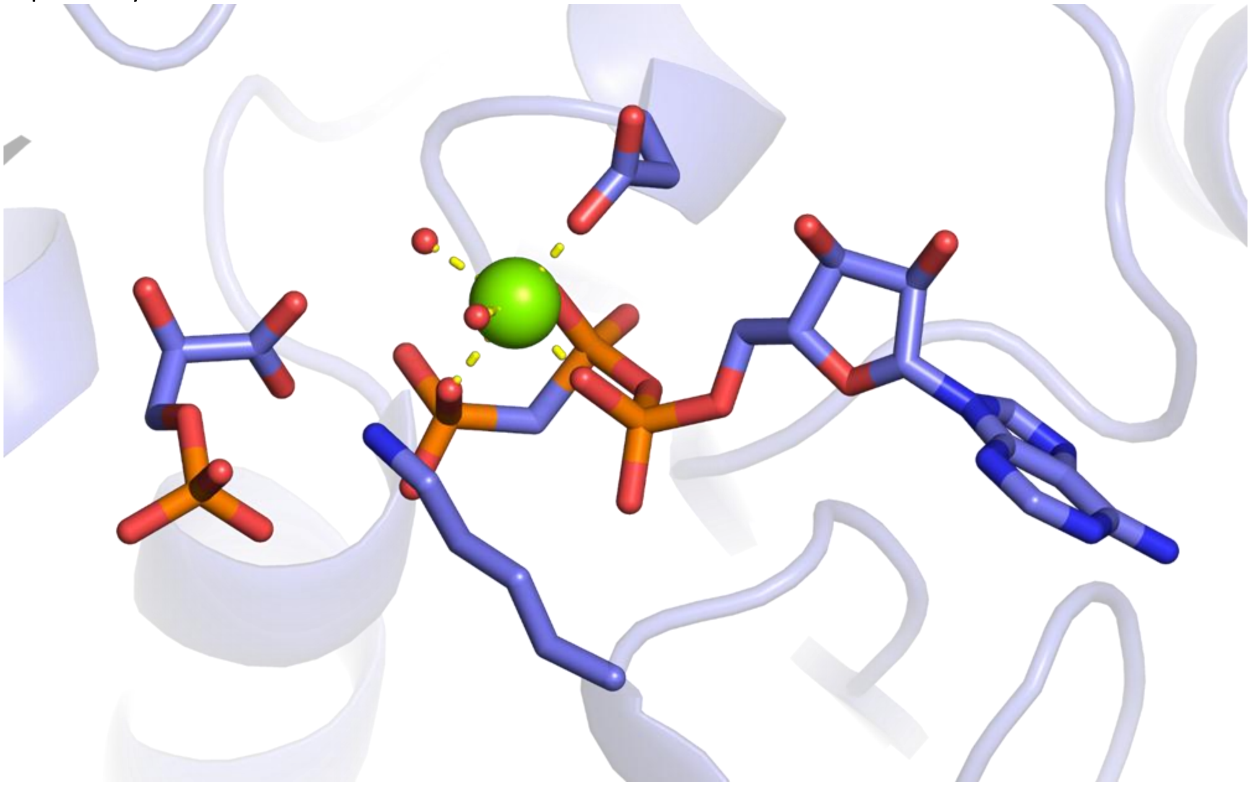

ATP and metal ion coordination in the representative structure 2X14.

Phosphoenolpyruvate/pyruvate domain

A few structures corresponding to EC 4.1.3.25 do not have a NTP residue or phosphatase activity, however their sequences do resemble the superfamily. In the analysis of the coordination, they are omitted. In addition to the magnesium in contact with the αβγ phosphates, a second one can be found near the pyruvate.

The superfamily also includes structures of the pyruvate phosphate dikinases (EC 2.7.9.1-2), which perform pyrophosphatase activity.[56]

Pyruvate kinase has a dual Mg^{2+} requirement: one metal ion binds directly to the enzyme and is required for substrate binding and catalysis, while a second Mg^{2+} coordinates the ATP triphosphate moiety to form the metal–nucleotide co-substrate; chelation of either metal abolishes activity, establishing that both enzyme-bound and nucleotide-bound Mg^{2+} are essential for phosphoryl transfer from PEP to ADP.[57]

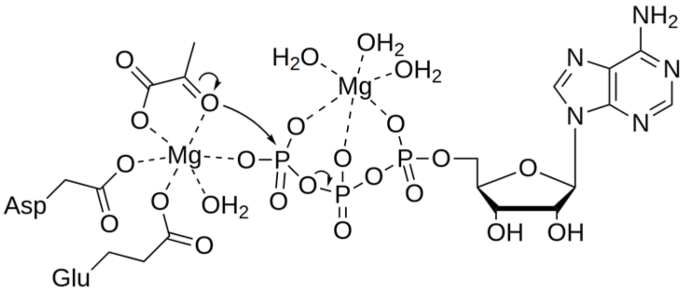

Typical coordination and the reaction facilitated by the members of the Phosphoenolpyruvate/pyruvate domain superfamily.

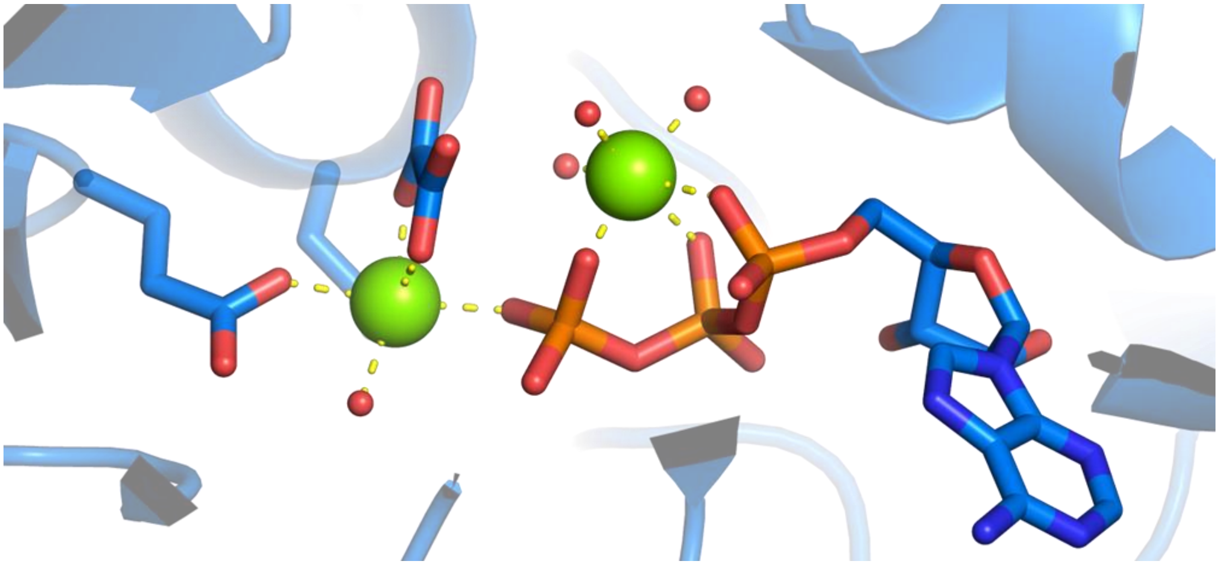

ATP and metal ion binding in the representative structure 6KSH.

Phosphofructokinase

Phosphofructokinases (PFKs) are enzymes that catalyze the production of fructose 1,6-bisphosphate (F-1,6-BP) early in the glycolytic pathway. In mammals, plants, yeasts, many protists, and bacteria, PFKs use ATP as the phosphate donor in this essentially irreversible reaction.[58] The Mg^{2+} ion is coordinated by all three phosphate groups, and by the carboxyl group of an aspartate that emerges from a large loop inserted near the active site.

Phosphofructokinase (PFK) is inactive in the absence of Mg^{2+}; in addition to providing the Mg–ATP co-substrate, free Mg^{2+} acts as an allosteric activator of the human erythrocyte isozyme, and excess Mg^{2+} above the stoichiometric amount for ATP chelation is required for full catalytic activity.[59]

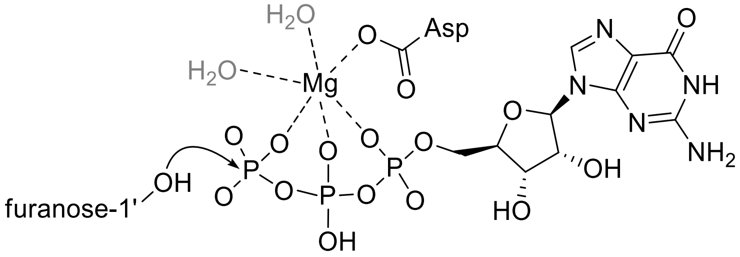

Sugar kinase reactivity and Mg binding in the Phosphofructokinase superfamily.

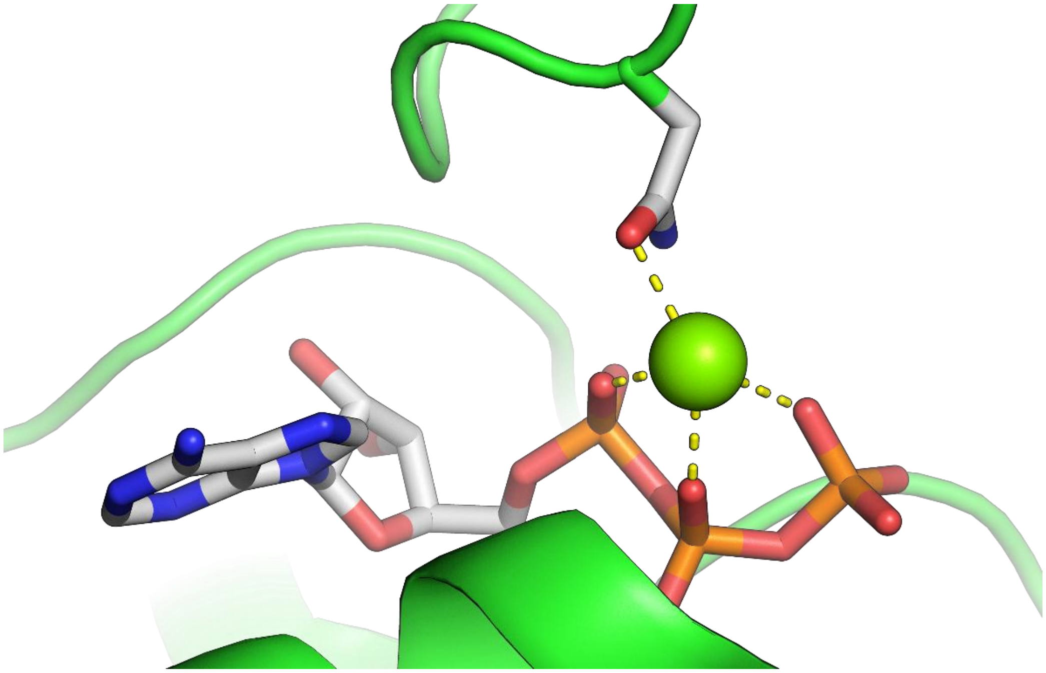

ATP and metal ion binding in the representative structure 3F5M.

Phosphatases with βγ coordination on the (-) side

P-loop containing nucleoside triphosphate hydrolases

By far the largest superfamily in our dataset. Despite the numbers, the coordination is quite conserved and the structures align well. In a small part of the superfamily, the coordinating threonine is missing, which resembles the conformation adopted after the phosphate hydrolysis in some enzymes.

P-loop NTPases require Mg^{2+} as an essential cofactor for NTP hydrolysis; in Ras GTPase, Mg^{2+} coordinates the β- and γ-phosphates of GTP and positions the attacking water molecule, and point mutations at the Mg^{2+}-binding residues abolish GTP hydrolysis and lock the protein in a constitutively inactive state, demonstrating that metal ion coordination is indispensable for catalysis.[60]

In support of the universal requirement, no helicase was found to exhibit significant ATPase activity without divalent cations.[61]

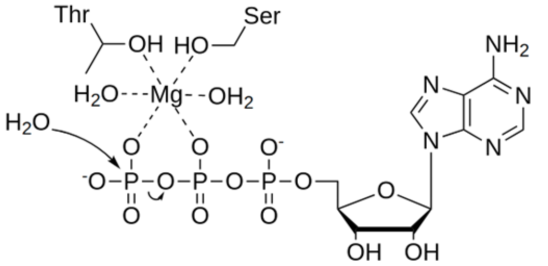

Typical coordination and the reaction catalyzed by the members of the P-loop containing nucleoside triphosphate hydrolases superfamily, demonstrated on ATP.

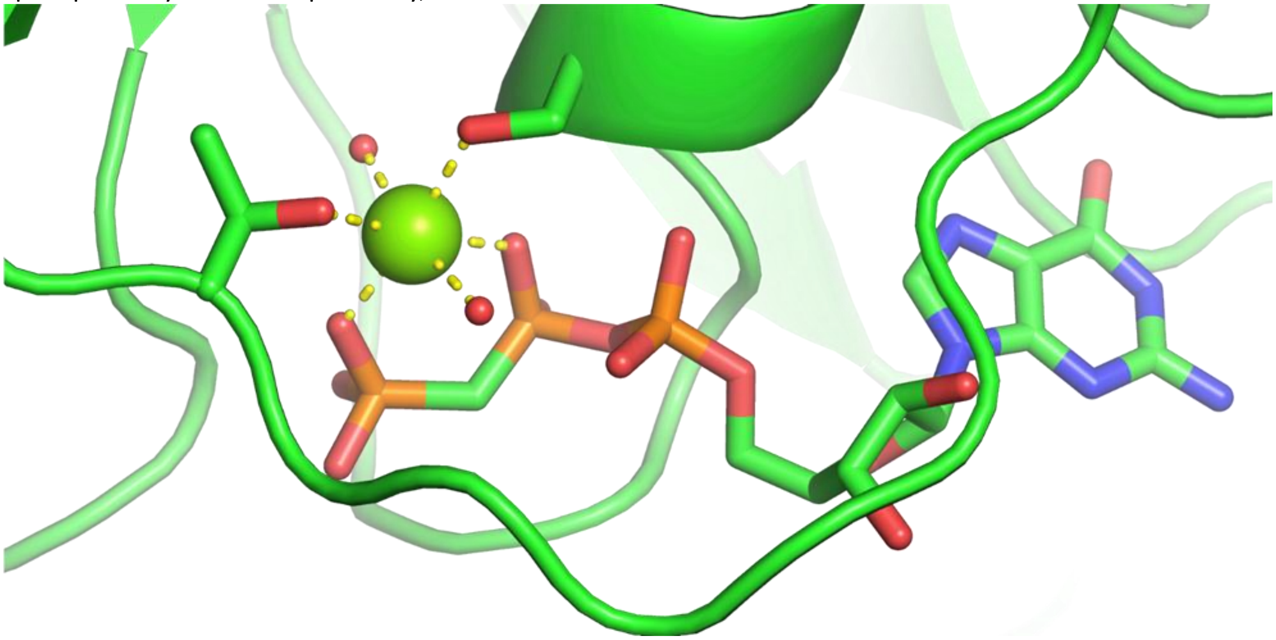

GTP and Mg^{2+} coordination in the representative structure 121P.

Tubulin nucleotide-binding domain-like

There is a good agreement in the active site of the PDB structures of the Tubulin nucleotide binding domain superfamily. It is a well-known GTPase,[62] although not all tubulin isoforms hydrolyze the bound GTP.

Tubulin requires Mg^{2+} for GTP binding, heterodimer stability, and microtubule polymerisation; Mg^{2+} promotes correct 13-protofilament assembly, while Zn^{2+} and Co^{2+} induce aberrant polymers with more than 13 protofilaments, and Mn^{2+} can substitute to drive assembly, confirming that divalent cation coordination is essential for productive tubulin self-assembly.[63]

MAP-dependent assembly of tubulin-GDP can proceed with minimal free Mg^{2+} (<3 µM) but is totally inhibited by EDTA; exogenous magnesium chloride stimulates polymerisation in a concentration-dependent manner, and Mg^{2+} plays a critical role in assembly distinct from its enhancement of GTP binding to the exchangeable site.[64]

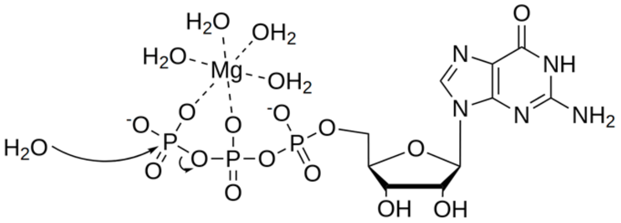

Typical coordination and the reaction catalyzed by the members of the Tubulin nucleotide-binding domain-like superfamily

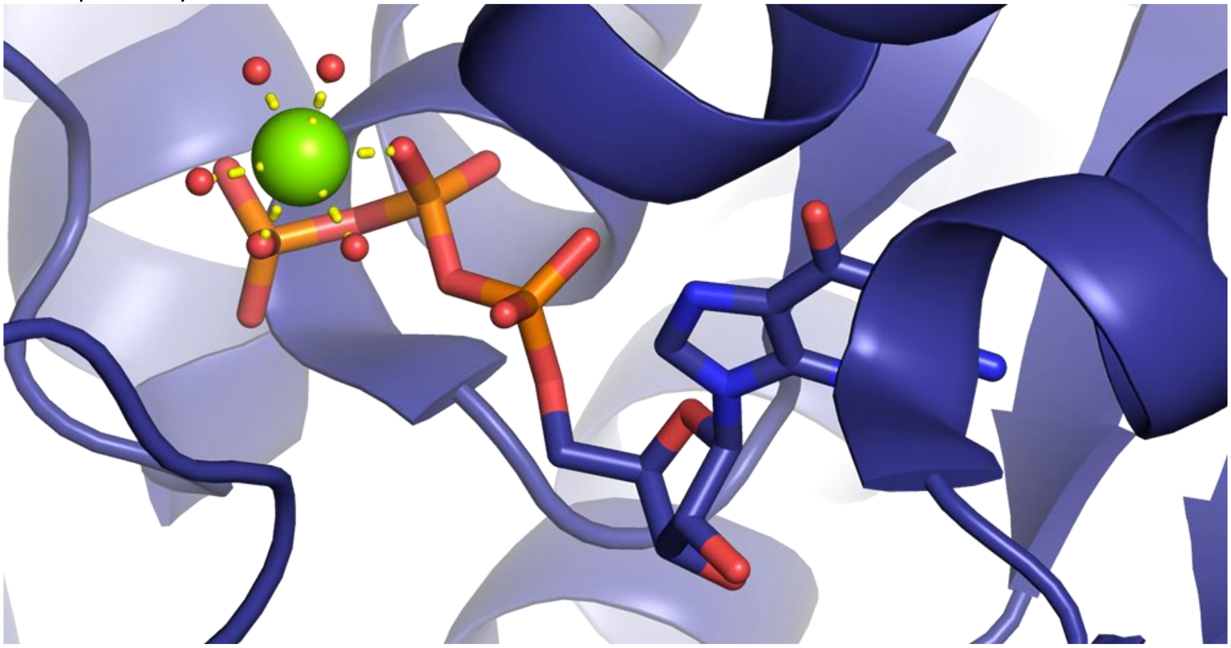

GTP and metal ion binding in the representative structure 7ALR.

PEP carboxykinase-like

The fold of the PEP carboxykinase-like superfamily resembles the one of P-loop containing nucleoside triphosphate hydrolases, especially the p-loop and the helix it transitions into. However, the structures often contain a second metal ion. There are examples working with ATP, GTP and ITP.

PEP carboxykinase (PEPCK) requires two divalent cations for catalysis: an enzyme-bound metal (preferably Mn^{2+}) and a second metal ion (Mg^{2+}) that coordinates the nucleotide triphosphate to form the Mg–NTP co-substrate; both sites must be occupied for productive phosphoryl transfer between oxaloacetate and the nucleotide, and the reaction is abolished when either metal is removed.[65]

In avian liver PEPCK, Mg^{2+} (and Mn^{2+}) activate catalysis; a monovalent cation (potassium or ammonium) is also required, and the divalent metal coordinates the nucleotide substrate to stabilise the phosphoryl-transfer transition state.[66]

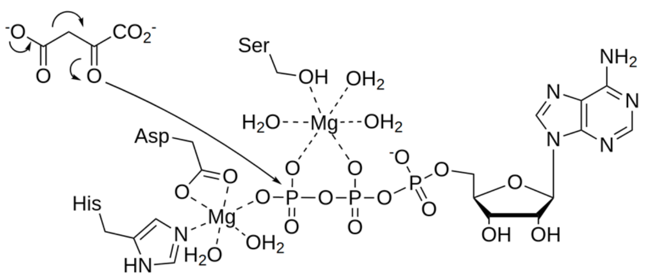

Typical coordination and the reaction catalyzed by the members of PEP carboxykinase-like superfamily.

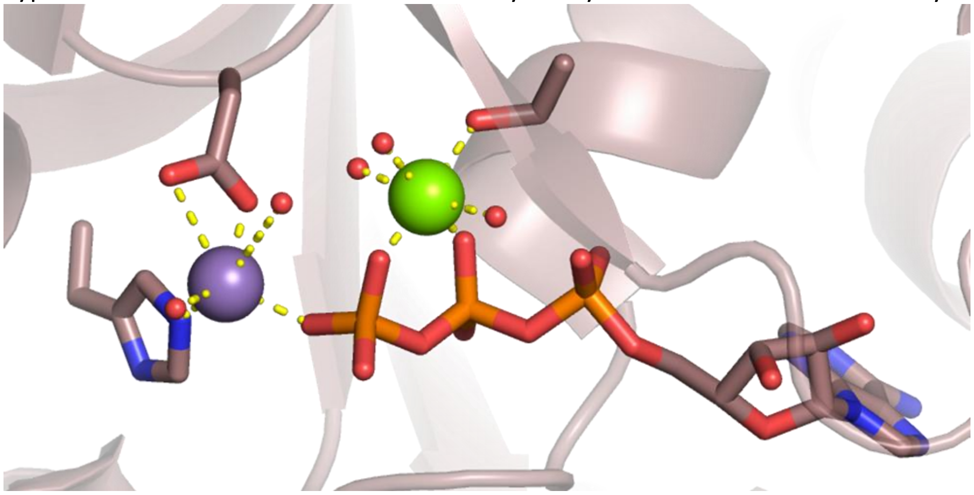
ATP and metal ion binding in the representative structure 2PY7.

Metal cation-transporting ATPase, ATP-binding domain N

The most difficult coordination case among the phosphatases is presented by ATP dependent cation transport proteins. Some of the Ca^{2+} transport proteins have a single αβγ coordinating ion but the nucleophile is far from the γ phosphate, others lack the Mg^{2+} pinch of the breaking phosphate bond. However, the only copper transporting protein structure (3A1C) has βγ coordination.

The true substrate for P-type ion-transporting ATPases is the Mg^{2+}–nucleotide complex; Mg^{2+} is an essential activator whose interaction with Na,K-ATPase modulates the phosphoenzyme cycle, and its removal abolishes ATPase activity.[67]

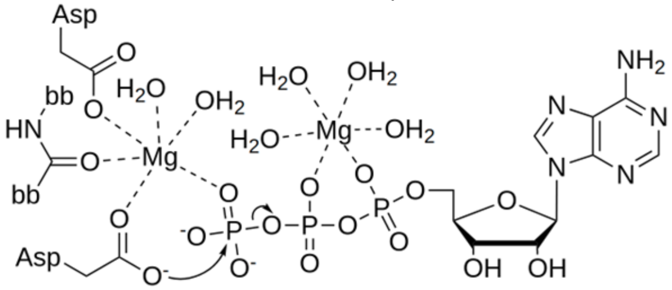

Assumed coordination of ATP in Metal cation transporting ATPases

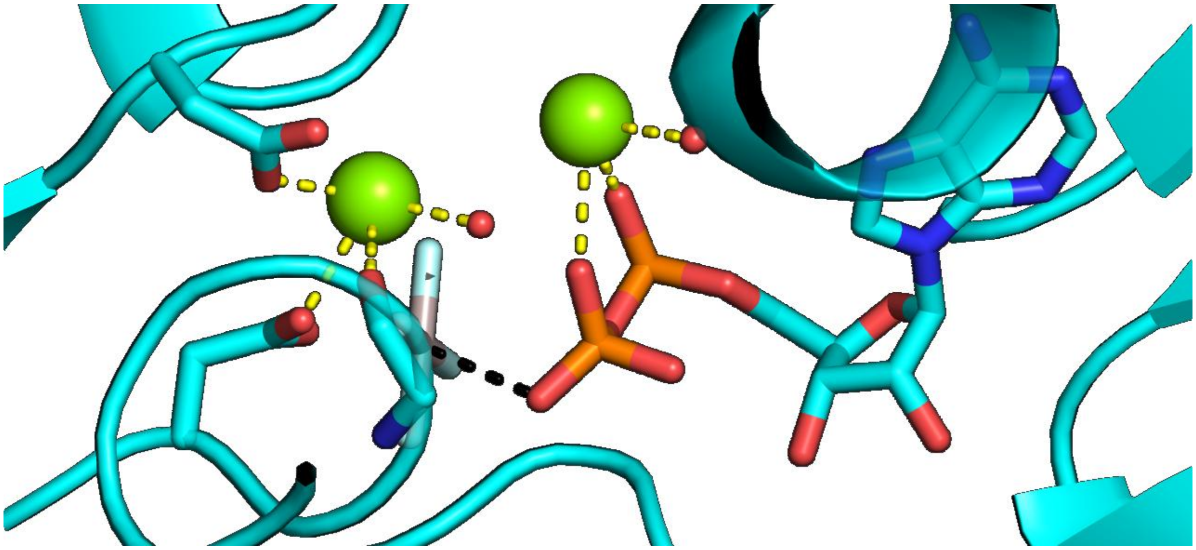
Metal ion coordination represented by the transition state mimicking crystal structure 3WGU.

MurD-like peptide ligases, catalytic domain

The active site of this superfamily is also similar to the P-loop containing nucleoside triphosphate hydrolases', but the serine is replaced by a threonine and a glutamate is involved in the coordination. It uses ATP to activate carboxylates for peptide synthesis.[68] A second metal ion is also present in some structures, in a position it may be involved in nucleophile coordination. Notably, it is a member of the Ribokinase-like fold, similarly to the Ribokinase-like superfamily.

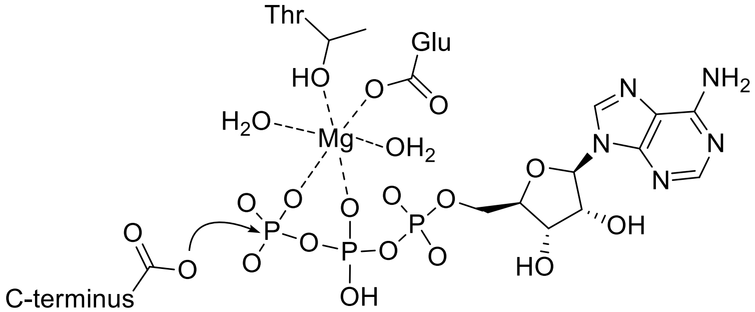

Two Mg^{2+} ions bridge negatively charged groups in the active site of MurD, and all Mur ligases require Mg^{2+} for catalytic activity.[69]

Typical coordination and the reaction catalyzed by the members of MurD-like peptide ligases, catalytic domain superfamily.

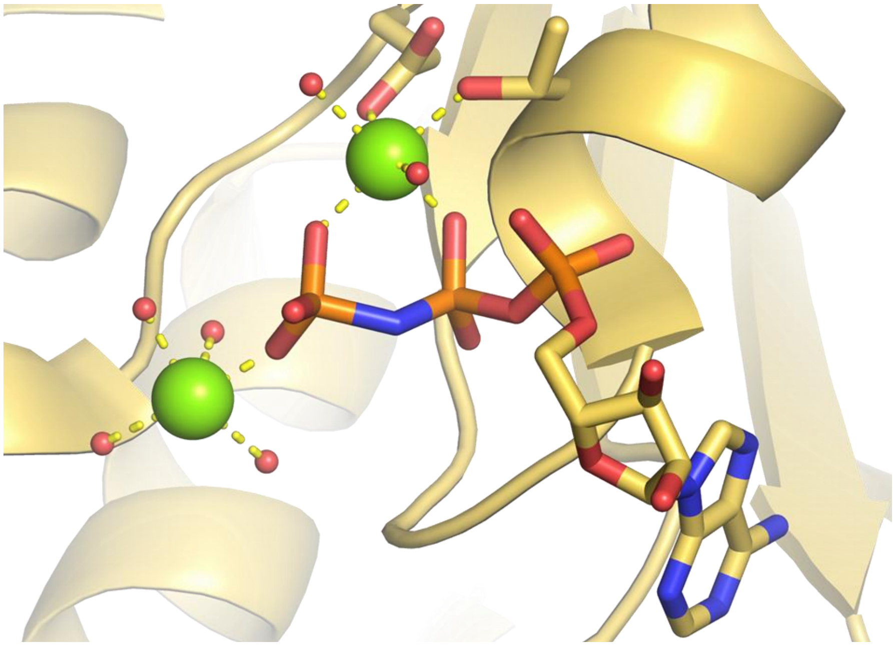
ATP and metal ion binding in the representative structure 6CAU.

Inositol-pentakisphosphate 2-kinase

Adopted from InterPro entry IPR043001.
Clear phosphatase activity with βγ coordinated magnesium cation. The majority of the structures do not contain NTP, but they align very well, the SUPFAM analysis does not assign them to any known fold/superfamily.
Crystal structures of IP5K in substrate and product complexes show that Mg^{2+} is strictly required: in the Michaelis complex one Mg^{2+} is coordinated by active-site residues Asp407 and Ser409, and in the product complex two Mg^{2+} ions bridge the inositide and nucleotide via Asp407, confirming Mg^{2+} as essential for phosphoryl transfer from ATP to IP5.[70]

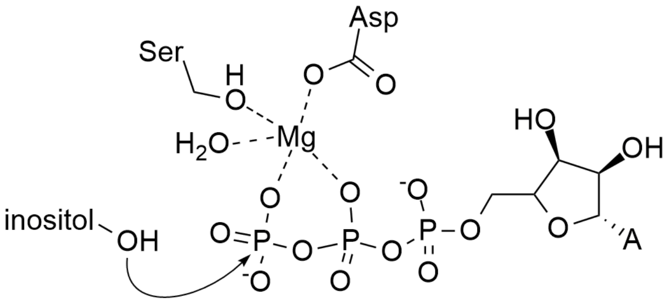

Ion coordination and kinase activity templated on the structure 2XAN.

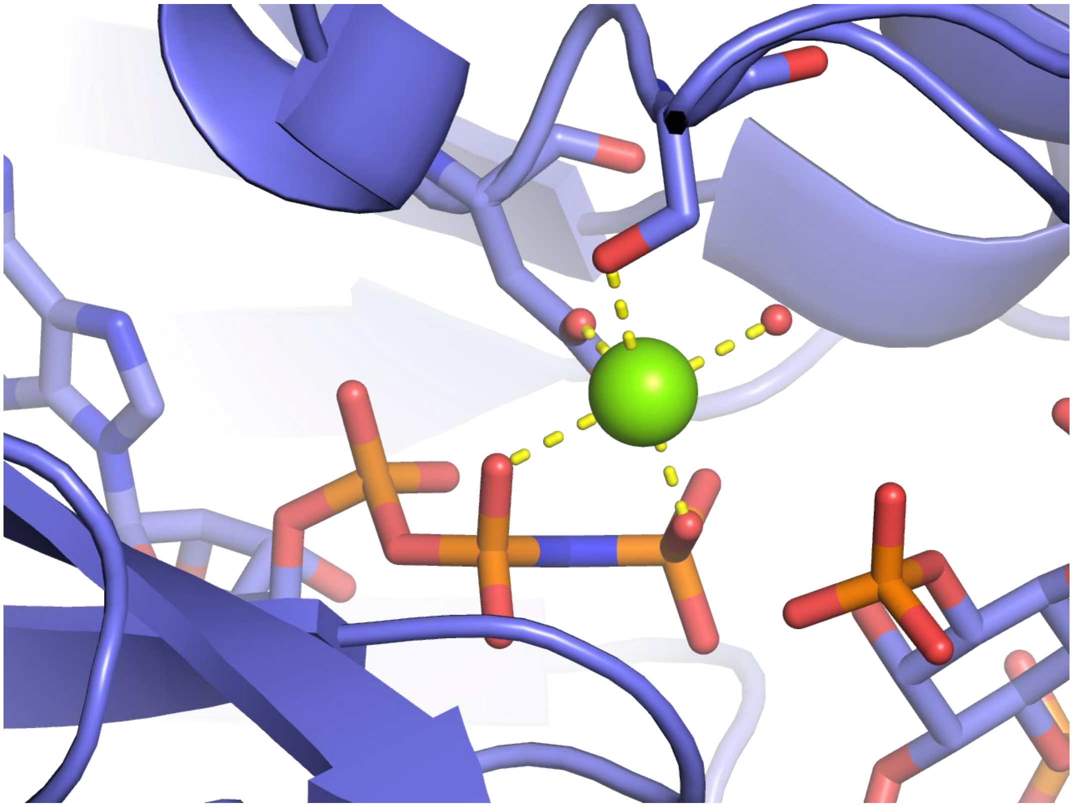

Incomplete ion coordination in the representative structure 2XAN.

YcaO-related McrA-glycine thioamidation protein

EC from sequence only: 6.2.2.3

This category is defined on the InterPro family level (IPR017667 YcaO-related McrA-glycine thioamidation protein) extended by homologous YcaO structures. There is no associated EC to this family, nor to the YcaO structures. However, for EC 6.2.2.3, there are 4 protein sequence matches in UniProt (A0ABV4ESY2, A0ABV2RYJ2, A0ABU0Q3X9, A0ABU0RZN3) that all are associated with the corresponding PFAM entry PF02624 (YcaO cyclodehydratase, ATP-ad Mg2+-binding). The proteins in this family are universal in and restricted to methanogenic archaea[71] and share homology with YcaO (ribosomal protein S12 methylthiotransferase) and its homologues involved in ATP-dependent formation of heterocycles in thiazole/oxazole-modified peptide antibiotics.[72] Methyl-coenzyme M reductase (MCR) is an essential enzyme found strictly in methanogenic and methanotrophic archaea that catalyzes a reversible reaction involved in the production and consumption of the potent greenhouse gas methane.[73] In the corresponding active site, one Mg^{2+} ion is coordinated by the β and γ phosphates, as well as the carboxyl group of a glutamate residue, and an additional three crystal waters as found in the selected representative structure (PDB 6CI7).

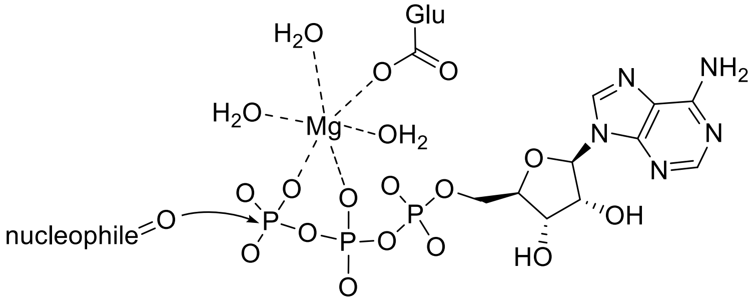

YcaO catalyzed phosphorylation according to Ref. [72].

YcaO-family cyclodehydratases, including LynD, catalyze an ATP-dependent heterocyclisation of peptide substrates and are predicted to require Mg^{2+} as the obligate counter-ion for the MgATP co-substrate; conserved active-site residues coordinate the nucleotide triphosphate moiety, and the metal ion is presumed to position the polyphosphate chain and activate the backbone carbonyl oxygen for nucleophilic ring closure.[74]

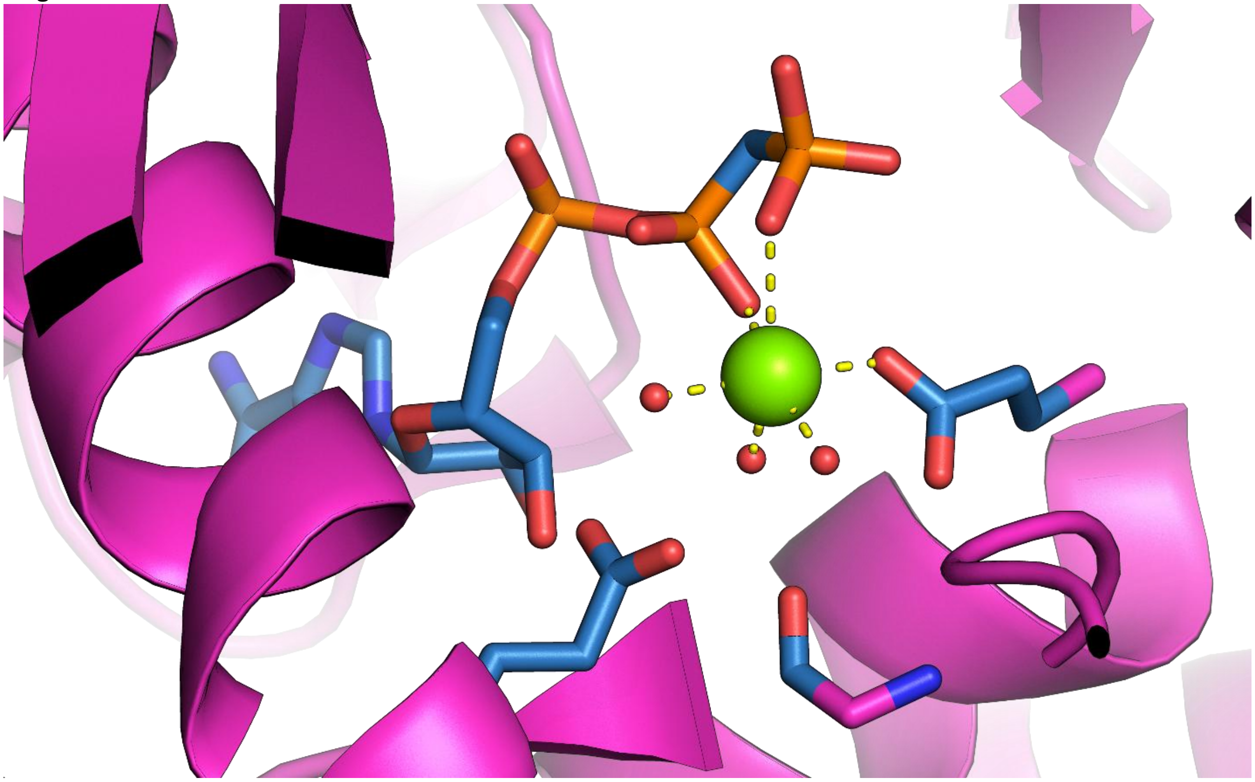

ATP and ion coordination in the representative structure 6CI7.

Peptidase G2, IMC autoproteolytic cleavage domain

There are 2 PDBs originating from the same publication we identified for this category, which is defined on the InterPro domain level (IPR021865 Peptidase G2, IMC autoproteolytic cleavage domain). There is no associated EC to this domain or to the structures of the pre-mature bacteriophage phi29 gene product 12. Yet, the authors describe that the autocleavage of the C-terminal domain is a post-trimerization event that is followed by a unique ATP-dependent release. In the active site, there are two glutamic acid residues that may be involved in the ATP hydrolysis.[75] Yet, based on the available two structures, a clear coordination could not be established. We are inclined to associate a BG(–) coordination. The PDB 3sucA structure displays an αβγ-coordination, yet the coordination sphere around the Mg^{2+} is not complete. In PDB 3gqkA, it is slightly shifted towards the γ phosphate with a βγ-coordination, and the two glutamate residues approach the coordination sphere. However, their distance for the coordination is not optimal and the coordination sphere is still not complete.

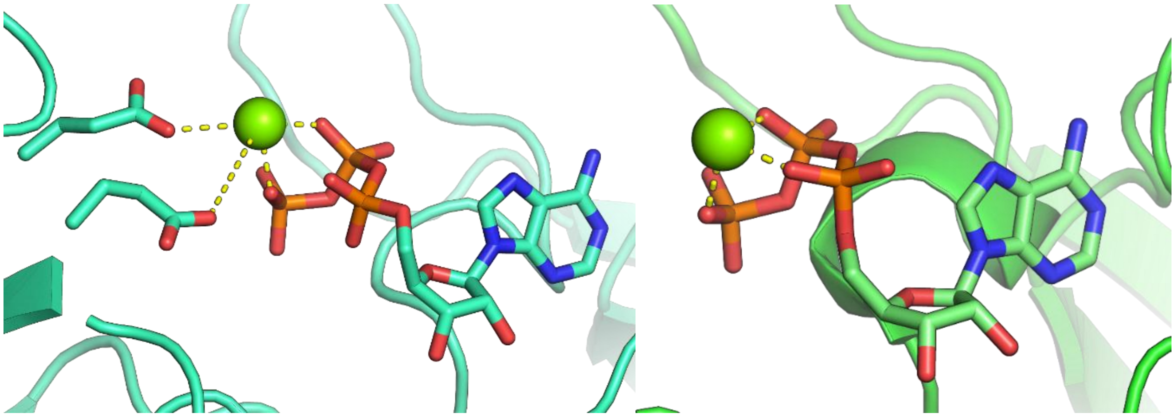

ATP-ion site in structures 3GQK (left) and 3SUC (right).

Ribokinase-like

The Ribokinase-like active site contains a second metal ion in αβ coordination in addition to the pinching cation.[76-78]

Ribokinase requires ATP and Mg^{2+} for activity; no activity is observed upon addition of EDTA, and two Mg^{2+} ions in the active site coordinate ATP and the ribose substrate.[79]

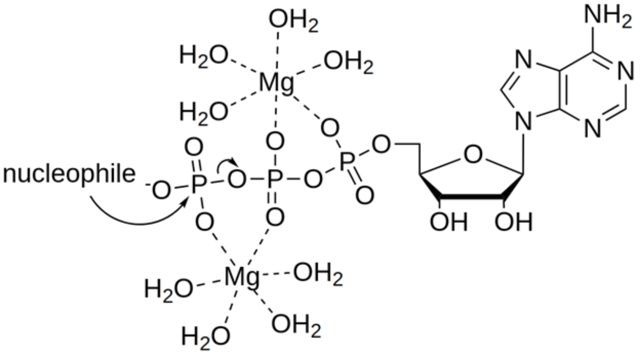

Typical coordination and the reaction facilitated by the Ribokinase-like superfamily.

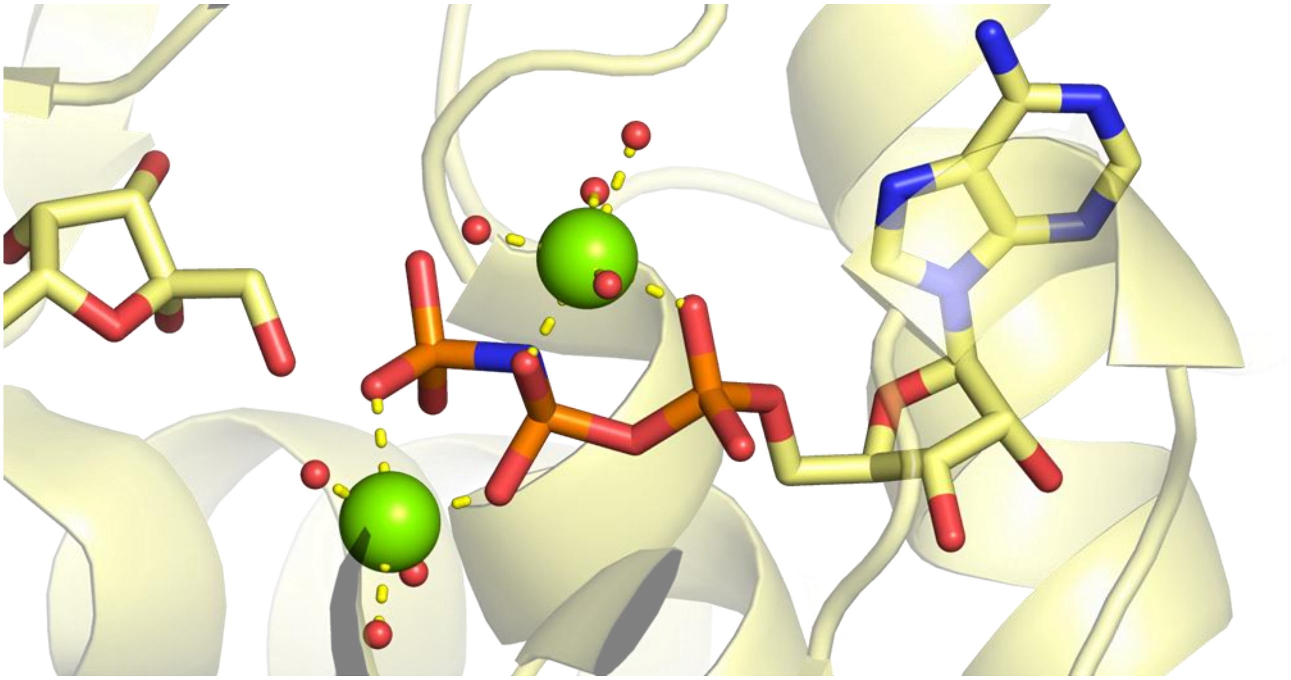

ATP and substrate binding represented by 2JG1.

DhaL-like

A set of specific kinases are assigned to the same *DhaL-like* superfamily, the representative structure contains a bound ATP analogue with poorly defined two-metal-ion coordination. Both ions remain in the ADP state, we assign αβ and βγ coordination only tentatively.

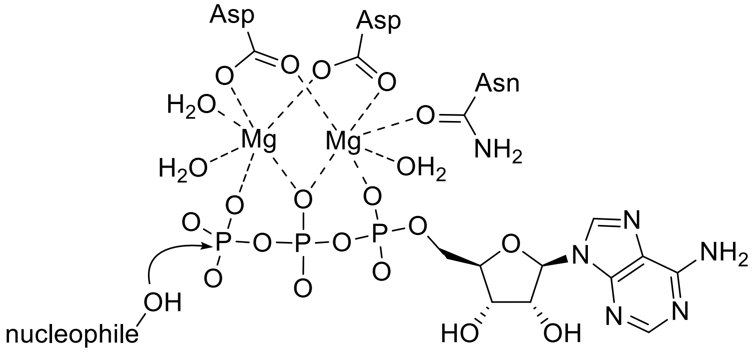

Proposed coordination scheme and catalyzed reaction of DhaL-like kinases.

Dihydroxyacetone kinases require Mg^{2+} for phosphoryl transfer activity; EDTA strips the tightly bound nucleotide cofactor of the DhaL subunit and destabilises the protein, and steady-state kinetic analysis confirms MgATP as the catalytic substrate (Garcia-Alles et al. 2004; Bächler et al. 2005).[80-81]

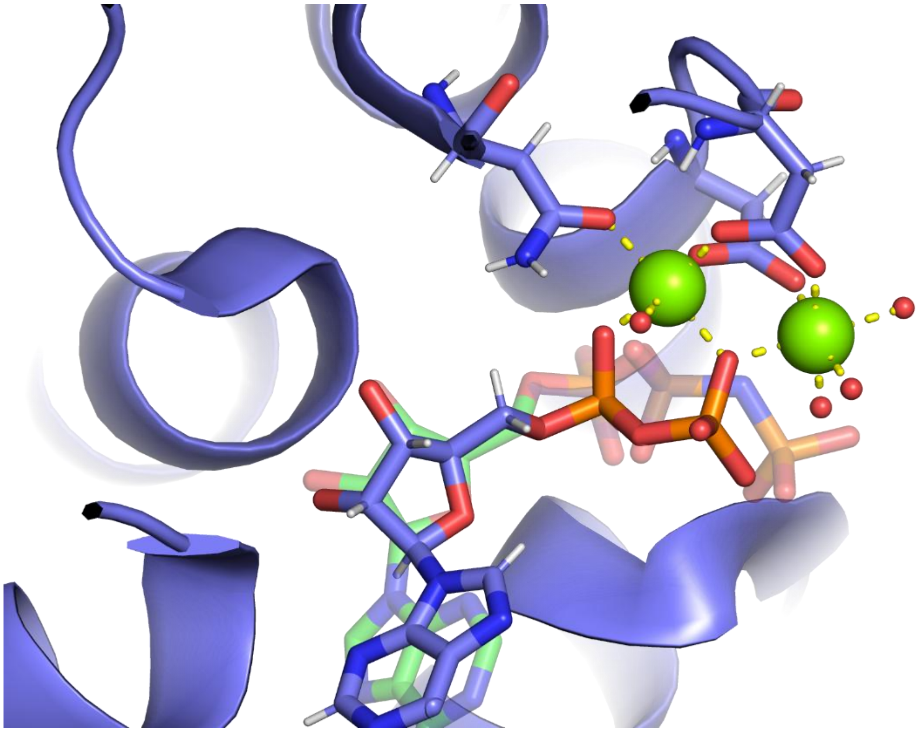

Metal ion coordination in the representative structure 7RM7 with ADP, ATP analog overlay is depicted from 1UN9 in green.

Calcium-dependent phosphotriesterase

Within this superfamily, we have identified an InterPro family (IPR008557 Alkaline phosphatase PhoX), the corresponding structures of which can cleave ATP,[82] while the rest of the superfamily does not process NTP, but these are phosphatases hydrolyzing phosphate ester ligands (EC 3.1.3.1). In the selected

representative structure (PDB 4AMF), a nonhydrolyzable analog (AMP-PCP) of the substrate ATP is present. The enzyme contains a complex active-site cofactor comprising two antiferromagnetically coupled ferric iron ions (Fe^{3+}), three calcium ions (Ca^{2+}), and an oxo group bridging three of the metal ions.[82] A Ca^{2+} is on each side of the triphosphate chain, both in a βγ-coordinated position. Each is further coordinated by one glutamate residue, one aspartate residue, and crystallographic waters. In the proposed mechanism, the phosphate cleavage is initially carried out by the iron oxide moiety, which is recovered by using a water from the calcium coordination sphere to form the inorganic phosphate.

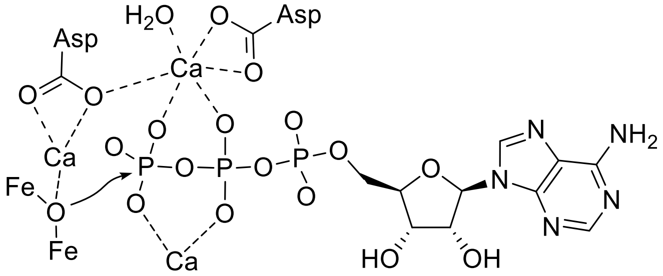

While the enzymes that possess phosphotriesterase activity are widely varied and utilize completely different protein scaffolds and even different catalytic mechanisms, they are all metal dependent hydrolases.[83]

Proposed phosphatase mechanisms in PhoX.[82] Many coordinating residues are omitted for clarity.

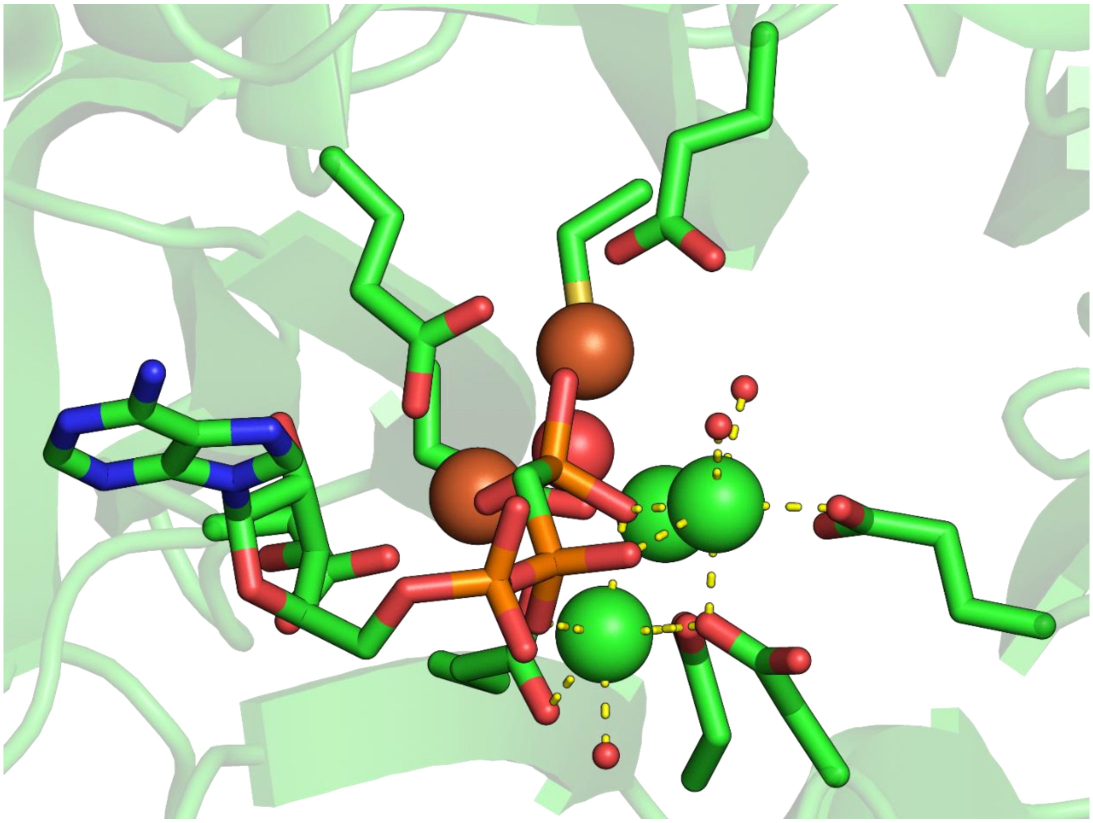

ATP and metal ion coordination in the representative structure 4AMF.

In the following superfamilies, ions feature in BG() position alongside an additional AG ion bridging the α and γ phosphates (Figure 5 in the main text).

Protein kinase-like (PK-like)

There is a variation in the ion coordination within the PK-like superfamily, there are structures missing one ion or the other from the two established positions. In addition, occasional examples are also identified with slightly different active site ion placement, despite being restricted to high resolution structures.

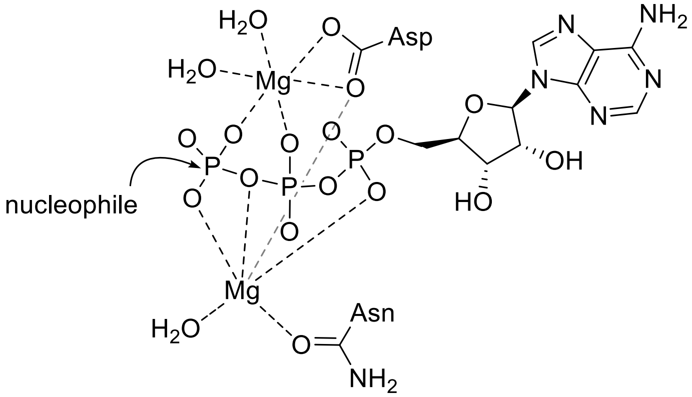

Mg^{2+} assists substrate binding and product release in protein kinase A; magnesium is an essential activator of kinase activity, with the second Mg^{2+} site modulating the rate-limiting conformational change.[84] Kinase reactivity and coordination amongst the members of the PK-like superfamily.

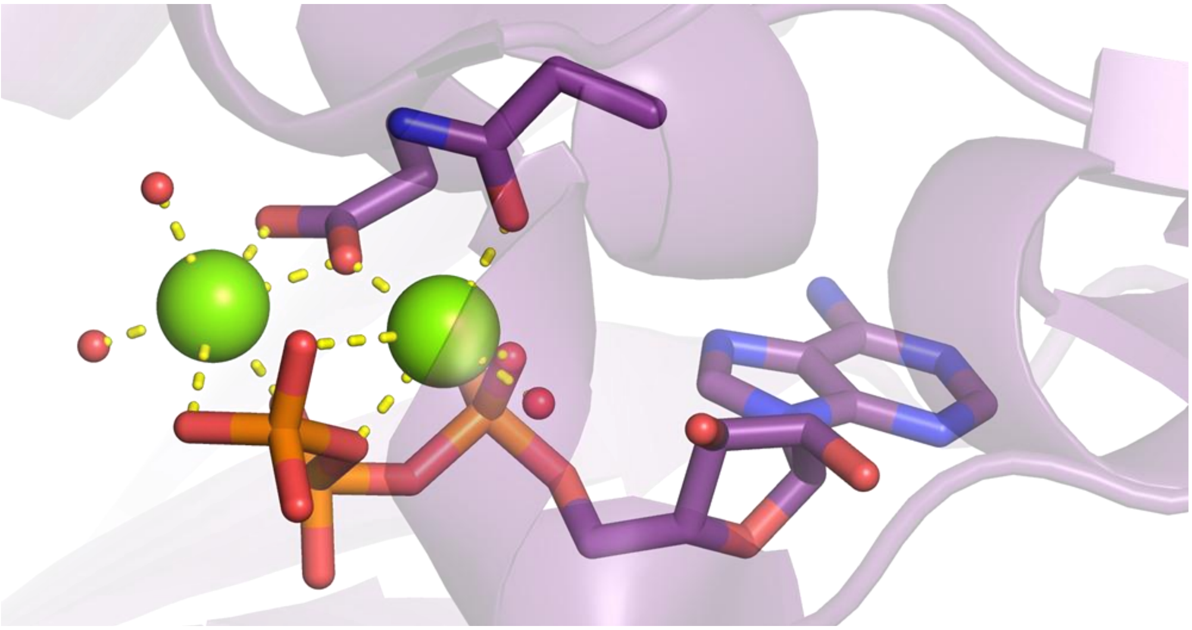

ATP and metal ion binding in the representative structure 3DNT.

Glutathione synthetase ATP-binding domain-like

There is a good agreement in the coordination of the two magnesium ions in the entire superfamily. The secondary structure around the active site is similar to the SAICAR synthase-like superfamily. The overall architecture is similar to the PK-like superfamily, this superfamily mostly covers ligases creating peptide modes, therefore the phosphate cleavage happens with a carboxylate nucleophile.

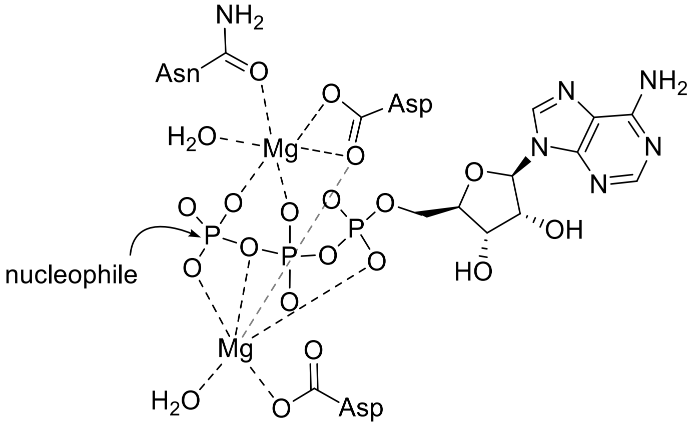

E. coli glutathione synthetase activity strictly depends on divalent cations — Mg^{2+} > Mn^{2+} > Co^{2+} — while Ca^{2+}, Zn^{2+} and Cu^{2+} do not support activity; EDTA inhibition confirms essential metal dependence.[85]
Typical coordination and the reaction catalyzed by the members of Glutathione synthetase ATP-binding domain-like superfamily.

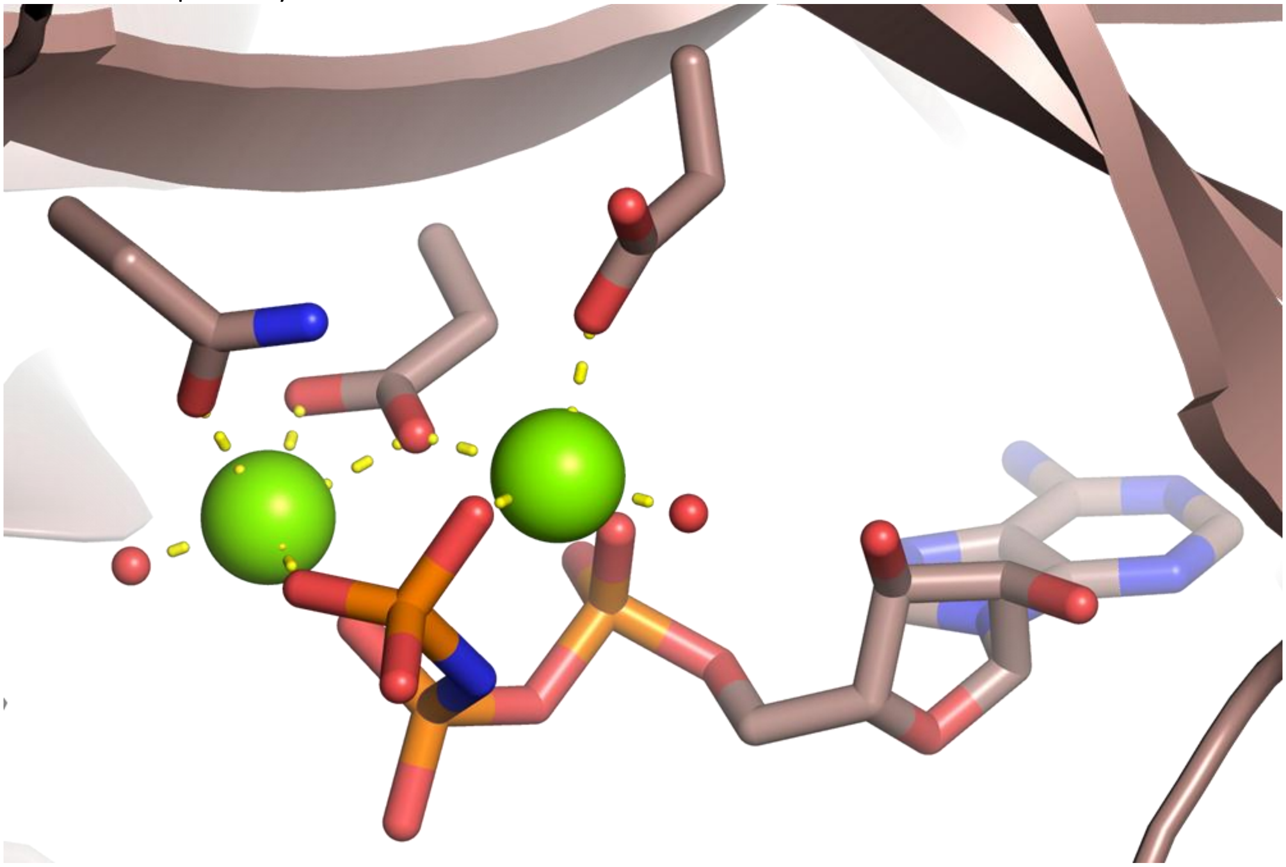

ATP and metal ion binding in the representative structure 5DGH.

SAICAR synthase-like

As mentioned above, both the fold and the coordination is quite similar to the Glutathione synthetase ATP-binding domain-like superfamily, some of the proteins perform the same reaction according to the EC assignments, while mostly SAICAR synthase-like members are involved in phosphorylating inositol derivatives.

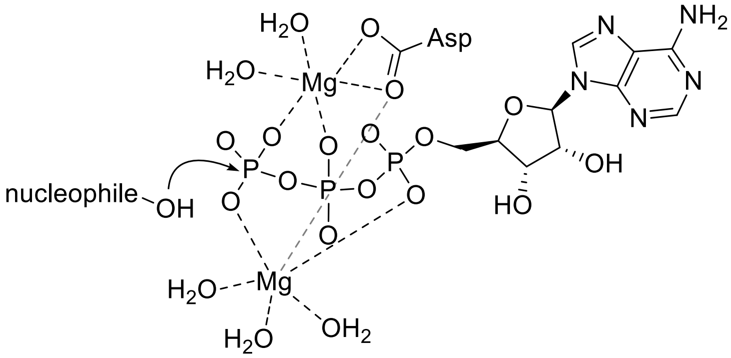

SAICAR synthase superfamily members are thought to require divalent metal ions for catalytic activity, consistent with the typical two-metal-ion mechanism of related ATP-dependent ligases.[86]

Typical coordination and the reaction catalyzed by the members of SAICAR synthase-like superfamily.

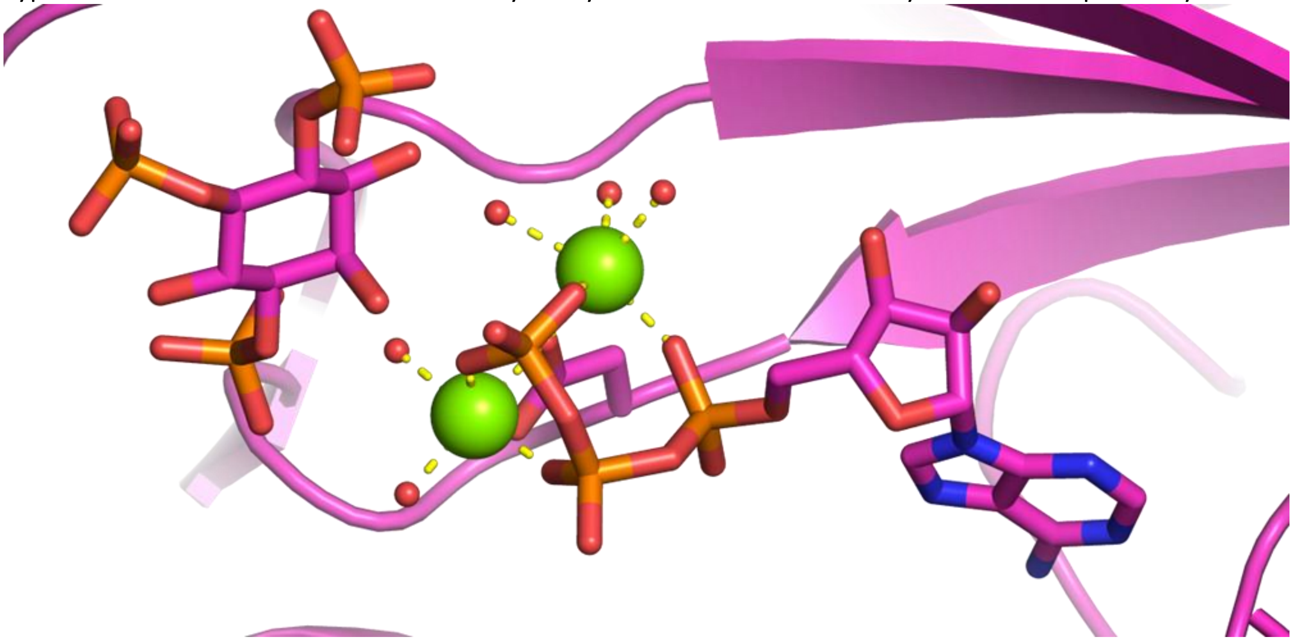

Substrate and metal ion binding in the representative structure 2AQX.

Diacylglycerol kinase (DgkA)-like

While many structures are categorized into the *NAD kinase/diacylglycerol kinase-like* superfamily, there are two ATP bound membrane proteins, which contain zinc ions and are not assigned by SUPFAM. While magnesium is a common ATP counter-ion in kinases, zinc can serve this role in DgkA.[87] A single sequence hit for EC 2.7.1.10 in UniProt (A0A8S0X7Q2) matches the PFAM PF01219 (Prokaryotic diacylglycerol kinase) family.

Undecaprenol kinase (UppK) strictly requires Mg^{2+} for phosphotransfer activity, and elimination of Mg^{2+} completely abolishes catalysis; beyond MgATP coordination, a separate enzyme-bound Mg^{2+} site is essential, and mutagenesis of the conserved aspartate residues coordinating this second metal ion markedly reduces activity. [88]

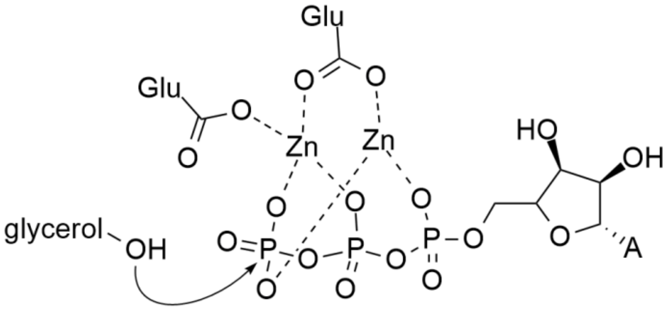

Zinc coordination and glycerol kinase reactivity scheme for the DgkA-like superfamily.

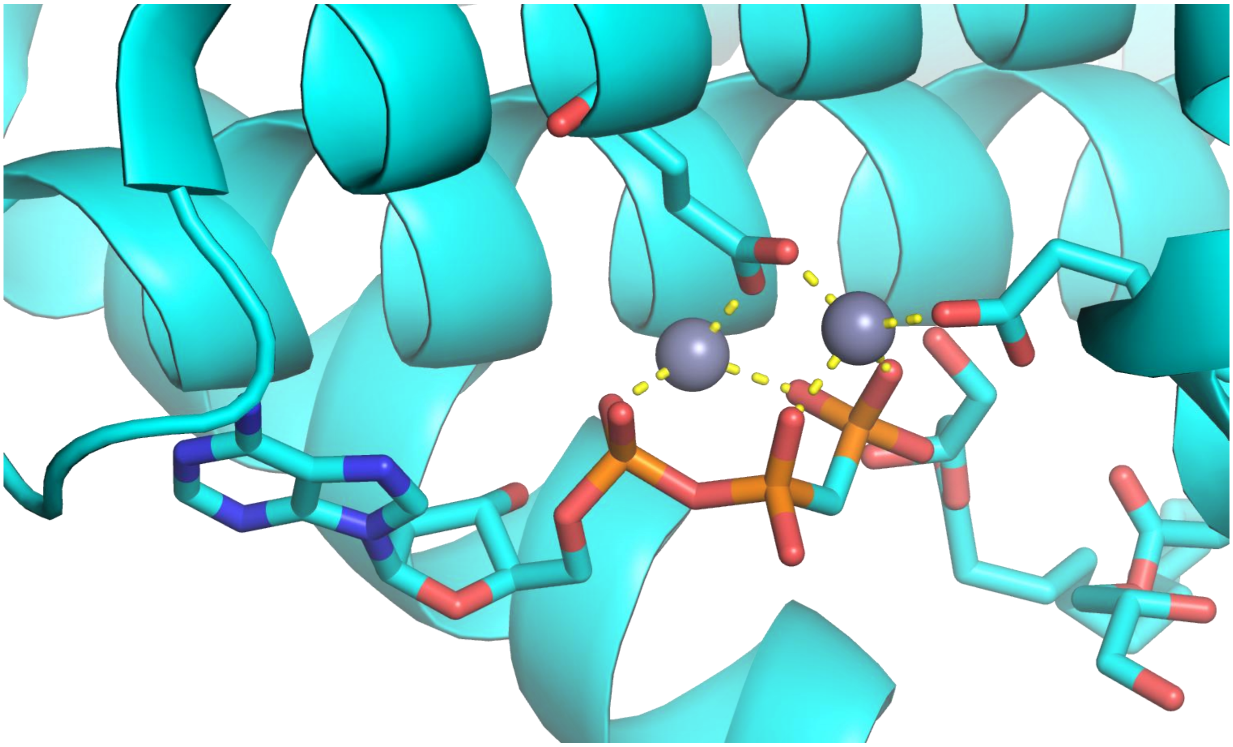

Zinc and ATP binding in the selected DgkA kinase 4UXX.

Metallo-dependent phosphatases

In the active site, there are two metal ions, usually manganese, iron, or zinc, both coordinated by a cage of histidine, aspartate, and asparagine residues.[89] They are general phosphatases, but may also work on NTPs.

In calcineurin (PP2B), a prototypical member of the Metallo-dependent phosphatases superfamily, Mg^{2+} serves as an obligate catalytic cofactor for phosphoserine/phosphothreonine hydrolysis: Mg^{2+}, Mn^{2+}, Co^{2+}, or Ni^{2+} can support catalysis, but Zn^{2+} cannot; Mg^{2+} acts solely at the catalytic step with no effect on Ca^{2+} or calmodulin binding.[90]

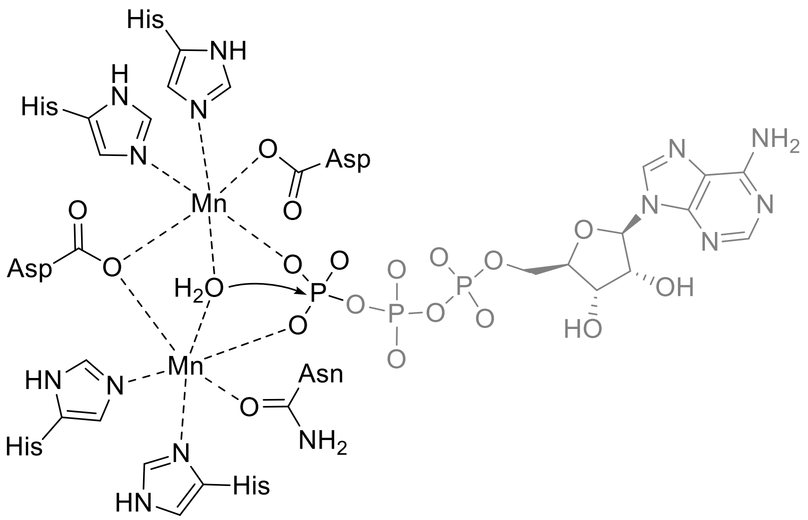

Ion coordination and phosphatase reactivity based on the structure 4LAC.

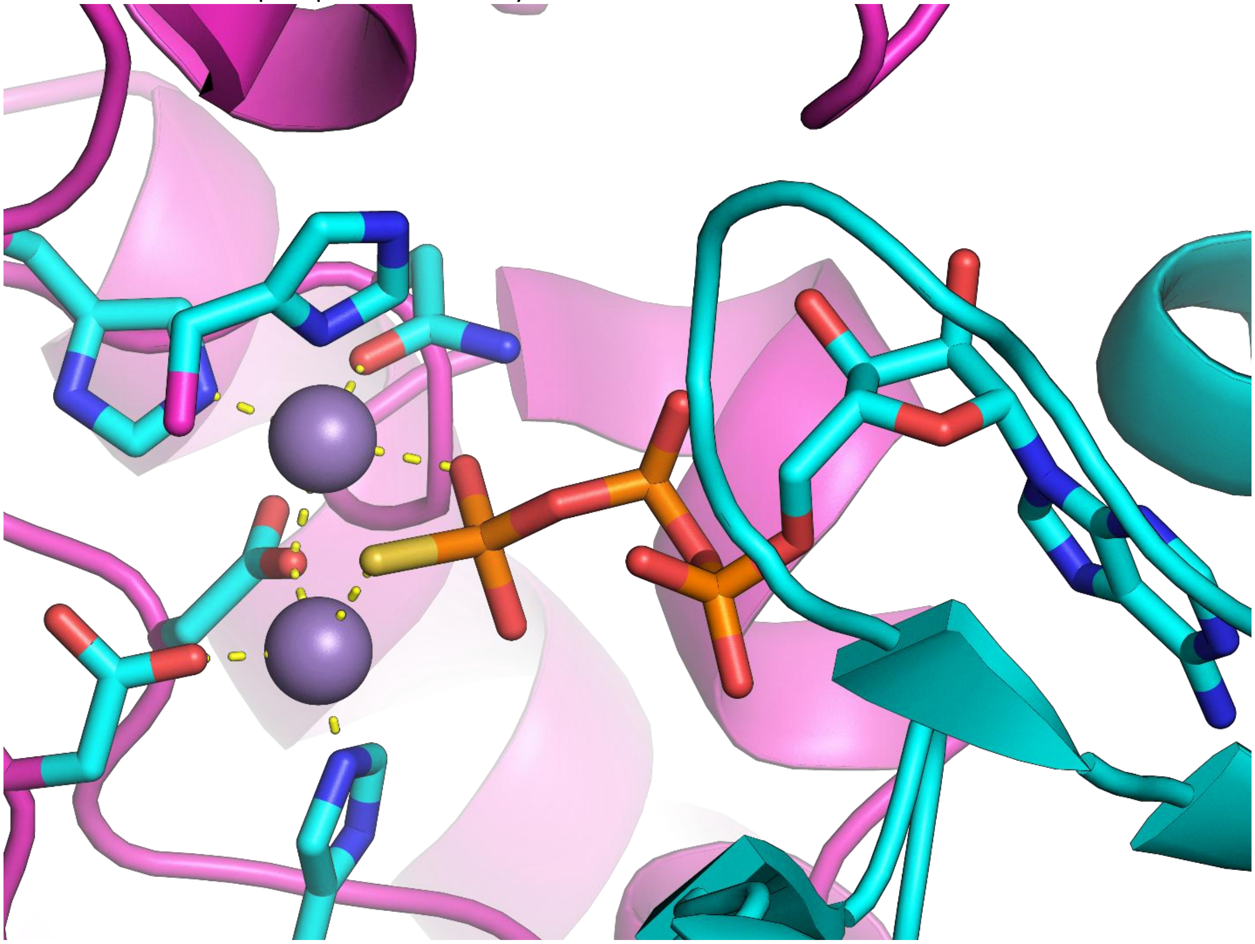

Ion and NTP coordination in the structure 4LAC.

11. Pyrophosphatase superfamilies

Pyrophosphatases with αβγ coordination on the (+) side

PRTase-like

Enzymes belonging to EC 2.7.6.1 are phosphoribosyl pyrophosphate synthetases (PRPS), and are key regulators of nucleotide metabolism.[91] For the synthesis of phosphoribosyl pyrophosphate they use ATP. As co-factor, an αβγ-coordinated Mg^{2+} ion is present at the active site. Interestingly, a histidine residue also participates in the Mg^{2+}-coordination in this superfamily. Two further water molecules also coordinate the Mg^{2+}.

Divalent Mg^{2+} is required for catalytic activity of orotate phosphoribosyltransferase, with maximal turnover achieved at 2 mM $MgCl_2$; spectroscopic studies indicate that the metal cofactor coordinates the PRPP co-substrate rather than the orotate substrate. [92]

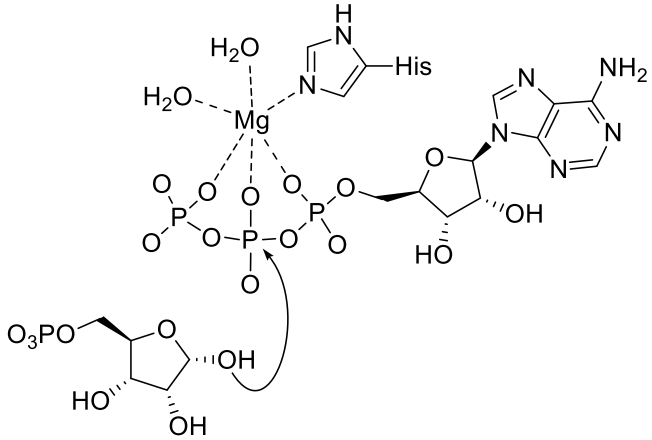

Pyrophosphorylation of ribose 5’-phopsphate in the 1’ position suggested for EC 2.7.6.1

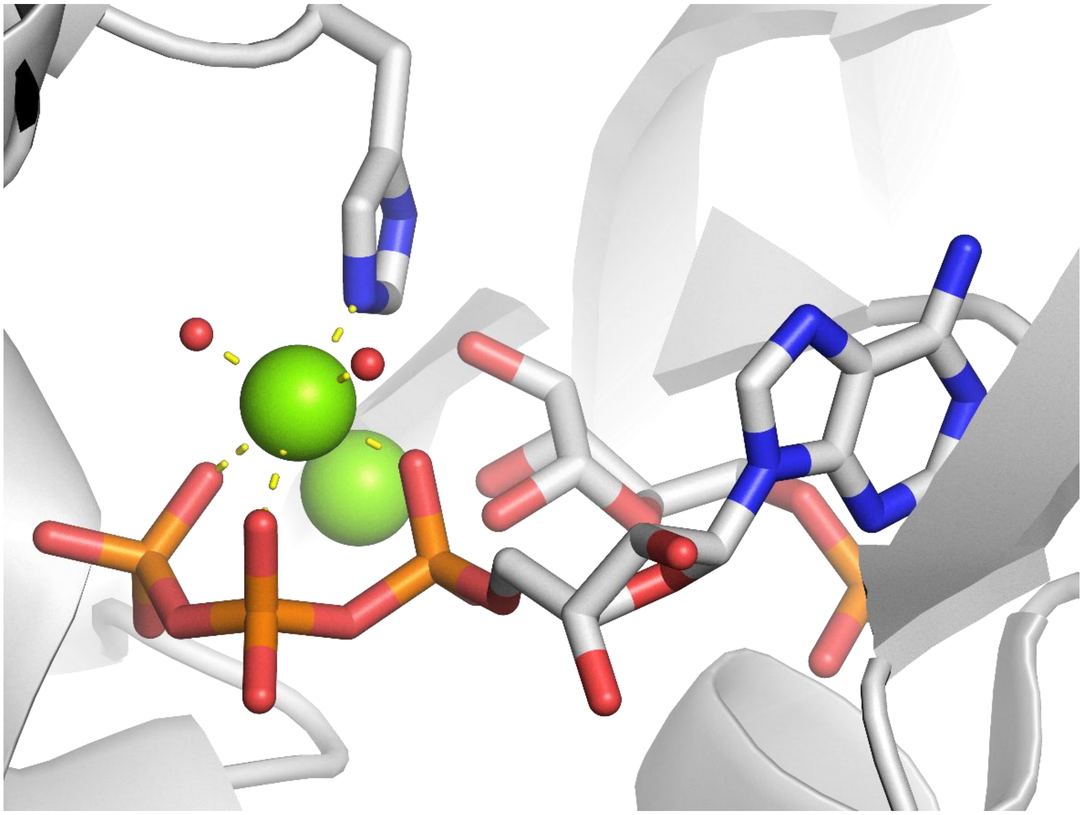

ATP and metal ion coordination in the representative structure 8DBJ.

All-alpha NTP pyrophosphatases

This fold has 2 catalytic metal ions. One Mg^{2+} ion is coordinated by the αβγ phosphate oxygens, while the other Mg^{2+} ion by only a β phosphate oxygen atom. Two aspartate and two glutamate residues are also involved in the coordination of the two Mg^{2+} ions.

Mycobacterial MazG, a nucleoside triphosphate pyrophosphohydrolase, strictly requires a divalent cation for hydrolysis of all canonical NTPs to NMPs and pyrophosphate; Mg^{2+} is the preferred cofactor, Mn^{2+} partially substitutes, and chelation with EDTA abolishes activity entirely.[93]

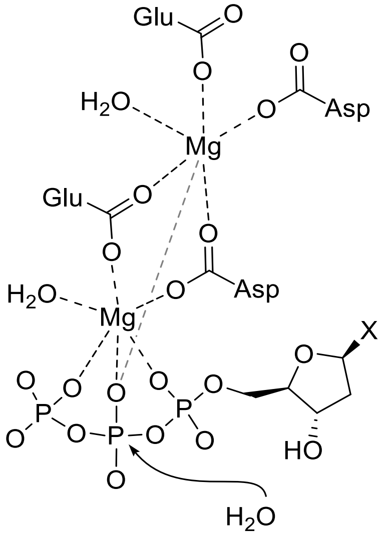

Metal ion coordination and pyrophosphatase reaction based on the dCTPase structure 6SQZ. While the assigned PDB structures are clearly pyrophosphatases, many sequences from this superfamily are linked to the EC 3.6.1.8, which has both phosphatase and pyrophosphatase activity associated and considered ambiguous.

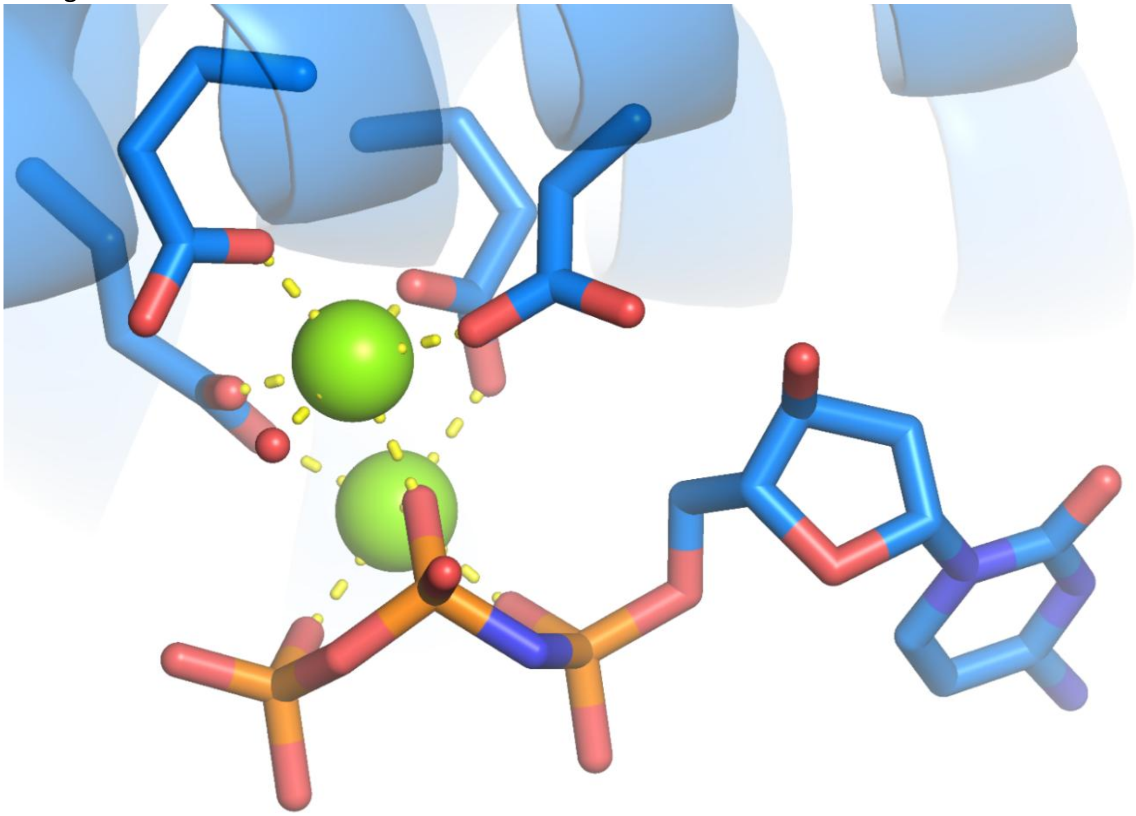

Metal ion and dCTP binding in the representative structure 6SQZ.

Adenine nucleotide alpha hydrolases-like

This superfamily includes stress-response proteins that do not necessarily catalyze ATP hydrolysis. The group of stress proteins binds a single Mg^{2+} ion which is octahedrally coordinated by the αβγ phosphate oxygen atoms and three additional water molecules.[94-95] The rest of the members are enzymes forming carbon-nitrogen bonds and have 2 metal ions. One Mg^{2+} ion is coordinated by the αβγ phosphates, whereas the other Mg^{2+} ion shows an αγ phosphate coordination. Two aspartate residues are also involved in the coordination of the Mg^{2+} ion at the αβγ position.

Adenylate kinase can be activated by direct binding of a Mg^{2+} ion to the enzyme in addition to ATP-complexed Mg^{2+}; Mg^{2+}-activated AK exhibits a 23 ± 3-fold increase in the forward reaction rate compared with the unactivated form.[96]

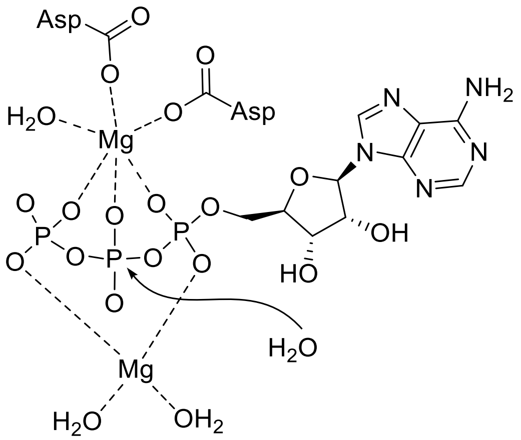

Mg^{2+} coordination and ATP pyrophosphatase reactivity. This superfamily has members from many AMP forming EC categories.

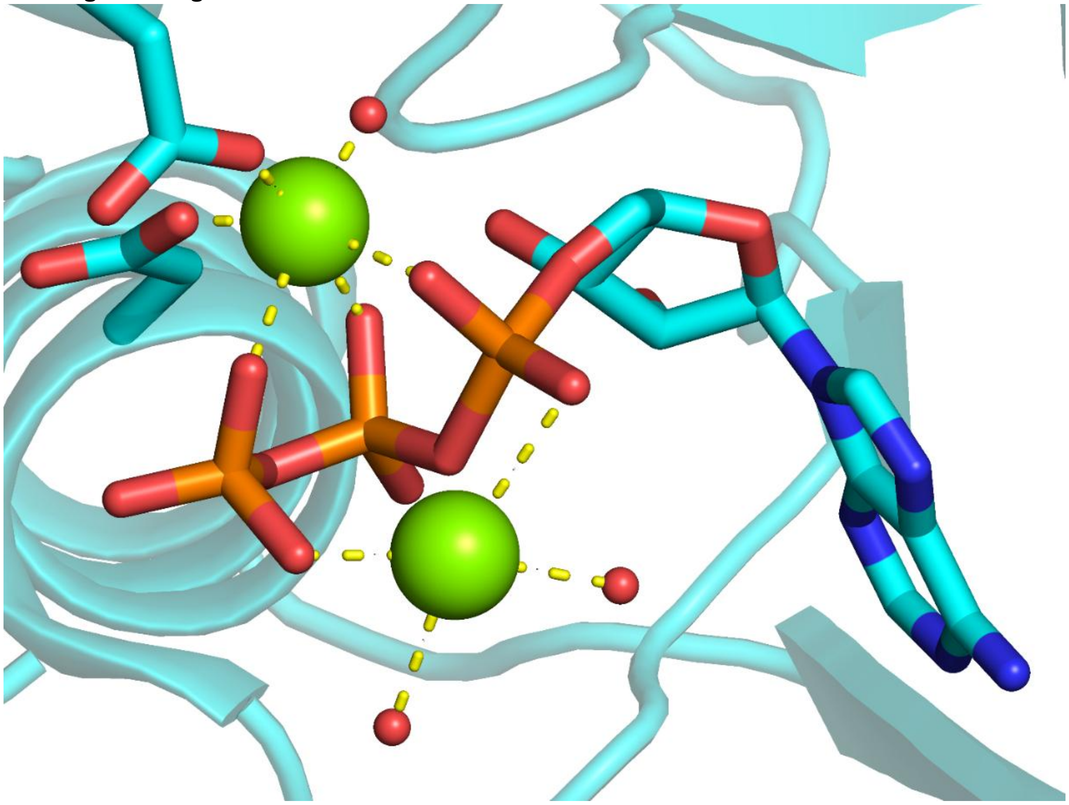

ATP and metal ion coordination in the representative structure 1MB9.

Nucleotide-diphospho-sugar transferases

Some of the UDP-N-acetylglucosamine diphosphorylases (EC 2.7.7.23) of this fold also function as glucosamine-1-phosphate N-acetyltransferases (EC 2.3.1.157). One Mg^{2+} ion has a clear αβγ phosphate coordination. A second Mg^{2+} ion is present close to the α phosphate in some of the structures. However, its role could be related to the correct positioning of the nucleotide and promoting the attack of the ligand hydroxyl group, it may not be directly involved in the enzymatic reaction. The Mg^{2+} ion which is

coordinated by an α-phosphoryl oxygen, is further coordinated by the side chains of two aspartate residues in its vicinity.
UDP-glucose pyrophosphorylase (UGPase) requires MgUTP as its true substrate, and free Mg^{2+} additionally acts as a cofactor in the pyrophosphorolysis direction with a Km of approximately 0.13 mM; although Mn^{2+} and Co^{2+} can partially substitute for Mg^{2+}, they support only reduced activity, confirming that Mg^{2+} is the preferred divalent cofactor. [97]

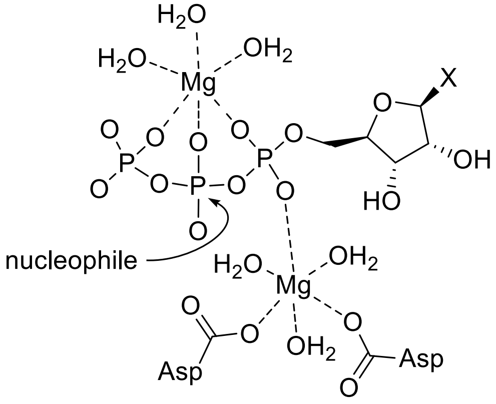

Nucleotide-diphospho-sugar transferases are assigned to many nucleotidyltransferase EC (2.7.7.-) and consequently thought to work with multiple NTPs and nucleophiles.

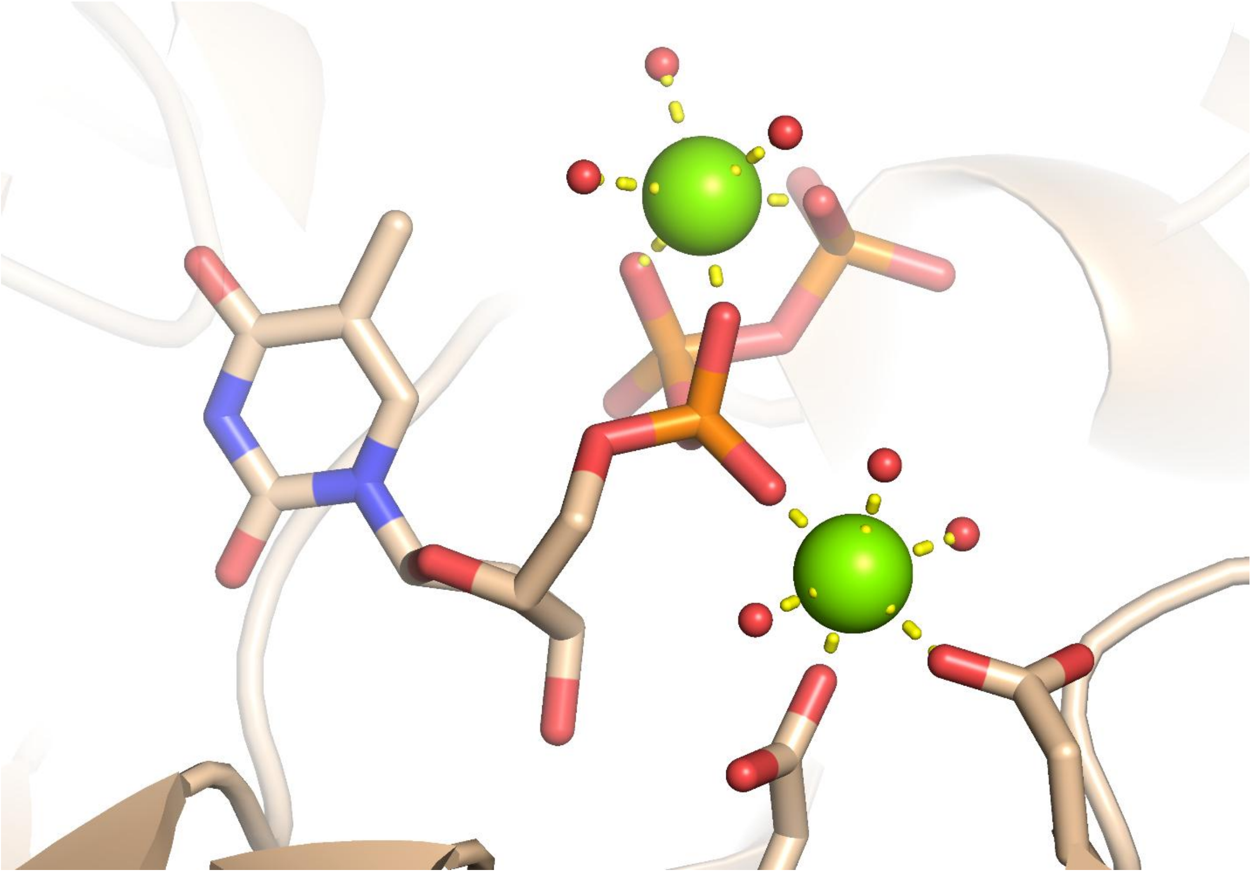

Metal ion and NTP coordination in the representative structure 6B5K.

Activating enzymes of the ubiquitin-like proteins

This superfamily has two metal ions. One Mg^{2+} ion is coordinated by the αβγ phosphate oxygens, and another one by only a β phosphate oxygen atom. Both Mg^{2+} ions are further coordinated by a conserved aspartate residue each, and the Mg^{2+} ion in the β position is further stabilized by a conserved glutamate residue through coordinating water molecules.

Asp576 of human UBA1 coordinates the Mg^{2+} ion interacting with the α- and β-phosphate oxygens of ATP; Asp576 mutations dramatically impair ATP·Mg^{2+} binding affinity and shift substrate binding mechanism, abolishing ordered kinetics of ubiquitin adenylation.[98]

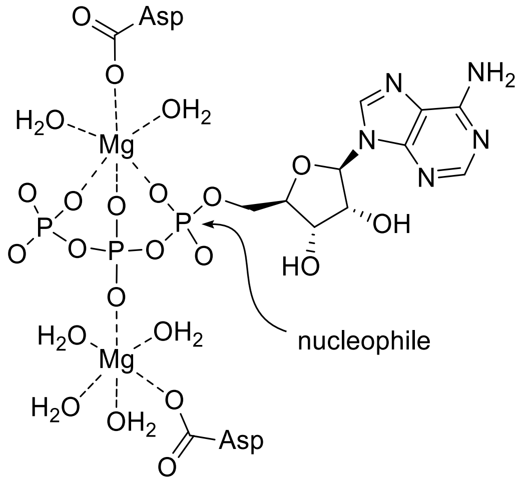

Adenyltransferase reactivity and metal ion coordination.

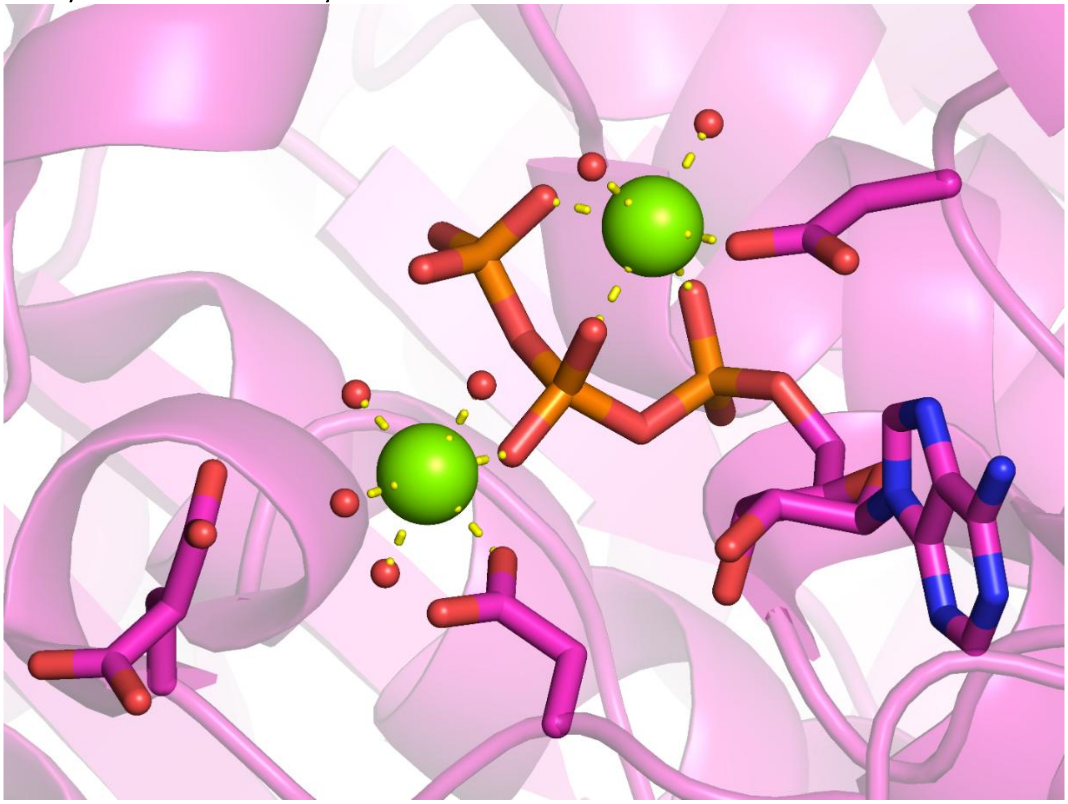

ATP and metal ion coordination in the representative structure 6H77.

Phosphopantoate/pantothenate synthetase superfamily

There is no corresponding SUPFAM superfamily, this category is named after the InterPro superfamily (IPR038138). The enzymes correspond to 4-phosphopantoate-beta-alanine ligases, also known as phosphopantothenate synthetases (EC 6.3.2.36), catalyzing the conversion of (R)-4-phosphopantoate and beta-alanine to 4'-phosphopantothenate in a two-step reaction, first of which is releasing a pyrophosphate[99] and it is a part of the CoA biosynthesis pathway.[100] The selected representative structure (PDB 3WDL) displays an αβγ-coordinated Mg^{2+} at the active site, which is further coordinated by an aspartate residue and a crystal water.

Comprehensive kinetic analysis of M. tuberculosis PanC establishes a Bi Uni Uni Bi Ping-Pong mechanism; KM(ATP)=2.6 mM, kcat=3.4 s^{-1}; pantoyl-adenylate intermediate confirmed as competent; two Mg^{2+} ions coordinate ATP phosphates and the catalytic Asp residues, required for adenylation of pantoate.[101]

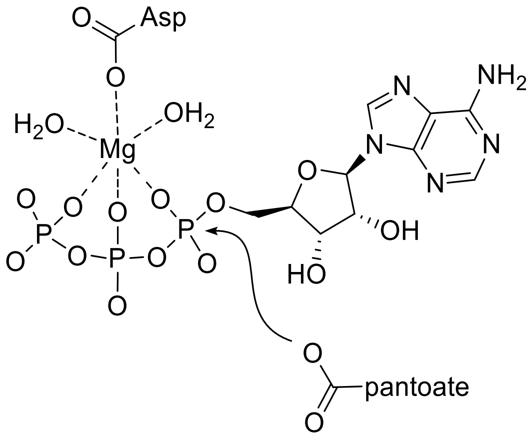

Pantoate carboxyl activation[99] and schematic Mg^{2+} coordination.

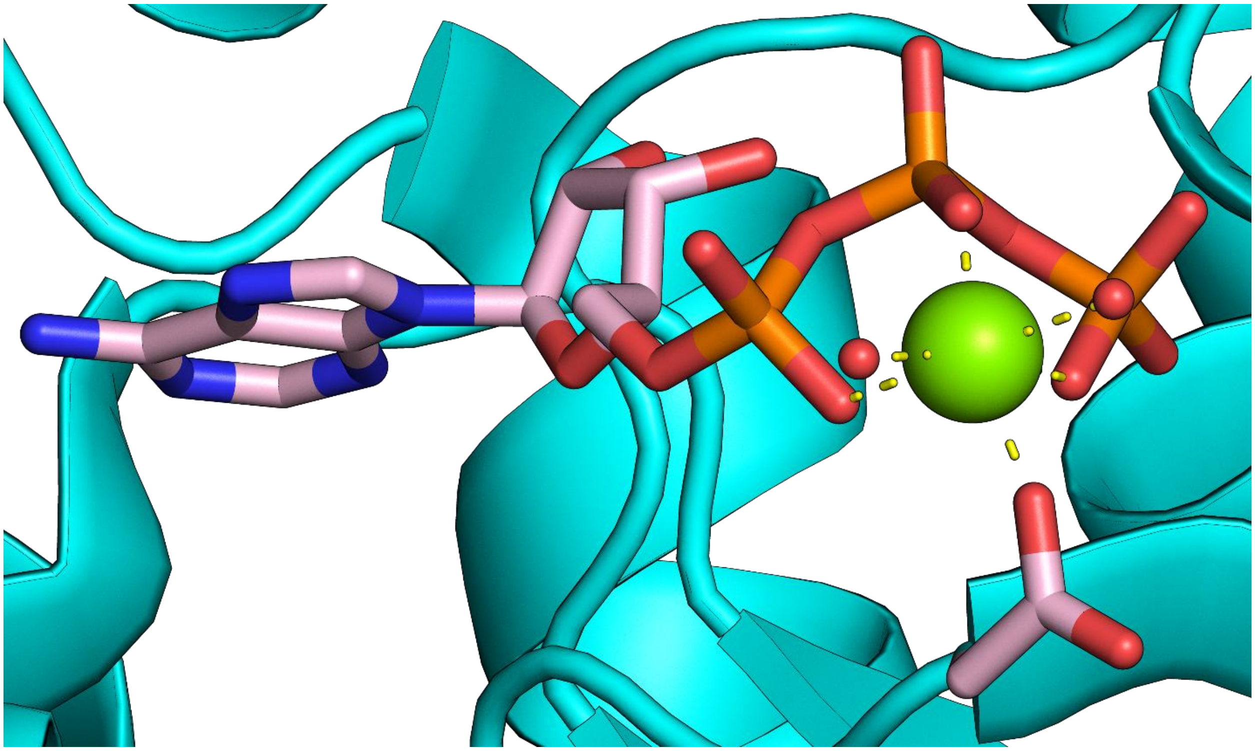

ATP and metal ion coordination in the representative structure 3WDL.

Virion DNA-directed RNA polymerase domain

There is no corresponding SUPFAM superfamily entry, this category is defined on the InterPro domain level (IPR049432). This domain is found in Virion DNA-directed RNA polymerase from Bacteriophage N4 (vRNAP, Q859P9), responsible for the transcription of the early region of the double-stranded linear DNA genome of the lytic coliphage.[102] It displays two metal ions at the active site in a typical polymerase configuration corresponding to the DNA-directed RNA polymerase function; a Mg^{2+} is coordinated by all three phosphate groups, and another is coordinated by the 3′ hydroxyl of the priming nucleotide and the α-phosphate of the incoming NTP. We note that while SUPFAM was not able to categorize the fold for this protein, ECOD assigns *Adenylyl and guanylyl cyclase catalytic domain-like*, matching the *DNA/RNA polymerase* SF. Enzymes with a modified C-terminal carboxy group exhibit a Mg^{2+}-dependent decrease in catalytic activity; the C-terminus is positioned close to the active site where Mg^{2+} ions are essential for catalysis.[103]

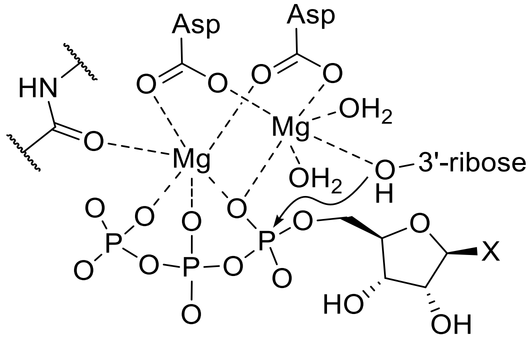

Polymerase reactivity (EC 2.7.7.6) and schematic metal coordination.

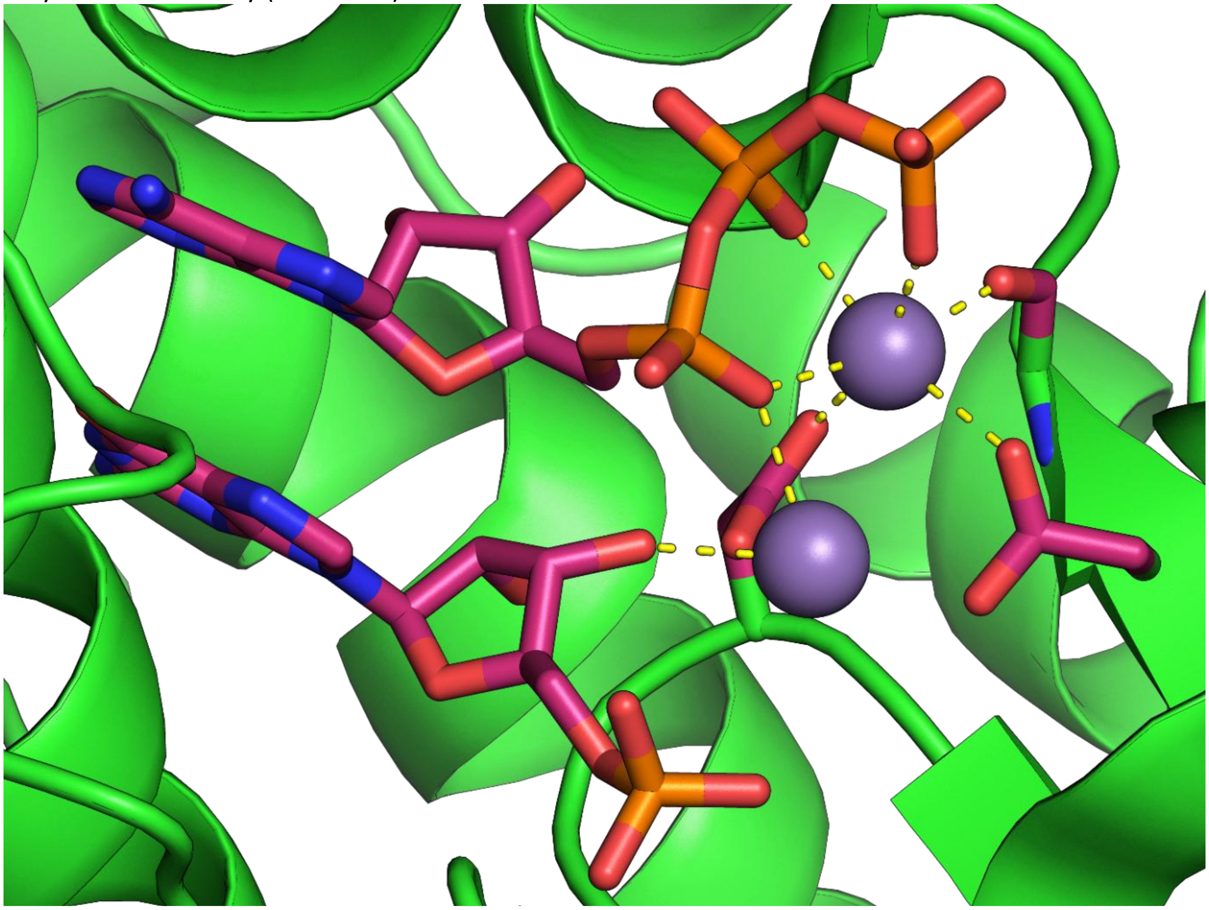

NTP, nucleophile and Mn^{2+} ion coordination in the representative structure 4FF3.

Pyrophosphatases with αβ coordination on the (+) side

6-hydroxymethyl-7,8-dihydropterin pyrophosphokinase, HPPK

EC: 2.7.6.3 (2-amino-4-hydroxy-6-hydroxymethyldihydropteridine diphosphokinases)

This superfamily has 2 Mg ions, one coordinating the αβ and another one coordinating the βγ phosphates. In some cases the Mg^{2+} ion with βγ coordination also coordinates the attacking hydroxyl group of the ligand. There are two aspartate residues on the nearby beta-sheet that both coordinate the two Mg ions. Fluorometric binding assay (anthraniloyl-ATP) directly measures MgATP affinity: Kd(MgATP) = 2.6 ± 0.06 µM; ATP affinity is 15-fold higher in the presence of Mg^{2+} than without; two Mg^{2+} ions in the active site coordinate both the ATP and HMDP substrates, making Mg^{2+} essential for substrate binding and pyrophosphoryl transfer.[104]

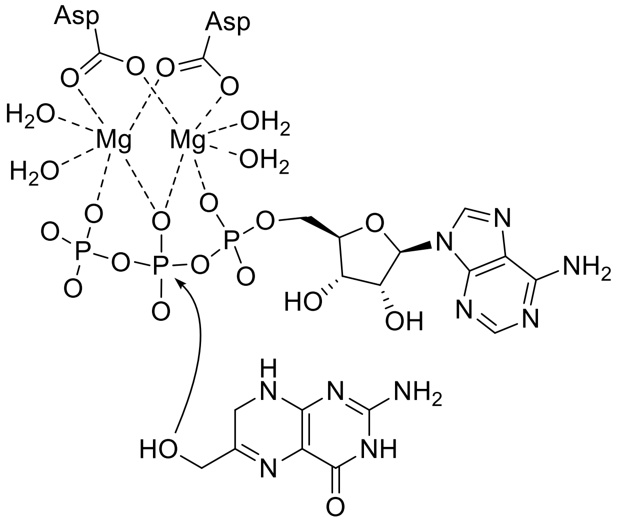

Scheme of ion coordination and specific pyrophosphokinase activity (EC 2.7.6.3).

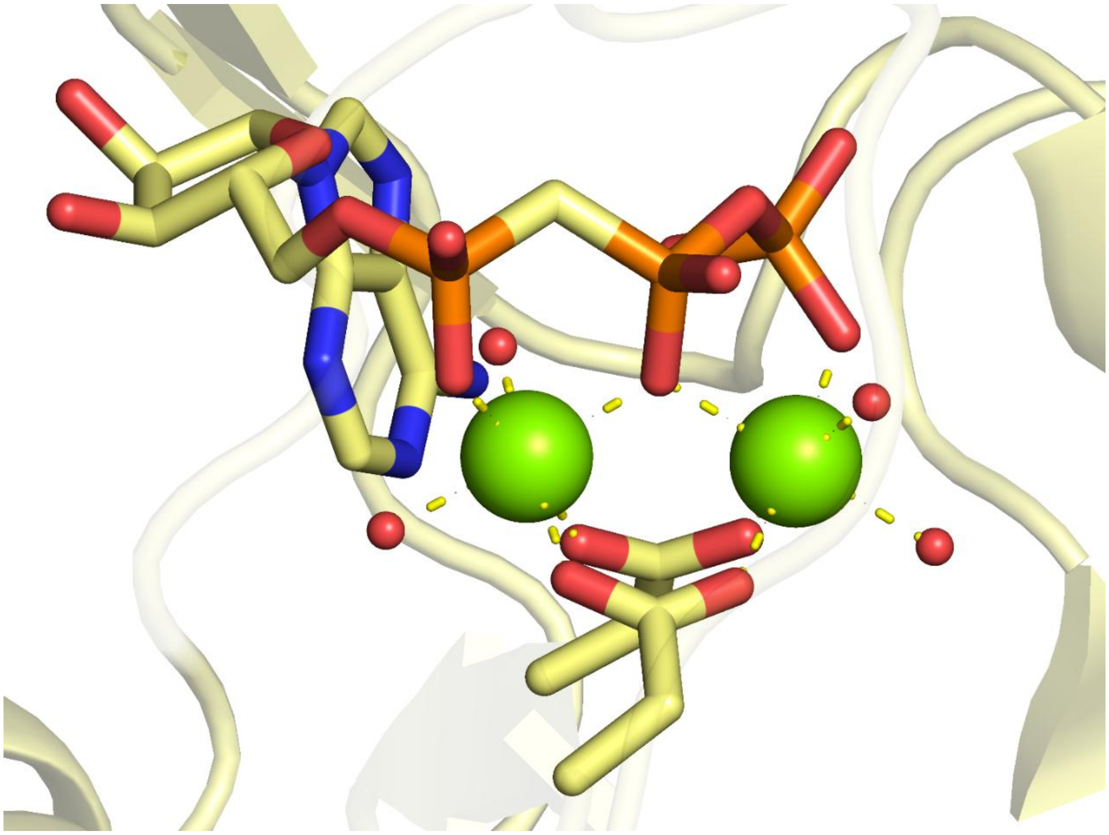

ATP and Mg^{2+} coordination in the representative structure 4CRJ.

Class II aaRS and biotin synthetases

Three metal ion positions are conserved in this superfamily, some resolved structures may be missing one or more of them. Two Mg ions coordinate the βγ phosphates while a third Mg^{2+} ion coordinates the αβ phosphates. A glutamate and a serine residue also take part in the Mg coordination.

Two highly conserved carboxylate residues of class II aaRSs participate with Mg^{2+} in ATP binding and coordination; these residues are absolutely required for activity; class II synthetases require 2-3 Mg^{2+} ions for the aminoacylation activation reaction, unlike class I (1 Mg^{2+}).[105]

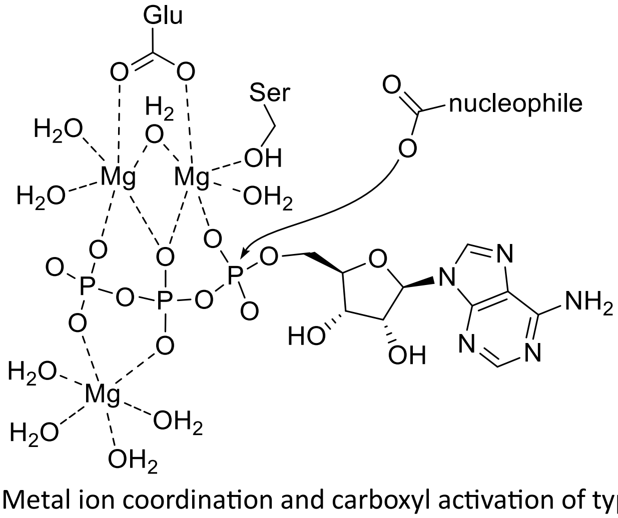

Metal ion coordination and carboxyl activation of typical amino acid tRNA synthetases.

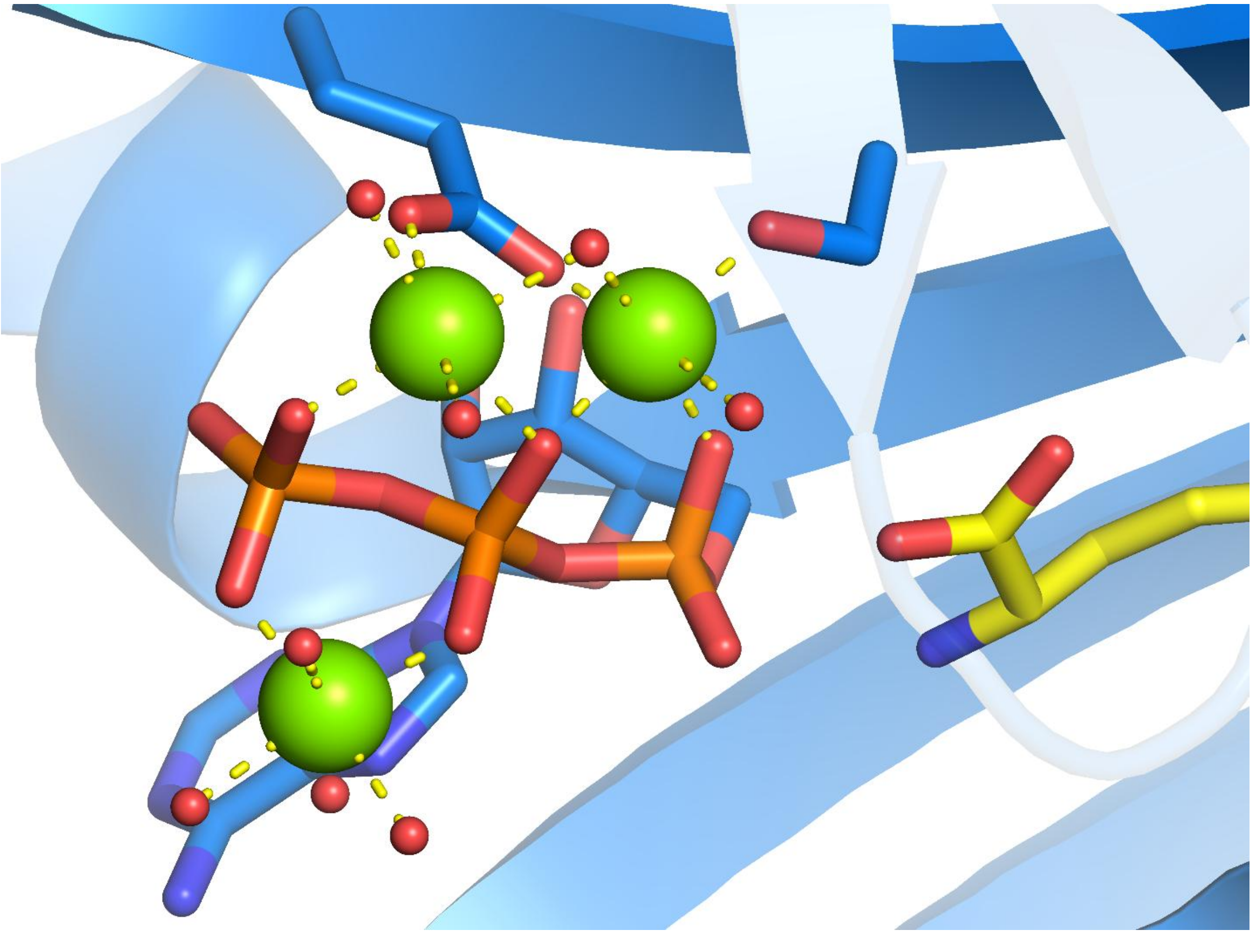

Substrate and ion coordination in the representative structure 6AAZ.

Nudix

This superfamily has 2 catalytic metal ions. One Mg^{2+} ion is coordinated by the αβ, while another one by the βγ phosphate oxygen atoms. The two Mg^{2+} ions are coordinated by a conserved glutamate residue, while the Mg^{2+} ion in the βγ position is further coordinated by an additional glutamate residue and the Mg^{2+} ion in the αβ position by a main-chain carbonyl group.

NudX-family hydrolases coordinate Mg^{2+} through conserved Nudix box glutamate residues that are essential for catalytic activity; kinetic and structural analyses support a two- or three-metal-ion mechanism in which divalent cation coordination by the Nudix box is required for phosphodiester bond hydrolysis. [106]

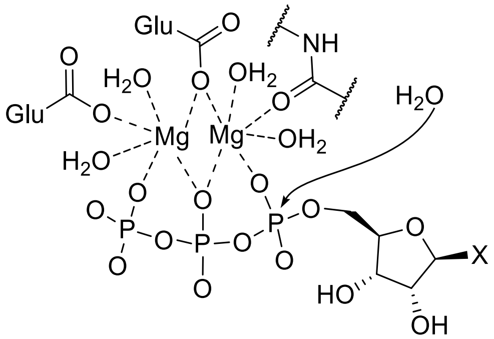

Schematic ion coordination and NTP pyrophosphate hydrolysis (EC 3.6.1.-)

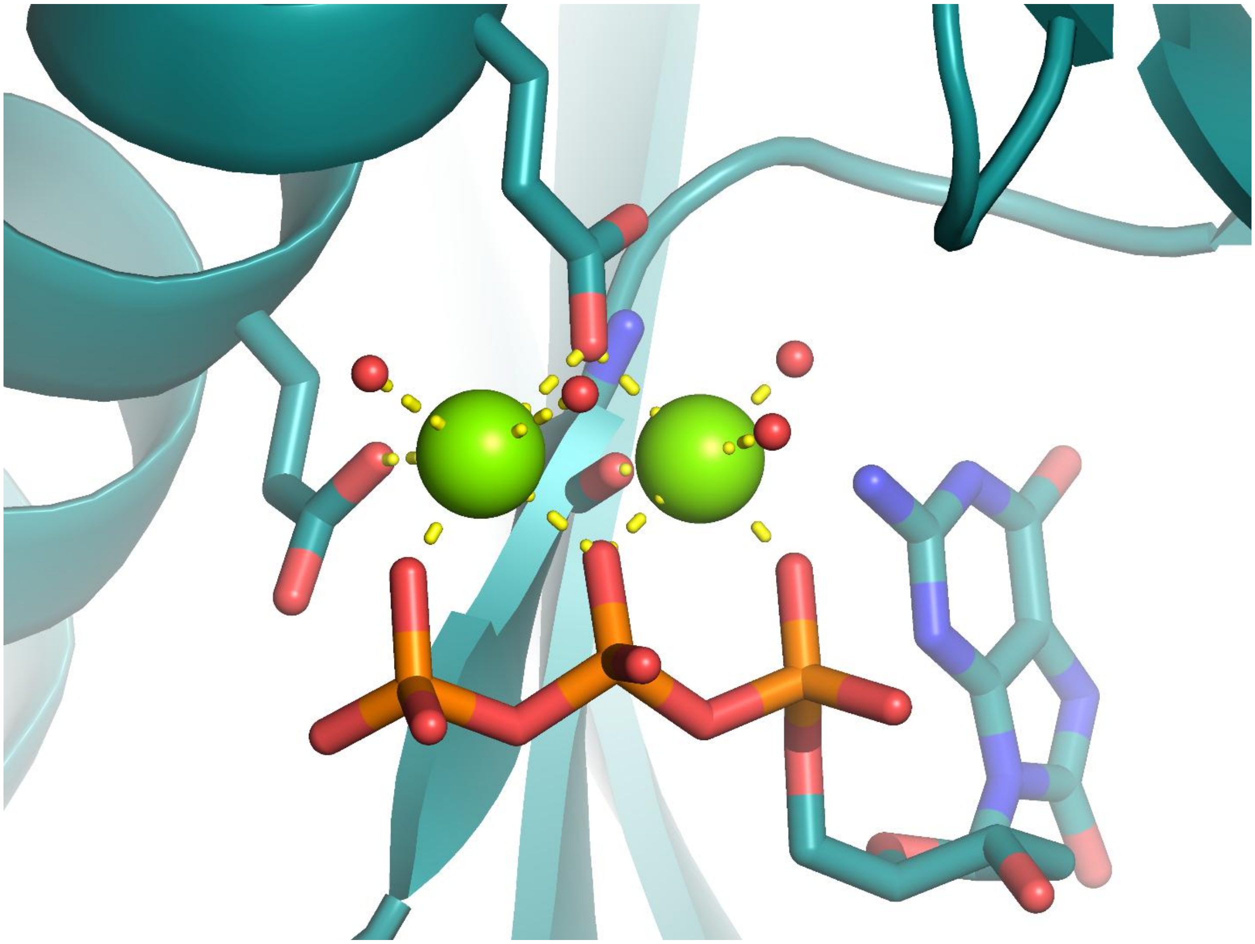

NTP and Mg^{2+} ion coordination in the representative structure 6FL4.

Beta and beta-prime subunits of DNA dependent RNA-polymerase

In our dataset only a few structures are found for this superfamily. Despite the excellent agreement upon alignment, only one with an NTP was resolved, while many RNA chains with Mg^{2+} at the 3' end were also identified. The structure 1TWF was resolved in the presence of two catalytic Mn^{2+} ions. One Mn^{2+} ion is coordinated by αβ, and the other by the βγ phosphate group oxygens. Two aspartate residues further coordinate both metal ions. Additionally, an extra aspartate residue coordinates each of them.

Site-directed mutagenesis of the three catalytic Asp residues of the DFDGD motif in the β' subunit (D460A, D462A, D464A) creates dominant-lethal, transcriptionally inactive RNAPs that retain promoter binding and open-complex formation; Fe^{2+}-mediated hydroxyl-radical mapping of the active site identifies these Asp residues as metal-ion ligands, directly demonstrating their essential catalytic role.[107]

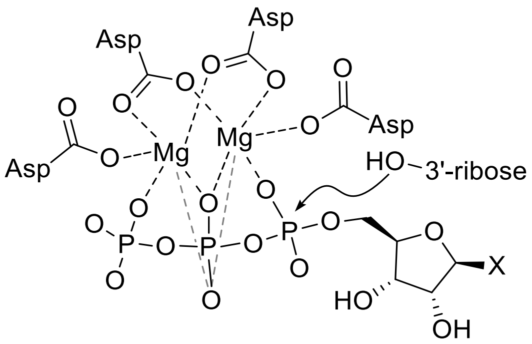

RNA polymerization scheme with Mg^{2+} ions (EC 2.7.7.6).

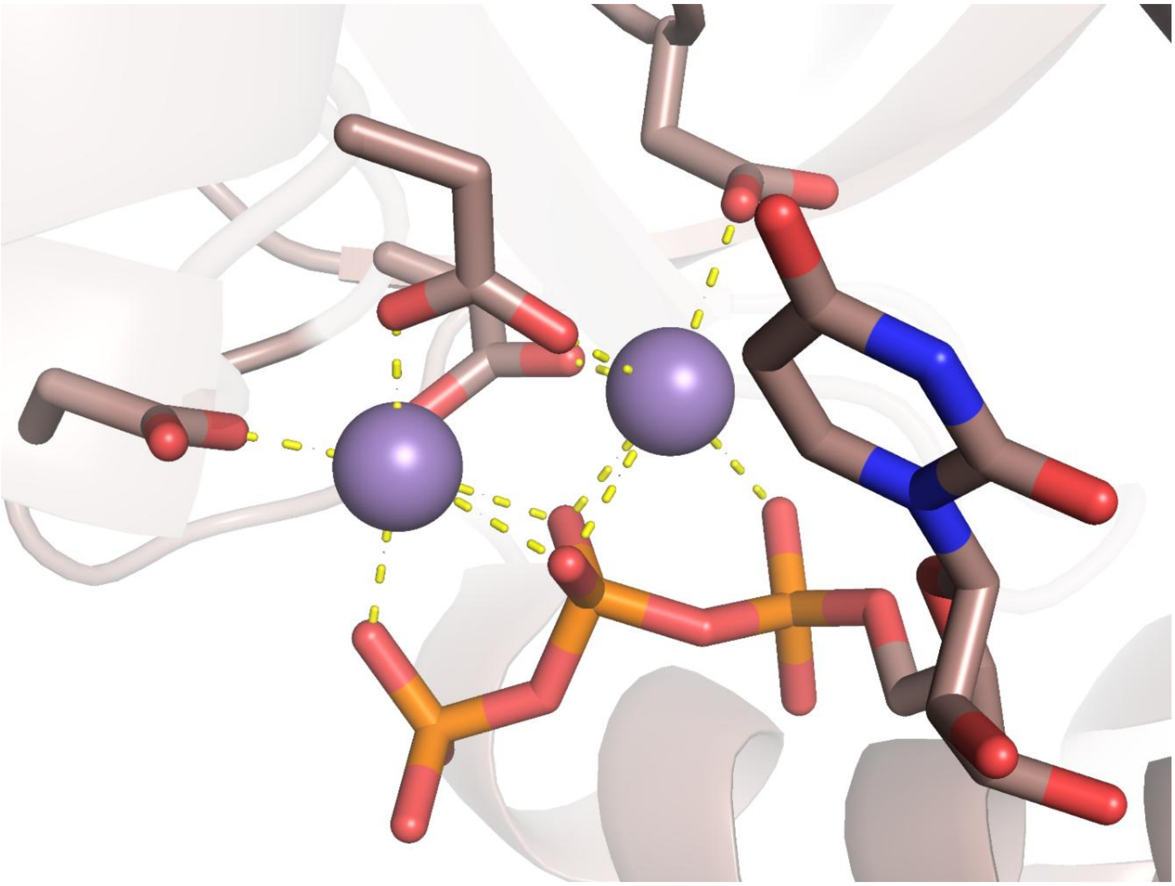

UTP and Mn^{2+} ion coordination in the representative structure 1TWF.

tRNA-splicing ligase RtcB-like

This superfamily was identified by InterPro (there is a corresponding SUPFAM entry: Hypothetical protein PH1602, d.261.1; exceptionally, here we use the InterPro name as it is more descriptive). Enzymes belonging to EC 6.5.1.8 make use of GTP, and two catalytic metal cation cofactors. Interestingly, in the case of this superfamily, these metal ions are manganese. The catalytic reaction is less effective with cobalt and nickel, whereas zinc and copper are inactive and potently inhibit manganese-dependent guanylylation.[108] Intriguingly, the αβ-coordinated catalytic Mn^{2+} ion is further coordinated by two histidine and a cysteine residue. An additional water molecule participates in its coordination. A second Mn^{2+} is in a βγ coordination position, and has a coordination number of only 5, which is formed by the previously mentioned cysteine, and an additional histidine and aspartate residues.

Guanylylation of RtcB is optimal with Mn^{2+}; both 2',3'-cyclic phosphodiesterase and ligase reactions require Mn^{2+} and are abolished by mutation of active-site residues; Mn^{2+} adopts pentahedral coordination contacting beta- and gamma-phosphates of GTP, essential for activation.[108]

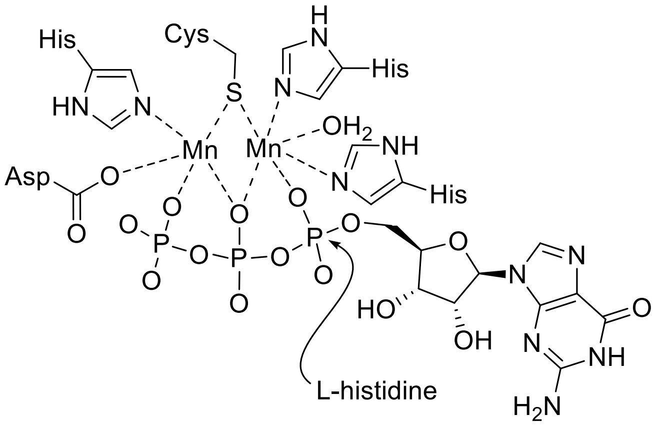

Metal coordination and reactivity (EC 6.5.1.8) RtcB-like ligases.

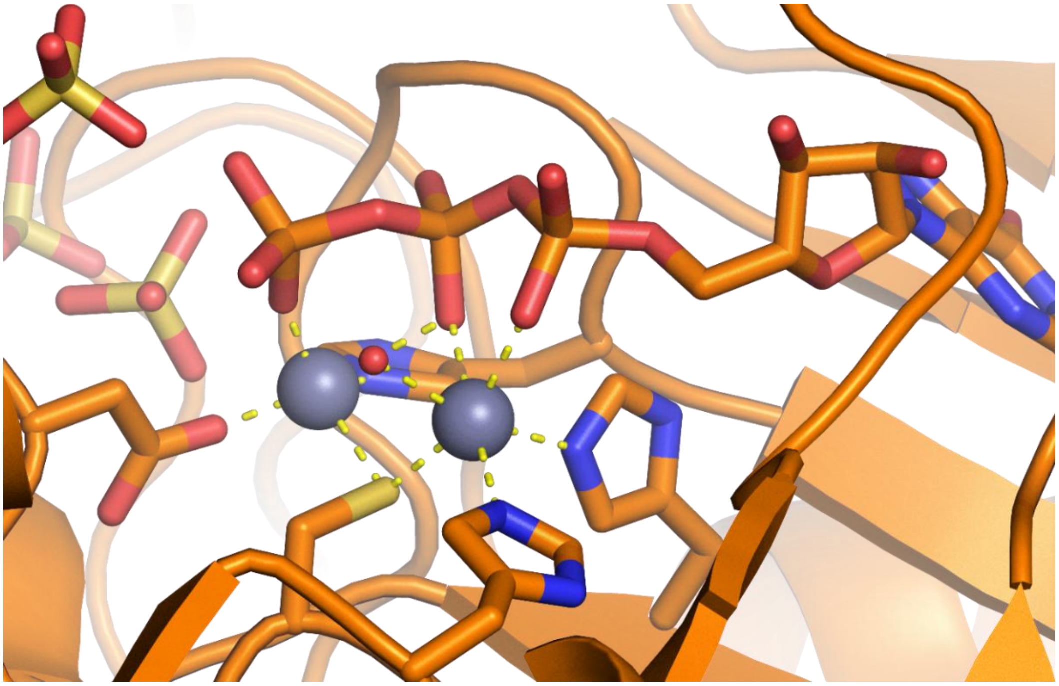

GTP and metal ion coordination in the representative structure 8DCD. The structure is resolved with Zn^{2+} ions that inhibit the reaction.

Aerobactin siderophore biosynthesis, IucA/IucC-like

Known ECs: 6.3.2.39/54-58

This entry is not classified at a superfamily level as no superfamily could be assigned to this particular group by SUPFAM. However, the InterPro database identifies them as belonging to the Aerobactin siderophore biosynthesis, IucA/IucC-like family. Its members exhibit a first αβ-coordinated and an additional αγ-coordinated Mg^{2+}. Interestingly, three carboxyl groups, sidechains of an aspartate and two glutamate residues, participate in the ion coordination. An additional water molecule also coordinates the Mg^{2+}.

LucA catalyzes Mg^{2+}-dependent, ATP-driven amide bond formation in aerobactin biosynthesis, following an ordered ter-reactant mechanism in which MgATP binds first; steady-state kinetic analysis establishes the Km values for all three substrates—ATP, citrate, and Nε-hydroxylysine—and confirms that Mg^{2+} is an obligate co-substrate for catalysis. [109]

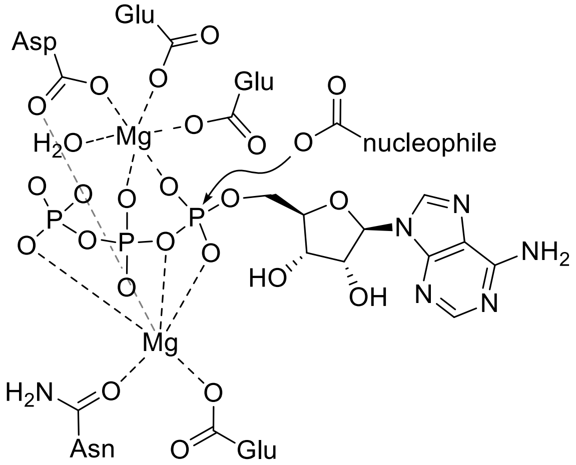

Proposed reactivity based on the associated peptide synthetase ECs 6.3.2.-.

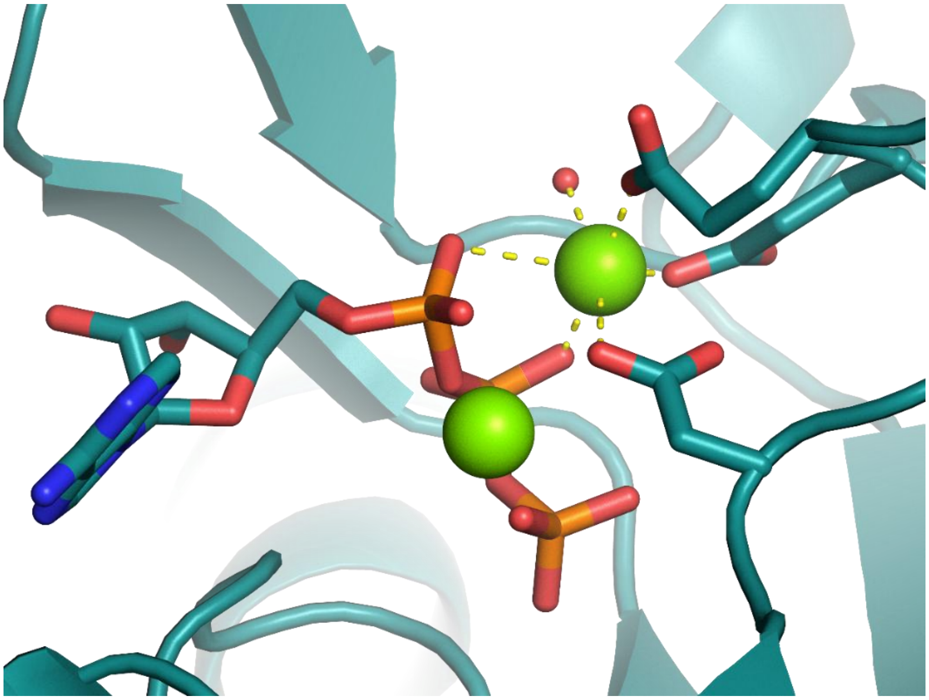

ATP and metal ion coordination in the representative structure 7TGK.

Pyrophosphatases with αβγ coordination on the (-) side

dUTPase-like

Members of this superfamily have one Mg^{2+} ion that coordinates the αβγ phosphates. There are dCTP deaminases in this superfamily (e.g. PDB 1XS1, 2QXX, 2V9X, 4XJC), not all of which are dUMP-forming. In the case where there is no diphosphatase activity of the given deaminase, the spatial exclusion of a potential nucleophilic water molecule is likely to prevent the hydrolysis of the phosphate chain of the bound nucleotide.[110]

Mg^{2+} is essential for dUTPase activity, dramatically enhancing substrate affinity—increasing binding of dUTP and dUDP by 100-fold and 10-fold, respectively—and is required for correctly positioning the α-phosphorus for in-line nucleophilic attack; k_{cat} and K_m change markedly in the presence of Mg^{2+} or Mn^{2+}.[111]

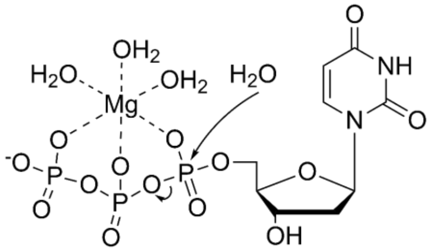

Reaction scheme of dUTP hydrolysis.

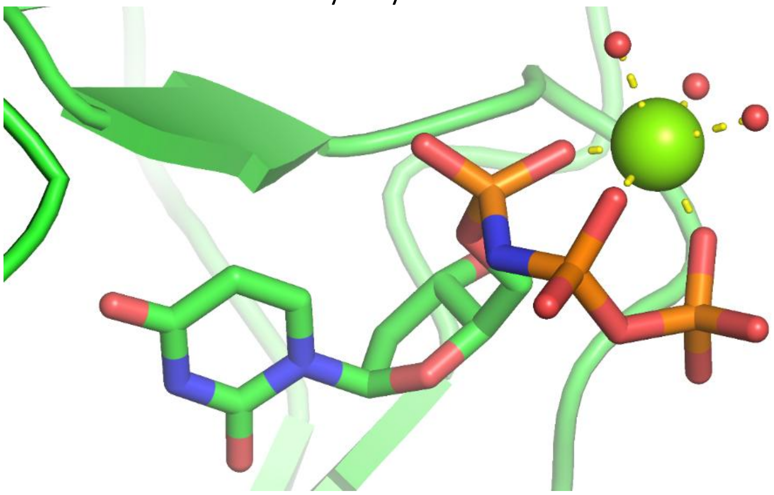

NTP and metal ion coordination in the representative structure 1RN8.

Nucleotidylyl transferase

One structurally conserved aspartate (or in some cases glutamate) residue points towards the Mg^{2+} ion and can contribute to its coordination mediated through the coordinating water molecules.

Radiolabelled [α-^{32}P]GTP transfer to the vaccinia virus capping enzyme demonstrates that GMP-enzyme intermediate formation is absolutely dependent on Mg^{2+}; Mn^{2+}, Co^{2+}, Ca^{2+}, Cu^{2+}, Ni^{2+} and Zn^{2+} all fail to support the guanylylation reaction, establishing strict Mg^{2+} specificity for this step.[112]

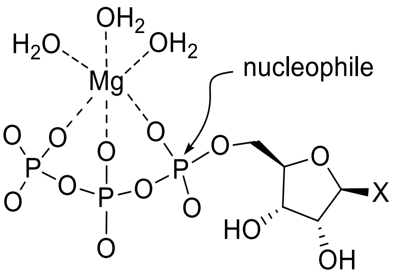

General nucleotylyl transfer reaction scheme with Mg^{2+} coordination. The superfamily is found among many EC categories, not only in nuceotidyltransferases (2.7.7.-) but also in ligases, like class I tRNA synthases (6.1.1.-).

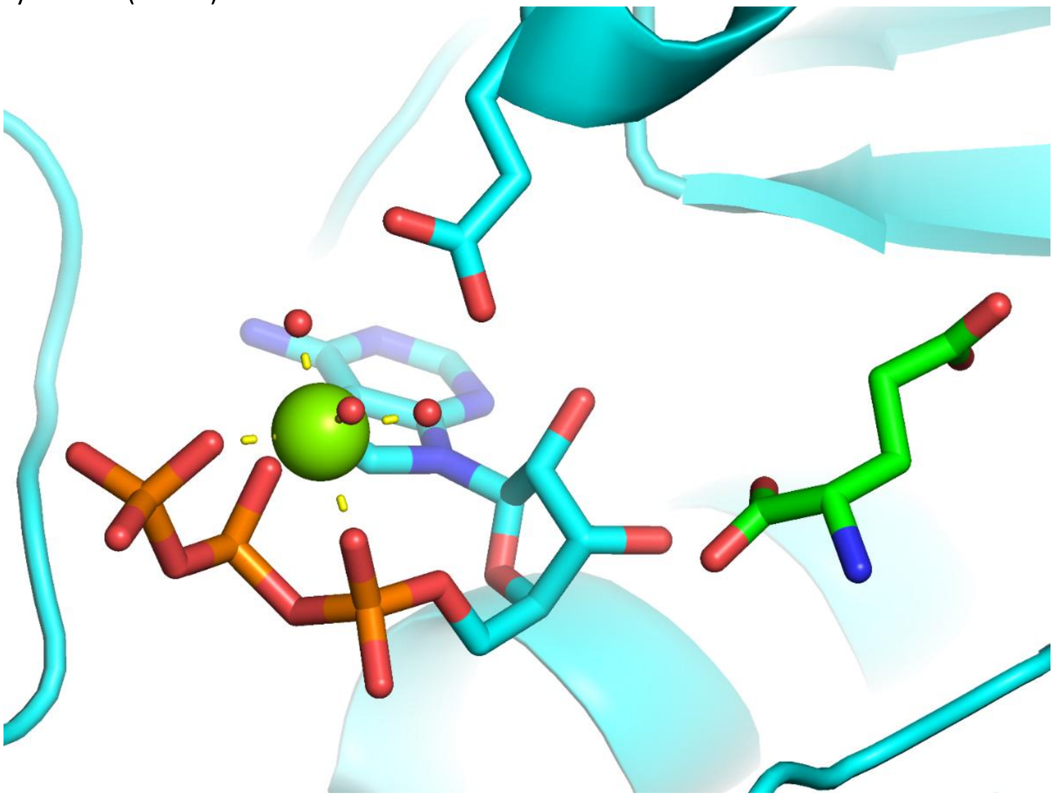

NTP and metal ion coordination alongside an amino acid substrate in the representative structure 1J09.

ITPase-like

The superfamily has limited structural data found in our analysis. The representative structure was resolved in the presence of a calcium which is coordinated by αβγ phosphate group oxygens as well as a glutamate residue and two water molecules, but for reactivity, the enzyme prefers Mg^{2+}. An additional Na^{+} ion is present coordinated by the α phosphate group. The substrate is typically a non-nucleic acid-forming NTP, e.g. XTP or ITP.

Direct biochemical characterisation of human ITPA: Km(ITP) = 0.51 mM, Km(dITP) = 0.31 mM, Km(XTP) = 0.57 mM; kcat(ITP) = 580 s^{-1}; Mg^{2+} or Mn^{2+} required for activity; inhibited by IDP; establishes absolute divalent cation requirement for pyrophosphohydrolase activity.[113]

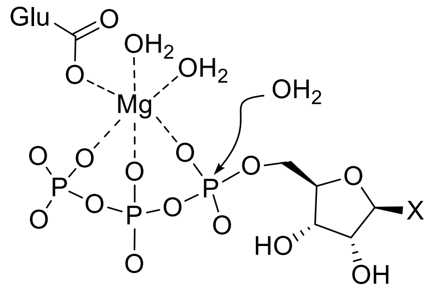

ITP pyrophosphatase reaction and Mg^{2+} ion coordination.

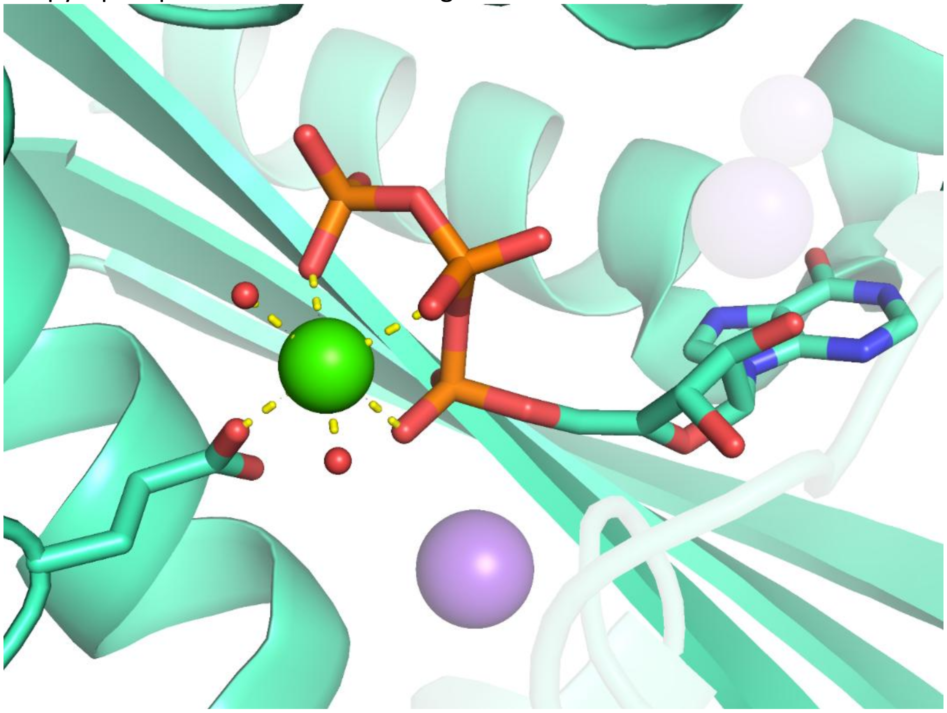

NTP and metal ion coordination in the representative structure 2Q16.

CoaB-like

Members of this superfamily are key enzymes in the production of coenzyme A. The eukaryotic enzymes require ATP, in contrast to the bacterial enzymes which require CTP. A single Mg^{2+} ion is coordinated by the αβγ phosphate oxygen atoms as well as by an aspartate residue conserved in all species.[114] The carboxylate group of the ligand is positioned in an orientation towards the Mg^{2+} ion that is ideal for a nucleophilic attack at the α-phosphate.

PPCS activity is supported by both Mg^{2+} and Mn^{2+}; the carboxylate of phosphopantothenate is activated by coordination to a Mg^{2+} ion that renders the CTP alpha-phosphate electrophilic; Km for CTP is 156 µM with a kcat of 2.9 s-1.[115]

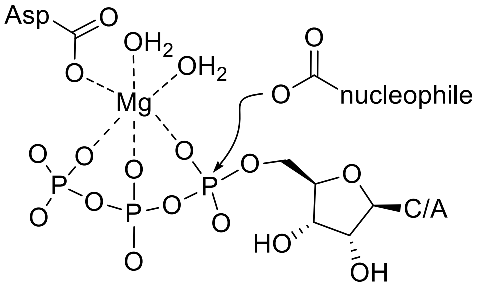

Scheme of the first step of the ligase reaction (EC 6.3.2.5/51) catalyzed by the CoaB-like superfamily.

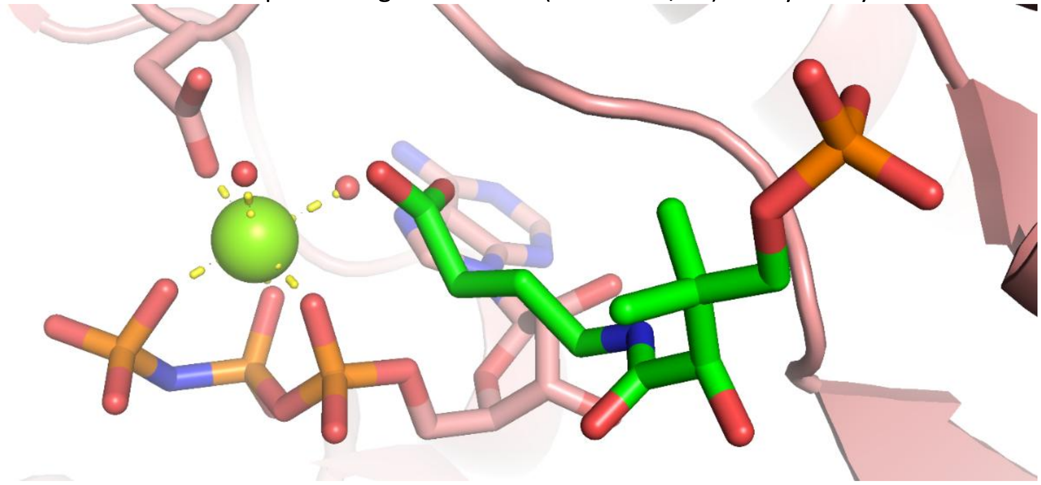

ATP and metal ion coordination in the representative structure 7EDZ.

EPT/RTPC-like or (**RNA 3'-terminal phosphate cyclase, RPTC, insert domain**)
Known EC: 6.5.1.4 (RNA 3'-terminal-phosphate cyclase (ATP))
Members of the superfamily may function both with Mg^{2+} and Mn^{2+}.[116] The ion is coordinated by nonbridging oxygens from each of the ATP phosphates (αβγ), two water molecules, and a glutamate residue.
Biochemical characterisation of S. aureus phosphatidylglycerophosphate synthase (SaPgsA), a CDP-alcohol phosphotransferase of the EPT/RTPC-like superfamily, demonstrates strict Mg^{2+} dependence: activity is highest with Mg^{2+}, abolished by Ca^{2+} or EDTA, and substantially reduced with Mn^{2+}, Co^{2+} or Zn^{2+}; crystal structures reveal a binuclear metal centre coordinated by the four conserved aspartates of the signature DxDGxxAR…GxxxDxxxD motif.[117]

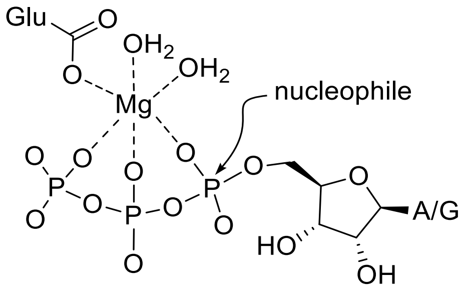

The ligase-like activation can be achieved by ATP and GTP, according to ECs 6.5.1.4-5.[118]

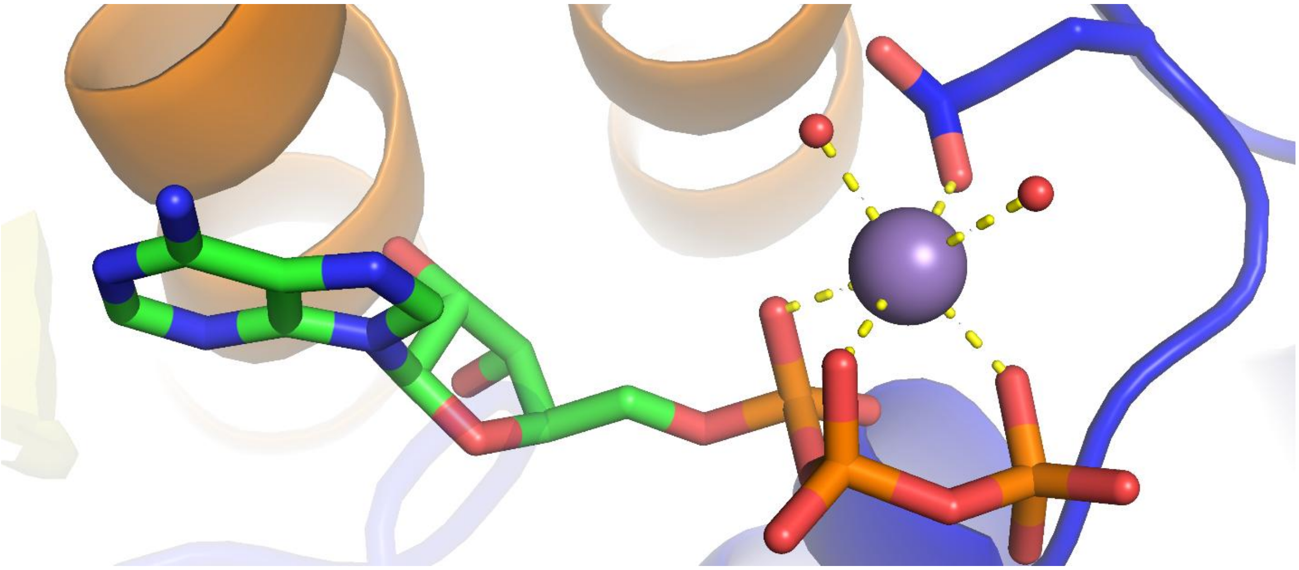

ATP and Mn^{2+} ion coordination in the representative structure 3TUX.

YrdC/RibB

Enzymes belonging to EC 2.7.7.87 are L-threonylcarbamoyladenylate synthases, while those belonging to EC 3.5.4.25 are GTP cyclohydrolase II, which mainly act on the guanine base but loses phosphates besides. We identified two remarkably different subgroups in this superfamily, those similar to PDB 3VTH and those to 7UF0. Only structures of the PDB 3VTH-like subgroup contained nucleotide (analogs) in our dataset. Our representative structure (3VTH) shows an αβγ-coordination of its catalytic Mg^{2+} ion. This ion is also coordinated by a serine residue, and presumably two water molecules are missing from the active site in this structure.

Mg^{2+}-coordinating residues at the active site are strictly conserved across the YrdC/Sua5 family; two invariant residues involved in nucleotide binding are essential for catalytic activity; MgATP is the obligate co-substrate for threonylcarbamoyladenosine (t^6A) formation at tRNA position 37.[119]

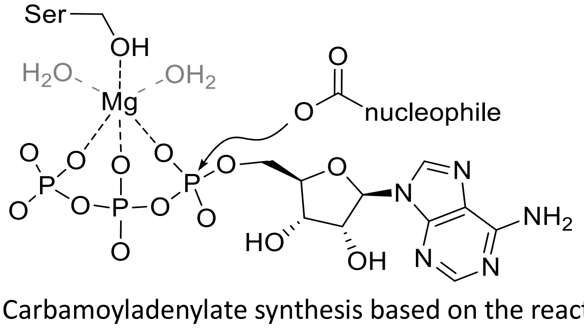

Carbamoyladenylate synthesis based on the reaction catalyzed by the structure 3VTH.

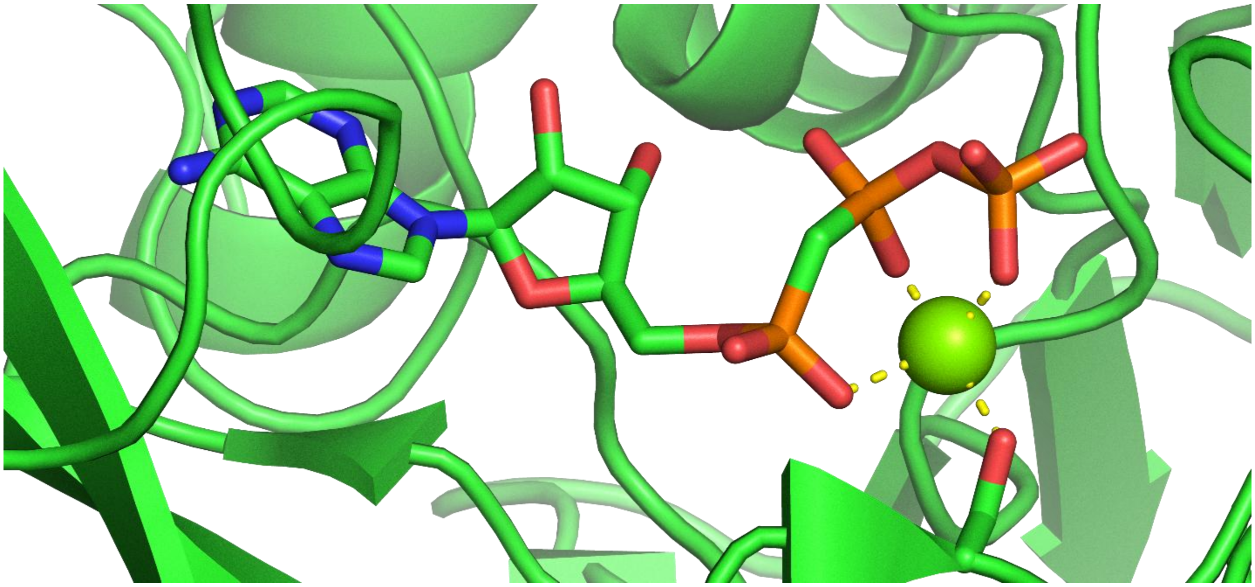

ATP and Mg^{2+} ion coordination in the representative structure 3VTH.

YojJ-like

The representative structure was solved in the presence of a Mn^{2+} ion, a substitute for magnesium. The metal ion is octahedrally coordinated by the αβγ phosphate groups of the nucleotide together with an aspartate residue and two water molecules. Despite the overall dimerization reaction, only one (d)ATP is present in the active site.

The diadenylate cyclase activity of DAC family enzymes is directly demonstrated by *in vitro* [^{32}P]-ATP assays, in which DisA orthologs synthesize c-di-AMP and pyrophosphate from two molecules of ATP.[120-121] The reaction is strictly dependent on divalent metal ions: in the absence of any divalent ion no activity is detected, while Mg^{2+}, Mn^{2+} or Co^{2+} all support catalysis.[122] Active-site mutations in the conserved DGA and RHR motifs (e.g., D75N or D86A; RHR→AAA) eliminate activity, confirming the catalytic role of the metal-coordinating aspartate and substrate-positioning arginines.[122]

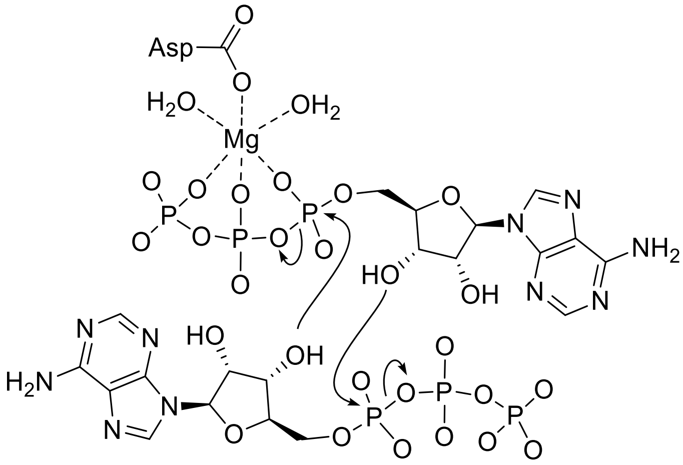

Presumed cyclization reaction and primary ion coordination.

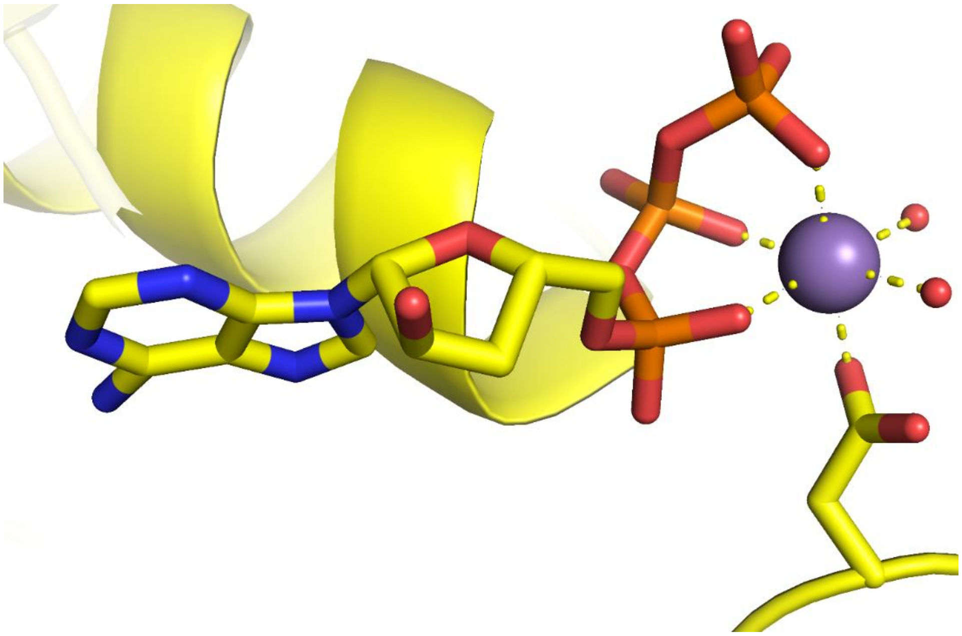

dATP and Mn^{2+} ion coordination in the representative structure 4YVZ.

Poly(A) polymerase catalytic subunit-like

Poly(A) polymerase catalyzes template-independent extension of the 3'-end of a DNA or RNA strand by one nucleotide at a time. The Poxvirus enzyme creates the 3'(poly)A tail of mRNAs, and is a heterodimer of a catalytic and a regulatory subunit, out of which this category corresponds to the catalytic subunit.[123] There are two metal ions at the active site, one is coordinated by all three phosphate groups of the nucleotide, and further coordinated by two aspartate residues, whereas the other is not coordinated by the NTP, but three aspartates and to the oligonucleotide's 3'OH nucleophile.

The crystal structure of the poly(A) polymerase–MgATP–RNA ternary complex reveals two active-site Mg^{2+} ions that are indispensable for catalysis: one coordinates the β- and γ-phosphates of ATP and stabilizes the leaving pyrophosphate, while the other positions the RNA 3′-OH for in-line nucleophilic attack; kinetic measurements confirm that Mg^{2+} is absolutely required and cannot be substituted by Ca^{2+}.[124]

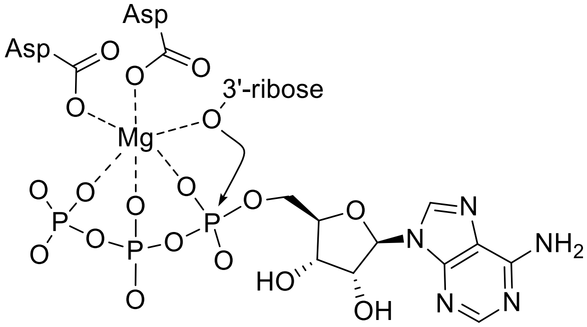

Scheme of the 3' adenylate addition catalyzed by 2.7.7.19.

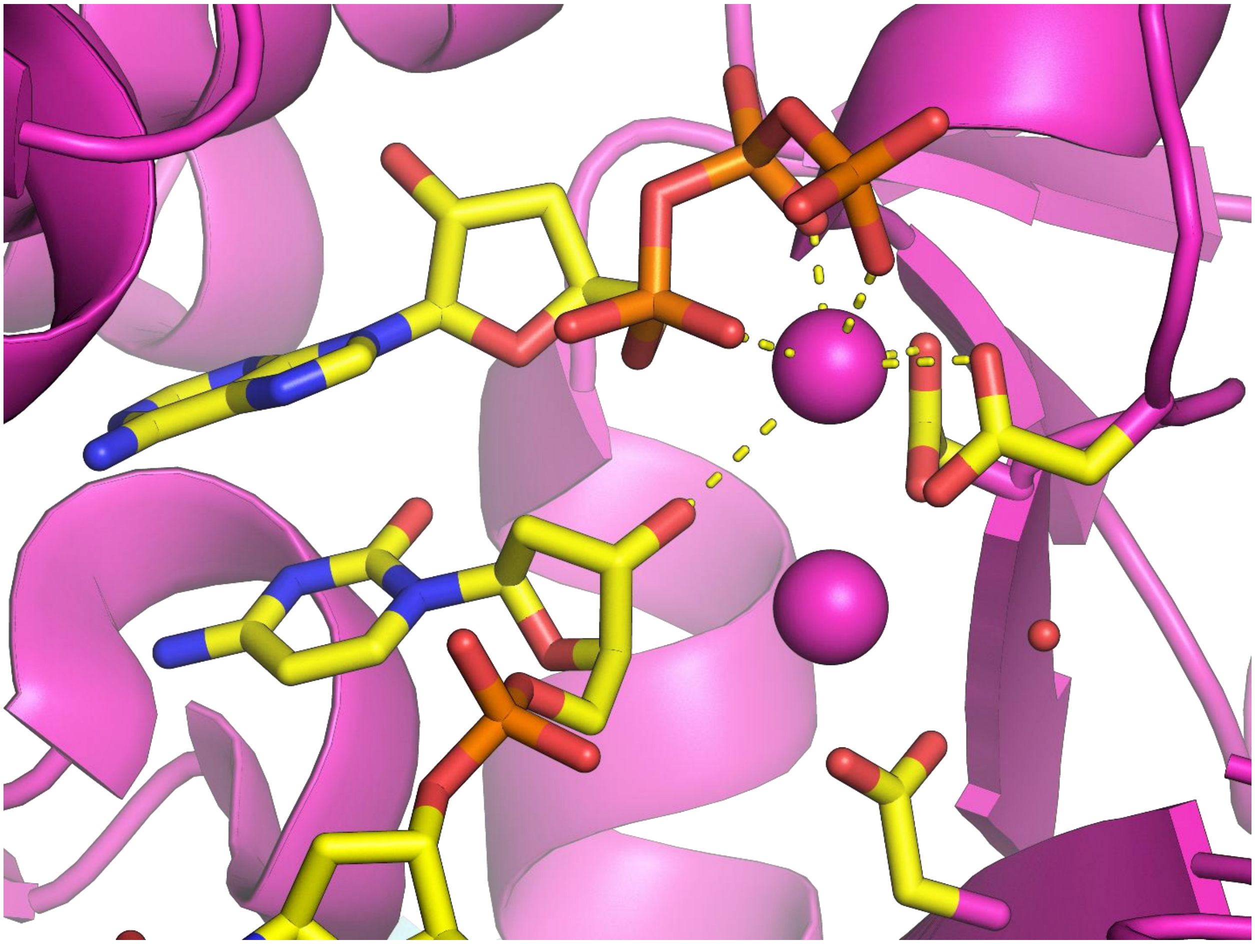

Substrate and metal ion binding in the representative structure 3ERC.

DNA/RNA polymerases

It is the most populated pyrophosphatase superfamily in our dataset. It exhibits a clear two-metal-ion coordination. In some of the structures a third metal ion is present (not far from the γ phosphate) which may possess a role in the release of the ligand.[125] All structures have a first Mg^{2+} ion with a clear αβγ phosphate coordination and a second Mg^{2+} that coordinates the α phosphate and the attacking hydroxyl group of the ligand nucleotide. Two aspartate residues are located on the nearby beta sheet and are involved in the coordination of both metal ions, whereas an additional glutamate coordinates only the second metal ion.

Stopped-flow fluorescence kinetics show that Asp882 (Mg-A ligand) is required for the fingers-closing conformational change, while Asp705 (Mg-B ligand) facilitates entry of the second Mg^{2+} into the active site; Asp→Ala mutants lose catalytic activity entirely, demonstrating that both Mg^{2+} coordination sites are essential for phosphoryl transfer.[126]

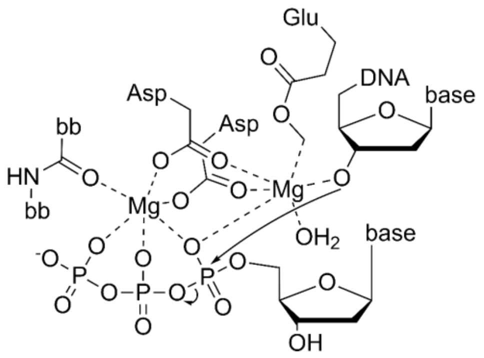

Typical ion coordination depicted in the example of DNA polymerization.

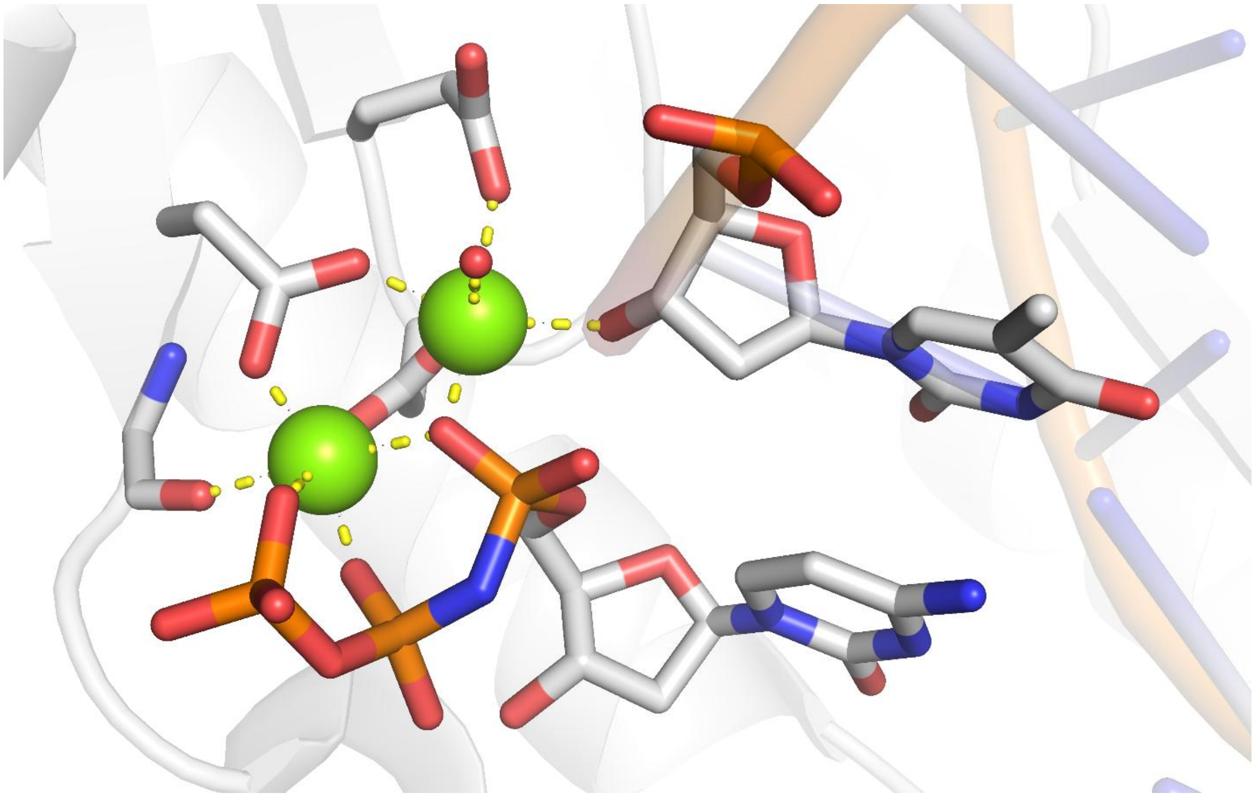
NTP, nucleic acid and metal ion coordination in the representative structure 4RU9.

Nucleotidyltransferase

Similar to the DNA/RNA polymerases, the majority of this superfamily belongs to the DNA-directed DNA polymerases. Even though the two folds are structurally remarkably different, the nucleotide, the catalytic residues, and the coordinating metal ions are positioned and oriented almost identically. Nucleotidyltransferases also have 2 metal ions at the catalytic site, the first Mg^{2+} coordinating the αβγ phosphates while the second Mg^{2+} the α phosphate and the attacking hydroxyl group of the ligand. Instead of the coordinating glutamate residue, all three residues are aspartates in the case of the Nucleotidyltransferases.

Biochemical characterization of human TdT establishes divalent cation requirement for all polymerization activity; metal ion preference: $Mg^{2+} \geq Co^{2+} > Mn^{2+}$; Km values for dNTP substrates measured under defined Mg^{2+} and Co^{2+} conditions; activity fully abolished by EDTA chelation, demonstrating essential metal dependence.[127]

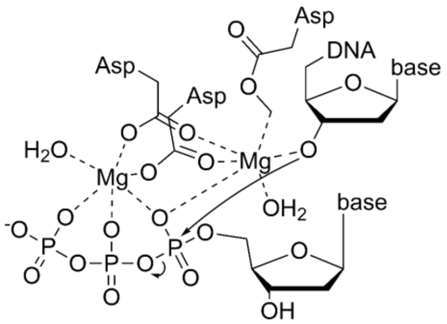

Typical ion coordination depicted in the example of DNA polymerization.

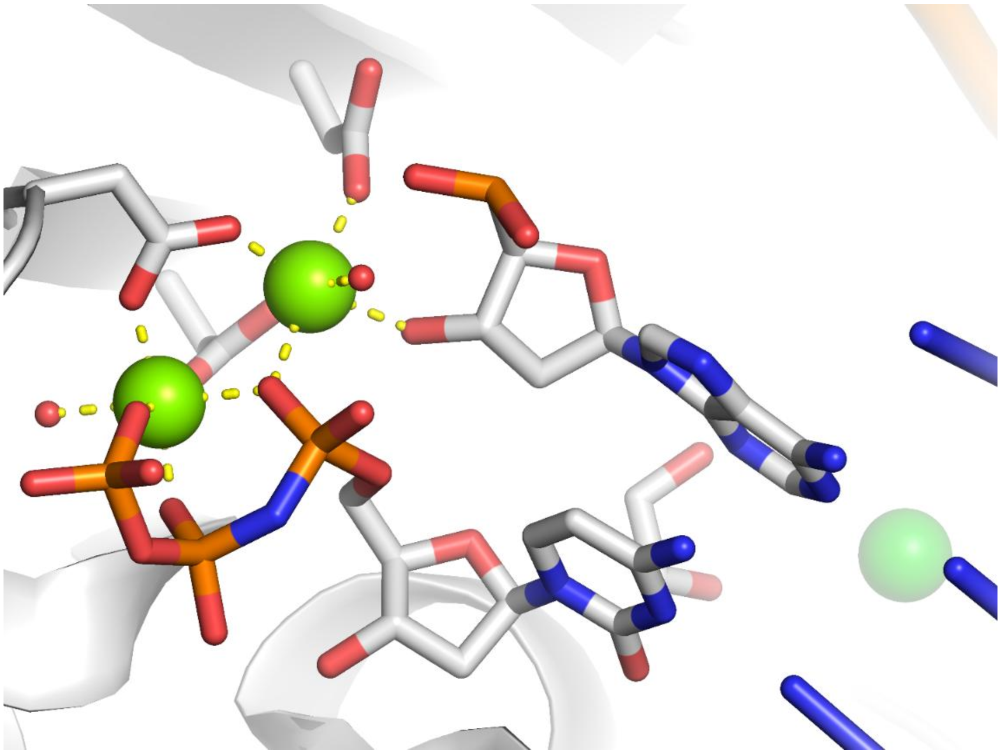

NTP, nucleic acid and metal ion coordination in the representative structure 6P1P.

Nucleotide cyclase

Members of the Nucleotide cyclase superfamily produce 3',5'-cyclic NMP and pyrophosphate while they hydrolyze NTP. Two metal ions are required for catalysis.

Intriguingly, even though Ca^{2+} typically inhibits NTP processing enzymes and Mg^{2+} is the typical catalytic enzyme, in this superfamily, in the 'soluble' adenylyl cyclase (sAC) enzyme active site (*S. platensis*) Ca^{2+} plays the pinching metal ion role at the ABG(–) site, while an α-coordinated Mg^{2+} coordinates the attacking hydroxyl group of the ATP, orienting it for the cyclase reaction.[128] Most enzymes of this SF, however, use Mg^{2+} in both ion positions. The metal ion coordinated by the αβγ phosphates is further coordinated by two aspartate residues in its vicinity.

Site-directed mutagenesis of the two Mg^{2+}-coordinating Asp residues in mammalian adenylyl cyclase: mutations abolish or dramatically reduce cAMP synthesis; biochemical assays establish that Mg-A (tightly bound, activates 3'-OH nucleophile) and Mg-B (labile, stabilises leaving pyrophosphate) are both essential for catalysis.[129]

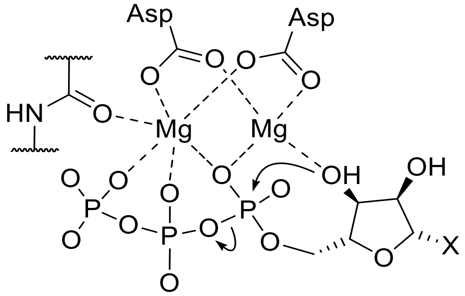

Nucleotide cyclisation and typical metal ion coordination.

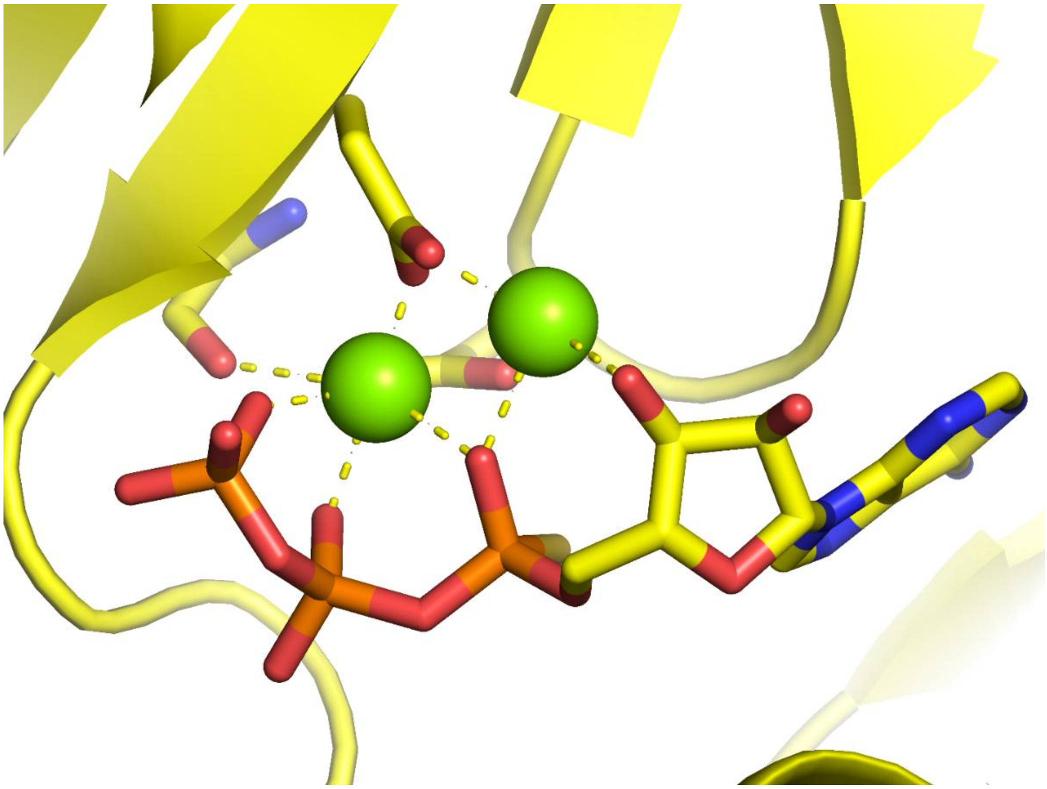

Metal ion and NTP coordination in the representative structure 1WC6.

Prim-pol domain

Members of this superfamily have two catalytic divalent metal ions. One is coordinated by nonbridging oxygens of the αβγ phosphates, and another one of only the α phosphate. Two conserved aspartate residues are included in the coordination of both Mn ions, and an additional aspartate/glutamate residue coordinates the Mn ion in the α position. Notably, prim-pol is a Mn ion dependent enzyme that shows significantly improved primase and polymerase activities when binding Mn, rather than Mg ions as cofactors.[130]

Glu116 of the DxE motif is critical for Mn^{2+}-dependent primase and TLS activities; PrimPol's active site requires the DxE motif to favour Mn^{2+} for optimal incoming nucleotide stabilisation; DNA binding is enhanced 34-fold and polymerase activity 400-1000-fold by Mn^{2+} compared with Mg^{2+}.[131]

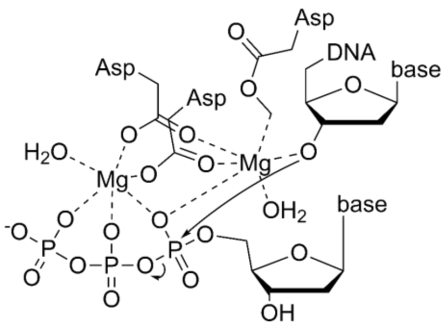

DNA polymerase reactivity and metal ion coordination associated with the Prim-pol domain superfamily.

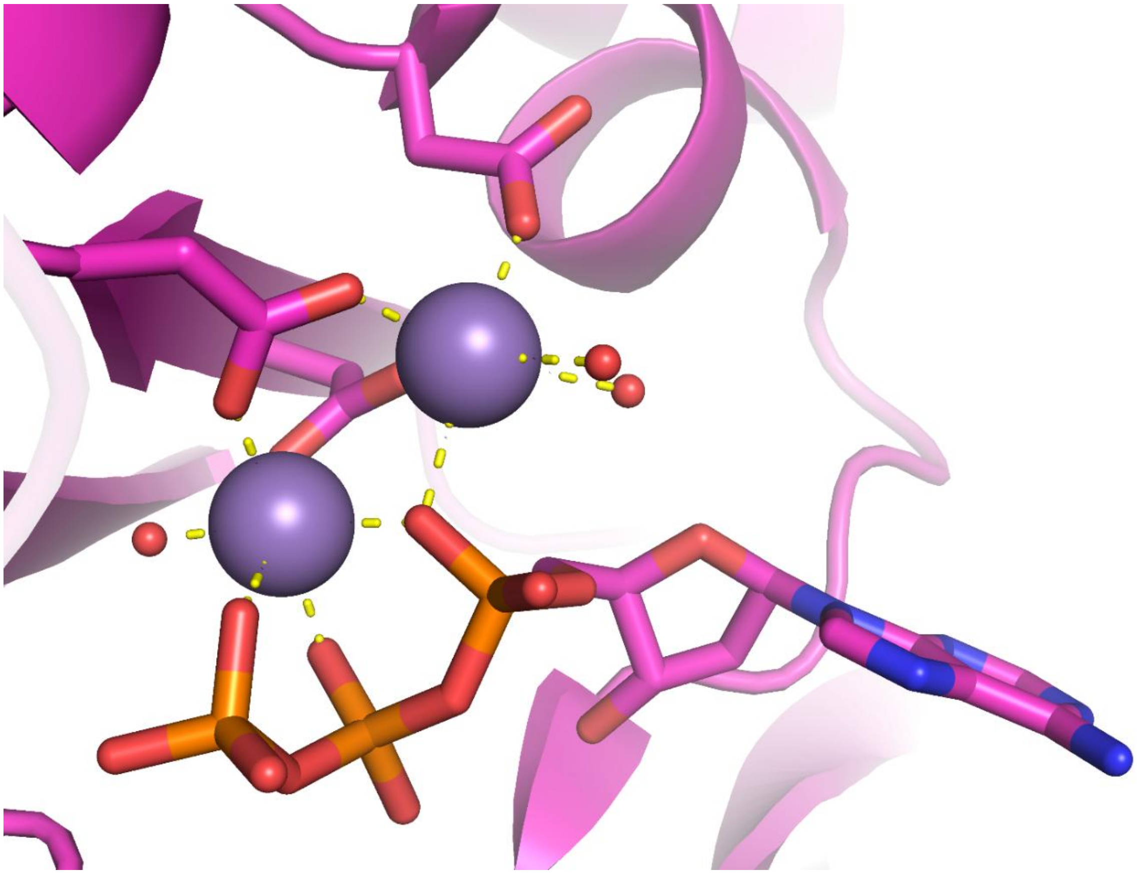

NTP and Mn^{2+} coordination in the representative structure 6R5D. The polymerization undergoes with both ions, Mn^{2+} is used for enhanced NTP affinity.

Influenza RNA-dependent RNA polymerase subunit PB1 (IPR001407)

Influenza RNA-dependent RNA polymerase is composed of three subunits; PB1, PA, and PB2, PB1 is the core of the complex and accounts for the polymerase activity.[132] They catalyze RNA-template-directed extension of the 3′ end of an RNA strand by one nucleotide at a time and can initiate a chain *de novo*. There is no corresponding SUPFAM category, this group is named at the InterPro family level (IPR001407), the corresponding ECOD domain classification is *helical bundle domain in reverse transcriptase-like polymerases*. The consensus coordination displays a typical polymerase arrangement, one Mg^{2+} coordinated by all three phosphate groups, and another that is coordinated by the 3′ hydroxyl of the priming nucleotide and the α-phosphate of the incoming NTP. We note that the fold coordinating the metal ions closely match the InterPro *Reverse transcriptase/Diguanylate cyclase* domain (IPR043128) found in *DNA/RNA polymerases* SF.

Isothermal titration calorimetry of the PA endonuclease domain shows two Mn^{2+} binding sites (500-fold higher affinity than Mg^{2+}); direct nuclease activity assays with Mg^{2+} vs Mn^{2+} show Mn^{2+} is preferred in vitro; eight active-site point mutants lose cleavage activity, confirming the metal coordination residues are catalytically essential.[133]

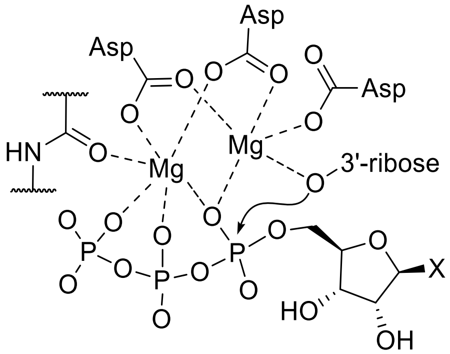

RNA polymerization and typical ion coordination.

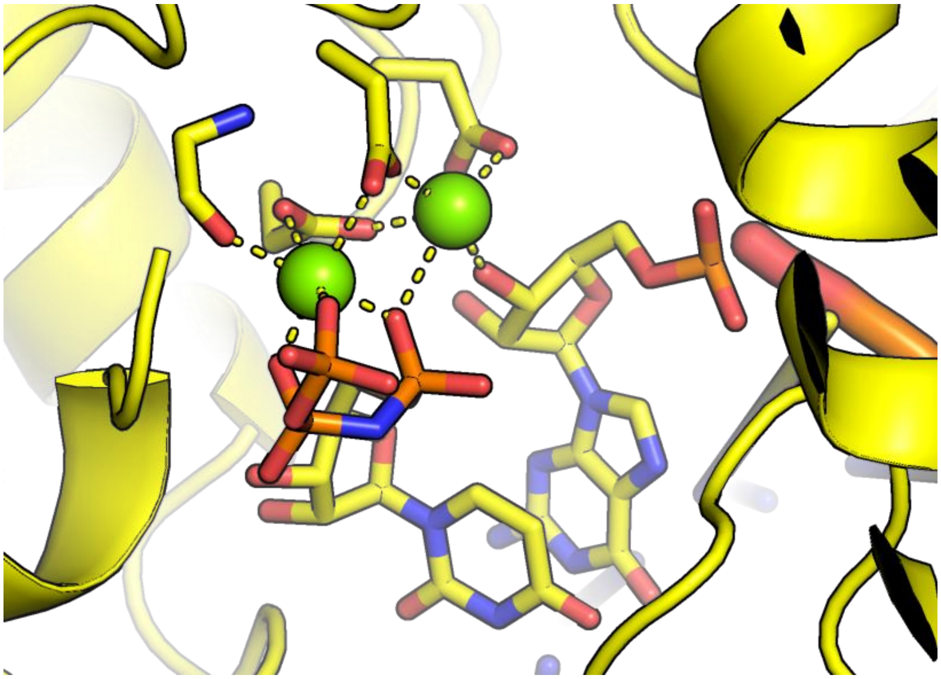
NTP, RNA and metal ion coordination in the representative structure 6T0V.

RNA-dependent RNA polymerase, eukaryotic-type (IPR007855)

This entry represents various eukaryotic RNA-dependent RNA polymerases, such as RCRP-1, RDRP-2 and RDRP-6. These enzymes are involved in the amplification of regulatory microRNAs during post-transcriptional gene silencing.[134] They catalyze RNA-template-directed extension of the 3′- end of an RNA strand by one nucleotide at a time and can initiate a chain *de novo*. There is no corresponding SUPFAM category, this group is named at the InterPro family level (IPR007855). The consensus coordination displays a typical polymerase arrangement, one Mg^{2+} coordinated by all three phosphate groups, and another that is coordinated by the 3′ hydroxyl of the priming nucleotide and the α-phosphate of the incoming NTP.

Pre-steady-state kinetic dissection of poliovirus 3Dpol reveals Mg^{2+}-dependent conformational change is rate-limiting for correct NTP incorporation; kpol and Kd(NTP) measured directly; Mg^{2+} concentration modulates fidelity and rate of phosphoryl transfer, establishing essential role of the two-metal-ion mechanism in RNA synthesis.[135]

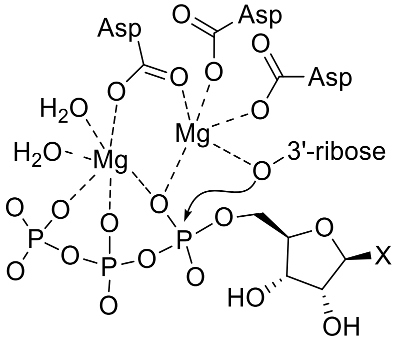

RNA polymerization and typical ion coordination.

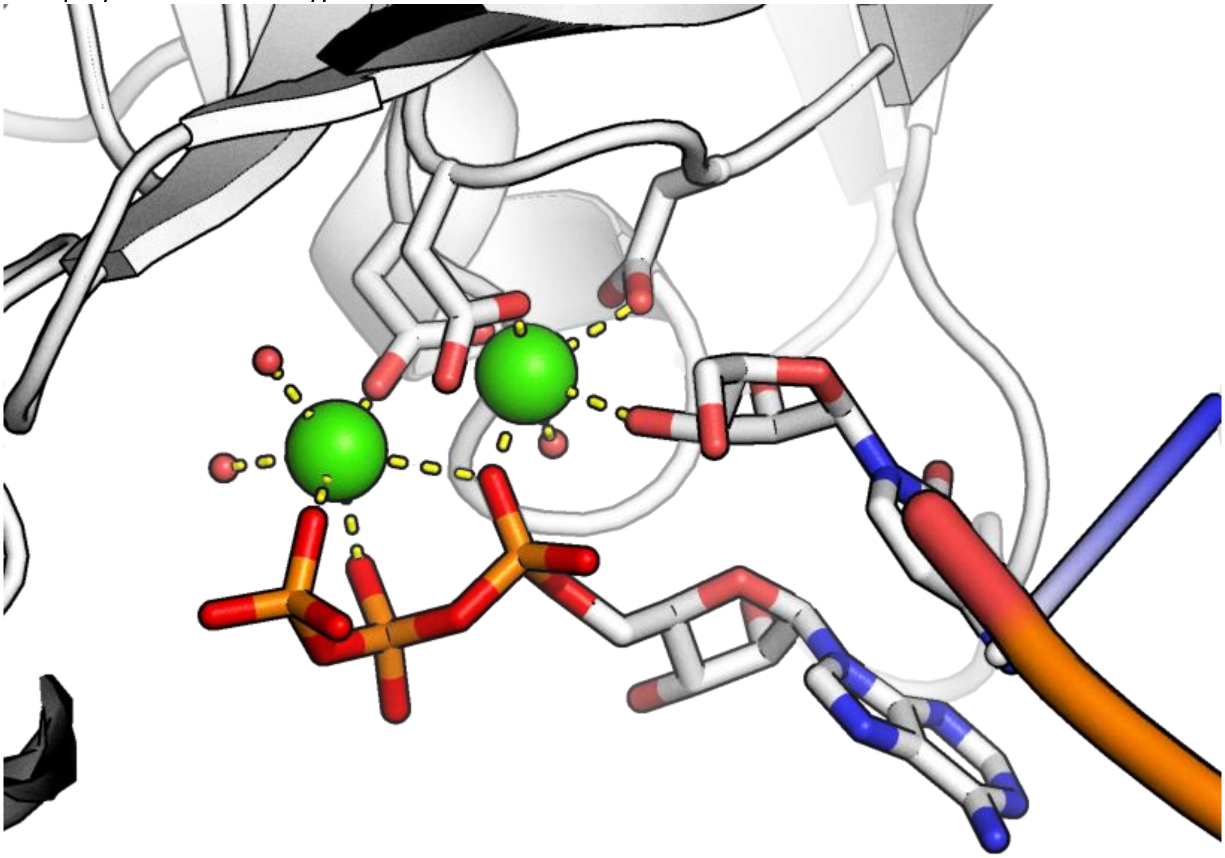

NTP, RNA and metal ion coordination in the representative structure 7Y7P.

Pyrophosphatases with αβ coordination on the (-) side

Fic-like

This superfamily has one Mg^{2+} ion which is coordinated by αβ phosphate oxygen atoms, and a nearby glutamate (sometimes aspartate) residue.

Crystal structures of the VopS Fic domain in complex with Mg·PPi show that Mg^{2+}, coordinated by the conserved active-site Asp and the β/γ-phosphate oxygens of the ATP-derived leaving group, is an obligate component of the AMPylation active site; kinetic analysis demonstrates a sequential mechanism in which MgATP is the nucleotide substrate, and mutagenesis of the metal-coordinating Asp abolishes all AMPylation activity.[136]

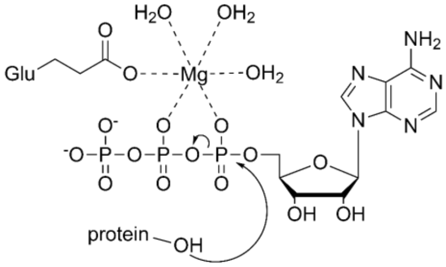

Protein adenylyltransferase activity, typical to Fic-like enzymes in EC 2.7.7.108. Further adenylation reactions may also be catalyzed (e.g. EC 2.7.7.1).

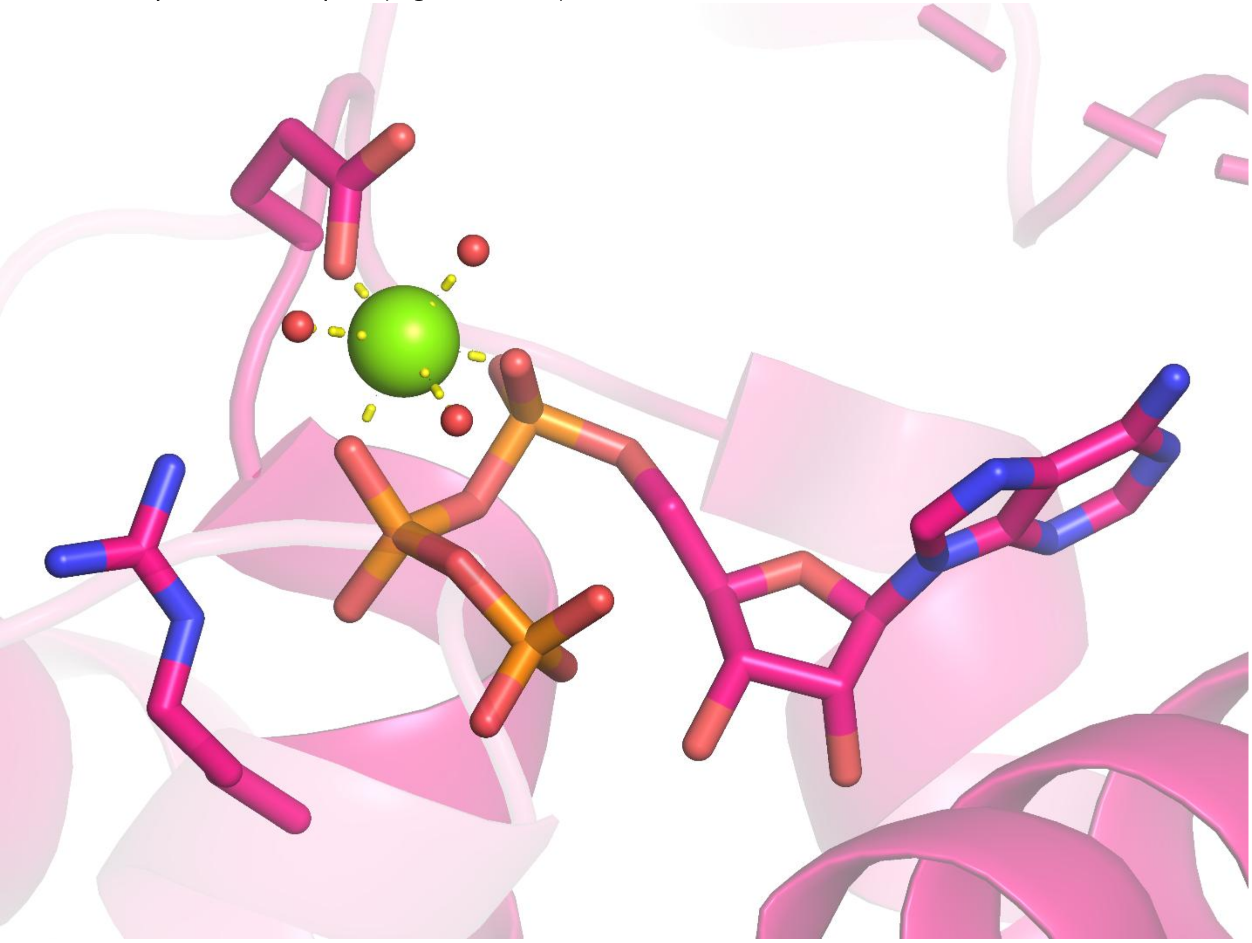

ATP and metal ion coordination in the representative structure 3ZCB.

Intramembrane CDP-alcohol synthase

Known ECs: 2.7.7.41 (phosphatidate cytidylyltransferase, bacterial CdsA), 2.7.7.67 (CDP-2,3-bis-(O-geranylgeranyl)-sn-glycerol synthase, archaeal CarS). Additional predicted members include the eukaryotic CTP-dependent lipid kinases in EC 2.7.1.108 (dolichol kinase), 2.7.1.174 (CTP-dependent diacylglycerol kinase DGK1), 2.7.1.182 (phytol kinase), and 2.7.1.216 (farnesol kinase).

The key structure found for this superfamily has a single Mg^{2+} ion is present that is coordinated by oxygens of the αβ phosphate groups.[137] There are additional 3 water molecules coordinating the Mg^{2+} ion. The lipid acceptor (archaeol/2,3-bis-(O-geranylgeranyl)-sn-glycerol-1-phosphate) is absent from the crystal. Site-directed mutagenesis of active-site residues and substrate-binding affinity analyses confirm that Mg^{2+} is essential for CTP binding and for the phosphodiester transfer reaction producing CDP-archaeol, establishing Mg^{2+} as an obligate cofactor for archaeal ether lipid biosynthesis.[137] Another apo structure is 4Q2G.

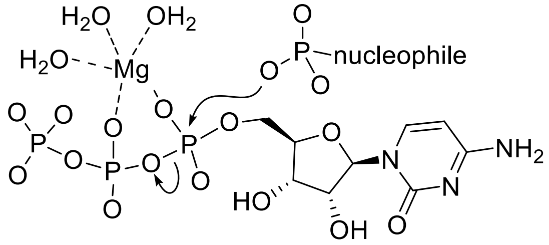

Cytidylyl transfer reaction for EC 2.7.7.67.

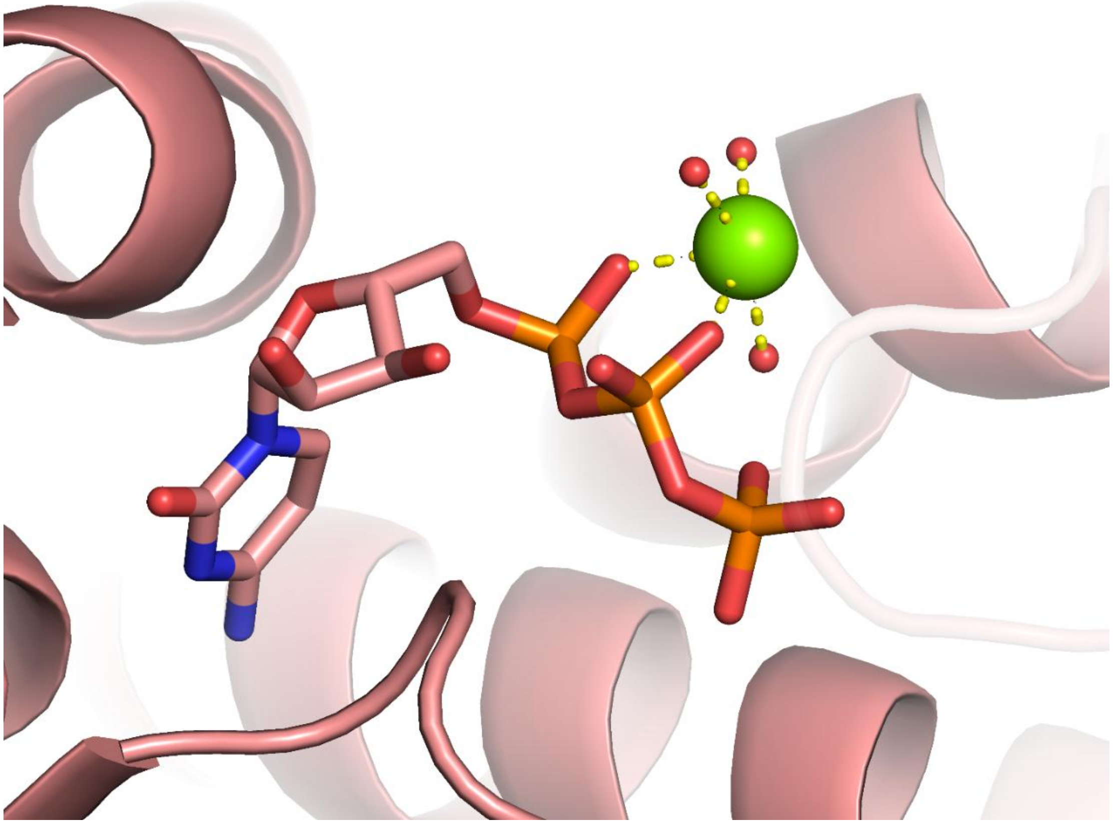

CTP and metal ion coordination in the representative structure 5GUF.

Note on divergent Phytol kinases:
We identified a divergent family of phytol kinases via a cluster analysis of sequences associated with EC 2.7.1.182 (see SI Section 4.), it also matches sequences from the InterPro family IPR039606. No structural data is available and no further biochemical experiments describe this family. AlphaFold predictions suggest ABG ion position, however, the CTP substrate molecule and the Mg^{2+} ion are not coordinated strongly by the protein and potentially an additional protein chain is required for catalytic activity (see also Figure S8, left).

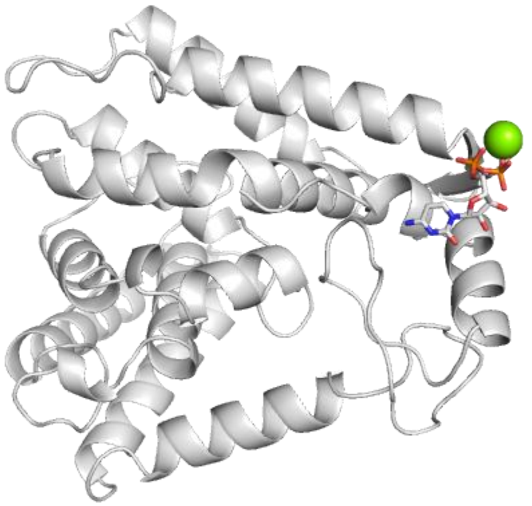

AlphaFold 3 predicted structure with CTP and Mg^{2+}.

Molybdenum cofactor biosynthesis proteins
There is one available (PDB 4CTA) that contains ATP, and several other structures (PDB 5ERR, 5ERT, 5ERM, 5ERV, 6FGD, 6HSU, 6HSO) were resolved in the presence of ADP. Using these structures and the selected representative PDB 4CTA, a clear αβ-coordination can be identified for the Mg^{2+}, which is also coordinated by an aspartate residue and 3 water molecules. The aspartate that binds the Mg^{2+} is absolutely conserved in CinA sequence alignments.[138]
Reconstitution of molybdenum cofactor biosynthesis in vitro requires Mg^{2+} at two sequential steps: MogA adenylates molybdopterin in a reaction that is strictly Mg·ATP-dependent, and MoeA then catalyzes molybdate ligation to the MPT-AMP intermediate in a Mg^{2+}-dependent manner, establishing an absolute requirement for magnesium throughout the final stages of Moco assembly.[139]

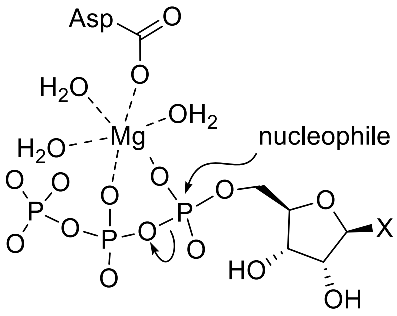

Proposed metal ion coordination and reactivity. In the associated ECs (2.7.7.75, 2.7.7.76, 4.6.1.17), different nucleosides all release pyrophosphate.

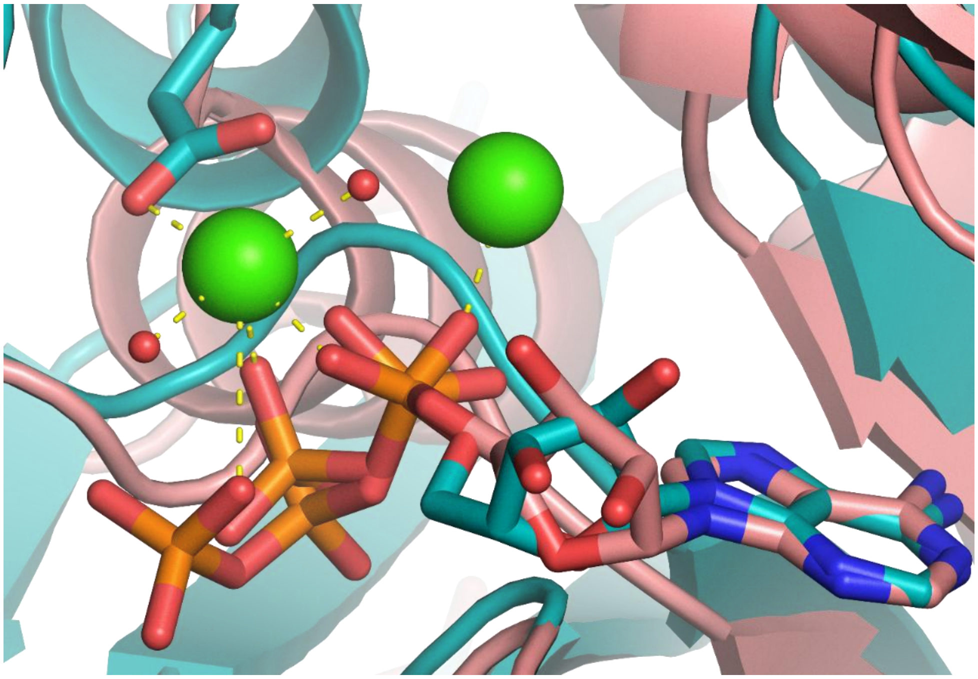

Nucleoside and metal ion coordination in the overlay of the ATP bound structure (4CTA, teal) and an ADP bound structure (salmon, 6FGD).

Adenylylcyclase toxin (the edema factor)

Edema factor (EF) is a component of anthrax toxin produced by Bacillus anthracis.[140] It is a calcium- and calmodulin-dependent adenylyl cyclase (EC 4.6.1.1) that significantly increases host intracellular cAMP levels, causing edema (fluid-filled swelling) and interfering with host intracellular signaling. The C-terminal region of EF contains the calmodulin-dependent activation domain and the catalytic site.[141] The cyclase reaction produces inorganic PP_i. The consensus coordination shows a Mg^{2+} ion coordinated by the α and β phosphates, further coordinated by two aspartate residues and one histidine residue.

The adenylyl cyclase activity of EF is inhibited by competition of Ca^{2+} with Mg^{2+} at the catalytic site; Mg^{2+} is essential for adenylyl cyclase activity; low Ca^{2+} activates via calmodulin, but high Ca^{2+} directly inhibits by displacing catalytic Mg^{2+}.[142]

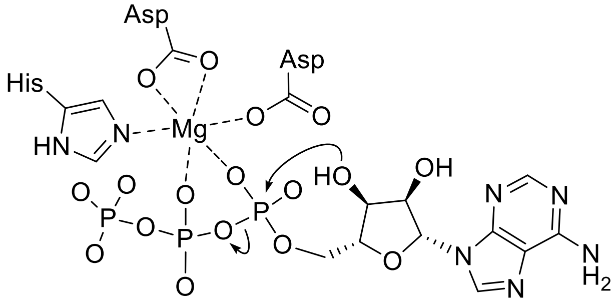

ATP cyclase activity and ion coordination based on the representative structure 1K90.

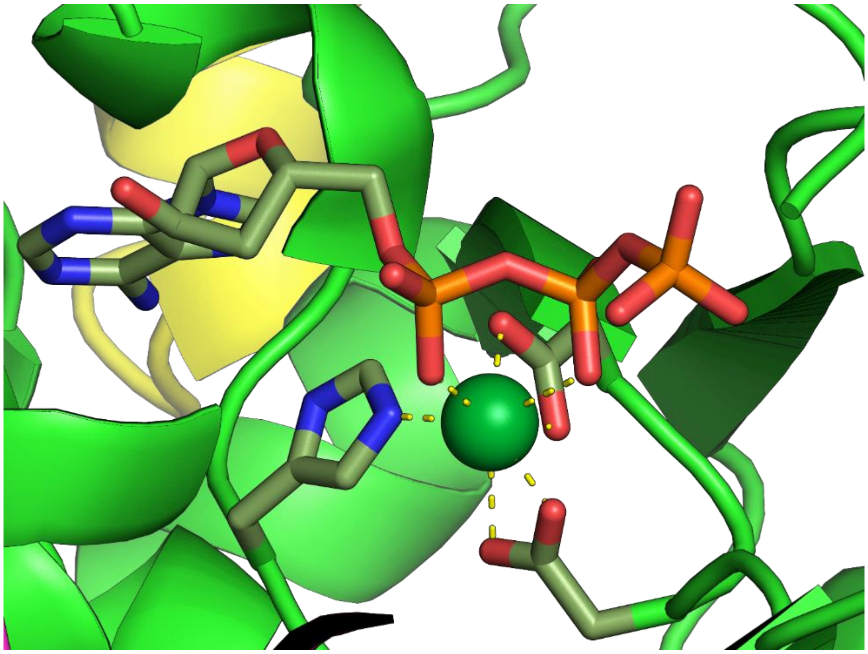

ATP and metal ion coordination in the representative structure 1K90. The structure is resolved with Yb^{3+}, which reduces cyclase activity.

DNA primase core

In our dataset only a few structures are found for this superfamily, some originating from the same study. The structures have 2 catalytic divalent metal ions. One is coordinated by the αβ and the other by the βγ phosphate group oxygens. The metal ion in the αβ position is coordinated by two aspartates, while one of the aspartates also coordinates the metal ion in the βγ position.

The magnesium ion binding site of E. coli primase has been precisely mapped to the catalytic centre by iron-substitution cleavage, identifying a cluster of conserved acidic residues within the DPD and EGYMD motifs as the metal ligands that coordinate the Mg^{2+} ion essential for primer RNA synthesis.[143]

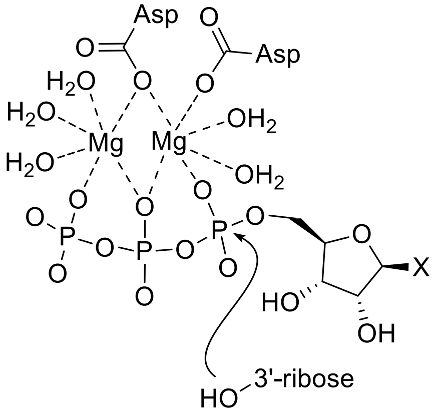

Primase activity (EC 2.7.7.101) and metal ion coordination scheme.

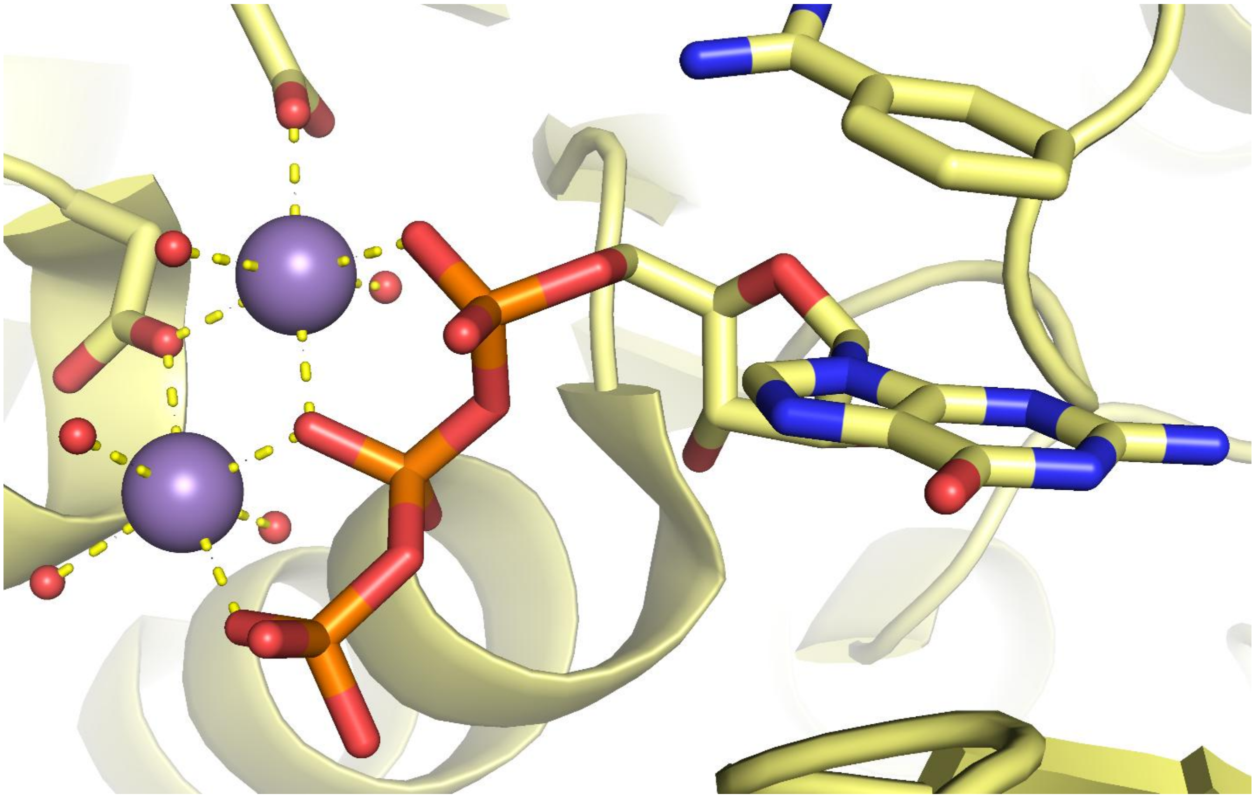

NTP and metal ion coordination in the representative structure 4EDK. Mn^{2+} was used for crystallization, the enzyme functions with both Mg^{2+} and Mn^{2+}.

Additional pyrophosphatases

Acetyl-CoA synthetase-like

The most significant exception to the Mg-pinch motif, this superfamily has one Mg^{2+} ion coordinated by the βγ phosphate oxygen atoms (there is no metal cation coordinating the αβ phosphate groups). We hypothesize that in the case of this superfamily, due to the negatively charged attacking group, the attack is more easily performed and does not necessitate the coordination between the αβ phosphates to hydrolyze ATP to AMP and pyrophosphate. A conserved glutamate residue can contribute to the second-shell coordination of the Mg^{2+} ion. Also, the superfamily was found to function with various ions[144] suggesting it has a role in the charge balance rather than activation.

Kinetic studies of the acetyl-CoA synthetase reaction demonstrate an obligate double requirement for divalent metal ions: Mg^{2+} is essential both for the adenylation half-reaction that forms the enzyme-bound acetyl-AMP intermediate and for the subsequent CoA thioesterification step, establishing Mg^{2+} as an obligate cofactor at each catalytic stage.[145]

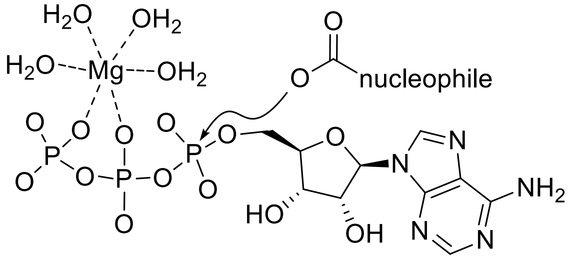

Carboxyl activation as a first step of ligase reactivity (EC 6.2.1.-) and non-pinching metal ion coordination.

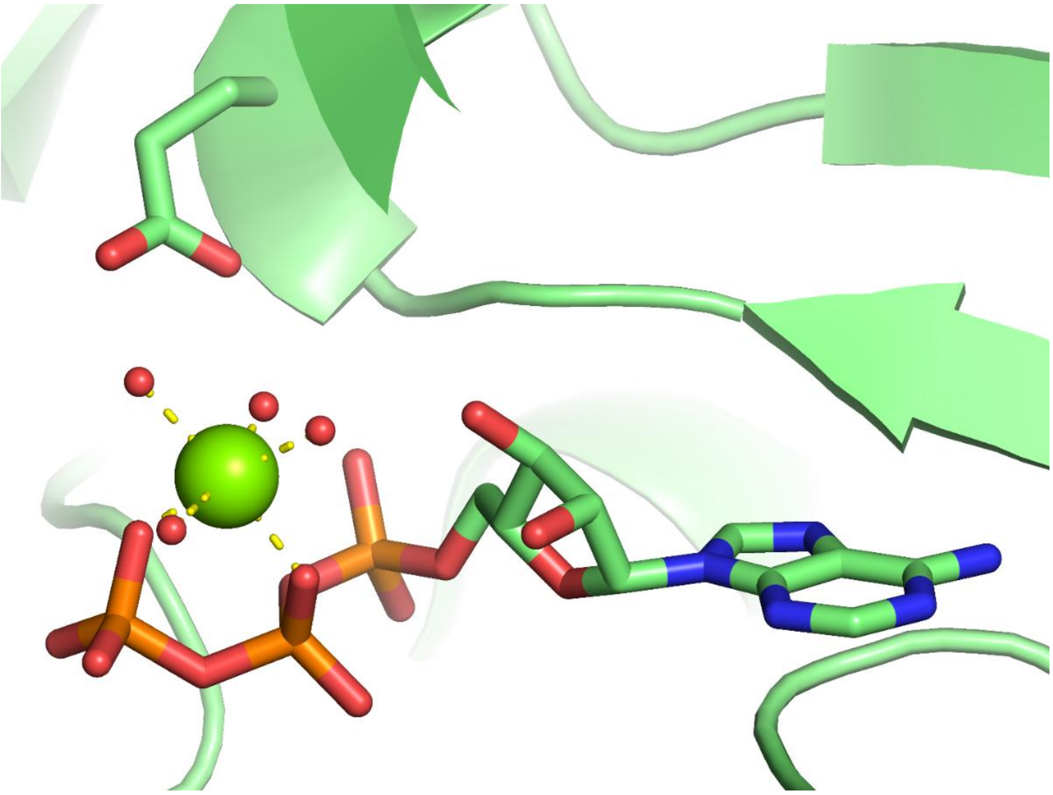

ATP and non-pinching Mg^{2+} coordination in the representative Ac-CoA synthetase-like structure 5BSM.

Alkaline phosphatase-like

Known ECs: 3.6.1.9

There is a family, ecto-nucleotide pyrophosphatase/phosphodiesterase (NPP) within the Alkaline phosphatase-like superfamily the members of which may catalyze ATP hydrolysis,[146] this family corresponds to the InterPro entry IPR002591 (Type I phosphodiesterase/nucleotide pyrophosphatase/phosphate transferase). The human NPP family can be classified into two groups according to their substrate preferences; the nucleotide-degrading proteins NPP1, 3 and 4, and NPP2, 6, and 7 that hydrolyze phospholipids or related molecules.[147] The catalytic center consists of two zinc ions held by seven protein side chains; the catalytic cycle of NPPs is initiated by binding of a substrate (e.g. ATP) to the zinc ions via its α-phosphate group.[148] Zn2 activates the catalytic threonine by greatly lowering its pKa[149] and allowing it to carry out a nucleophilic attack on the phosphorus atom, followed by departure of a leaving group[150] facilitated by Zn1.[147]

The Mg^{2+} ion in the M3 site is required for full enzyme activity; a strong correlation exists between occupancy of this Mg^{2+}-binding site and the conformation of Ser102 nucleophile, clarifying why Mg^{2+} in the third metal site is required for catalysis.[151]

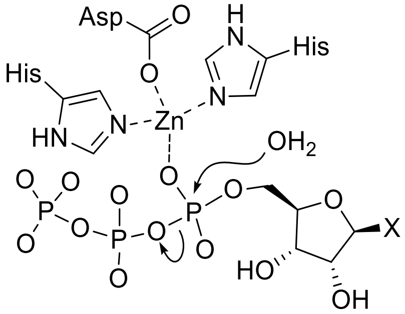

Zn^{2+} coordination and proposed pyrophosphate hydrolysis.

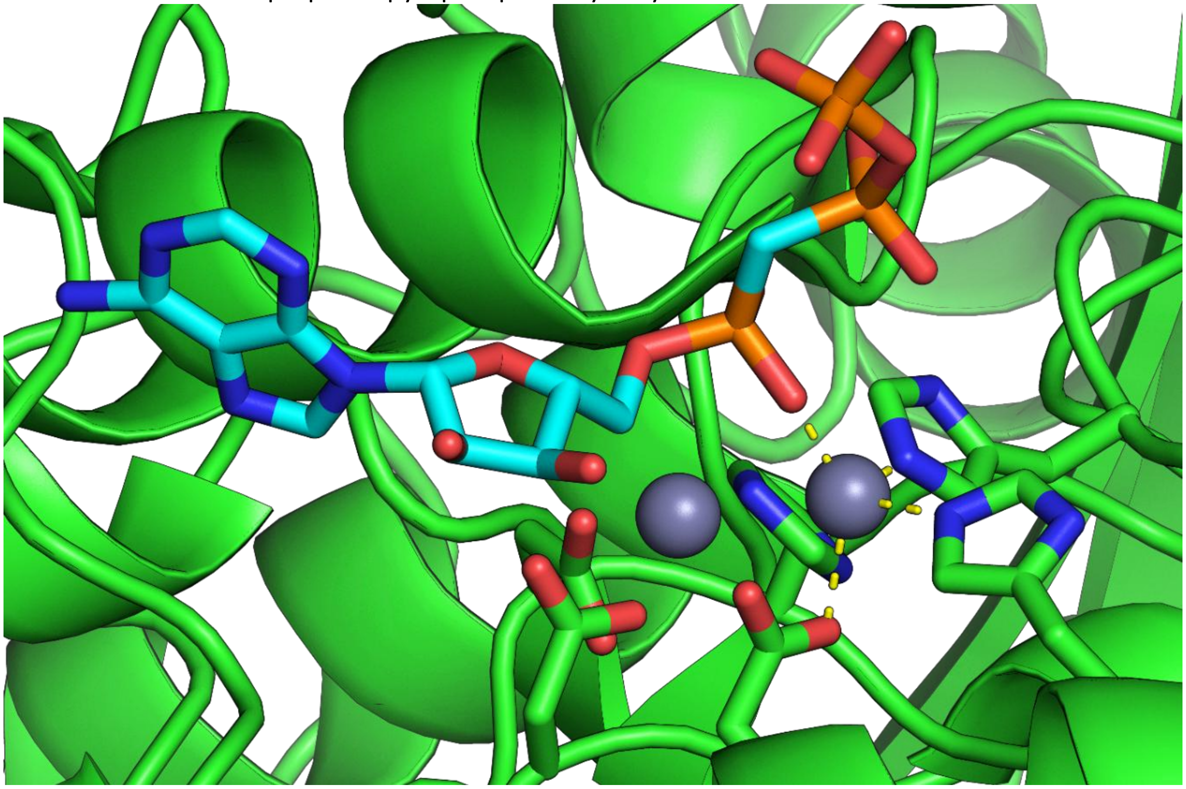

Active site structure of 6C02.

Thiamin pyrophosphokinase, catalytic domain

Known ECs: 2.7.6.2, 2.7.6.3

In the available structures we identified for this superfamily already the product state is present with thiamine diphosphate and AMP. Yet, based on the position of the Mg^{2+} the available product structures it seems clear that there is an αγ-coordinated Mg^{2+} (as seen e.g. in PDB 2F17), which might be αβγ before the reaction. Furthermore, based on other structures (where the AMP is missing, yet other Mg^{2+} ions are present) we hypothesize that a second, βγ-coordinated Mg^{2+} may be present at the active site. There are altogether 4 aspartate residues coordinating the metal ions.

The reaction requires MgATP; plots of initial velocity vs MgATP show sigmoidal character when Mg^{2+}/ATP=1, but addition of excess Mg^{2+} restores activity — demonstrating that free Mg^{2+} is required in addition to the MgATP complex.[152]

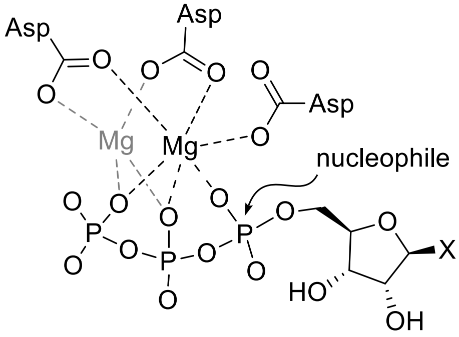

Hypothetical Mg^{2+} coordination and reaction based on product structures.

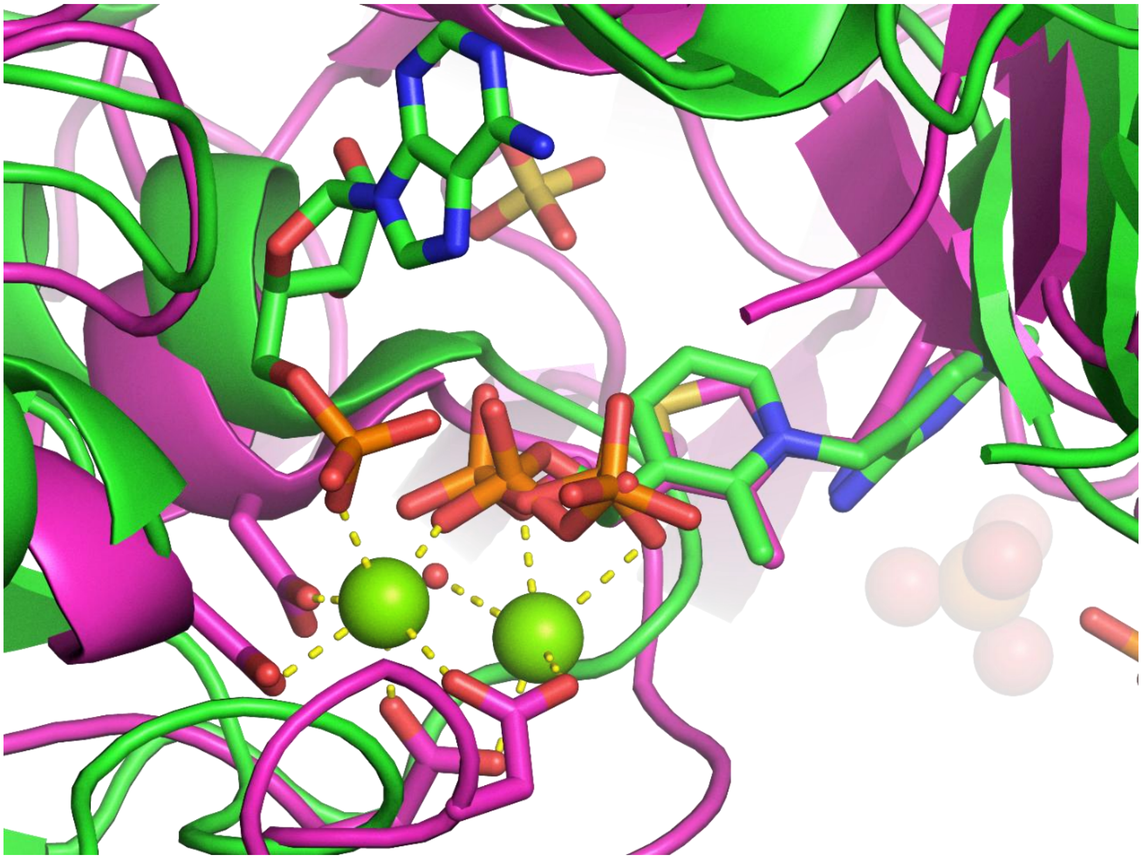

Active site reconstruction overlaying the AMP bound structure 2F17 and the pyrophosphorylated product 3IHK.

DNA ligase/mRNA capping enzyme, catalytic domain

A two metal-ion mechanism was proposed to occur for the DNA and RNA ligases of this superfamily. A first Mg^{2+} ion may facilitate the precise positioning of the β and γ-phosphate groups, while another Mg^{2+} ion, which is not directly coordinating the cleavage of the bond between the departing PPi group and the rest of the ATP. It binds to the α-phosphate group of the ATP and promotes the formation of a covalent bond with a conserved lysine residue (such a second Mg^{2+} ion is not present in the representative structure)[153-155] In our dataset, the position of a divalent metal ion binding to the α-phosphate group of ATP is well conserved, however most probably due to the lack of catalytically active complexes as the DNA or RNA is missing in the structures, the triphosphate chain of the ATP has a large degree of freedom and shows considerable conformational variability together with the other metal ion in the βγ position. There is no metal ion neither in the αβ or αβγ position in the structures in our dataset, however we cannot exclude that this is due to the lack of the presence of the DNA/RNA ligand.

Human DNA Ligase I requires multiple Mg^{2+} ions for catalysis, with an essential Mg^{2+} binding more tightly to ATP than to the enzyme; the affinity for Mg^{2+} changes along the reaction coordinate affecting ligation efficiency.[156]

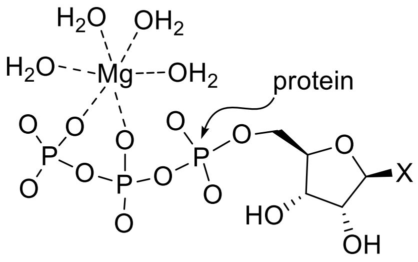

Protein adenylation as the first step of DNA ligase activity. The ion coordination is adopted from structure 1XDN.

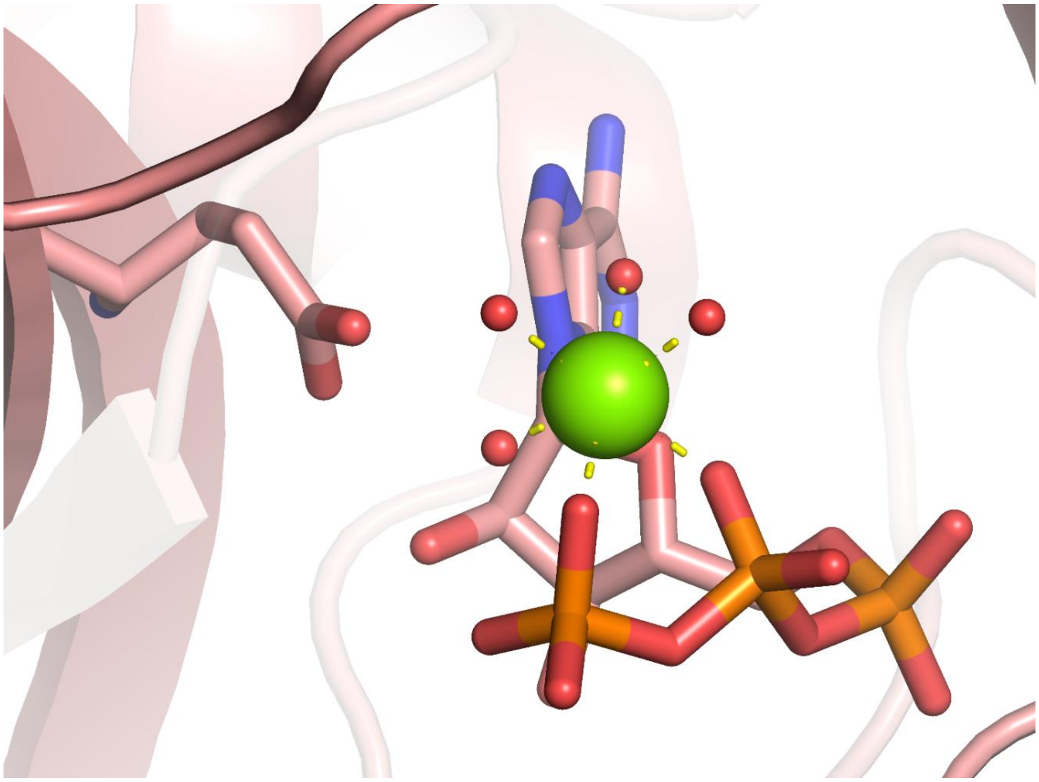

ATP and Mg^{2+} coordination in the structure 1XDN.

RibA-like

There is only one available structure with a triphosphate chain for this superfamily (PDB 2BZ0). Due to the usage of a GTP analog, GMPcPP, it is not evident to deduce the metal ion coordination, yet the most probable coordination seems to be αβ.

GTP cyclohydrolase II (RibA) catalyzes the first committed step of riboflavin biosynthesis, converting GTP to 2,5-diamino-6-ribosyl-4(3H)-pyrimidinone 5′-phosphate, formate, and pyrophosphate; a catalytic Zn^{2+} ion activates a water molecule that—oriented via a hydrogen bond to Tyr105—attacks C-8 of the guanine ring to initiate ring opening, Arg128 then acts as the nucleophile forming a transient covalent guanylyl-GCH-II intermediate, and Mg^{2+} is required for pyrophosphate release and overall turnover.[157]

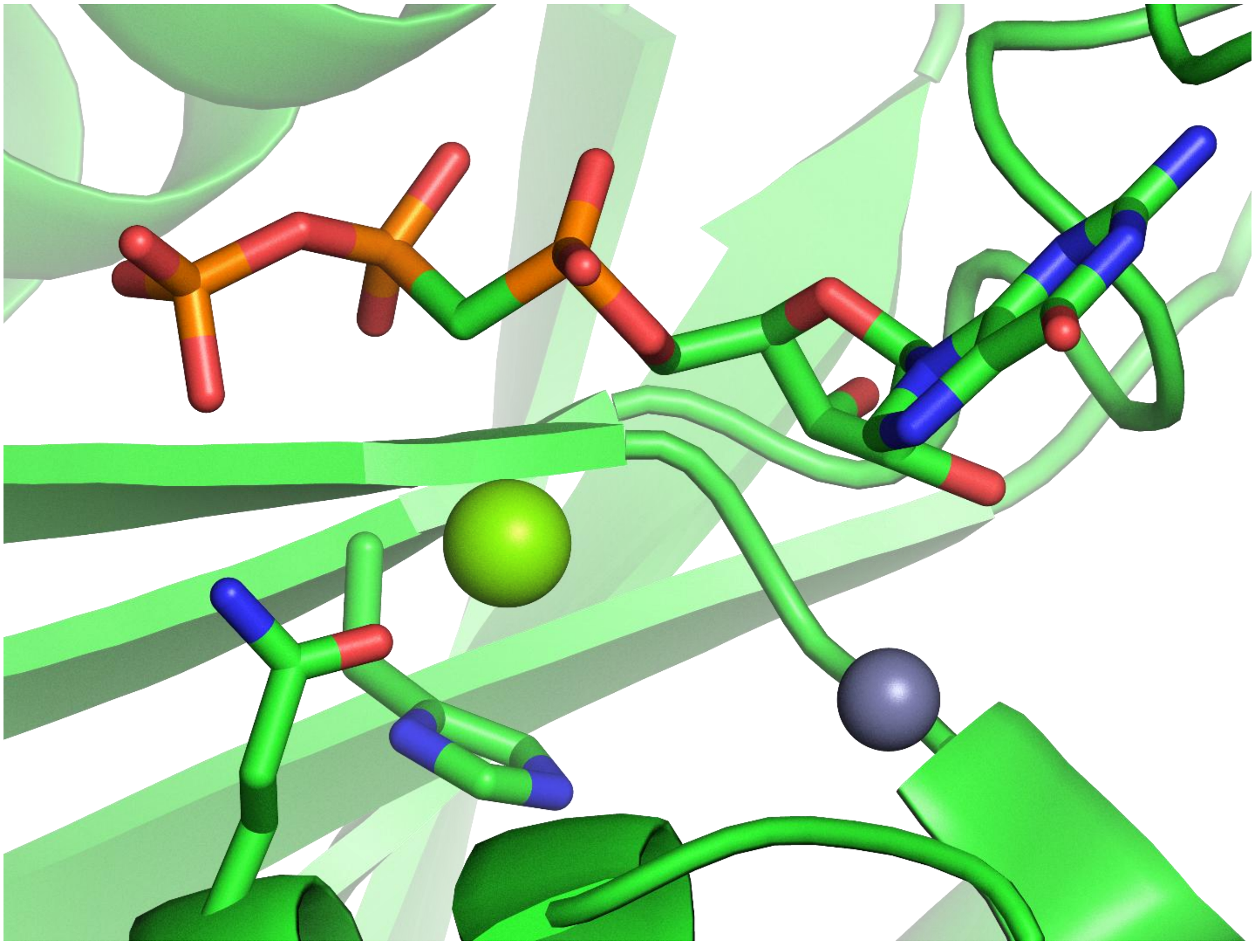

Active site structure of 2BZ0.

BioW-like

The pimeloyl-CoA synthetase BioW (EC 6.2.1.14, InterPro IPR005499, SCOP ID 3002146) catalyzes the first committed step of biotin biosynthesis in many bacteria, activating pimelate via an adenylated intermediate before its transfer to coenzyme A: pimelate + ATP + CoA → pimeloyl-CoA + AMP + PPi. BioW is structurally distinct from all other adenylate-forming enzymes and was proposed to define a new fold for this class of pyrophosphatases.[158]

The representative postcatalytic complex (PDB 5FLL, *B. subtilis* BioW) contains the pimeloyl-adenylate intermediate, the pyrophosphate leaving group, and two Mg^{2+} ions per active site (Mg–Mg distance 5.06 Å). The two Mg^{2+} ions sit on opposite faces of the α-phosphate, each bridging the AMP α-phosphate and both phosphates of the pyrophosphate via short (≤ 2.2 Å) inner-sphere contacts, and differ in their non-substrate ligands: one is coordinated by two conserved protein aspartate carboxylates (Asp195 and Asp196) and one water on the protein face of Pα, while the other is exposed to solvent and completed by three water molecules. A complementary precatalytic complex (PDB 5TV8, *A. aeolicus* BioW with the non-hydrolyzable analog AMP-CPP, pimelate, and a single Mg^{2+}) shows the metal positioned between the α and β phosphates of the intact ATP analog. Given the consistent presence of the protein-coordinated metal and the conservation of its aspartate ligands across both structures, this ion is most likely the catalytic Mg^{2+}, while the second, water-coordinated metal seen only in the postcatalytic complex is most plausibly a postcatalytic spectator. The SF is therefore tentatively assigned as AB(+) or ABG(+) given the lack of direct mechanistic and complementary structural information for this SF.

Catalytic activity of BioW has been directly demonstrated in multiple organisms.[158-159] Activity is abolished by EDTA and varies linearly with [Mg^{2+}]; Mn^{2+} and Co^{2+} partially substitute for Mg^{2+} but Ca^{2+} does not, confirming a strict divalent-metal requirement at the active site. Unfortunately, however, these experiments did not verify whether one or two metal ions are required.

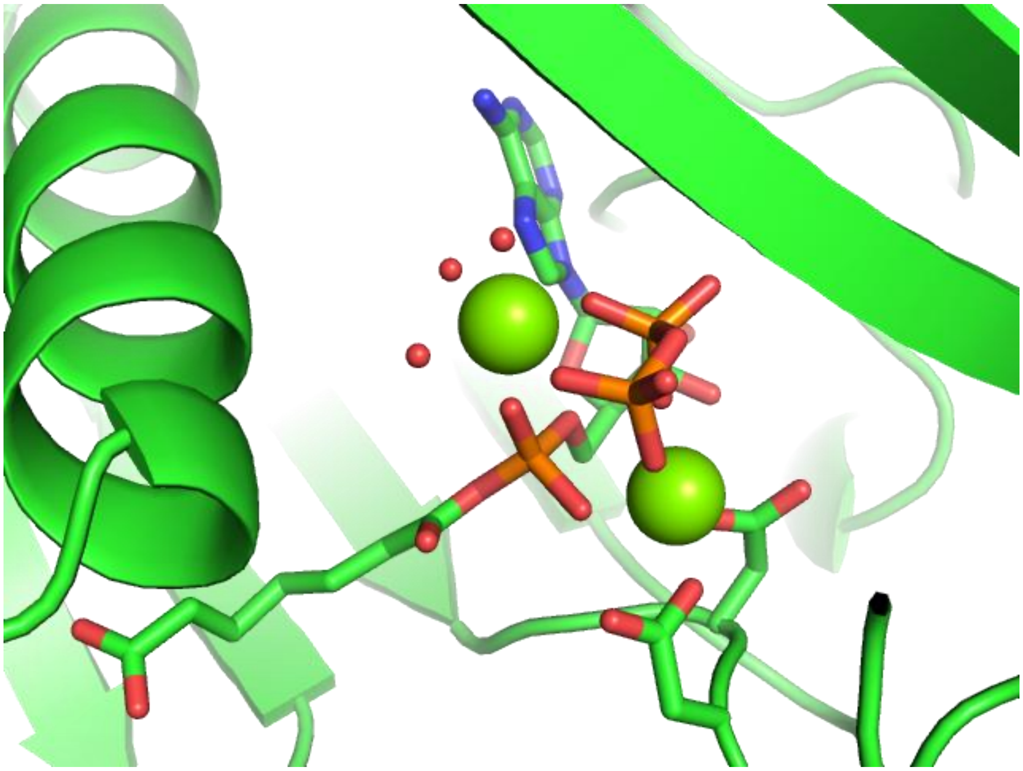

ATP and two Mg^{2+} ion coordination in the representative structure 5FLL.

12. Other superfamilies without sufficient structural information:

Glycerate kinase I

Known ECs: 2.7.1.31 (`glycerate 3-kinase`), 2.7.1.165 (`glycerate 2-kinase`)

Similar folds, but low sequence identity, no structure with phosphates.[160] The AlphaFold 3 prediction agrees with the position of the inorganic phosphate, but suggests an atypical AB coordination for the Mg^{2+} cation.

Class I glycerate kinase requires the MgATP complex as co-substrate, with ATP as the preferred phosphoryl donor and Mg^{2+} as the essential divalent cation for phosphoryl transfer activity. [161]

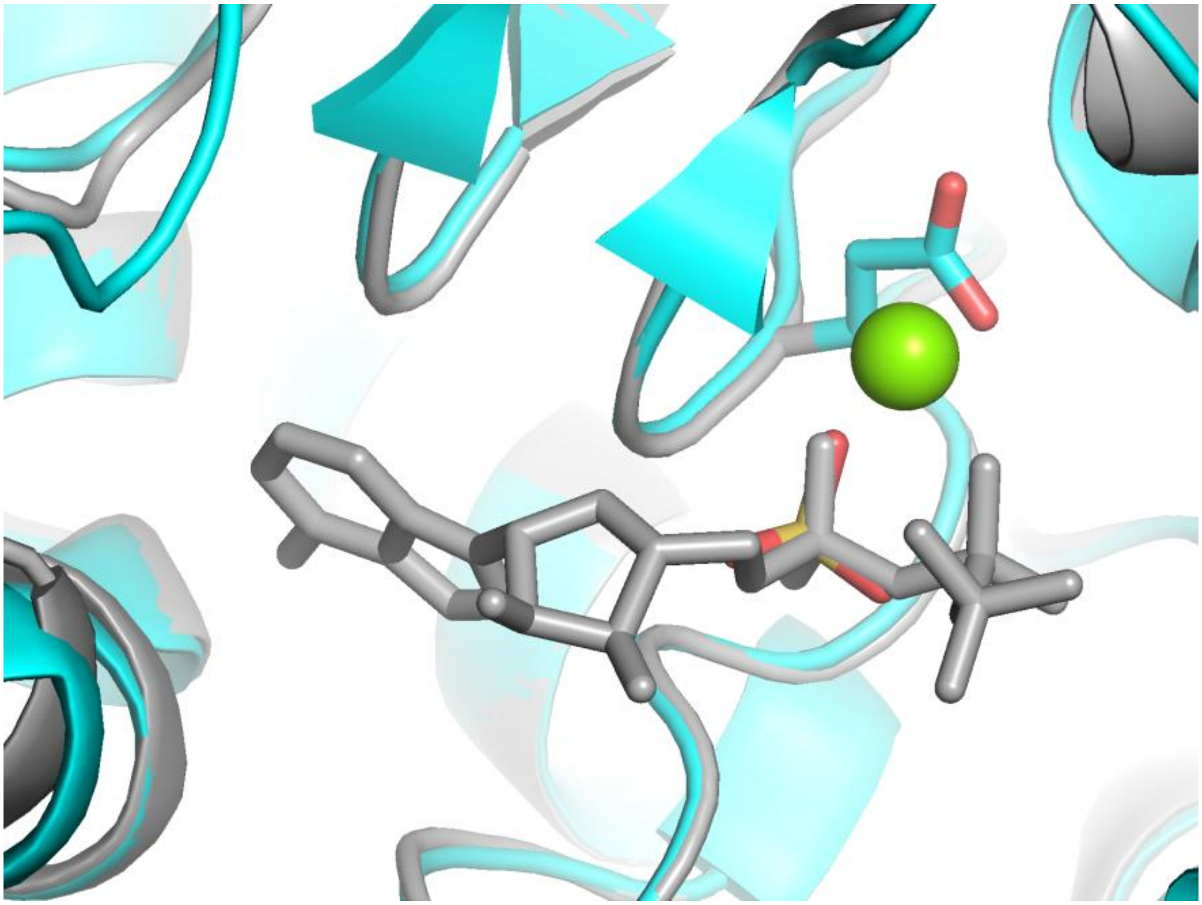

AlphaFold 3 predicted structure with ATP and Mg^{2+} (grey) and the template 1TO6 (cyan).

YgbK-like

Known ECs: 2.7.1.217, 2.7.1.219, 2.7.1.220, 2.7.1.231

There are only seven PDB structures associated with the InterPro entry Four-carbon acid sugar kinase, nucleotide binding domain superfamily (IPR042213). They align well, at their active sites an ADP and no ions are present. AlphaFold 3 predicts ABG coordination in and a crowded NTP site.

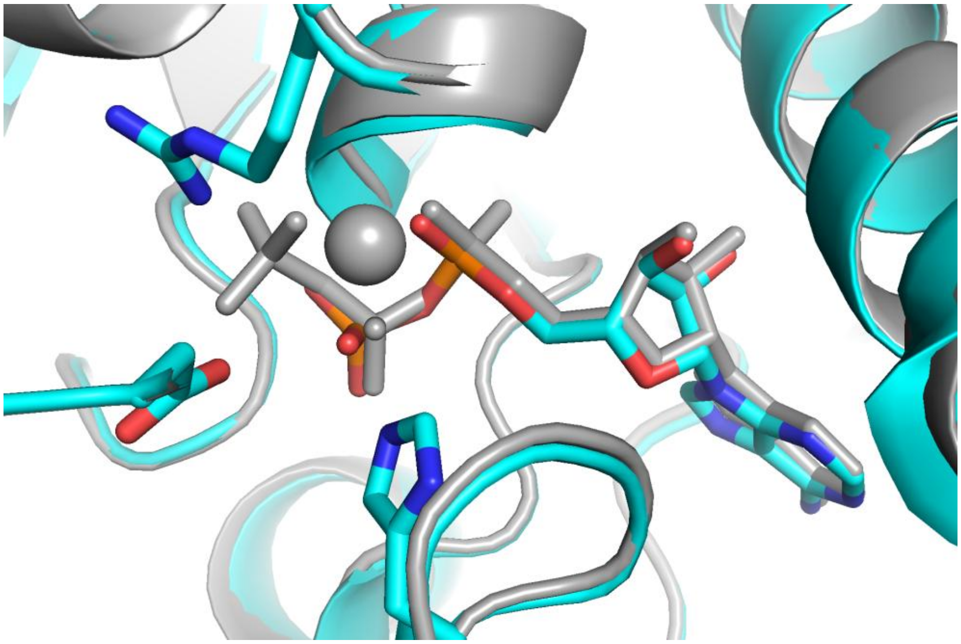

AlphaFold 3 predicted structure with ATP and Mg^{2+} (grey) and the template 5DMH (cyan, with ADP, without Mg2+).

NagB/RpiA/CoA transferase-like

Known EC: 6.3.3.2 (`5-formyltetrahydrofolate cyclo-ligase`)

The only available structures are in the presence of ADP with αβ coordinated ions, hence the ATP binding could not be determined.

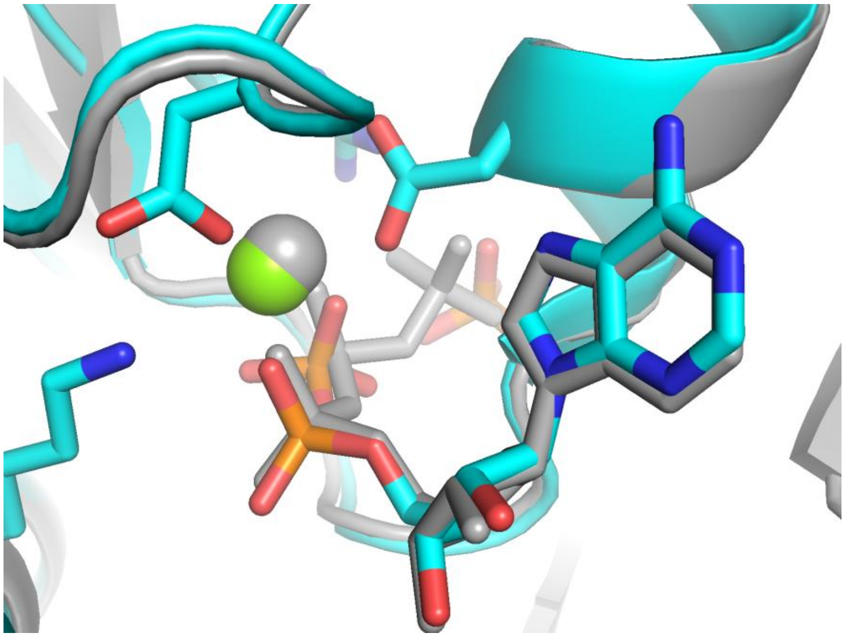

AlphaFold 3 predicted structure with ATP and Mg^{2+} ions (grey) and the template 2JCB (cyan).

HIT-like

Known ECs 2.7.7.10, 2.7.7.53, 3.6.1.17, 3.6.1.29

The HIT-like SF covers the synthesis and breakdown of bisadenosyl tri- and tetraphosphates and similar substrates that are uncommon triphosphate containing molecules. The metal ion dependence of these enzymes is unclear. While Mg^{2+} is reported to enhance catalytic activity,[162] bound ions are not resolved in the available structures (except for a K+ ion of a mutant HIT-like enzyme, 1GUP), and some works suggested that divalent metal ions are not essential for catalytic activity.[163]

FHIT catalyzes Mg^{2+}-dependent hydrolysis of diadenosine triphosphate (Ap3A) to AMP and ADP, and dinucleoside polyphosphate hydrolases in general require millimolar Mg^{2+} or Mn^{2+}—which are equally stimulatory—for optimal activity, while Zn^{2+} is inhibitory. [164]

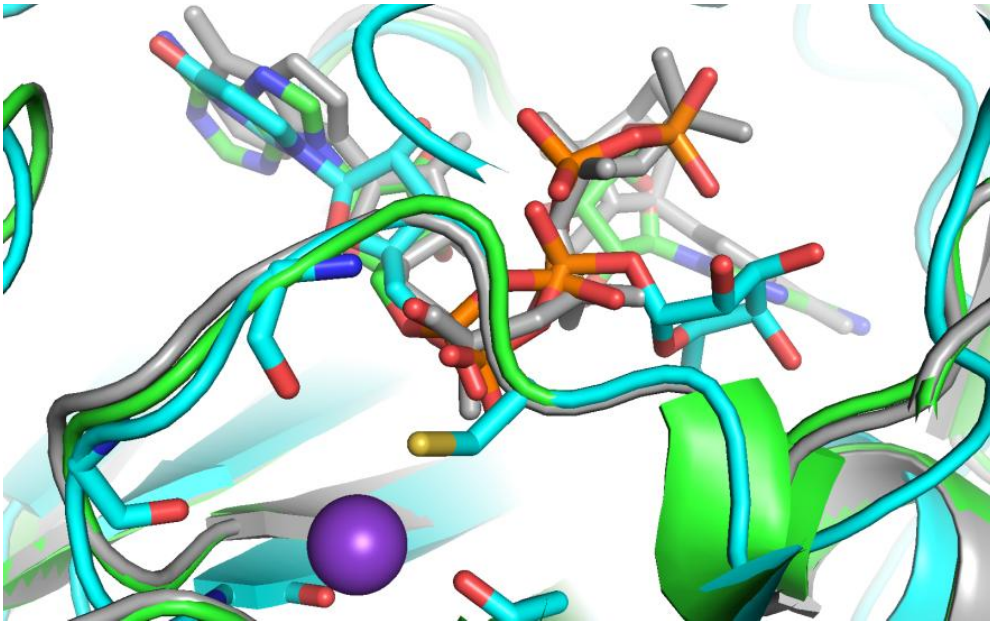

AlphaFold 3 predicted structure with the substrate diadenosine 5',5′′'-P(1),P(4)-tetraphosphate Ap4A (grey) and the template 4I5V (green), as well as 1GUP (cyan). Potassium (purple sphere) and nearby ligands (sticks) are shown for 1GUP.

tRNA(Ile2) 2-agmatinylcytidine synthetase TiaS

Known EC: 6.3.4.22

Unfortunately for this superfamily the available structures, one in the presence of ATP (PDB 3AMT) and three structures resolved with the ATP analog, AMPCPP (PDBs 3AMU, 4RVZ, 6AGG), do not enable us to determine the corresponding metal ion coordination. The former two PDBs lack the coordinating metal ion whereas the quality of the latter two is not sufficient for our analysis. Other class I aminoacyl tRNA synthetase enzymes feature ABG coordination (e.g. LeuRS, 7NU0), which is also in agreement with the AlphaFold 3 prediction.

TiaS, the archaeal tRNA C34 modification enzyme, forms a ternary ATP–agmatine–Mg^{2+} complex in the active site; Mg^{2+} stabilises the ATP molecule and coordinates the TCKD domain to enable the C34 phosphorylation step required for biosynthesis of agmatidine in archaeal tRNA-Ile2. [165]

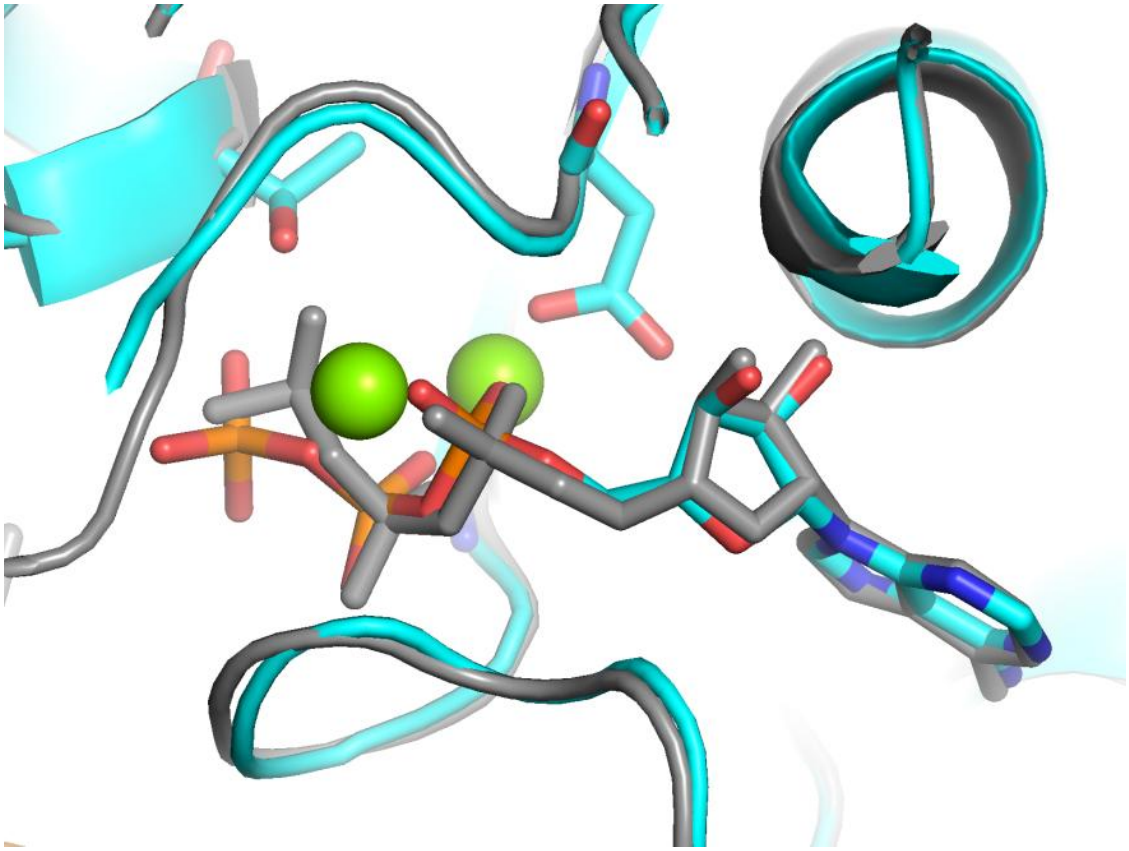

AlphaFold 3 predicted structure with ATP and Mg^{2+} ions (grey) and the template 3AMT (cyan).

CTP-dependent diacylglycerol kinase 1-like (IPR037997)
Phytol/farnesol kinase (IPR039606)

Known ECs: 2.7.1.174, 2.7.1.182, and 2.7.1.216

We have not identified any available experimental structures for these families (defined via InterPro), it was not possible to deduce the corresponding metal ion coordination. However, AlphaFold 3 predicted structures aligned well to the *Intramembrane CDP-alcohol synthase* pyrophosphatase SF, we therefore do not consider these as novel SFs.

FomD-like

Known ECs: 3.6.1.15

This group of prokaryotic proteins includes Nucleoside triphosphate/diphosphate phosphatase from Staphylococcus aureus (SA1684 or Ntdp), Cytidylyl-2-hydroxypropylphosphonate hydrolase from Streptomyces wedmorensis (FomD), and Probable ribonuclease FAU-1 from Pyrococcus abyssi.[166] We identified two metal ions at the active site, one of which is coordinated by the P_β and the other by the P_γ.[166] Yet, the available structures do not make it possible to establish a clear consensus on the coordination.

FomD (characterized in *Streptomyces wedmorensis*) and its *Pseudomonas* ortholog PsfC efficiently hydrolyze cytidylyl-(S)-HPP in a binuclear metal-dependent reaction; Mn^{2+} and Co^{2+} support activity, whereas Mg^{2+} does not, establishing an atypical metal preference within the metallohydrolase superfamily.[167-168]

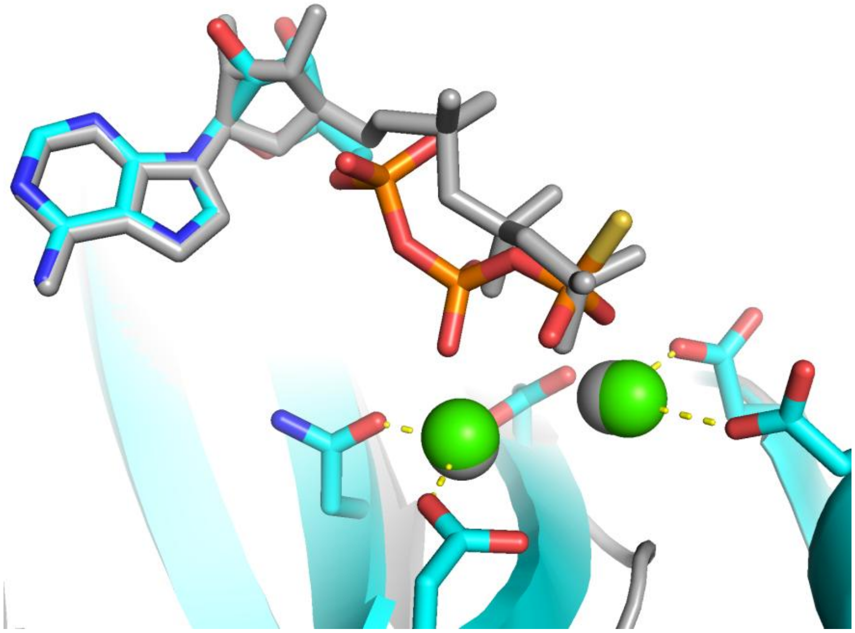
AlphaFold 3 predicted structure with ATP and Mg^{2+} ions (black) and the template 7D8I (cyan).

Phospholipase D/nuclease

There is one structure available with in the presence of NTP and metal ions at the active site (PDB 1xdp). Two Mg^{2+} ions are very (likely erroneously) close to each other (3.1 Å apart), one close to the α while the other close to the γ phosphate. Their coordination sphere is not complete. The AlphaFold 3 prediction mostly agrees with the NTP positioning.

Members of the phospholipase D (PLD) superfamily, which share a conserved HxKxxxxD catalytic motif, are activated by divalent cations including Mg^{2+}; in Serratia nuclease, a structurally related member, two Mg^{2+} ions in the active site coordinate the catalytic water molecule and the scissile phosphate to enable double-displacement catalysis. [169]

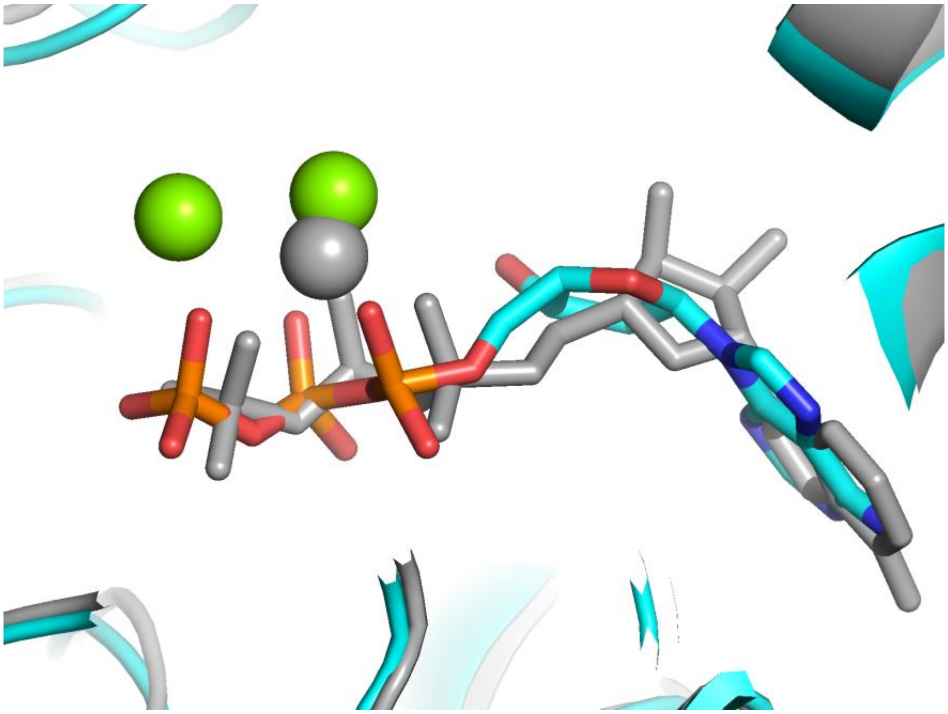

AlphaFold 3 predicted structure with ATP and Mg^{2+} (gray) and the template 1XDP (cyan).

Nicotinate/Quinolinate PRTase C-terminal domain-like

Known ECs: 6.3.4.21 (Nicotinate phosphoribosyltransferace)

The nicotinate phosphoribosyltransferase catalyzes the synthesis of beta-nicotinate D-ribonucleotide from nicotinate and 5-phospho-D-ribose 1-phosphate at the expense of ATP, even though it can also form β-nicotinate D-ribonucleotide and diphosphate from nicotinate and 5-phospho-α-D-ribose 1-diphosphate in the absence of ATP. Yet, the utilization of ATP results in a much lower K_m for nicotinate and shifts the reaction towards the products.[170] Unfortunately, there are no available structures in the presence of ATP, the coordination could not be established for this superfamily. The AlphaFold predictions are also ambiguous regarding both the ATP and metal ion positioning.

Quinolinate phosphoribosyltransferase (QAPRTase) catalyzes the Mg^{2+}-dependent phosphoribosyl transfer from PRPP to quinolinate, releasing CO_2 and PPi to yield nicotinate mononucleotide (NaMN), the entry point into the NAD^+ salvage pathway; steady-state kinetics reveal an ordered mechanism in which quinolinate binds first and MgPRPP is the catalytically active substrate species; Mg^{2+} is essential for coordinating the pyrophosphate leaving group of PRPP and stabilising the pentacoordinate phosphorus transition state.[171]

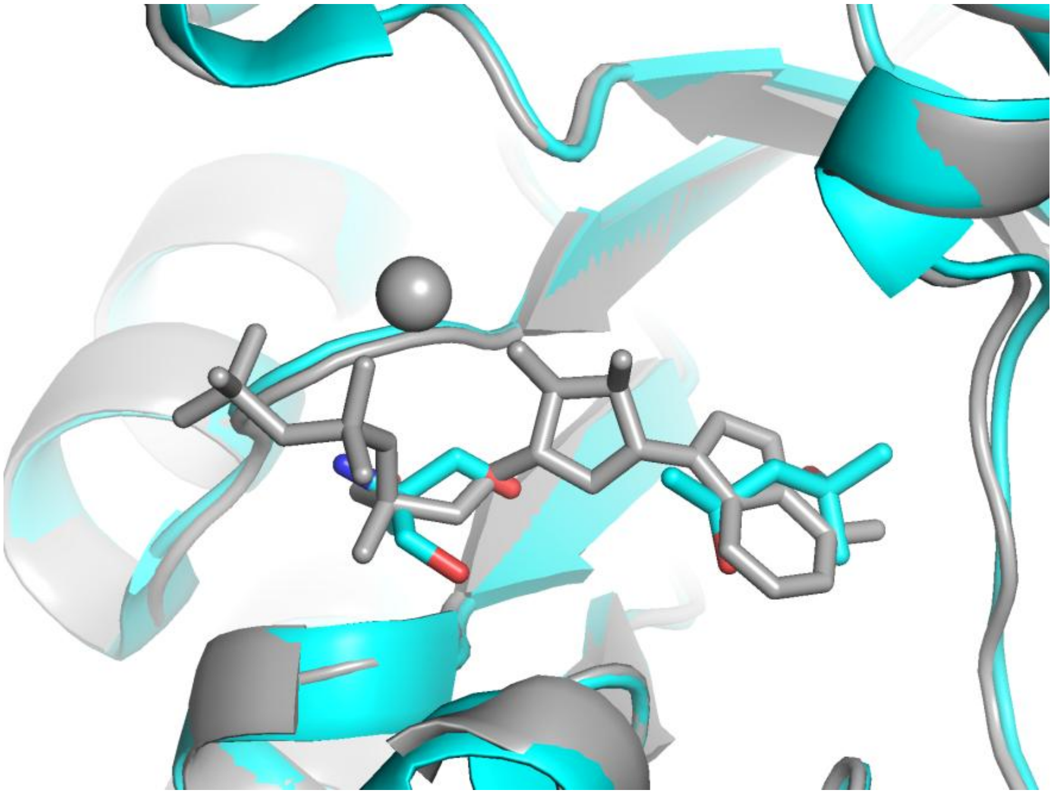

AlphaFold 3 predicted structures with ATP and Mg^{2+} ions (grey) and the template 2I1O (cyan).

Apyrase

Known ECs: 3.6.1.5 (acting on NTPs), 3.6.1.6 (acting on NDPs)

Apyrases are active against both di- and triphosphate nucleotides (NDPs and NTPs) and hydrolyze NTPs to nucleotide monophosphates (NMPs) in two distinct successive phosphate-releasing steps, with NDPs as intermediates. The eukaryotic enzymes require Ca^{2+}, but Mg^{2+} can substitute.[172-173] Unfortunately, there are no available structures in the presence of NTP, the coordination could not be established. The DALI alignment of the SF representatives revealed a surprisingly close resemblance to the *Alkaline phosphatase PhoX* family (InterPro entry IPR008557), the only protein family within the *Calcium-dependent phosphotriesterase* SF (b.68.6) capable of NTP processing. The *Alkaline phosphatase PhoX* family has a complex active-site with Ca^{2+} ions playing the pinching roles in BG(+) and BG(–) positions. The AlphaFold 3 prediction suggests a BG coordinated metal ion.

The purified porcine pancreatic ATP diphosphohydrolase (NTPDase) hydrolyzes both ATP and ADP, with the metal–nucleotide complex as the true substrate; Ca^{2+} is the preferred activating cation — increasing both Kapp and Vapp — whereas increasing free Mg^{2+} paradoxically raises K_m while reducing $V_{ma}{}^{x}$, indicating distinct kinetic modes for the two cations; Mg-ATP is nonetheless hydrolyzed, and divalent metal ion binding is essential for catalytic activity in all NTPDase family members.[174]

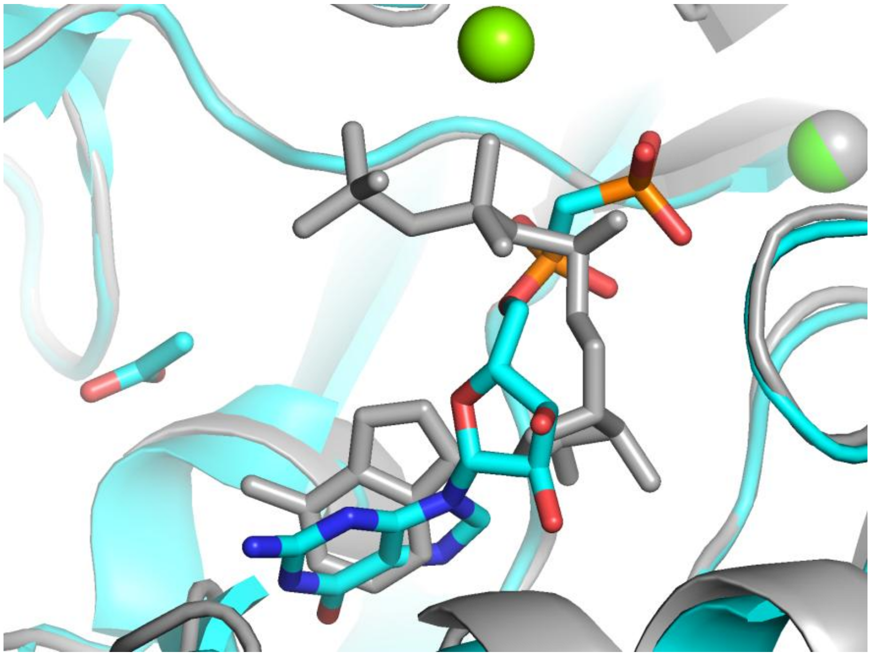
AlphaFold 3 predicted structure with ATP and Mg^{2+} (grey) and the template 1S1D (cyan).

Transglutaminase, two C-terminal domains

Known ECs: 2.3.2.13

The calcium-dependent multi-functional transglutaminase 2 possesses protein cross-linking and GTP hydrolysis activities.[175-176] Unfortunately, there are no structures available in the presence of metal ions, hence the coordination could not be established. The AlphaFold 3 prediction places the GTP to the same position as the crystal structure and needs multiple ions to position one close the GTP, in an ABG coordinated site.

Transglutaminase 2 (TG2) is activated by Ca^{2+} at a dedicated regulatory binding site, while Mg^{2+} acts as a competitive inhibitor by occupying the same site; as a result, the intracellular Mg^{2+}/Ca^{2+} ratio is a key determinant of TG2 activity and crosslinking function. [177]

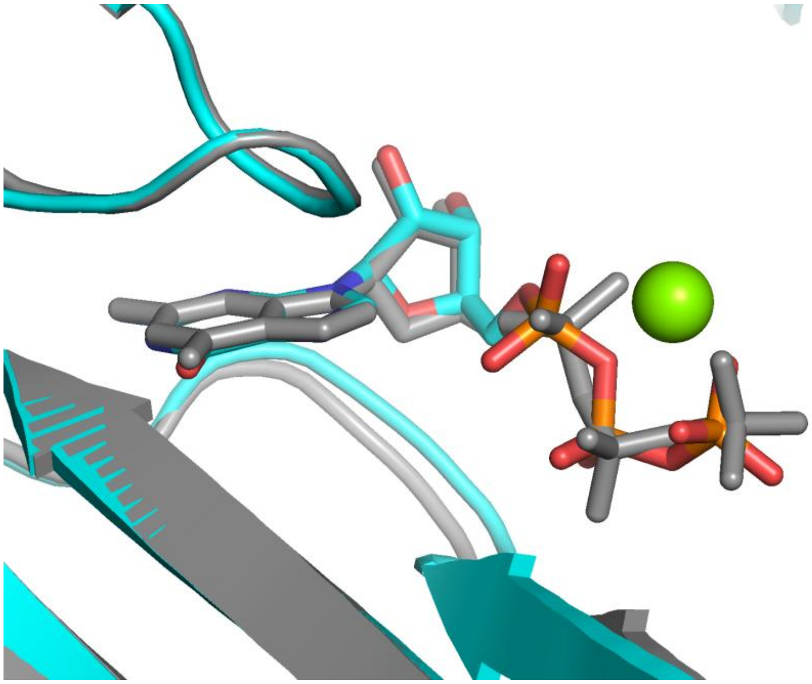

AlphaFold 3 predicted structure with GTP and Mg^{2+} ions (gray) and the template 6A8P (cyan).

S-adenosyl-L-methionine-dependent methyltransferases

This category consists of S-adenosyl-L-methionine-dependent methyltransferases (SAM MTase), which is not an NTP processing reaction. However, some viral proteins that belong to this superfamily were suggested to possibly possess guanylyl-transferase (GTase) activity,[178] where during the guanylyl-transfer process GMP is transferred to a 5′-end diphosphate viral RNA acceptor producing a pyrophosphate by-product [PDB 7fgg, 6z0u (Chikungunya Virus):[179-180]; PDB 4v03, 5dto, 8gzp (Dengue Virus):[181-183]; PDB 7v1h (Omsk hemorrhagic fever virus):[184]]. The exact coordination could not be established for this superfamily, as the coordination spheres of the bound Mg^{2+} only involve the phosphate groups. PDB 4v0r and 5dto suggest a βγ-coordination, whereas 7v1h suggests an αβ-coordination, which we think would be the favorable arrangement for its putative pyrophosphatase activity. AlphaFold cannot determine a single metal ion coordination, the predicted structures agree on the nucleotide binding only.

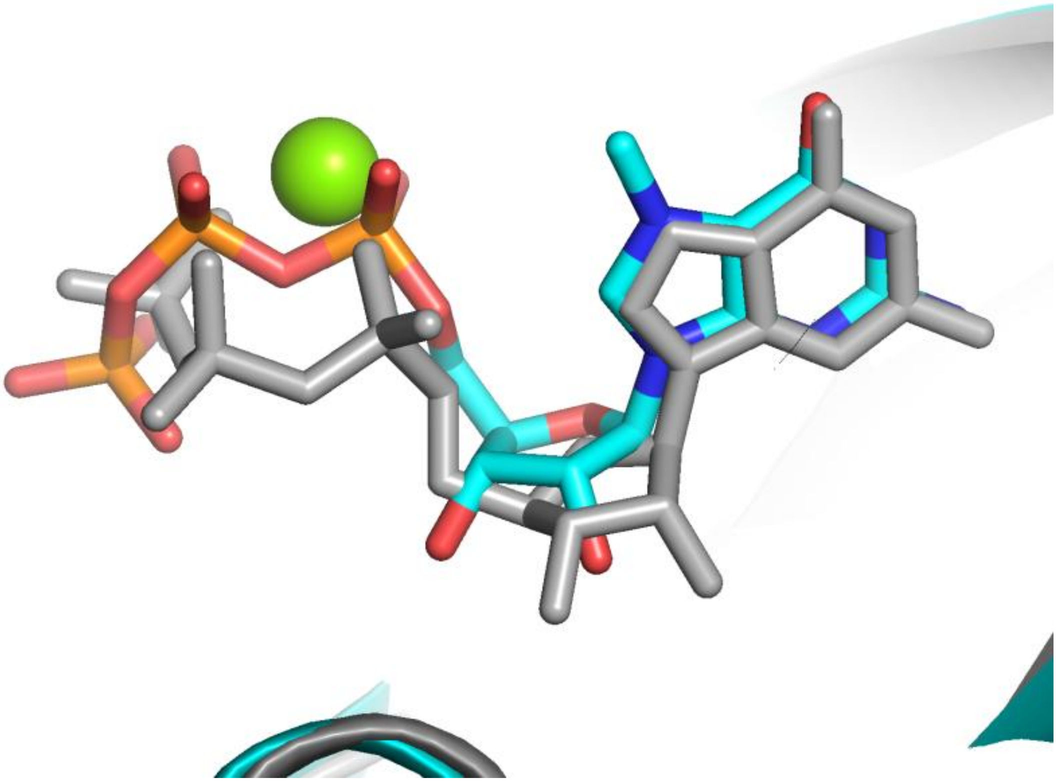

AlphaFold 3 predicted structures with GTP and Mg^{2+} ions (grey) and the template 7FGG (cyan).

RPB5-like RNA polymerase subunit

Known ECs: 2.7.7.6

This category can be found in prokaryotic subunit H and the C terminus of eukaryotic RPB5. Prokaryotes contain a single DNA-dependent RNA polymerase (RNAP) that is responsible for the transcription of all genes, while eukaryotes have three classes of RNAPs (I-III). They catalyze the DNA-template-directed extension of the 3'- end of an RNA strand by one nucleotide at a time, while releasing pyrophosphate as byproduct. There are no structures available with nucleotides and ions that could help us definitely conclude on the metal ion coordination. Yet since all other polymerases exhibit the same typical metal coordination, we predict that this superfamily will also share the similarity in its active site, with a Mg^{2+} coordinated by all three phosphate groups, and another Mg^{2+} that is coordinated by the 3' hydroxyl of the priming nucleotide and the α-phosphate of the incoming NTP. AlphaFold also predicts a typical two-metal catalytic polymerase site.

RNA polymerase II employs a catalytic two-Mg^{2+} centre coordinated by conserved aspartate residues of the RPB1 subunit; RPB5, which contributes to the jaw domain that clamps downstream DNA, is required for stable assembly of the active polymerase complex and for maintaining the geometry of this essential metal centre. [185]

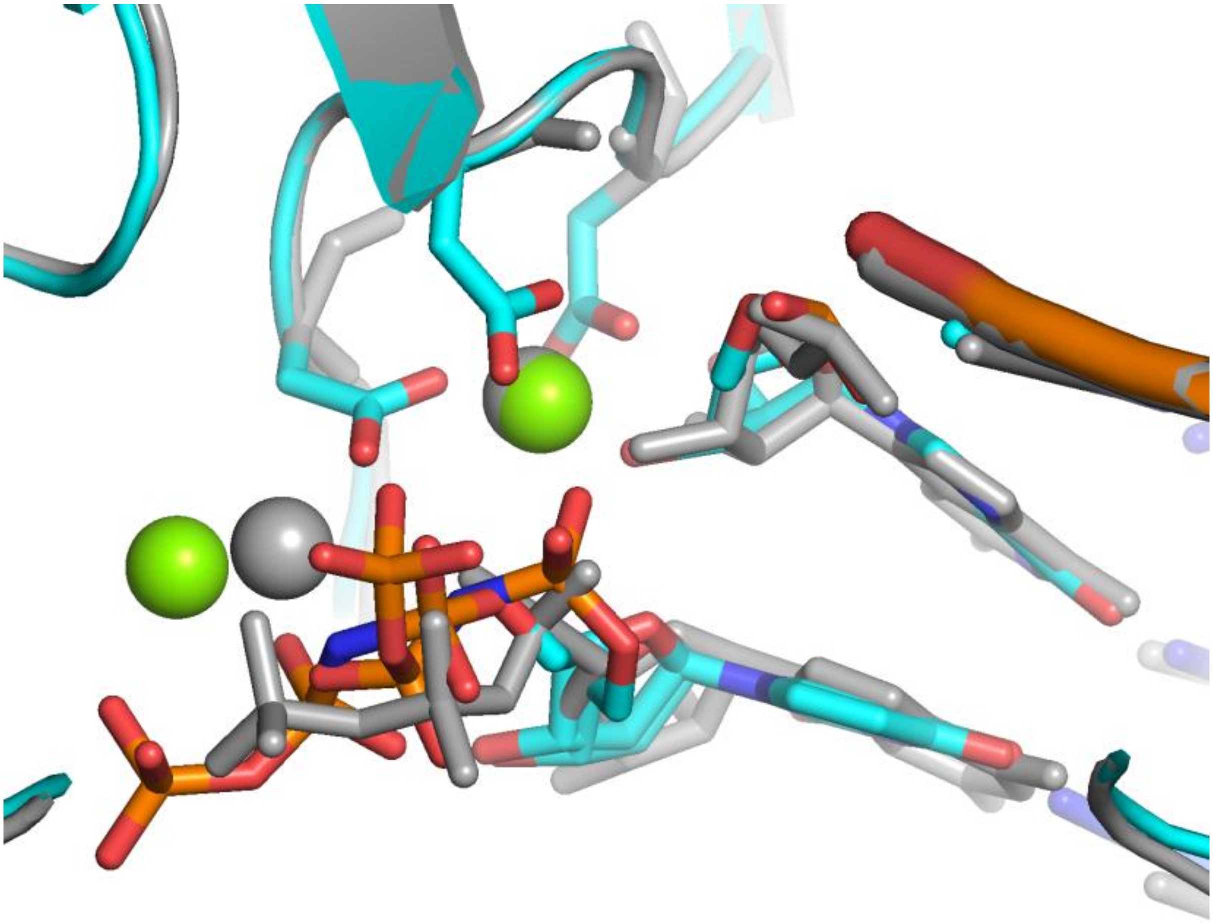

AlphaFold 3 predicted structure with GTP and Mg^{2+} ions (grey) and the template 5W51 (cyan).

Phosphatidate cytidylyltransferase, mitochondrial

Known ECs: 2.7.7.41

There is no corresponding SUPFAM superfamily for this phosphatidate cytidylyltransferase present in the mitochondrial inner membrane. We named the SF according to the matching the InterPro family IPR015222. Mitochondrial phosphatidate cytidylyltransferases catalyze the synthesis of CDP-diacylglycerol from CTP and phosphatidate. The AlphaFold predictions using the template (PDB 7ECD) position the CTP in agreement with the template crystal structure.

Tam41, a mitochondrial CDP-diacylglycerol synthase essential for cardiolipin biosynthesis, strictly requires Mg^{2+} for activity and is inhibited by EDTA; although Co^{2+} supports approximately 80% and Cu^{2+} approximately 30% of wild-type activity, Mg^{2+} is clearly the preferred and physiologically relevant divalent cofactor. [186]

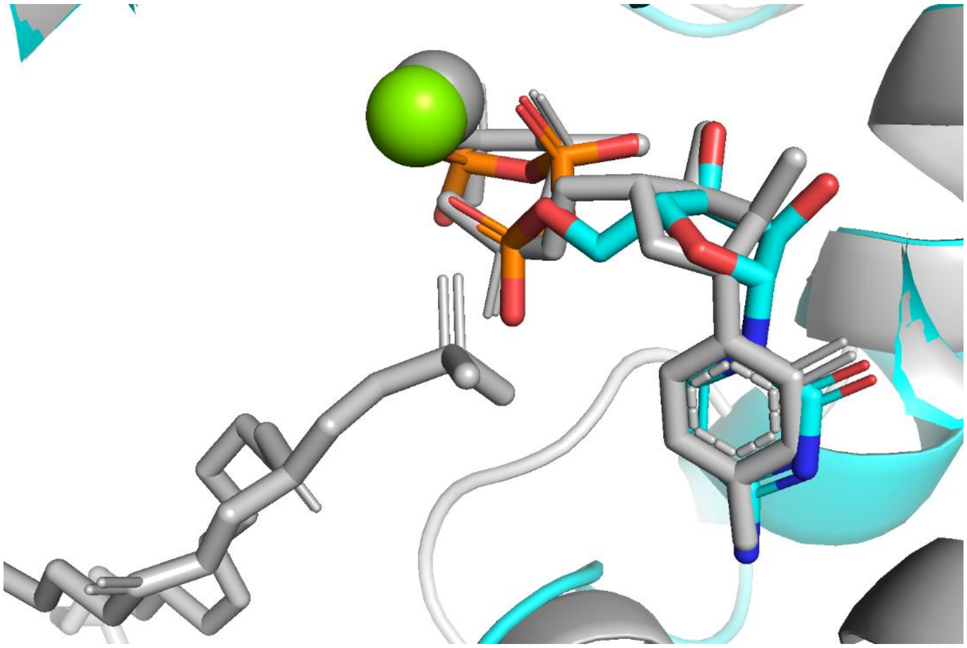

AlphaFold 3 predicted structures with CTP and Mg^{2+} ions (grey) and the template 7ECD (cyan). The phosphatidate molecule shown in grey is predicted to be close to the other substrate CTP.

Molybdenum cofactor biosynthesis protein C, MoaC

Known ECs: 2.7.7.77, 4.6.1.17

This superfamily corresponds to the molybdenum cofactor biosynthesis protein MoaC from prokaryotes and eukaryotes, also known as cyclic pyranopterin monophosphate synthases. Molybdenum cofactor guanylyltransferases (EC 2.7.7.77) catalyze the guanylation of the molybdenum cofactor, which occurs only in prokaryotes. In bacteria the cyclic pyranopterin monophosphate synthase reaction (EC 4.6.1.17) is catalyzed by MoaC.[187] There are no available structures with metal ions to comment on the metal ion coordination of this superfamily, the AlphaFold 3 prediction does not agree with the NTP binding mode.

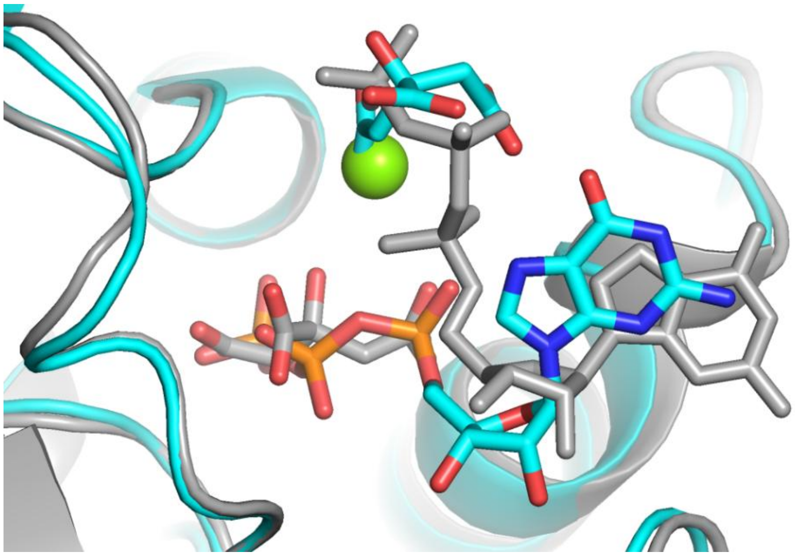

AlphaFold 3 predicted structure with GTP and Mg^{2+} (grey) and the template 3JQM (cyan).

GTP cyclohydrolase MptA

Known ECs: 3.5.4.39

There are no available structures for this category which is defined as an InterPro family (IPR022840). MptA requires Fe^{2+} for activity.[188] Unfortunately, due to the lack of available structures, we are unable to comment on the metal ion coordination, apart from AlphaFold predictions, which suggest an ABG ion position.

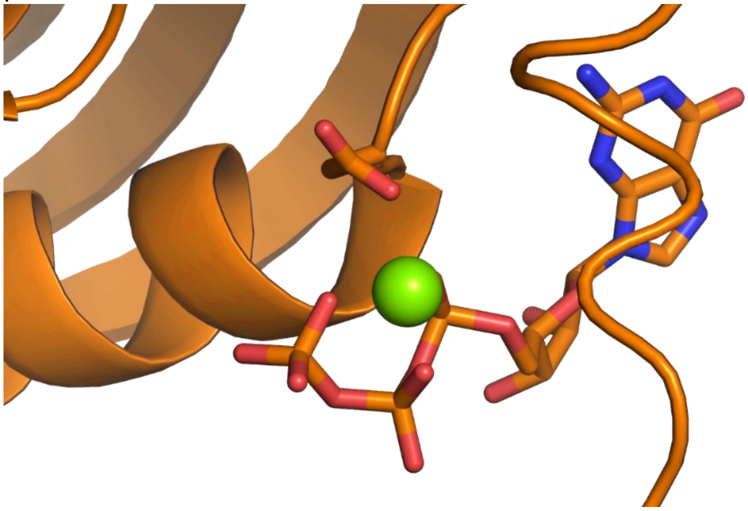

AlphaFold 3 predicted structure with ATP and Mg^{2+}.

13. Triphosphatase superfamilies

S-adenosylmethionine synthetase

Known ECs: 2.5.1.6

S-adenosylmethionine synthetase is the enzyme that catalyzes the formation of S-adenosylmethionine from methionine and ATP, the sequence of S-adenosylmethionine synthetases is highly conserved throughout isozymes and species.[189] The reaction is suggested to initiate by the cleavage of C5'-O5' bond of ATP, caused by the action of a histidine residue; simultaneously C5' of ATP is the target of a nucleophilic attack by Sδ of Met, leading to the bond formation between Met and C5' of ATP to produce SAM and the release of triphosphate. The triphosphate is then hydrolyzed, producing pyrophosphate and P_i.[190] There are two Mg^{2+} ions in the active site, one is coordinated by all three phosphate groups on the (-) side, which may facilitate the second step of the reaction when the PPP_i is hydrolyzed, while another is coordinated by P_α and P_γ on the opposite side.

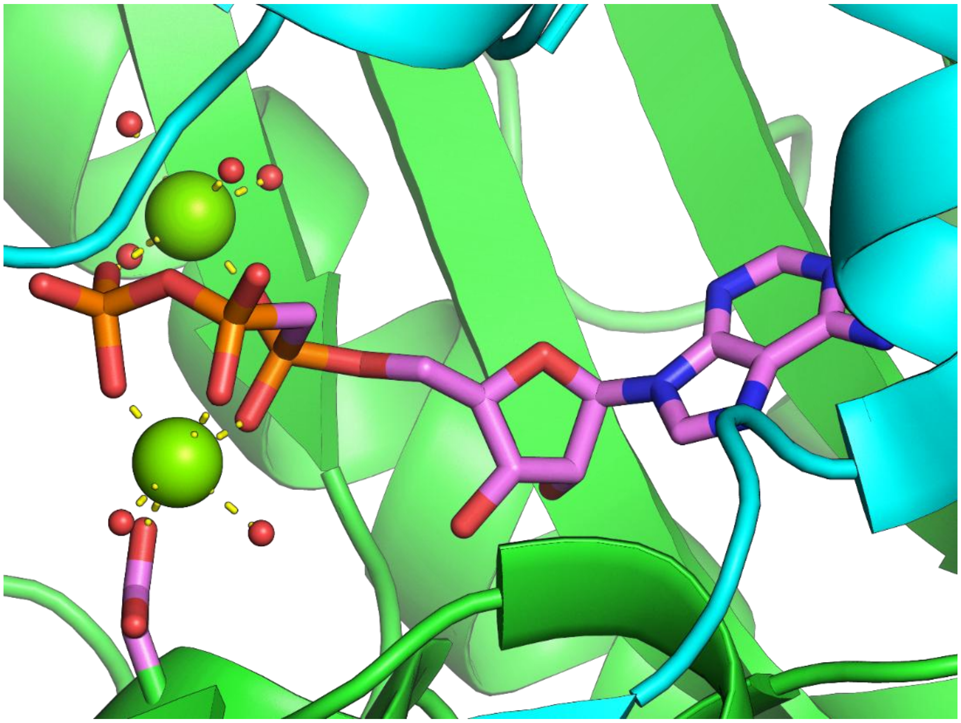

ATP and Mg^{2+} binding in the representative structure, 6vd0.

Cobalamin adenosyltransferase-like

Known ECs: 2.5.1.154, 2.5.1.17

Enzymes belonging to this superfamily catalyze the conversion of cobalamin (vitamin B12) into its coenzyme form, adenosylcobalamin (AdoCbl) or coenzyme B12.[191] Depending on the EC, the triphosphate may be hydrolyzed into P_i and PP_i during catalysis (EC 2.5.1.154). We identified two metal ions at the active site, one of which is coordinated by all three phosphate groups on the (+) side, while the other is on the opposite side coordinated by the P_{α}.

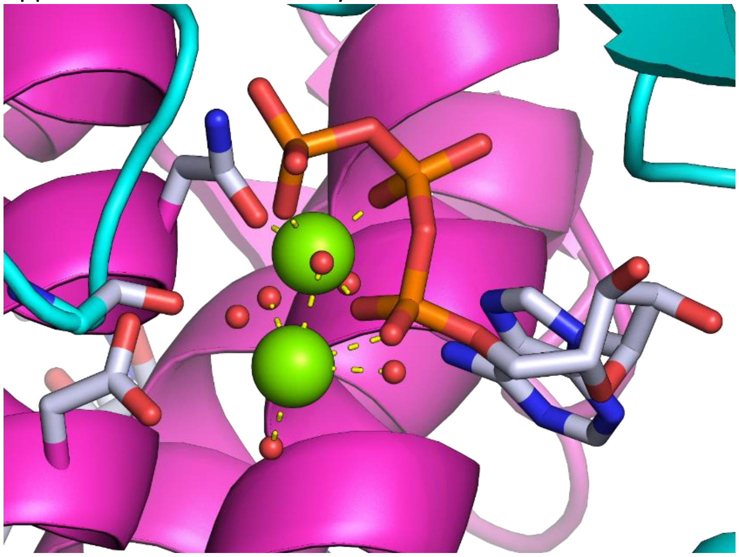

Substrate and metal ion binding in the representative structure, 6D5K.

Tetrahydrobiopterin biosynthesis enzymes-like

Known ECs: 4.2.3.12, 4.1.2.50

This category is not strictly NTP processing, as it processes 7,8-Dihydroneopterin 3'-triphosphate. During the reaction PPP_i is released, while 6-Pyruvoyltetrahydropterin is produced. 6-Pyruvoyl tetrahydrobiopterin synthase (PTPS) catalyzes the conversion of dihydroneopterin triphosphate to 6-pyruvoyl tetrahydropterin, the second of three enzymatic steps in the synthesis of tetrahydrobiopterin from GTP.[192] A bound Zn(II) responsible for the enzymatic activity, its binding site is believed to be formed by three histidine residues.[193] Some members of this superfamily are GTP cyclohydrolase I (EC 3.5.4.16), which does not process the triphosphate chain of the GTP, hence is irrelevant for our analysis. All available structures in the presence of NTP and metal ion correspond to GTP cyclohydrolase I (they have a Zn ion coordinated by the P_α), however, we did not identify such structures for EC 4.2.3.12 or 4.1.2.50 hence the coordination could not be established.

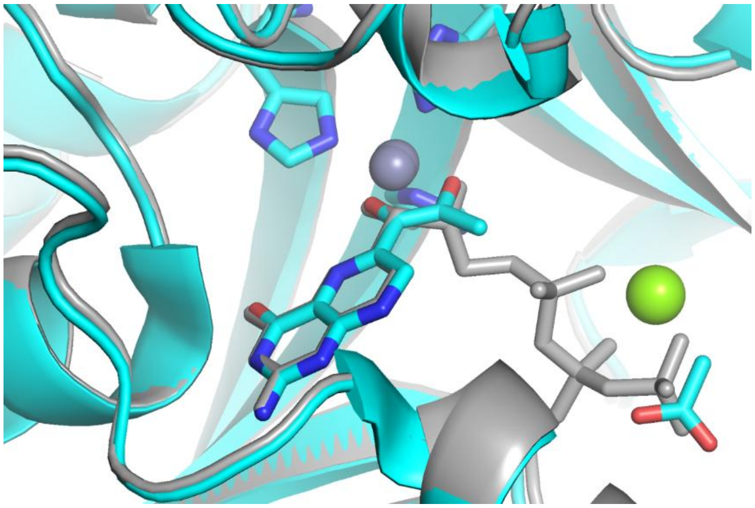

AlphaFold 3 predicted structure with 7,8-dihydroneopterin 3'-triphosphate, Zn^{2+} and Mg^{2+} (grey) and the template 4NTK (cyan). The AlphaFold prediction suggests an ion in the ABG(+) position.

HD-domain/PDEase-like

Known ECs: 3.1.5.1

Members of this superfamily having an HD-domain catalyze the hydrolysis of dNTPs into their constituent triphosphate and 2'-deoxynucleoside.[194] Based on the selected representative structure PDB 6txe, there are three octahedrally coordinated metal ions (2 Mg^{2+} and an Fe) that support catalysis. A Mg^{2+} is coordinated by P_β and P_γ, and both the second Mg^{2+} and the Fe are α-coordinated. A crystal water bridges the two metal ions with an approximately tetrahedral Fe–O–Mg bond angle, and possibly this water is the source of the nucleophile that attacks the P_α during the dNTP hydrolysis.[194]

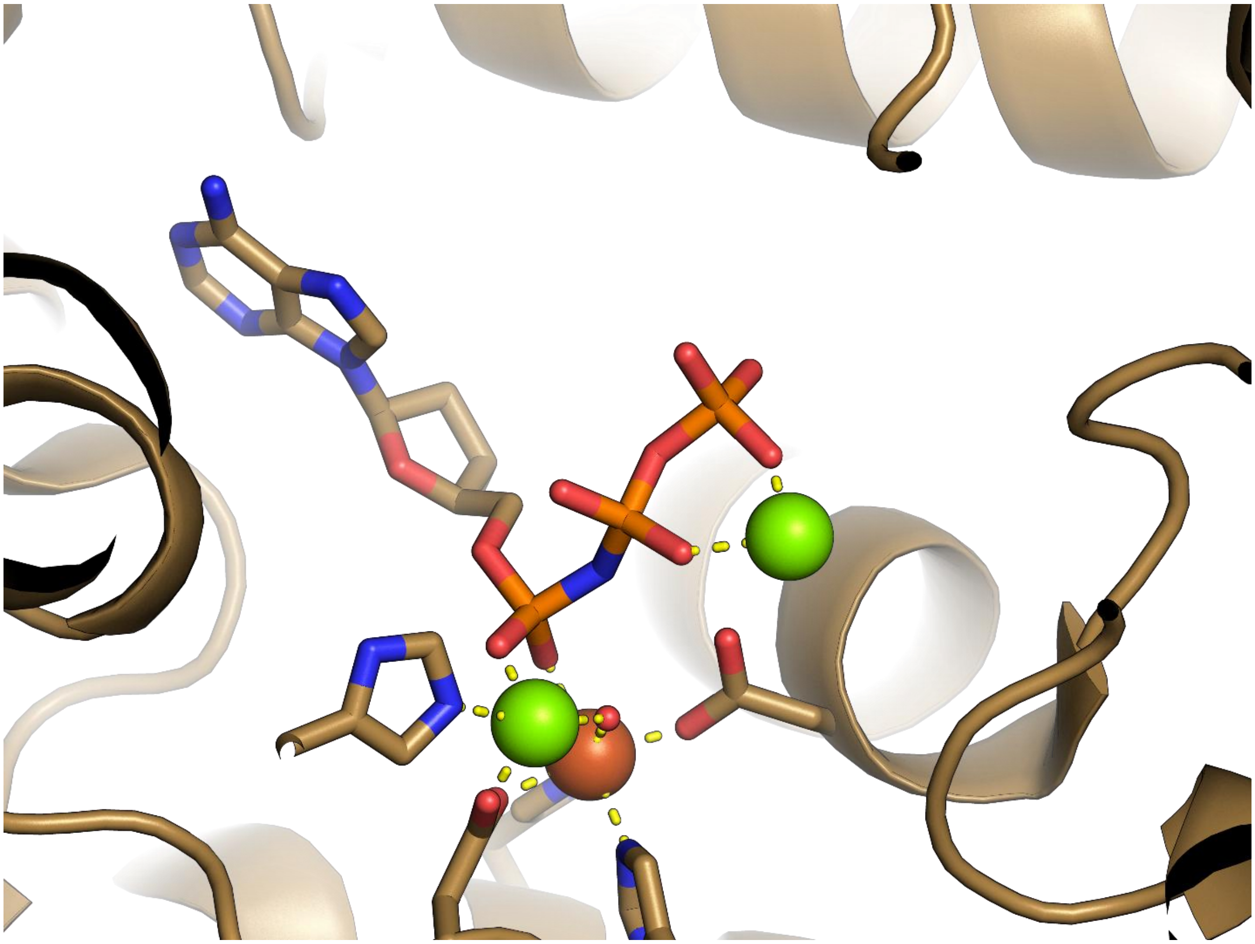

Substrate and metal ion binding in the representative structure, 6tx0.

Known ECs: 3.1.3.112

As a special member of the HD-domain superfamily, OxsA has been shown to catalyze the sequential hydrolysis of tri-, di-, and mono-phosphorylated oxetanocin A compounds, as well as dATP, dADP, and dAMP, releasing one molecule of inorganic phosphate at each step. Based on the selected structure PDB 5TK7, there are two octahedrally coordinated Mg ions. One of the Mg ions is used for the sequential hydrolysis of β/γ-phosphate groups, while the other one coordinated by its HD-motif is used for elimination of α-phosphate group. The active site of OxsA switches from a dinuclear to a mononuclear metal center when β/γ-phosphates are eliminated from the substrate. In a resolved structure of OxsA (PDB Code 5TK8), where the substrate is monophosphorylated oxetanocin A, only one metal ion rather than two metal ions is used to catalyze the hydrolysis of mono-phosphorylated oxetanocin A.[195]

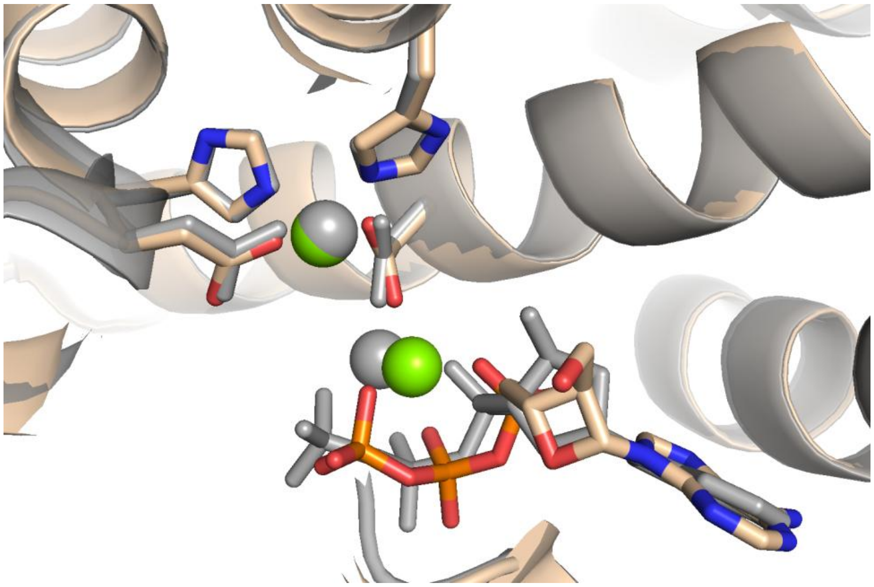

A predicted structure (grey) of OxsA with dATP, two Mg^{2+} ions, and the template 5TK7 (light orange). The prediction suggests an Mg^{2+} ion in the ABG(-) position, consistent with the crystal structure.

Appendix

QM Geometries for the model enzymatic transition states and reactants

```
101
Ras reactant -4302.99154436721
 C           1.7578509721        2.5294206637        -9.2469958698
 H           0.9719698150        1.7733564156        -9.1570316630
 H           2.6840642028        2.1272928158        -8.8279258322
 H           1.4483173182        3.3660583144        -8.6613259268
 C           1.8905510735        2.8910932955       -10.7194829691
 O           0.9117360989        3.3241444904       -11.3662328537
 N           3.0829982700        2.6651319323       -11.2940435038
 H           3.8299543541        2.2788749083       -10.7245426488
 C           3.3267254952        2.6598525237       -12.7386596241
 H           2.4159575904        2.3504024230       -13.2575563194
 H           4.1213950149        1.9353602851       -12.9242214778
 H           3.6356023470        3.6398026572       -13.1032634325
 C           7.0915871058        4.6236746700        -7.2488141723
 H           6.7589883779        4.4139077021        -6.2306843559
 H           7.9273877373        3.9740995992        -7.4985069508
 N           5.9673573924        4.3096574206        -8.1766251691
 H           6.1993647859        4.5174176173        -9.1665976960
 H           5.0976894267        4.7970425547        -7.9057772538
 H           5.7679603800        3.2725488348        -8.1819941034
 H           7.3940093152        5.6839616408        -7.3352555764
 C          11.7348915158        1.8500160836       -11.6469469169
 H          12.5935509404        1.2503011288       -11.9704318277
 H          10.9200112214        1.7874435834       -12.3693953693
 O          11.2400490554        1.3412390256       -10.4040134722
 H          11.9024906694        1.5690059441        -9.6865750054
 H          12.0473659544        2.9146019238       -11.5297016895
 C           8.5289918055       -4.3044823105        -9.6769553216
 O           9.3627472505       -5.2329421690        -9.6807318942
 H           7.7734735271       -4.2843472724       -10.4700939620
 N           8.4546669194       -3.2996822753        -8.8018289414
 H           7.5934639281       -2.7340973317        -8.8086648084
 C           9.4252765773       -3.0231455246        -7.7625893940
```

H	10.3674373237	-3.4983010101	-8.0489633183
C	9.6443373200	-1.4787550593	-7.6838673196
H	8.6758015903	-1.0119603593	-7.4830487826
O	10.0047815634	-1.0058229872	-8.9913266699
H	10.8056565973	-1.4924381877	-9.3211133964
C	10.6828096873	-1.0178408127	-6.6643228085
H	10.8690766833	0.0492113902	-6.8103685429
H	11.6350851568	-1.5489926925	-6.7879005560
H	10.3326014184	-1.1609674574	-5.6377645429
C	9.0083849311	-3.5756369800	-6.3824148713
O	7.8413381343	-3.5764361693	-5.9816715490
N	10.0636987327	-4.0004912148	-5.6460174388
H	11.0140367594	-4.0328137934	-6.0380617392
H	9.9910495276	-4.2033641317	-4.6402187716
C	5.7553471683	1.5059577616	-4.6706786629
O	5.6225675805	2.1207728878	-3.5987094973
H	6.5924703882	0.8167760652	-4.8086055629
N	4.9108371106	1.5917519297	-5.7120144059
H	5.2023457635	1.2674191781	-6.6495857249
H	4.1102694203	2.2458682084	-5.7167710700
C	1.5531547587	-2.0178964014	-6.1030096254
H	0.6486648405	-1.5010169200	-6.4335076765
H	1.3985653022	-3.0959592455	-6.2483316649
H	1.7174531115	-1.8401239672	-5.0167373250
C	2.7759162404	-1.6131702602	-6.9062867402
O	3.9241210606	-1.8054056523	-6.4660792961
N	2.5729568865	-1.0431669464	-8.1079004809
H	3.3757928945	-0.7644457946	-8.6814186437
H	1.6270799998	-0.8704186450	-8.4582627692
C	3.0255916564	-2.1448797128	-11.4493271606
H	2.3788965190	-2.9498142630	-11.0979429433
H	3.8021956594	-1.9857118418	-10.7035623033
N	3.6152259487	-2.5754111064	-12.7261734116
H	3.1288778996	-3.3403642250	-13.2149980039
C	4.7816951816	-2.1212301714	-13.2050078889
N	5.5139910593	-1.2404771221	-12.5217505530
H	6.2842093929	-0.7467658592	-12.9716819500
H	5.2722609083	-0.9272147051	-11.5590658314
N	5.2210868858	-2.5851397535	-14.3962273382
H	4.5642854777	-3.0024120078	-15.0619994865
H	6.1316968420	-2.2718948052	-14.7586302588
H	2.4428442064	-1.2082770423	-11.5917992180
MG	9.2499028273	0.7731393798	-9.8694426191
C	6.0608778718	1.6576762156	-15.7603853254
H	6.1540159451	0.5702365438	-15.6659177622
H	5.2535366668	1.9949034732	-15.0989702202
O	7.2865351461	2.2979624695	-15.3943465760
P	8.0185135423	1.9281029524	-13.9845710965
O	9.3915165280	2.5397020000	-14.0532590135
O	7.8507626880	0.4347076677	-13.6863287652
O	7.0211673728	2.7494314752	-12.9711045429
P	7.1420386060	2.7906401781	-11.3194445752
O	6.7135222102	4.1895594187	-10.9087853549
O	8.5345908067	2.3181336320	-10.9661429373
O	6.0161594999	1.7303174648	-10.8911648469
P	5.9776444357	0.6976951674	-9.4961400157
O	5.5376277264	1.6325431019	-8.3575973163
O	7.4043681036	0.1598536401	-9.3602723215
O	4.9122462023	-0.2992191377	-9.9362618024
H	5.8164354536	1.9138957019	-16.8070702628
O	9.5323076189	1.9603698067	-8.1868179962
H	9.0783114503	2.0230330006	-7.3200190530
H	10.3541912049	2.5371135566	-8.0907647129

O	9.3406630708	-0.4202495616	-11.6443224373
H	8.7638987514	-0.1158614791	-12.3972420991
H	9.2209950440	-1.3907075196	-11.5767254520
O	5.9273480417	-2.8210233217	-7.9733045141
H	5.2085031252	-2.2706726769	-7.5890518153
H	6.3636734892	-3.2076132387	-7.1914683991

101

Ras TS -4302.95313924386

C	1.8489409884	2.5052063365	-9.2895148761
H	1.1009613523	1.7159584033	-9.1694357742
H	2.8140539773	2.1509405722	-8.9149420782
H	1.5215370873	3.3267823780	-8.6929569684
C	1.8915268861	2.8740656523	-10.7678210663
O	0.8449877648	3.2641709265	-11.3389578550
N	3.0466761438	2.6922100373	-11.4137908148
H	3.8939337858	2.3364901178	-10.9469669521
C	3.1911312045	2.7215460246	-12.8696249706
H	2.2377359932	2.4787761422	-13.3418707508
H	3.9422986852	1.9713580217	-13.1209635185
H	3.5303816867	3.6953494537	-13.2134443874
C	6.9672894580	4.3049580622	-7.3162107702
H	6.5842133974	4.0718871054	-6.3213166996
H	7.8603777095	3.7154174716	-7.5085723145
N	5.9395672657	3.9012743409	-8.3205217628
H	6.2287396622	4.0864428585	-9.3205628040
H	5.0273506898	4.3617195733	-8.1670343874
H	5.8140609212	2.8656206266	-8.2683199441
H	7.2045513860	5.3837732275	-7.3820116942
C	11.6534661384	1.8095696870	-11.6481150467
H	12.5174599182	1.1962920620	-11.9336883353
H	10.8717883370	1.7618556908	-12.4083456564
O	11.0872900243	1.3174347866	-10.4320485709
H	11.7409934062	1.4904850465	-9.6937542396
H	11.9810465215	2.8669874601	-11.5157705521
C	8.5626881996	-4.2248335732	-9.7081431155
O	9.2197904776	-5.2780663717	-9.5803097497
H	7.8193562683	-4.1702742712	-10.5133058240
N	8.6488250784	-3.1406760799	-8.9320066739
H	8.0974535455	-2.3023130384	-9.1825309002
C	9.6424693902	-2.9715048108	-7.8817555262
H	10.5377065634	-3.5401565921	-8.1489658196
C	10.0081802939	-1.4562106659	-7.7785053431
H	9.1206171609	-0.9177282712	-7.4451435997
O	10.2430268301	-0.9645699768	-9.0969056654
H	10.9925822711	-1.4527616660	-9.5211387170
C	11.1771472100	-1.1163852658	-6.8612391597
H	11.4821747822	-0.0859535943	-7.0609610795
H	12.0388694181	-1.7738914927	-7.0229717633
H	10.8803330837	-1.1695210321	-5.8107924021
C	9.1249035634	-3.4625228858	-6.5222426051
O	7.9471254094	-3.3150720931	-6.1780114257
N	10.0895293302	-3.9980966929	-5.7384421284
H	11.0556377387	-4.0925986510	-6.0820831589
H	9.9606938365	-4.1591866984	-4.7303602550
C	5.6656967667	1.6408658593	-4.4260923043
O	5.4384930111	2.2783449551	-3.3835747312
H	6.5264734942	0.9737768189	-4.4929243037
N	4.8848253842	1.6380231157	-5.5217543692
H	5.2851641167	1.2827607374	-6.4254960509
H	4.0471509160	2.2367968260	-5.6106254569
C	1.6215854087	-2.3110680567	-5.9829320009
H	0.6509380596	-1.8847663288	-6.2371373326

H	1.5576658972	-3.4055213991	-6.0409411024
H	1.8905292521	-2.0457765596	-4.9348402206
C	2.6808091466	-1.8598611367	-6.9336614368
O	3.8794312751	-2.3245282064	-6.7251191379
N	2.4623679533	-1.0389217833	-7.9273091743
H	3.2967413399	-0.7991309760	-8.5204590122
H	1.5108800249	-0.7496851078	-8.1827980299
C	3.1400848299	-1.8064213222	-11.3953019585
H	2.5948943061	-2.6330475527	-10.9330084788
H	3.9559842474	-1.5314974009	-10.7218392429
N	3.6666668813	-2.2783405567	-12.6816477548
H	3.1750234604	-3.0782641859	-13.1034487642
C	4.8350682987	-1.8804480782	-13.2100878412
N	5.5834746162	-0.9333367673	-12.6490985985
H	6.3816062459	-0.5173823339	-13.1435505637
H	5.3193842746	-0.3921006828	-11.8231793179
N	5.2697270781	-2.4931774769	-14.3378637266
H	4.6002468309	-2.9507420129	-14.9615276719
H	6.1616642635	-2.1957259299	-14.7564970013
H	2.4628088714	-0.9405603884	-11.5497557887
MG	9.0505881135	0.6250725953	-9.8999971315
C	6.1024501539	1.6627341733	-15.8244760739
H	6.1412149561	0.5712638869	-15.7215843229
H	5.3314671107	2.0459923810	-15.1435848161
O	7.3684950223	2.2380674776	-15.5047620840
P	8.0390395379	1.9131504800	-14.0422451709
O	9.4248183020	2.5079846575	-14.0895835076
O	7.8990099518	0.4004401664	-13.7713386397
O	7.0125900923	2.7179269040	-13.1089093860
P	6.8097751999	2.5665043590	-11.4115446074
O	6.5509862250	4.0298700910	-10.9868404977
O	8.1707314371	2.0290003762	-10.9502977448
O	5.6178534199	1.6309936102	-11.2269045267
P	6.1664504987	-0.3390041871	-8.4017968444
O	5.9087589859	1.1028836434	-7.9380925426
O	7.4474101829	-0.5476188025	-9.2452696344
O	4.9046142280	-1.0603585939	-8.9581135969
H	5.8412452436	1.9183124962	-16.8664935665
O	9.3722306076	1.6988133543	-8.1122322059
H	8.8927988002	1.8137362801	-7.2680066702
H	10.1889683441	2.2844455049	-8.0204958235
O	9.2940853071	-0.4930631075	-11.7041907287
H	8.7368306229	-0.1486855694	-12.4598382043
H	9.2422680594	-1.4715081089	-11.7340074990
O	6.3920844999	-1.1606344665	-6.9792008363
H	4.5381606664	-1.9578147020	-7.3995795224
H	6.9508799374	-1.9667623278	-6.9586414557

132
dUTPase reactant

C	-8.4229901646	5.5374618856	-2.9757534319
O	-9.3564467626	5.7826540565	-2.2100155439
H	-7.7450681635	4.6938785604	-2.7752565121
N	-8.1528113528	6.2200547199	-4.1088171669
H	-7.2626289944	6.0184153086	-4.5780901869
C	-8.9777608591	7.3031645622	-4.6376067727
H	-9.7964883884	7.4594318176	-3.9351050444
H	-8.4301494014	8.2511340657	-4.7794892330
H	-9.3929767465	6.9531012578	-5.5878106914
C	-12.9640383602	5.7945066378	-6.8311555778
H	-13.5577715788	6.5956487646	-6.3826533359
H	-13.4819311166	5.4082209605	-7.7143993757
C	-12.7351943882	4.6464531982	-5.8313852867

O	-12.2526427486	3.5748747255	-6.2837954044
O	-12.9971691265	4.8611285958	-4.6113037567
H	-11.9661290905	6.1937444957	-7.1188521611
CA	-15.2779581511	1.4662272397	-1.0864322733
C	-13.8340975124	5.7237743954	-0.8236380985
H	-14.5285680645	5.7168547900	0.0228711565
H	-14.3146605561	5.2303112415	-1.6729791995
O	-12.6326484916	5.0072397883	-0.4609157151
P	-12.7269974504	3.4480243502	-0.0669676411
O	-13.6050838947	2.6847570911	-1.0442452578
O	-11.3576276688	2.9362725229	0.2618502129
O	-13.5421464812	3.5724195521	1.3792856146
P	-14.9884634998	2.9661813016	1.8164109992
O	-15.2253942518	1.6541875423	1.0790095407
O	-15.0035259901	2.9820106380	3.3236050139
O	-16.0121953679	4.0904504875	1.2556532776
P	-17.3218085462	3.6851722592	0.2486005748
O	-18.1985069879	2.7887516503	1.1557340979
O	-16.7166575682	2.9557835318	-0.9658488697
O	-17.9025854519	5.0569912212	-0.1076719942
C	-13.4865870718	7.1418107110	-1.2124010698
H	-14.4176296291	7.6264285527	-1.5417754176
O	-12.9644195927	7.8580470197	-0.0672791441
C	-11.9921619678	8.8111472161	-0.4757547086
H	-12.2967120070	9.8118717177	-0.1712283672
N	-10.7199499541	8.5549431918	0.2474346527
C	-10.1833291382	7.2902818513	0.2054116643
H	-10.8064105207	6.5290013714	-0.2424931617
C	-10.0379326111	9.6117112132	0.8414363556
O	-10.4654043457	10.7690046826	0.8813426532
N	-8.8091966641	9.2708675684	1.3801970388
H	-8.3822800389	9.9885437129	1.9861420802
C	-8.1879462802	8.0270105232	1.3286000101
O	-7.0511518970	7.8725364720	1.8213556852
C	-8.9548982113	7.0004637494	0.6929291623
H	-8.5696857318	5.9930074751	0.6277128377
C	-11.8450219210	8.6633929718	-1.9982796937
H	-10.8133530916	8.7733369328	-2.3315068213
H	-12.4486275520	9.4218188988	-2.4885857034
C	-12.4579274026	7.3035263115	-2.3436110728
H	-11.7084161663	6.5061110828	-2.3085978679
O	-13.0761296288	7.3356822703	-3.6137797362
H	-13.0590815705	6.4173705455	-4.0024804852
O	-9.4633032054	8.1548099739	4.8010065532
H	-10.0427890317	8.8951064630	4.5111031517
H	-8.5251279860	8.3964830736	4.6335356366
O	-15.3839015009	1.1598524497	5.2472007604
H	-15.3831211354	0.2865440999	4.8280913790
H	-15.3125182805	1.8007844250	4.4982105449
O	-9.7591584216	6.4139842391	6.8887658938
H	-10.5615622188	5.8841998059	6.7024840409
H	-9.6673276200	7.0503902616	6.1367390827
O	-11.6029633697	2.7755400865	-3.2142353023
H	-12.0016447309	3.5211873287	-3.7129426181
H	-12.2453480703	2.6035904233	-2.5050032182
O	-15.7635985065	-1.0239626885	-4.3451397004
H	-14.9974244417	-1.2579398934	-4.9192345439
H	-15.6425125791	-1.5715331002	-3.5229272009
O	-15.8727477462	12.0039019635	1.2175101289
H	-15.0723235338	12.1227962151	0.6587673603
H	-16.6050948200	11.9397081155	0.5674848082
O	-12.7319488587	1.0974532508	-5.0514207237
H	-12.1405046755	1.2011405680	-4.2752859749

H	-12.6103691477	1.9729195198	-5.4915791865
O	-16.9839045355	0.1682044413	-1.0656414907
H	-17.8684799655	0.5202388707	-0.8519699848
H	-17.0664081376	-0.8198814116	-1.1390896304
O	-15.1428814377	1.4029683158	-3.2033564964
H	-14.3118032014	1.5950301095	-3.6923847554
H	-15.4623414608	0.5803124642	-3.6526614726
O	-14.1765854352	-0.3336194458	-1.0279268706
H	-13.3491206711	-0.2357152169	-1.5611030318
H	-14.6488040650	-1.1064118852	-1.4446870226
C	-11.3879390162	5.5633793799	3.5241122485
H	-11.1699559416	6.4519988042	4.1165445550
H	-10.5934876960	5.4593051136	2.7756467598
O	-12.6576244416	5.7557033838	2.9151362721
H	-12.8475774974	5.0082566351	2.3225660439
H	-11.3638546036	4.6544318043	4.1822951579
C	-10.8895972979	-0.4592815451	-1.5256581889
O	-11.7898103242	-0.2274086591	-2.3508393082
H	-10.4900365785	-1.4884087396	-1.4265675427
N	-10.3695280506	0.4501231775	-0.6951602857
H	-10.7592558137	1.3968371807	-0.6297622816
H	-9.7401297209	0.1809188794	0.0590063314
C	-16.2772048138	7.2099650814	-5.1494341114
H	-15.7389050115	7.3244692634	-6.0930110510
H	-15.6195870386	7.5465384914	-4.3410285463
N	-16.6100988108	5.7917060360	-4.9938955719
H	-16.3713909954	5.1492160504	-5.7799739631
C	-16.8360179283	5.2120100548	-3.8028598735
N	-16.8839520974	5.9272766832	-2.6662037313
H	-17.2289090386	5.4926051554	-1.8035693956
H	-16.9494029614	6.9473100034	-2.6679668187
N	-17.0366082491	3.8909747802	-3.7314890677
H	-16.9196294331	3.4384351619	-2.8229747818
H	-16.9051938101	3.3072196718	-4.5882034590
H	-17.2021254023	7.8223371694	-5.1772180384
N	-13.9540416841	9.9995240849	3.6017773335
H	-14.7600185059	10.4265418796	3.1566716851
C	-13.9765435402	9.0301427679	4.5865680631
H	-14.8680171323	8.4917993306	4.8925316123
C	-12.6504225793	10.3360679226	3.3917988454
H	-12.3390932108	11.0663359131	2.6573698963
N	-11.8434323062	9.6385356064	4.1733559783
C	-12.6649129612	8.8263825599	4.9253602281
H	-12.2632654759	8.1449098859	5.6618768223
C	-20.6193529228	6.8255034969	0.8805561802
H	-21.5838255681	7.3159978584	0.7195049826
H	-19.8913815642	7.5802258712	1.2043023142
O	-20.2398498457	6.2280284595	-0.3584180301
H	-19.3595960585	5.7534948169	-0.2564528891
H	-20.7319893136	6.0764535451	1.7123650673
C	-20.7794449229	1.6226160447	-0.5405438470
H	-21.3853333864	0.8559118578	-1.0307967979
H	-20.4610587050	2.3457551457	-1.3030808571
O	-19.6574941471	0.9732734429	0.0516409048
H	-19.0760558936	1.6824200092	0.4932026010
H	-21.4083165980	2.1702672440	0.2108938131

132
dUTPase TS

C	-8.4453611575	5.5347128637	-2.9688009264
O	-9.4192058925	5.7474178828	-2.2462192317
H	-7.7761291771	4.6852111144	-2.7640515992
N	-8.1256413871	6.2526972100	-4.0671618129

H	-7.2243435358	6.0583053332	-4.5180005584
C	-8.9427868644	7.3357472071	-4.6092312317
H	-9.7655083062	7.5033199583	-3.9137908314
H	-8.3907595314	8.2793708182	-4.7541987506
H	-9.3531840378	6.9793370027	-5.5586317946
C	-12.8663728165	5.6390050423	-6.6755603076
H	-13.4900615683	6.4033733067	-6.2061321159
H	-13.4029558161	5.2199841539	-7.5293443757
C	-12.5216914028	4.5297207088	-5.6687702042
O	-12.0065710081	3.4628361030	-6.0787925848
O	-12.7483590748	4.7903607837	-4.4347871734
H	-11.9141760686	6.1034050178	-7.0191810579
CA	-15.3783779539	1.4660761734	-0.9807489940
C	-13.8148542023	5.7194434387	-0.9520471521
H	-14.3185770208	5.5869357659	0.0066481533
H	-14.4562734613	5.3269197535	-1.7449476793
O	-12.5691269825	4.9895157651	-0.9176735744
P	-12.4837136430	3.4015926545	-0.9387601757
O	-13.7279886323	2.6449107442	-1.3140069242
O	-11.2953976468	2.8730370202	-0.2023987762
O	-13.5180512612	3.6630866531	1.2666827283
P	-14.8384562725	3.0473414438	1.7465785056
O	-15.1913481588	1.6972522954	1.0570724119
O	-14.9879474830	3.0126394235	3.2695169092
O	-16.0195215604	4.1233696189	1.2088717086
P	-17.3169708314	3.7109014489	0.2797448575
O	-18.2266402205	2.8285403228	1.1817185336
O	-16.7932750269	2.9489941188	-0.9658034816
O	-17.9312931430	5.0692493350	-0.1234792760
C	-13.4951165268	7.1709118216	-1.2412853828
H	-14.4291948571	7.6721885036	-1.5342680273
O	-12.9672800796	7.8229075133	-0.0591317941
C	-12.0297844413	8.8247685078	-0.4190587804
H	-12.3556972051	9.7975329470	-0.0514841597
N	-10.7413177587	8.5558810896	0.2728024827
C	-10.2242165065	7.2838921739	0.2362752009
H	-10.8737738910	6.5256789977	-0.1780139433
C	-10.0337084172	9.6126068596	0.8369365065
O	-10.4442133379	10.7750951012	0.8611630864
N	-8.8011442836	9.2620179252	1.3620008532
H	-8.3619158250	9.9790940673	1.9592566975
C	-8.1992396679	8.0088219860	1.3153844294
O	-7.0613152653	7.8385638468	1.7991935376
C	-8.9871558037	6.9874383940	0.6966643402
H	-8.6175381325	5.9735789059	0.6449655523
C	-11.8991792789	8.7746543202	-1.9500317043
H	-10.8785154566	8.9509682419	-2.2918924803
H	-12.5466919844	9.5283600739	-2.3858931964
C	-12.4506965954	7.4066650578	-2.3421823361
H	-11.6669074195	6.6461348486	-2.2948353987
O	-13.0260271790	7.4159333101	-3.6380613177
H	-12.9698220371	6.4928475298	-3.9766301102
O	-9.4383261487	8.1755063795	4.7717586335
H	-10.0269176449	8.9085345347	4.4825520521
H	-8.5031869516	8.4246461485	4.6055570606
O	-15.3813554150	1.2186181831	5.1798039382
H	-15.3779057113	0.3548562887	4.7407408790
H	-15.2933168177	1.8727120401	4.4301792655
O	-9.7790000436	6.4310683062	6.8554038574
H	-10.5786344355	5.9001383499	6.6566044682
H	-9.6739075735	7.0620134254	6.1011203675
O	-11.7242339075	3.3630299065	-2.6509181764
H	-12.1964664141	3.9315368063	-3.4329357361

H	-11.7020315664	2.4198553621	-2.9096010175
O	-15.7832439652	-1.0774230384	-4.3364266000
H	-15.0119939316	-1.3087073606	-4.9030943253
H	-15.6634974566	-1.6150994198	-3.5068344676
O	-15.8654277241	12.0098357062	1.2265182340
H	-15.0726872643	12.1413129415	0.6600093899
H	-16.6021448107	11.9321803205	0.5836447249
O	-12.6395511267	0.9944328889	-4.9561950255
H	-12.1070462356	0.6503635620	-4.2148201431
H	-12.2891471143	1.8715122811	-5.2339070289
O	-17.0298070872	0.1299492020	-0.9902437999
H	-17.9114948850	0.4829321494	-0.7560464719
H	-17.1075864118	-0.8565173322	-1.0762388978
O	-15.3655247867	1.3744696281	-3.1476658824
H	-14.5355158768	1.6567705536	-3.5706992058
H	-15.6015875733	0.5402362847	-3.6216883699
O	-14.1534093139	-0.3284419525	-1.0761763052
H	-13.3703692640	-0.1723268650	-1.6419442864
H	-14.6220861912	-1.1112688395	-1.4778122003
C	-11.4380707628	5.5530338835	3.4295070867
H	-11.2358694190	6.4575420581	4.0062517893
H	-10.6287903230	5.4429199504	2.6958609299
O	-12.6940969629	5.7048291953	2.8012411435
H	-12.8975639686	4.9221462040	2.2258274402
H	-11.3993345772	4.6606965695	4.1107614310
C	-10.7797647061	-0.3808491291	-1.5841290834
O	-11.5424482876	0.0412067095	-2.4848296400
H	-10.4362787523	-1.4351510014	-1.5837146758
N	-10.3574297720	0.3643196345	-0.5723010057
H	-10.7078105140	1.3336363186	-0.4554836653
H	-9.7803187342	-0.0087983246	0.1801922927
C	-16.2961750921	7.2100008023	-5.1183638894
H	-15.7480398185	7.3164434963	-6.0580269547
H	-15.6474731919	7.5540563797	-4.3054811372
N	-16.6356488423	5.7938499728	-4.9547002785
H	-16.3895437763	5.1489161203	-5.7338090210
C	-16.8673273684	5.2203614900	-3.7547220364
N	-16.9302167169	5.9444262380	-2.6273017513
H	-17.2672385057	5.5143157264	-1.7507808013
H	-16.9906406572	6.9648123470	-2.6389000248
N	-17.0426523095	3.8989307428	-3.6754487341
H	-16.9738904197	3.4509941598	-2.7537866457
H	-16.9195113970	3.3133266427	-4.5295707737
H	-17.2180333496	7.8258734544	-5.1597918865
N	-13.9622693366	9.9839139161	3.5983656498
H	-14.7746056248	10.4061293739	3.1603752308
C	-13.9692159067	9.0032094210	4.5715581779
H	-14.8553725884	8.4583302591	4.8824598928
C	-12.6633846384	10.3354958952	3.3840197739
H	-12.3644273953	11.0789422702	2.6577615162
N	-11.8443126955	9.6376741182	4.1531423882
C	-12.6538139369	8.8088190221	4.9007360056
H	-12.2414553666	8.1200268717	5.6241625608
C	-20.6100575931	6.8170427673	0.8884333137
H	-21.5699034755	7.3206108349	0.7378686605
H	-19.8701935236	7.5614040852	1.2103627839
O	-20.2451546232	6.2202126353	-0.3536676761
H	-19.3626709308	5.7307153083	-0.2542203960
H	-20.7248279190	6.0672757118	1.7195498180
C	-20.7381527374	1.6070568777	-0.5153675364
H	-21.3306290432	0.8280352475	-1.0051325459
H	-20.4116832184	2.3226543367	-1.2820524951
O	-19.6214203207	0.9822221694	0.1083559928

H	-19.0431496574	1.7200586187	0.5426261636
H	-21.3857026785	2.1608438128	0.2157252898

109
RNase H reactant -4441.93726806178

C	40.4294273722	14.8696068097	8.5314211217
H	41.2319598512	15.2898652722	9.1400905342
H	40.8746403795	14.1231970396	7.8607252118
C	39.4329621687	14.1104599438	9.4048164230
O	38.2896649143	13.8040342348	8.9015012345
O	39.8154831248	13.7908481243	10.5482900805
H	39.9470183419	15.6635617514	7.9082044009
C	28.1764355982	16.8952669390	10.6890225537
H	27.1806284854	16.5888525116	10.3512020026
H	28.1247939139	17.0892045441	11.7638865021
H	28.4645640606	17.8282719763	10.1539944341
C	29.1751512511	15.7989921403	10.3778272395
O	29.2952478668	15.3121002025	9.2307453012
N	29.9604260861	15.3986307682	11.3974636997
H	30.7364198828	14.7766929417	11.2138263862
H	29.8846459811	15.8078401146	12.3396844988
C	33.7458175332	15.9060480141	7.9565867459
H	33.7367314853	16.0736974880	6.8736375506
H	32.8952694460	15.2704683495	8.2092824785
C	35.0706739982	15.2764675945	8.3257162924
O	35.0975302708	14.2647950693	9.2587245533
O	36.1277946156	15.6013815127	7.8297809693
H	33.6246724032	16.8826548140	8.4725675325
C	36.7494075264	9.2210999558	7.2133663433
H	37.6398589669	8.6051847304	7.0671289705
H	35.9940668623	8.6024191796	7.7151464782
C	37.1116349592	10.4038022540	8.1184704902
O	38.1964312983	10.3303462115	8.7458656638
O	36.2764909175	11.3654952372	8.2579540212
H	36.3652110431	9.5158102897	6.2075592104
N	41.1219101178	8.2492556046	14.8654892862
H	41.9674956503	7.8532317494	15.3193484122
C	40.1204570706	7.4996222808	14.2957087585
H	40.2208080914	6.4440562074	14.0863165223
C	40.7213912071	9.5365080480	14.8921187115
H	41.3251427013	10.3347384707	15.3020711998
N	39.5197218399	9.6568895783	14.3383352465
C	39.1387641218	8.3905209485	13.9530743221
H	38.1863336701	8.2193995361	13.4747746296
C	43.5334519482	12.1778773804	12.8529741038
H	43.4285781384	12.1113028986	13.9399084057
H	44.3262434890	11.5038327723	12.5184521062
C	42.1774888629	11.8205054960	12.2598646836
O	41.1626170264	12.3298262301	12.8223913594
O	42.1278312551	11.0531158611	11.2481342363
H	43.7827183236	13.2254510439	12.5556082374
C	33.2946890248	10.7314672005	9.8123022719
H	33.6379479368	11.2988474416	8.9364275919
O	31.8638456371	10.7023206634	9.8465084796
C	31.3481548478	11.6151513801	10.8509323173
H	30.5726606323	12.2150275212	10.3683670758
H	33.7042550841	9.7009067673	9.7486496122
H	30.8919025305	11.0818914731	11.6849680524
C	32.5574663335	12.4670770526	11.2581918064
H	32.5037497246	12.8910482238	12.2645032580
O	32.7620820564	13.5552384078	10.3319131774
H	34.2198713122	13.9832612917	9.7025346941
C	33.7026121250	11.4550216305	11.0970558241

H	33.7150261492	10.7706777200	11.9520817569
O	34.9774612354	12.0708564073	10.9883645772
P	36.1279285457	11.7794969473	12.2303395058
O	37.2537038671	12.4768190564	11.4183871448
O	36.0659954584	10.3369114807	12.5861510714
O	35.5682131330	12.7298383722	13.3987602504
C	35.4741799627	14.1535318066	13.1345866731
H	34.9324202763	14.3151478763	12.1991204244
H	36.4830404854	14.5604572363	13.0394731369
C	34.7437498189	14.8522409340	14.2562437946
H	34.9020531408	15.9325562262	14.1129872545
O	33.3418950751	14.5628893293	14.2189148042
C	32.8159591675	14.4996218057	15.5687651242
H	31.9727233762	15.1911660356	15.6406080920
H	32.4782061638	13.4973361610	15.8100568143
C	33.9701117963	14.9592481278	16.4893551517
H	33.9081504621	14.5030968762	17.4835974646
O	34.0368312110	16.3737247746	16.5890829416
H	33.7358799106	16.6822071293	17.4709183149
C	35.1765610415	14.4649071616	15.6723849505
H	35.2456448889	13.3756606478	15.7446633347
O	36.4391397715	15.0475155584	15.9981616981
P	37.6981194081	14.1642486673	16.5593532609
O	38.9331602351	14.5904322225	15.7705703163
O	37.3448663182	12.6787036105	16.5266140097
O	37.8376625976	14.5534429624	18.1024152195
C	37.6187615409	15.8412267762	18.7443039005
H	37.1855610645	16.5598706316	18.0423591422
H	38.5844095780	16.2049922193	19.0924460687
H	36.9343258897	15.6857103629	19.5946436040
O	38.1638998524	11.8021183625	13.7257651454
H	37.9890659792	12.2117163687	14.5955987359
H	38.6880626056	10.9431696111	13.9483038894
O	39.1111607407	14.5108986059	13.1127269843
H	38.7478988059	15.2911450906	12.6389641341
H	38.9958534131	14.6468720122	14.0900788122
O	39.4565287413	10.8255294530	11.1572367929
H	39.0029427485	10.7261358775	10.2925913007
H	40.4321302517	10.7457825584	11.0223578078
O	31.1418778329	13.6632334664	8.2384821684
H	30.4397890502	14.3072568782	8.5707344298
H	32.1314845921	13.4996143287	9.5458504431
MG	36.6689634702	12.8444860794	9.5312325035
MG	39.2386759180	12.6670863930	12.1517872215
O	37.2076880245	9.9213349207	16.3441971970
H	37.6874494070	9.7220645377	15.5213167989
H	37.1891158608	10.9035033342	16.3793969985
O	29.9196104693	11.3942845881	7.3392205301
H	30.6522195155	12.8362891855	7.9918515635
H	30.2037751474	10.5405585329	7.7261811860
H	28.9248765657	11.3031823066	7.2462156835

109
RNase H TS

C	40.4144134398	14.8857245203	8.4876204567
H	41.2209829279	15.3041970650	9.0936218966
H	40.8619599173	14.1465174938	7.8108487187
C	39.4306134016	14.1243050875	9.3830740644
O	38.2868543286	13.7584565352	8.9491855888
O	39.8810875393	13.8641474549	10.5282730848
H	39.9347712744	15.6871909901	7.8756450262
C	28.1741754318	16.8967283083	10.6891462915
H	27.1788713926	16.5910392055	10.3493448009

H	28.1206273865	17.0916462347	11.7637380807
H	28.4640435697	17.8288636002	10.1535767460
C	29.1717726313	15.7985357966	10.3769725750
O	29.2835995593	15.3136193265	9.2292877710
N	29.9614711859	15.3983179753	11.3931543693
H	30.7396164908	14.7777449287	11.2064616087
H	29.8845191872	15.8053974522	12.3353013890
C	33.7425229504	15.8842610421	7.9986692457
H	33.7134887665	16.0355993372	6.9148294631
H	32.9033743738	15.2430063025	8.2751612107
C	35.0744924657	15.2765249864	8.3681818847
O	35.1058303565	14.2815325292	9.2996776404
O	36.1345304573	15.6191247321	7.8752596245
H	33.6199211055	16.8662339039	8.5021559138
C	36.7580796266	9.2124511605	7.2083063545
H	37.6377200677	8.5832987715	7.0563920557
H	36.0018106247	8.6008476801	7.7174050052
C	37.1343146691	10.3948452315	8.1141214872
O	38.2075087347	10.2853035870	8.7719463687
O	36.3317540838	11.3740173565	8.2296172293
H	36.3681836225	9.5069261126	6.2039862722
N	41.0068890232	8.3141203816	14.8445090464
H	41.8732577061	7.9533373374	15.2943280196
C	40.0429944515	7.5058262671	14.2835137701
H	40.1965196802	6.4508763397	14.0828322594
C	40.5409692209	9.5759009167	14.8750017041
H	41.0957604856	10.4045706962	15.2912161372
N	39.3177664554	9.6293437503	14.3317249838
C	39.0036109349	8.3334029220	13.9565354402
H	38.0476002249	8.0954453838	13.5192707628
C	43.5256052494	12.1792794465	12.8626074218
H	43.4233430230	12.1154081399	13.9499593164
H	44.3121077229	11.5002455703	12.5240426771
C	42.1705835746	11.8226576874	12.2753658674
O	41.1535243138	12.3336614945	12.8497886975
O	42.1103524407	11.0592458844	11.2683207718
H	43.7780397241	13.2249300735	12.5611092676
C	33.3028695721	10.7474411342	9.7849165260
H	33.6179254994	11.2895071966	8.8826631648
O	31.8690843117	10.7029759394	9.8416963846
C	31.3612082750	11.6160637807	10.8479962494
H	30.5825734472	12.2104302409	10.3635991649
H	33.7212347385	9.7176376801	9.7424466370
H	30.9037391029	11.0844642192	11.6849208646
C	32.5653965942	12.4800983526	11.2463028512
H	32.5212470646	12.8797490866	12.2638932148
O	32.7151642752	13.6076721075	10.3522833118
H	34.2350597906	13.9669396142	9.7422831694
C	33.7675648451	11.5265500923	11.0301025569
H	33.8206937237	10.8502921905	11.8957893908
O	34.9896818946	12.1759571048	10.8880966695
P	36.4536277973	11.7771922086	12.5197835100
O	37.3563182035	12.5114686918	11.4990653578
O	36.1158315500	10.3271817404	12.6047900421
O	35.6345379899	12.7666076742	13.4769012517
C	35.4827175288	14.1791912914	13.1397972707
H	34.9360409680	14.2603189064	12.2003173833
H	36.4715215438	14.6302611156	13.0233151935
C	34.7313430700	14.8751472398	14.2501668263
H	34.8771898384	15.9563439381	14.1077589641
O	33.3350867080	14.5714834119	14.2168882849
C	32.8120959338	14.5058520134	15.5666322104
H	31.9651990056	15.1932538468	15.6434813849

```
 H          32.4783208119       13.5015595133       15.8063937382
 C          33.9661363146       14.9716599729       16.4891592876
 H          33.9106897627       14.5071139299       17.4800365930
 O          34.0276638996       16.3847707782       16.5997527959
 H          33.7211048476       16.6880809042       17.4822718716
 C          35.1676818969       14.4880779159       15.6629299840
 H          35.2366479338       13.3991869587       15.7290362066
 O          36.4402874215       15.0649257932       15.9748179951
 P          37.7024534641       14.1780708569       16.5019771751
 O          38.9447194899       14.6551590698       15.7576057849
 O          37.3876311012       12.6799897395       16.3667227879
 O          37.8018244647       14.4884390241       18.0666337487
 C          37.6261911467       15.7905091976       18.7160774177
 H          37.2043221009       16.5167570752       18.0151458064
 H          38.6039533735       16.1300635439       19.0558685014
 H          36.9441903651       15.6583711269       19.5723007458
 O          37.9364093561       11.7419807416       13.7423464136
 H          37.7156675008       12.1510822977       14.6287695934
 H          38.4931153186       10.8111758474       13.9477883273
 O          39.0657224448       14.4885330431       13.1198098067
 H          38.7089112543       15.2687388505       12.6359541296
 H          38.9890533752       14.6581539469       14.0957960286
 O          39.4433109140       10.8511668948       11.1560964246
 H          38.9720445786       10.7337367574       10.2961863633
 H          40.4117189361       10.7276307799       11.0182451078
 O          31.1275380169       13.6660555825        8.2080879520
 H          30.4274496725       14.3050731746        8.5448934399
 H          32.1166545218       13.5215213823        9.5515817216
MG          36.5494157425       12.7666411235        9.6495200045
MG          39.3236482973       12.7384136431       12.0515549580
 O          37.2707841748        9.8867295646       16.3891230126
 H          37.9197361689        9.7006957267       15.6846787701
 H          37.2196536023       10.8653780361       16.4212488058
 O          29.9201280529       11.3856518602        7.3310470136
 H          30.6423166239       12.8335535162        7.9755833678
 H          30.2113983284       10.5413867918        7.7331590415
 H          28.9257217663       11.2915499078        7.2455368264
```

```
29
phosphatase model reactant
C   1.55316  -2.01790   -6.10301
H   1.72934  -1.75633   -5.05963
H   0.64867  -1.50102   -6.43351
H   1.39857  -3.09596   -6.24833
C   2.77592  -1.61317   -6.90629
O   3.92412  -1.80541   -6.46608
N   2.57296  -1.04317   -8.10790
H   3.37579  -0.76445   -8.68142
H   1.62708  -0.87042   -8.45826
C   6.06088   1.65768  -15.76039
H   6.15402   0.57024  -15.66592
H   5.25354   1.99490  -15.09897
O   7.28653   2.29796  -15.39435
P   8.01851   1.92810  -13.98457
O   9.39152   2.53970  -14.05326
O   7.85076   0.43471  -13.68633
O   7.02117   2.74943  -12.97110
P   7.14204   2.79064  -11.31944
O   6.71352   4.18956  -10.90879
O   8.53459   2.31813  -10.96614
O   6.01616   1.73032  -10.89117
P   5.97764   0.69770   -9.49614
O   5.53763   1.63254   -8.35760
```

O	7.40437	0.15985	-9.36027
O	4.91225	-0.29922	-9.93626
H	5.81644	1.91390	-16.80707
O	5.92735	-2.82102	-7.97331
H	5.20850	-2.27067	-7.58905
H	6.36367	-3.20761	-7.19147

29
phosphatase model TS

C	1.56358	-2.24168	-5.95739
H	1.76751	-1.90451	-4.94110
H	0.64753	-1.74776	-6.28840
H	1.41136	-3.32733	-6.01987
C	2.76053	-1.89538	-6.81437
O	3.90779	-2.22875	-6.44456
N	2.54957	-1.22506	-7.95867
H	3.34565	-0.98508	-8.55920
H	1.60632	-0.97627	-8.26779
C	6.05588	1.66216	-15.79850
H	6.11933	0.57138	-15.70655
H	5.25977	2.01775	-15.13227
O	7.29898	2.26552	-15.43992
P	7.98394	1.90990	-13.99384
O	9.36699	2.50887	-14.04221
O	7.83669	0.39674	-13.73414
O	6.96110	2.68969	-13.02164
P	6.97108	2.65363	-11.31766
O	6.62934	4.11008	-10.94954
O	8.40639	2.23436	-10.97307
O	5.89316	1.63881	-10.90709
P	6.10950	0.09733	-8.99717
O	5.90447	1.29074	-8.08779
O	7.52165	-0.29366	-9.41853
O	4.93101	-0.61418	-9.61106
H	5.81212	1.92525	-16.84368
O	6.17057	-1.24493	-7.30371
H	5.28293	-1.63941	-7.05058
H	6.85164	-1.91296	-7.06621

28
pyrophosphatase model reactant

Mg	-15.27795815	1.46622724	-1.08643227
C	-12.95523700	5.77684100	-6.81084000
H	-13.55380800	6.57753000	-6.36669000
H	-13.46965900	5.38841400	-7.69484900
C	-12.72850800	4.62807600	-5.80619500
O	-12.26523500	3.54750400	-6.25402400
O	-12.98172600	4.85420900	-4.58428200
H	-11.95869900	6.18105300	-7.09858800
C	-13.83253400	5.70778600	-0.88519600
H	-14.51597781	5.68511119	-0.06221944
H	-14.28733492	5.25090926	-1.73919821
O	-12.63239500	4.98487900	-0.53439200
P	-12.74600100	3.42812500	-0.13640100
O	-13.64167100	2.66679200	-1.09321300
O	-11.37882700	2.90889400	0.19287200
O	-13.54785500	3.57860900	1.32472900
P	-14.98891000	2.98473200	1.79269900
O	-15.24259300	1.66340500	1.07808300
O	-14.99193400	3.01410400	3.30045900
O	-16.02459300	4.10371600	1.23758100
P	-17.34960400	3.68940800	0.25680000
O	-18.21444200	2.79835000	1.18183800

O	-16.76272800	2.94967300	-0.95977000
O	-17.93701300	5.05684600	-0.10440800
O	-11.50408000	2.81816600	-3.29145900
H	-12.03332000	3.53973300	-3.69565000
H	-12.05635000	2.47255400	-2.58099100
H	-13.58340655	6.72289976	-1.11406183

28

pyrophosphatase model TS

Mg	-15.37837795	1.46607617	-0.98074899
C	-12.87562302	5.64716271	-6.65450284
H	-13.48716864	6.41658095	-6.18101929
H	-13.42550930	5.22504693	-7.49879496
C	-12.52361379	4.53398505	-5.64826766
O	-12.01595360	3.47384236	-6.06529098
O	-12.73865864	4.78878338	-4.40666465
H	-11.92455957	6.10410634	-7.01235766
C	-13.83351764	5.73897006	-0.94306369
H	-14.37916258	5.64081490	-0.02789312
H	-14.41681908	5.34694573	-1.74989474
O	-12.59235172	5.00518499	-0.84498083
P	-12.50886412	3.40905991	-0.88709489
O	-13.75480923	2.66321196	-1.28798581
O	-11.33141434	2.87070482	-0.13450873
O	-13.53417152	3.65114171	1.28117026
P	-14.85916342	3.03444552	1.74589014
O	-15.20782722	1.68379025	1.05680341
O	-14.99395473	3.01285903	3.26704724
O	-16.02825150	4.11337158	1.19902049
P	-17.32770663	3.70278482	0.27537569
O	-18.23350048	2.82195281	1.18239079
O	-16.81245108	2.93796746	-0.97219202
O	-17.93725925	5.06465253	-0.12633574
O	-11.74322770	3.38278056	-2.55944909
H	-12.19232655	3.95060465	-3.37104375
H	-11.74125721	2.44231665	-2.84575749
H	-13.62351897	6.77243721	-1.12402284

Note 1: ECs associated to superfamily members

# Phosphatases (Pγ leaving group): 42 SFs

ATPase domain of HSP90 chaperone/DNA topoisomerase II/histidine kinase: 3.6.1.8, 2.7.11.2, 2.7.11.4, 4.6.1.2, 2.7.13.1, 2.7.11.1, 3.6.4.10, 2.7.13.3, 2.7.10.2, 4.6.1.1, 2.7.13.2, 5.6.1.2
Carbamate kinase-like: 2.7.2.8, 2.7.2.11, 2.7.4.26, 2.7.4.22, 2.7.2.4, 2.7.2.2, 2.7.4.4, 2.7.4.31, 2.7.2.19, 2.7.2.17
GHMP Kinase C-terminal domain: 4.1.1.33, 2.7.4.2, 2.7.1.39, 4.6.1.2, 2.7.1.148, 2.7.1.167, 2.7.6.5, 2.7.1.36, 2.7.1.169, 2.7.1.6, 2.7.7.30, 2.7.1.185, 2.7.1.46, 2.7.1.52, 4.1.1.99, 2.7.1.43, 2.7.1.44, 2.7.1.157, 2.7.1.177, 2.7.1.168, 6.3.2.49, 2.7.11.1, 3.6.4.10, 3.6.4.13, 2.7.1.71
Nucleoside diphosphate kinase NDK: 2.7.4.6, 2.7.13.3
GroEL equatorial domain-like: 5.6.1.7
ParB/Sulfiredoxin: 2.7.1.225, 1.8.98.2
NAD kinase/diacylglycerol kinase-like: 2.7.1.107, 2.7.1.86, 2.7.1.91, 2.7.1.23, 2.7.1.138, 2.7.1.94, 2.7.1.93
Riboflavin kinase-like: 2.7.1.26, 2.7.7.2, 2.7.1.161
PurM N-terminal domain-like: 2.7.9.3, 6.3.3.1, 6.3.5.3, 2.7.4.16, 6.1.1.24, 6.3.4.13
CYTH-like phosphatases: 4.6.1.1, 2.7.7.50, 3.6.1.28
CofE-like: 6.3.2.34, 6.3.2.31
Actin-like ATPase domain: 4.6.1.1, 2.7.1.30, 2.7.1.4, 2.7.1.5, 2.7.2.7, 2.7.1.59, 2.7.1.189, 2.7.1.170, 2.7.2.15, 2.7.1.60, 2.7.1.55, 2.7.1.58, 2.7.1.12, 2.7.2.1, 2.7.1.16, 2.7.1.17, 2.7.1.51, 2.7.1.1, 2.7.1.33, 2.7.1.2, 2.7.1.47, 2.7.1.14, 1.3.7.8, 2.7.1.215, 2.7.1.179, 2.7.1.8, 2.7.1.232, 2.7.1.233, 6.1.2.2, 2.7.1.214, 2.7.1.7, 2.7.1.188, 2.7.1.53, 3.5.2.14, 6.4.1.8, 2.7.1.85, 2.7.1.157, 2.7.1.27, 3.5.2.9, 3.6.1.40, 3.6.1.11, 2.7.10.2, 2.7.11.1, 6.3.4.15, 1.16.99.1, 2.7.4.1, 6.4.1.6, 2.7.1.34
Glutamine synthetase/guanido kinase: 6.3.1.11, 6.3.2.3, 6.3.2.2, 2.7.3.2, 2.7.3.5, 2.7.14.1, 2.7.3.3, 6.3.1.2, 2.7.3.4, 2.7.3.1, 6.3.4.12, 6.3.1.6, 6.3.1.18, 6.3.1.19, 6.3.5.6, 6.3.5.7, 2.7.3.10, 2.7.3.6, 2.7.3.7, 2.7.3.8
GlnB-like: 6.3.5.10
Phosphoglycerate kinase: 2.7.2.3, 2.7.2.10
Phosphoenolpyruvate/pyruvate domain: 2.7.1.40, 2.7.9.2, 2.7.9.1, 2.7.7.104, 6.2.1.9, 2.7.10.2, 2.7.11.1, 2.7.1.238
Phosphofructokinase: 2.7.1.11, 2.7.1.184, 2.7.1.144
P-loop containing nucleoside triphosphate hydrolases: 3.6.1.8, 7.1.2.2, 2.5.1.17, 2.7.12.2, 2.7.1.164, 2.7.1.145, 2.7.4.25, 2.7.4.13, 2.7.11.17, 2.7.4.8, 2.7.1.76, 2.7.4.2, 2.7.1.105, 2.7.4.9, 3.6.5.5, 2.7.10.2, 6.3.4.3, 6.3.4.4, 2.7.1.71, 2.7.1.12, 3.6.5.2, 2.7.1.48, 2.7.4.3, 2.7.4.10, 2.7.1.19, 2.7.4.14, 2.7.1.78, 2.7.1.113, 6.3.3.3, 2.7.1.130, 3.6.5.1, 2.7.1.21, 3.1.5.1, 2.7.1.25, 2.7.1.33, 2.7.1.74, 2.7.4.1, 2.7.1.24, 3.6.5.4, 2.7.11.13, 6.3.4.2, 2.7.1.67, 6.3.5.12, 6.3.3.7, 6.3.4.25, 1.3.7.15, 6.3.5.11, 2.7.1.176, 2.7.4.23, 2.7.2.16, 2.7.1.156, 2.7.4.34, 2.7.1.224, 6.3.5.9, 2.7.1.186, 7.3.2.6, 6.3.5.10, 2.7.4.4, 3.6.1.15, 2.7.11.1, 3.6.5.3, 2.7.6.2, 7.2.2.1, 3.6.4.6, 7.6.2.5, 7.4.2.8, 7.6.2.4, 5.6.1.1, 7.2.2.16, 7.5.2.4, 7.5.2.3, 7.6.2.2, 5.6.1.8, 5.6.1.6, 7.5.2.6, 7.4.2.6, 3.6.4.13, 2.7.13.3, 7.6.2.10, 2.7.1.23, 7.3.2.2, 7.6.2.11, 7.6.2.6, 7.4.2.4, 7.6.2.14, 7.3.2.3, 4.6.1.1, 7.6.2.12, 7.3.2.5, 7.2.2.7, 7.2.2.20, 7.5.2.13, 7.5.2.7, 7.2.2.11, 7.6.2.9, 7.5.2.9, 2.3.1.193, 7.4.2.12, 7.6.2.8, 7.6.2.16, 7.4.2.10, 7.2.2.18, 7.4.2.11, 7.2.2.4, 7.6.2.7, 7.5.2.10, 1.18.6.1, 7.3.2.1, 7.4.2.13, 7.5.2.11, 7.2.2.17, 7.3.2.7, 7.5.2.12, 7.4.2.9, 7.6.2.13, 7.5.2.5, 7.4.2.7, 2.7.2.1, 7.6.2.3, 7.4.2.14, 7.5.2.1, 7.3.2.4, 7.2.2.2, 5.6.1.4, 7.6.2.15, 7.4.2.5, 7.4.2.1, 7.5.2.8, 7.2.2.5, 5.6.1.3, 5.6.1.5, 3.6.4.7, 1.18.6.2, 1.19.6.1, 2.7.1.18, 2.7.1.236, 2.7.10.3, 2.7.4.11, 2.7.4.12, 2.7.4.15, 2.7.4.19, 5.6.1.9, 7.4.2.2, 7.4.2.3, 7.5.2.14, 7.5.2.2
Tubulin nucleotide-binding domain-like: 3.6.5.6, 6.3.2.25
PEP carboxykinase-like: 4.1.1.32, 4.1.1.49
Metal cation-transporting ATPase ATP-binding domain N: 7.2.2.8, 7.2.2.10, 7.6.2.1, 7.2.2.19, 7.2.2.13, 7.2.2.6, 7.2.2.14, 7.1.2.1, 7.6.2.16, 7.2.2.9, 7.2.2.3, 7.2.2.12, 7.2.2.15, 7.2.2.21, 7.2.2.22

MurD-like peptide ligases catalytic domain: 6.3.5.10, 6.3.2.10, 6.3.2.17, 6.3.2.12, 6.3.5.13, 6.3.2.9, 6.3.2.37, 6.3.2.8, 6.3.2.7, 6.3.2.13, 6.3.2.29, 6.3.2.30, 6.3.2.53, 6.4.1.9, 6.3.2.45, 5.1.1.23, 6.3.2.16
Inositol-pentakisphosphate 2-kinase: 2.7.1.158
YcaO-related McrA-glycine thioamidation protein: 6.2.2.3, 6.2.2.1, 6.2.2.2
Peptidase G2 IMC autoproteolytic cleavage domain: N/A
Ribokinase-like: 2.7.1.64, 2.7.1.4, 2.7.1.11, 4.2.1.93, 2.7.1.35, 2.7.1.83, 2.7.1.20, 2.7.1.73, 2.7.1.15, 2.7.1.3, 2.7.1.49, 2.7.1.50, 2.7.1.184, 2.7.4.7, 2.7.1.187, 2.7.1.144, 2.7.1.45, 2.7.1.2, 2.7.1.218, 2.7.1.223, 2.7.1.92, 2.7.7.70, 2.7.1.178, 2.7.1.213, 2.7.1.13, 2.7.1.101, 6.4.1.8, 2.7.1.58, 6.3.1.12, 2.7.1.56, 2.7.1.239, 2.7.1.234, 2.7.1.168
DhaL-like: 2.7.1.29, 2.7.1.210, 2.7.1.209, 2.7.1.28, 2.7.2.18
Calcium-dependent phosphotriesterase: 3.1.3.1, 2.7.13.3
Protein kinase-like (PK-like): 2.7.1.153, 6.5.1.1, 2.7.10.1, 2.7.11.11, 2.7.12.2, 2.7.11.24, 2.7.11.22, 2.7.11.21, 2.7.1.230, 2.7.11.14, 2.7.1.190, 2.7.11.17, 2.7.1.183, 2.7.11.7, 2.7.1.39, 2.7.11.15, 2.7.10.2, 2.7.1.82, 2.7.11.30, 2.7.1.95, 2.7.11.10, 2.7.11.31, 2.7.1.23, 2.7.1.154, 2.7.1.3, 2.7.1.163, 2.7.1.32, 2.7.1.17, 2.7.11.23, 2.7.1.137, 2.7.1.162, 2.7.1.119, 2.7.1.100, 2.7.11.25, 2.7.11.12, 2.7.11.19, 2.7.1.1, 2.7.12.1, 2.7.1.22, 2.7.11.26, 2.7.11.13, 2.7.1.67, 2.7.1.175, 2.7.1.89, 2.7.1.72, 2.7.1.87, 2.7.1.166, 2.7.1.8, 2.7.1.181, 2.7.1.222, 2.7.1.103, 2.7.1.221, 2.7.1.81, 2.7.1.65, 2.7.1.156, 2.7.11.1, 2.7.11.18, 2.7.1.235, 2.7.11.20, 2.7.13.3, 2.7.11.34, 2.7.11.5, 2.7.1.171, 2.7.1.172, 2.7.11.16, 2.7.7.108, 2.7.1.136, 2.7.1.88, 2.7.11.28, 2.7.11.29, 2.7.11.3, 2.7.11.35, 2.7.11.6, 2.7.11.9
Glutathione synthetase ATP-binding domain-like: 2.3.3.8, 6.4.1.4, 6.3.5.5, 6.3.4.16, 6.4.1.1, 6.4.1.3, 6.3.2.3, 6.3.2.2, 6.3.1.9, 2.7.9.2, 6.3.3.1, 6.3.2.23, 2.7.1.159, 6.4.1.2, 6.3.4.6, 6.3.1.8, 6.1.2.1, 6.3.4.13, 2.7.4.21, 6.3.4.14, 6.4.1.7, 2.7.9.1, 6.3.4.18, 2.7.1.134, 2.7.4.24, 6.3.3.5, 6.2.1.56, 6.2.1.5, 6.3.2.4, 6.3.4.23, 2.7.3.13, 6.3.2.32, 6.2.1.9, 6.3.1.17, 6.3.2.29, 6.3.2.48, 6.2.1.39, 6.3.2.47, 6.3.2.49, 6.3.2.43, 6.3.2.11, 6.3.2.30, 6.3.1.21, 6.3.4.24, 6.3.1.12, 6.3.2.59, 6.3.2.42, 6.3.2.41, 6.2.1.13, 6.3.2.33, 6.2.1.18, 6.4.1.5, 2.7.1.58, 6.2.1.4, 2.7.9.6, 2.7.9.5, 2.7.13.3, 2.7.9.4, 6.3.2.35, 6.3.2.61, 6.3.4.26, 6.3.2.65, 6.3.2.44, 6.3.2.62
SAICAR synthase-like: 2.7.1.127, 2.7.1.151, 2.7.1.149, 2.7.1.140, 2.7.1.68, 2.7.4.21, 6.3.2.6, 2.7.1.150, 2.7.1.153, 2.7.11.1
Diacylglycerol kinase (DgkA)-like (prokarytic): 2.7.1.10
Metallo-dependent phosphatases: 3.1.3.1, 3.6.1.15, 2.7.11.5, 3.6.1.5
Glycerate kinase I: 2.7.1.31, 2.7.1.165
YgbK-like: 2.7.1.219, 2.7.1.231, 2.7.1.217, 2.7.1.220
NagB/RpiA/CoA transferase-like: 6.3.3.2, 3.6.5.3, 2.7.1.29, 6.3.4.22
tRNA(Ile2) 2-agmatinylcytidine synthetase TiaS: 6.3.4.22
FomD-like: 3.6.1.15, 3.6.1.63
Phospholipase D/nuclease: 2.7.4.1
Nicotinate/Quinolinate PRTase C-terminal domain-like: 6.3.1.10, 3.5.2.14, 6.3.4.21
Apyrase: 3.6.1.5, 3.6.1.42, 3.6.1.6
Transglutaminase two C-terminal domains: N/A

# Pyrophosphatases (PβPγ leaving group): 44 SFs

PRTase-like: 2.7.6.1, 6.3.4.19, 3.5.2.14, 6.3.2.47
all-alpha NTP pyrophosphatases: 3.6.1.8, 3.6.1.12, 3.6.1.23, 3.6.1.31
Adenine nucleotide alpha hydrolases-like: 2.8.1.13, 6.3.1.5, 2.7.7.2, 6.3.5.1, 6.3.4.5, 6.3.5.4, 6.3.5.2, 2.7.7.4, 6.3.4.19, 6.3.3.4, 6.3.4.20, 4.4.1.37, 6.3.1.14, 5.1.1.23, 6.3.3.6, 2.7.1.108, 6.3.4.24, 2.8.1.15, 2.7.13.3, 2.8.1.14, 2.7.11.1
Nucleotide-diphospho-sugar transferases: 2.7.7.68, 2.7.7.62, 2.7.7.24, 2.7.7.40, 2.7.7.64, 3.6.5.2, 2.7.7.38, 2.7.7.43, 2.7.7.60, 2.7.7.27, 2.7.7.13, 2.7.7.33, 2.7.7.74, 2.7.7.23, 2.7.7.9, 2.7.7.103, 2.7.7.83, 2.7.7.91,

2.7.7.77, 2.7.7.106, 2.7.7.71, 2.7.7.90, 2.7.7.81, 2.7.7.76, 2.7.7.82, 2.7.7.99, 2.7.7.105, 2.7.7.92, 6.4.1.8, 2.7.1.52, 2.7.1.168, 4.6.1.17, 2.7.7.107, 2.7.7.11, 2.7.7.28, 2.7.7.32, 2.7.7.34, 2.7.7.44
Activating enzymes of the ubiquitin-like proteins: 2.7.7.80, 2.7.7.73, 2.7.7.100, 6.2.1.64, 6.2.1.45, 6.2.1.55
Phosphopantoate/pantothenate synthetase superfamily: 6.3.2.36
Virion DNA-directed RNA polymerase domain: 2.7.7.6
6-hydroxymethyl-7,8-dihydropterin pyrophosphokinase HPPK: 6.3.2.12, 2.7.6.3
Class II aaRS and biotin synthetases: 6.1.1.4, 6.1.1.14, 6.1.1.15, 6.1.1.20, 6.1.1.6, 6.3.4.15, 6.1.1.11, 6.3.1.1, 6.1.1.7, 6.1.1.27, 6.1.1.21, 6.1.1.26, 6.1.1.22, 6.3.1.20, 6.1.1.17, 6.1.1.23, 6.1.1.12, 6.3.4.11, 6.3.4.9, 6.3.4.10, 6.1.1.3, 2.7.7.75, 2.7.11.1, 6.2.1.14, 2.7.1.33, 6.3.1.4
Nudix: 6.3.2.25, 3.6.1.23, 2.7.7.1, 3.6.1.9, 2.7.7.96, 3.6.1.65, 2.7.6.2, 3.6.1.67, 3.6.1.69, 3.6.1.55, 3.6.1.56, 3.6.1.52, 3.6.1.58, 3.6.1.64
beta and beta-prime subunits of DNA dependent RNA-polymerase: 2.7.7.6, 2.7.7.48, 3.6.1.15
tRNA-splicing ligase RtcB-like superfamily: 6.5.1.8
Aerobactin siderophore biosynthesis IucA/IucC-like: 6.3.2.38, 6.3.2.39, 6.3.2.54, 6.3.2.55, 6.3.2.56, 6.3.2.57, 6.3.2.58, 6.3.2.63, 6.3.2.64
dUTPase-like: 3.6.1.23, 3.5.4.30, 2.7.7.49
Nucleotidylyl transferase: 6.1.1.1, 6.1.1.4, 6.1.1.10, 6.3.2.1, 6.1.1.16, 6.1.1.6, 2.7.1.26, 2.7.7.3, 6.1.1.5, 2.7.7.1, 2.7.7.15, 2.7.7.2, 2.7.7.39, 6.3.1.13, 2.7.1.25, 2.7.7.18, 6.1.1.9, 6.1.1.2, 6.1.1.19, 2.7.7.14, 2.7.7.4, 6.1.1.17, 6.1.1.18, 2.7.7.70, 2.7.7.104, 2.7.7.93, 6.2.1.22, 6.4.1.8, 6.1.1.24, 2.7.1.237, 3.6.1.73, 6.1.1.15, 2.7.1.24, 2.7.7.109, 2.7.7.110
ITPase-like: 3.6.1.15, 3.6.1.66, 3.6.1.73
CoaB-like: 6.3.2.51, 6.3.2.5
EPT/RTPC-like: 6.5.1.4, 6.5.1.5, 2.7.1.71
YrdC/RibB: 2.7.7.87, 3.5.4.25
YojJ-like: 2.7.7.85
BioW-like: 6.2.1.14
Poly(A) polymerase catalytic subunit-like: 2.7.7.19
DNA/RNA polymerases: 2.7.7.7, 2.7.7.6, 2.7.7.4, 2.7.7.48, 3.6.1.15, 3.6.4.13, 2.7.7.49, 2.7.7.50, 2.7.7.19
Nucleotidyltransferase: 2.7.7.7, 4.6.1.1, 2.7.7.52, 2.7.7.72, 2.7.7.47, 2.7.7.42, 2.7.7.84, 2.7.6.5, 2.7.7.19, 2.7.1.24, 2.7.7.59, 2.7.7.46, 2.7.7.65, 2.7.7.31, 2.7.7.85, 2.7.7.108, 2.7.7.86, 2.7.7.61, 2.7.7.66, 2.7.7.57
Nucleotide cyclase: 4.6.1.1, 2.7.7.65, 4.6.1.2, 4.6.1.6, 4.6.1.26, 2.7.11.1, 3.5.4.29, 2.7.7.79
Prim-pol domain: 2.7.7.7
Influenza RNA-dependent RNA polymerase subunit PB1: 2.7.7.48
RNA-dependent RNA polymerase eukaryotic-type: 2.7.7.48
Fic-like: 2.7.7.1, 2.7.11.1, 2.7.7.108
Intramembrane CDP-alcohol synthase: 6.3.2.39, 2.7.1.66, 2.7.7.67, 2.7.1.174, 2.7.1.182, 2.7.1.216, 2.7.7.41
Molybdenum cofactor biosynthesis proteins: 2.7.7.75, 2.7.7.76, 4.6.1.17
Adenylylcyclase toxin (the edema factor): 4.6.1.1
DNA primase core: 2.7.7.101
Acetyl-CoA synthetase-like: 1.13.12.7, 6.2.1.8, 6.2.1.12, 6.2.1.1, 6.2.1.57, 6.2.1.20, 6.2.1.2, 6.1.1.13, 6.2.1.33, 6.2.1.26, 5.1.1.11, 6.2.1.32, 6.2.1.30, 6.2.1.25, 6.2.1.3, 6.2.1.53, 6.2.1.51, 6.2.1.72, 6.2.1.70, 6.2.1.50, 6.2.1.35, 6.2.1.46, 6.3.2.40, 2.7.7.97, 6.3.2.50, 6.2.1.66, 6.2.1.27, 6.1.3.1, 6.2.1.48, 6.3.1.15, 6.2.1.44, 6.2.1.7, 6.2.1.47, 6.7.1.1, 6.2.1.62, 6.2.1.15, 6.2.1.40, 6.2.1.17, 6.2.1.65, 6.2.1.61, 6.2.1.68, 6.2.1.63, 6.2.1.24, 6.3.2.20, 6.2.1.76, 6.3.2.14, 6.2.1.37, 6.2.1.16, 6.2.1.71, 6.2.1.60, 6.2.1.43, 6.2.1.42, 6.2.1.41, 6.2.1.13, 6.2.1.75, 6.2.1.69, 1.2.1.95, 6.3.2.46, 6.2.1.31, 6.2.1.23, 6.2.1.19, 1.3.7.8, 6.2.1.67, 6.3.2.26, 6.2.1.59, 6.2.1.49, 6.2.1.54, 6.3.2.52, 6.7.1.2, 6.2.1.36, 6.2.1.77, 6.2.1.78, 6.4.1.10, 6.4.1.11, 6.4.1.12, 6.2.1.10, 6.2.1.11, 6.2.1.28, 6.2.1.38, 6.2.1.52, 6.2.1.58, 6.2.1.6, 6.2.1.73, 6.2.1.74

Alkaline phosphatase-like: 3.6.1.9, 3.1.3.1, 3.6.1.29
Thiamin pyrophosphokinase catalytic domain: 2.7.6.2, 2.7.6.3
DNA ligase/mRNA capping enzyme catalytic domain: 6.5.1.1, 2.7.7.50, 6.5.1.6, 6.5.1.3, 6.5.1.7, 2.7.7.48, 2.7.1.78, 2.7.7.88
RibA-like: 3.5.4.25
HIT-like: 2.7.7.53, 2.7.7.10, 6.3.2.39, 3.6.1.29
S-adenosyl-L-methionine-dependent methyltransferases: 2.7.7.19, 2.7.7.48, 3.1.3.84, 3.4.19.12, 3.6.1.15, 3.6.1.74, 3.6.4.13
Phosphatidate cytidylyltransferase, mitochondrial: 2.7.7.41
Molybdenum cofactor biosynthesis protein C MoaC: 2.7.7.77, 4.6.1.17
GTP cyclohydrolase MptA: 3.5.4.39
RPB5-like RNA polymerase subunit: N/A

# Triphosphatases (PαPβPγ leaving group): 4 SFs

S-adenosylmethionine synthetase: 2.5.1.6
Cobalamin adenosyltransferase-like: 2.5.1.17, 2.5.1.154
Tetrahydrobiopterin biosynthesis enzymes-like: 4.2.3.12, 4.1.2.50
HD-domain/PDEase-like: 6.1.1.14, 2.7.6.5, 3.1.5.1, 2.7.7.59, 3.6.1.40, 3.6.1.11, 2.7.7.18, 2.7.4.1

Note 2: NTP processing ECs with no associated superfamily

Several individual ECs originally lacking SF assignment could be placed by combining sequence and AlphaFold-based structural alignment. Notable examples include the macrolide 2'-kinase MphB (EC 2.7.1.136), confirmed as PK-like by TM-align to MphA (TM = 0.87, RMSD 2.4 Å vs PDB 5IGI); the bacteriophage 5-methyl-dCMP kinase (EC 2.7.4.19), assigned to P-loop NTPases via its structural and substrate identity to T4 dNMP kinase gp1 (PDB 1DEK); and the N-methylphosphoethanolamine cytidylyltransferase (EC 2.7.7.57), assigned to the Nucleotidyltransferase SF through its closest homolog ECT1 (TM = 0.68 to PDB 4MVC). 17 ECs remain without confident SF assignment after verification (literature, KEGG/UniProt sequence searches, AlphaFold + TM-align).
We also excluded FAST kinases (EC 2.7.11.8), as this is not NTP processing — the EC is miscategorized.
The remaining uncategorized ECs are:
2.7.1.54 2.7.1.84 2.7.1.102 2.7.1.122 2.7.2.6 2.7.2.14 2.7.4.18 2.7.4.32 2.7.6.4 2.7.7.45
3.6.1.39 6.3.1.7 6.3.2.24 6.3.2.60 6.3.4.7 6.3.4.8 6.3.4.17

References

1. Gough, J.; Karplus, K.; Hughey, R.; Chothia, C., Assignment of homology to genome sequences using a library of hidden Markov models that represent all proteins of known structure. *J Mol Biol* **2001,** *313* (4), 903-19.
2. Pandurangan, A. P.; Stahlhacke, J.; Oates, M. E.; Smithers, B.; Gough, J., The SUPERFAMILY 2.0 database: a significant proteome update and a new webserver. *Nucleic Acids Res* **2019,** *47* (D1), D490-D494.
3. Fox, N. K.; Brenner, S. E.; Chandonia, J. M., SCOPe: Structural Classification of Proteins--extended, integrating SCOP and ASTRAL data and classification of new structures. *Nucleic Acids Res* **2014,** *42* (Database issue), D304-9.
4. Chandonia, J. M.; Guan, L.; Lin, S.; Yu, C.; Fox, N. K.; Brenner, S. E., SCOPe: improvements to the structural classification of proteins - extended database to facilitate variant interpretation and machine learning. *Nucleic Acids Res* **2022,** *50* (D1), D553-D559.

5. Blum, M.; Chang, H. Y.; Chuguransky, S.; Grego, T.; Kandasaamy, S.; Mitchell, A.; Nuka, G.; Paysan-Lafosse, T.; Qureshi, M.; Raj, S.; Richardson, L.; Salazar, G. A.; Williams, L.; Bork, P.; Bridge, A.; Gough, J.; Haft, D. H.; Letunic, I.; Marchler-Bauer, A.; Mi, H.; Natale, D. A.; Necci, M.; Orengo, C. A.; Pandurangan, A. P.; Rivoire, C.; Sigrist, C. J. A.; Sillitoe, I.; Thanki, N.; Thomas, P. D.; Tosatto, S. C. E.; Wu, C. H.; Bateman, A.; Finn, R. D., The InterPro protein families and domains database: 20 years on. *Nucleic Acids Res* **2021,** *49* (D1), D344-D354.

6. Berman, H. M.; Westbrook, J.; Feng, Z.; Gilliland, G.; Bhat, T. N.; Weissig, H.; Shindyalov, I. N.; Bourne, P. E., The Protein Data Bank. *Nucleic Acids Res* **2000,** *28* (1), 235-42.

7. Dürr, S. L.; Bohuszewicz, O.; Berta, D.; Suardiaz, R.; Jambrina, P. G.; Peter, C.; Shao, Y.; Rosta, E., The Role of Conserved Residues in the DEDDh Motif: the Proton-Transfer Mechanism of HIV-1 RNase H. *ACS Catalysis* **2021,** *11* (13), 7915-7927.

8. Lopata, A.; Jambrina, P. G.; Sharma, P. K.; Brooks, B. R.; Toth, J.; Vertessy, B. G.; Rosta, E., Mutations Decouple Proton Transfer from Phosphate Cleavage in the dUTPase Catalytic Reaction. *ACS Catalysis* **2015,** *5* (6), 3225-3237.

9. Nagy, G. N.; Suardiaz, R.; Lopata, A.; Ozohanics, O.; Vekey, K.; Brooks, B. R.; Leveles, I.; Toth, J.; Vertessy, B. G.; Rosta, E., Structural Characterization of Arginine Fingers: Identification of an Arginine Finger for the Pyrophosphatase dUTPases. *J Am Chem Soc* **2016,** *138* (45), 15035-15045.

10. Berta, D.; Gehrke, S.; Nyiri, K.; Vertessy, B. G.; Rosta, E., Mechanism-Based Redesign of GAP to Activate Oncogenic Ras. *J Am Chem Soc* **2023,** *145* (37), 20302-20310.

11. Varga, B.; Barabas, O.; Takacs, E.; Nagy, N.; Nagy, P.; Vertessy, B. G., Active site of mycobacterial dUTPase: structural characteristics and a built-in sensor. *Biochem Biophys Res Commun* **2008,** *373* (1), 8-13.

12. Shao, Y.; Gan, Z.; Epifanovsky, E.; Gilbert, A. T. B.; Wormit, M.; Kussmann, J.; Lange, A. W.; Behn, A.; Deng, J.; Feng, X.; Ghosh, D.; Goldey, M.; Horn, P. R.; Jacobson, L. D.; Kaliman, I.; Khaliullin, R. Z.; Kuś, T.; Landau, A.; Liu, J.; Proynov, E. I.; Rhee, Y. M.; Richard, R. M.; Rohrdanz, M. A.; Steele, R. P.; Sundstrom, E. J.; Woodcock, H. L.; Zimmerman, P. M.; Zuev, D.; Albrecht, B.; Alguire, E.; Austin, B.; Beran, G. J. O.; Bernard, Y. A.; Berquist, E.; Brandhorst, K.; Bravaya, K. B.; Brown, S. T.; Casanova, D.; Chang, C.-M.; Chen, Y.; Chien, S. H.; Closser, K. D.; Crittenden, D. L.; Diedenhofen, M.; DiStasio, R. A.; Do, H.; Dutoi, A. D.; Edgar, R. G.; Fatehi, S.; Fusti-Molnar, L.; Ghysels, A.; Golubeva-Zadorozhnaya, A.; Gomes, J.; Hanson-Heine, M. W. D.; Harbach, P. H. P.; Hauser, A. W.; Hohenstein, E. G.; Holden, Z. C.; Jagau, T.-C.; Ji, H.; Kaduk, B.; Khistyaev, K.; Kim, J.; Kim, J.; King, R. A.; Klunzinger, P.; Kosenkov, D.; Kowalczyk, T.; Krauter, C. M.; Lao, K. U.; Laurent, A. D.; Lawler, K. V.; Levchenko, S. V.; Lin, C. Y.; Liu, F.; Livshits, E.; Lochan, R. C.; Luenser, A.; Manohar, P.; Manzer, S. F.; Mao, S.-P.; Mardirossian, N.; Marenich, A. V.; Maurer, S. A.; Mayhall, N. J.; Neuscamman, E.; Oana, C. M.; Olivares-Amaya, R.; O'Neill, D. P.; Parkhill, J. A.; Perrine, T. M.; Peverati, R.; Prociuk, A.; Rehn, D. R.; Rosta, E.; Russ, N. J.; Sharada, S. M.; Sharma, S.; Small, D. W.; Sodt, A.; Stein, T.; Stück, D.; Su, Y.-C.; Thom, A. J. W.; Tsuchimochi, T.; Vanovschi, V.; Vogt, L.; Vydrov, O.; Wang, T.; Watson, M. A.; Wenzel, J.; White, A.; Williams, C. F.; Yang, J.; Yeganeh, S.; Yost, S. R.; You, Z.-Q.; Zhang, I. Y.; Zhang, X.; Zhao, Y.; Brooks, B. R.; Chan, G. K. L.; Chipman, D. M.; Cramer, C. J.; Goddard, W. A.; Gordon, M. S.; Hehre, W. J.; Klamt, A.; Schaefer, H. F.; Schmidt, M. W.; Sherrill, C. D.; Truhlar, D. G.; Warshel, A.; Xu, X.; Aspuru-Guzik, A.; Baer, R.; Bell, A. T.; Besley, N. A.; Chai, J.-D.; Dreuw, A.; Dunietz, B. D.; Furlani, T. R.; Gwaltney, S. R.; Hsu, C.-P.; Jung, Y.; Kong, J.; Lambrecht, D. S.; Liang, W.;

Ochsenfeld, C.; Rassolov, V. A.; Slipchenko, L. V.; Subotnik, J. E.; Van Voorhis, T.; Herbert, J. M.; Krylov, A. I.; Gill, P. M. W.; Head-Gordon, M., Advances in molecular quantum chemistry contained in the Q-Chem 4 program package. *Molecular Physics* **2014,** *113* (2), 184-215.
13. Brooks, B. R.; Brooks, C. L.; Mackerell, A. D.; Nilsson, L.; Petrella, R. J.; Roux, B.; Won, Y.; Archontis, G.; Bartels, C.; Boresch, S.; Caflisch, A.; Caves, L.; Cui, Q.; Dinner, A. R.; Feig, M.; Fischer, S.; Gao, J.; Hodoscek, M.; Im, W.; Kuczera, K.; Lazaridis, T.; Ma, J.; Ovchinnikov, V.; Paci, E.; Pastor, R. W.; Post, C. B.; Pu, J. Z.; Schaefer, M.; Tidor, B.; Venable, R. M.; Woodcock, H. L.; Wu, X.; Yang, W.; York, D. M.; Karplus, M., CHARMM: The biomolecular simulation program. *Journal of Computational Chemistry* **2009,** *30* (10), 1545-1614.
14. Frisch, M. J.; Trucks, G. W.; Schlegel, H. B.; Scuseria, G. E.; Robb, M. A.; Cheeseman, J. R.; Scalmani, G.; Barone, V.; Petersson, G. A.; Nakatsuji, H.; Li, X.; Caricato, M.; Marenich, A.; Bloino, J.; Janesko, B. G.; Gomperts, R.; Mennucci, B.; Hratchian, H. P.; Ortiz, J. V.; Izmaylov, A. F.; Sonnenberg, J. L.; Williams-Young, D.; Ding, F.; Lipparini, F.; Egidi, F.; Goings, J.; Peng, B.; Petrone, A.; Henderson, T.; Ranasinghe, D.; Zakrzewski, V. G.; Gao, J.; Rega, N.; Zheng, G.; Liang, W.; Hada, M.; Ehara, M.; Toyota, K.; Fukuda, R.; Hasegawa, J.; Ishida, M.; Nakajima, T.; Honda, Y.; Kitao, O.; Nakai, H.; Vreven, T.; Throssell, K.; Jr. Montgomery, J. A.; Peralta, J. E.; Ogliaro, F.; Bearpark, M.; Heyd, J. J.; Brothers, E.; Kudin, K. N.; Staroverov, V. N.; Keith, T.; Kobayashi, R.; Normand, J.; Raghavachari, K.; Rendell, A.; Burant, J. C.; Iyengar, S. S.; Tomasi, J.; Cossi, M.; Millam, J. M.; Klene, M.; Adamo, C.; Cammi, R.; Ochterski, J. W.; Martin, R. L.; Morokuma, K.; Farkas, O.; Foresman, J. B.; Fox, D. J. *Gaussian 09, Revision E*, Gaussian, Inc.: Wallingford CT, 2016.
15. Glendening, E. D.; Reed, A. E.; Carpenter, J. E.; Weinhold, F. *NBO Version 3.1*, Gaussian Inc.: Pittsburgh PA, 2003.
16. Holm, L., Benchmarking fold detection by DaliLite v.5. *Bioinformatics* **2019,** *35* (24), 5326-5327.
17. Fu, L.; Niu, B.; Zhu, Z.; Wu, S.; Li, W., CD-HIT: accelerated for clustering the next-generation sequencing data. *Bioinformatics* **2012,** *28* (23), 3150-2.
18. Katoh, K.; Standley, D. M., MAFFT multiple sequence alignment software version 7: improvements in performance and usability. *Mol Biol Evol* **2013,** *30* (4), 772-80.
19. Price, M. N.; Dehal, P. S.; Arkin, A. P., FastTree 2--approximately maximum-likelihood trees for large alignments. *PLoS One* **2010,** *5* (3), e9490.
20. Sillitoe, I.; Dawson, N.; Lewis, T. E.; Das, S.; Lees, J. G.; Ashford, P.; Tolulope, A.; Scholes, H. M.; Senatorov, I.; Bujan, A.; Ceballos Rodriguez-Conde, F.; Dowling, B.; Thornton, J.; Orengo, C. A., CATH: expanding the horizons of structure-based functional annotations for genome sequences. *Nucleic Acids Res* **2019,** *47* (D1), D280-D284.
21. Sillitoe, I.; Bordin, N.; Dawson, N.; Waman, V. P.; Ashford, P.; Scholes, H. M.; Pang, C. S. M.; Woodridge, L.; Rauer, C.; Sen, N.; Abbasian, M.; Le Cornu, S.; Lam, S. D.; Berka, K.; Varekova, I. H.; Svobodova, R.; Lees, J.; Orengo, C. A., CATH: increased structural coverage of functional space. *Nucleic Acids Res* **2021,** *49* (D1), D266-D273.
22. UniProt, C., UniProt: the Universal Protein Knowledgebase in 2023. *Nucleic Acids Res* **2023,** *51* (D1), D523-D531.
23. UniProt, C., UniProt: the Universal Protein Knowledgebase in 2025. *Nucleic Acids Res* **2025,** *53* (D1), D609-D617.
24. Kruger, M.; Linke, W. A., The giant protein titin: a regulatory node that integrates myocyte signaling pathways. *J Biol Chem* **2011,** *286* (12), 9905-12.

25. Paysan-Lafosse, T.; Blum, M.; Chuguransky, S.; Grego, T.; Pinto, B. L.; Salazar, G. A.; Bileschi, M. L.; Bork, P.; Bridge, A.; Colwell, L.; Gough, J.; Haft, D. H.; Letunic, I.; Marchler-Bauer, A.; Mi, H.; Natale, D. A.; Orengo, C. A.; Pandurangan, A. P.; Rivoire, C.; Sigrist, C. J. A.; Sillitoe, I.; Thanki, N.; Thomas, P. D.; Tosatto, S. C. E.; Wu, C. H.; Bateman, A., InterPro in 2022. *Nucleic Acids Res* **2023,** *51* (D1), D418-D427.

26. Guigo, R., Genome annotation: From human genetics to biodiversity genomics. *Cell Genom* **2023,** *3* (8), 100375.

27. Marahiel, M. A.; Stachelhaus, T.; Mootz, H. D., Modular Peptide Synthetases Involved in Nonribosomal Peptide Synthesis. *Chem Rev* **1997,** *97* (7), 2651-2674.

28. Fischbach, M. A.; Walsh, C. T., Assembly-line enzymology for polyketide and nonribosomal Peptide antibiotics: logic, machinery, and mechanisms. *Chem Rev* **2006,** *106* (8), 3468-96.

29. Nakamura, T.; Zhao, Y.; Yamagata, Y.; Hua, Y. J.; Yang, W., Watching DNA polymerase eta make a phosphodiester bond. *Nature* **2012,** *487* (7406), 196-201.

30. Yang, W.; Weng, P. J.; Gao, Y., A new paradigm of DNA synthesis: three-metal-ion catalysis. *Cell Biosci* **2016,** *6* (1), 51.

31. Murayama, Y.; Ehara, H.; Aoki, M.; Goto, M.; Yokoyama, T.; Sekine, S. I., Structural basis of the transcription termination factor Rho engagement with transcribing RNA polymerase from Thermus thermophilus. *Sci Adv* **2023,** *9* (6), eade7093.

32. Serrano, A.; Sebastian, M.; Arilla-Luna, S.; Baquedano, S.; Herguedas, B.; Velazquez-Campoy, A.; Martinez-Julvez, M.; Medina, M., The trimer interface in the quaternary structure of the bifunctional prokaryotic FAD synthetase from Corynebacterium ammoniagenes. *Sci Rep* **2017,** *7* (1), 404.

33. Gallagher, D. T.; Kim, S. K.; Robinson, H.; Reddy, P. T., Active-site structure of class IV adenylyl cyclase and transphyletic mechanism. *J Mol Biol* **2011,** *405* (3), 787-803.

34. Vogt, M. S.; Ngouoko Nguepbeu, R. R.; Mohr, M. K. F.; Albers, S. V.; Essen, L. O.; Banerjee, A., The archaeal triphosphate tunnel metalloenzyme SaTTM defines structural determinants for the diverse activities in the CYTH protein family. *J Biol Chem* **2021,** *297* (1), 100820.

35. Bardwell, L., Pseudokinases: Flipping the ATP for AMPylation. *Curr Biol* **2019,** *29* (1), R23-R25.

36. Sreelatha, A.; Yee, S. S.; Lopez, V. A.; Park, B. C.; Kinch, L. N.; Pilch, S.; Servage, K. A.; Zhang, J.; Jiou, J.; Karasiewicz-Urbanska, M.; Lobocka, M.; Grishin, N. V.; Orth, K.; Kucharczyk, R.; Pawlowski, K.; Tomchick, D. R.; Tagliabracci, V. S., Protein AMPylation by an Evolutionarily Conserved Pseudokinase. *Cell* **2018,** *175* (3), 809-821 e19.

37. Elnatan, D.; Betegon, M.; Liu, Y.; Ramelot, T.; Kennedy, M. A.; Agard, D. A., Symmetry broken and rebroken during the ATP hydrolysis cycle of the mitochondrial Hsp90 TRAP1. *Elife* **2017,** *6.*

38. Brunle, S.; Eisinger, M. L.; Poppe, J.; Mills, D. J.; Langer, J. D.; Vonck, J.; Ermler, U., Molybdate pumping into the molybdenum storage protein via an ATP-powered piercing mechanism. *Proc Natl Acad Sci U S A* **2019,** *116* (52), 26497-26504.

39. Marshall, M.; Cohen, P. P., A kinetic study of the mechanism of crystalline carbamate kinase. *Journal of Biological Chemistry* **1966,** *241* (17), 4197-4208.

40. Pieslinger, A. M.; Hoepflinger, M. C.; Tenhaken, R., Cloning of Glucuronokinase from Arabidopsis thaliana, the Last Missing Enzyme of the myo-Inositol Oxygenase Pathway to Nucleotide Sugars. *J. Biol. Chem.* **2010,** *285* (5), 2902-2910.

41. Biondi, R. M.; Baehler, P. J.; Reymond, C. D.; Bhatt, M., Role of Mg2+ in the autophosphorylation reaction of nucleoside diphosphate kinase. *Arch. Biochem. Biophys.* **1998,** *353* (1), 85-92.
42. Walker, T. E.; Shirzadeh, M.; Sun, H. M.; McCabe, J. W.; Roth, A.; Moghadamchargari, Z.; Clemmer, D. E.; Laganowsky, A.; Rye, H.; Russell, D. H., Temperature Regulates Stability, Ligand Binding (Mg2+ and ATP), and Stoichiometry of GroEL-GroES Complexes. *J. Am. Chem. Soc.* **2022,** *144* (6), 2667-2678.
43. Biteau, B.; Labarre, J.; Toledano, M. B., ATP-dependent reduction of cysteine-sulphinic acid by Saccharomyces cerevisiae sulphiredoxin. *Nature* **2003,** *423*, 875-878.
44. Willett, E.; Jiang, V.; Koder, R. L.; Banta, S., NAD+ Kinase Enzymes Are Reversible, and NAD+ Product Inhibition Is Responsible for the Observed Irreversibility of the Human Enzyme. *Biochemistry* **2022,** *61* (17), 1862-1873.
45. Merrill, A. H. J.; Froehlich, J. A.; McCormick, D. B., Affinity chromatographic purification and properties of flavokinase (ATP:riboflavin 5'-phosphotransferase) from rat liver. *J. Biol. Chem.* **1980,** *255* (4), 1335-1338.
46. Veres, Z.; Kim, I. Y.; Scholz, T. D.; Stadtman, T. C., Selenophosphate synthetase. Enzyme properties and catalytic reaction. *Journal of Biological Chemistry* **1994,** *269* (14), 10597-10603.
47. Gallagher, D. T.; Kim, S.-K.; Robinson, H.; Reddy, P. T., Active-Site Structure of Class IV Adenylyl Cyclase and Transphyletic Mechanism. *Journal of Molecular Biology* **2011,** *405* (3), 787-803.
48. Bettendorff, L.; Wins, P., Thiamine triphosphatase and the CYTH superfamily of proteins. *FEBS J* **2013,** *280* (24), 6443-55.
49. Li, H.; Graupner, M.; Xu, H.; White, R. H., CofE Catalyzes the Addition of Two Glutamates to F420-0 in F420 Coenzyme Biosynthesis in Methanococcus jannaschii. *Biochemistry* **2003,** *42* (32), 9771-9778.
50. Vorobiev, S.; Strokopytov, B.; Drubin, D. G.; Frieden, C.; Ono, S.; Condeelis, J.; Rubenstein, P. A.; Almo, S. C., The structure of nonvertebrate actin: implications for the ATP hydrolytic mechanism. *Proc Natl Acad Sci U S A* **2003,** *100* (10), 5760-5.
51. Frieden, C., Polymerization of actin: mechanism of the Mg2+-induced process at pH 8 and 20 degrees C. *Proc. Natl. Acad. Sci. USA* **1983,** *80* (21), 6513-6517.
52. Tholey, G.; Bloch, S.; Ledig, M.; Mandel, P.; Wedler, F. C., Chick brain glutamine synthetase and Mn2+-Mg2+ interactions. *Neurochem. Res.* **1987,** *12* (12), 1041-1047.
53. Hauf, W.; Schmid, K.; Gerhardt, E. C. M.; Huergo, L. F.; Forchhammer, K., Interaction of the Nitrogen Regulatory Protein GlnB (PII) with Biotin Carboxyl Carrier Protein (BCCP) Controls Acetyl-CoA Levels in the Cyanobacterium Synechocystis sp. PCC 6803. *Front. Microbiol.* **2016,** *7*, 1700.
54. Jonsson, A.; Nordlund, S., In vitro studies of the uridylylation of the three PII protein paralogs from Rhodospirillum rubrum: the transferase activity of R. rubrum GlnD is regulated by alpha-ketoglutarate and divalent cations but not by glutamine. *J Bacteriol* **2007,** *189* (9), 3471-8.
55. Rojas-Pirela, M.; Andrade-Alviarez, D.; Rojas, V.; Kemmerling, U.; Caceres, A. J.; Michels, P. A.; Concepcion, J. L.; Quinones, W., Phosphoglycerate kinase: structural aspects and functions, with special emphasis on the enzyme from Kinetoplastea. *Open Biol* **2020,** *10* (11), 200302.
56. Chastain, C. J.; Failing, C. J.; Manandhar, L.; Zimmerman, M. A.; Lakner, M. M.; Nguyen, T. H. T., Functional evolution of C4 pyruvate, orthophosphate dikinase. *Journal of Experimental Botany* **2011,** *62* (9), 3083-3091.

57. Gupta, R. K.; Oesterling, R. M., Dual divalent cation requirement for activation of pyruvate kinase; essential roles of both enzyme- and nucleotide-bound metal ions. *Biochemistry* **1976,** *15* (13), 2881-2887.
58. McNae, I. W.; Martinez-Oyanedel, J.; Keillor, J. W.; Michels, P. A. M.; Fothergill-Gilmore, L. A.; Walkinshaw, M. D., The Crystal Structure of ATP-bound Phosphofructokinase from Trypanosoma brucei Reveals Conformational Transitions Different from those of Other Phosphofructokinases. *Journal of Molecular Biology* **2009,** *385* (5), 1519-1533.
59. Etiemble, J.; Simeon, J.; Picat, C.; Boivin, P., Influence of free Mg2+ on the kinetics of human erythrocyte phosphofructokinase. *Biochimie* **1981,** *63* (1), 61-5.
60. Farnsworth, C. L.; Feig, L. A., Dominant inhibitory mutations in the Mg(2+)-binding site of RasH prevent its activation by GTP. *Mol Cell Biol* **1991,** *11* (10), 4822-9.
61. Keum, Y. S.; Jeong, Y. J., Development of chemical inhibitors of the SARS coronavirus: viral helicase as a potential target. *Biochem Pharmacol* **2012,** *84* (10), 1351-8.
62. Nogales, E.; Downing, K. H.; Amos, L. A.; Lowe, J., Tubulin and FtsZ form a distinct family of GTPases. *Nat Struct Biol* **1998,** *5* (6), 451-8.
63. Gaskin, F., In vitro microtubule assembly regulation by divalent cations and nucleotides. *Biochemistry* **1981,** *20* (5), 1318-22.
64. Grover, S.; Hamel, E., The magnesium-GTP interaction in microtubule assembly. *Eur J Biochem* **1994,** *222* (1), 163-72.
65. Holyoak, T.; Sullivan, S. M.; Nowak, T., Structural insights into the mechanism of PEPCK catalysis. *Biochemistry* **2006,** *45* (27), 8254-63.
66. Lee, M. H.; Hebda, C. A.; Nowak, T., The role of cations in avian liver phosphoenolpyruvate carboxykinase catalysis. Activation and regulation. *Journal of Biological Chemistry* **1981,** *256* (24), 12793-12801.
67. Apell, H. J.; Hitzler, T.; Schreiber, G., Modulation of the Na,K-ATPase by Magnesium Ions. *Biochemistry* **2017,** *56* (7), 1005-1016.
68. Boneca, I. G.; Šink, R.; Kotnik, M.; Zega, A.; Barreteau, H.; Gobec, S.; Blanot, D.; Dessen, A.; Contreras-Martel, C., Crystallographic Study of Peptidoglycan Biosynthesis Enzyme MurD: Domain Movement Revisited. *Plos One* **2016,** *11* (3).
69. Bertrand, J. A.; Auger, G.; Martin, L.; Fanchon, E.; Blanot, D.; Le Beller, D.; van Heijenoort, J.; Dideberg, O., Determination of the MurD mechanism through crystallographic analysis of enzyme complexes. *Journal of Molecular Biology* **1999,** *289* (3), 579-590.
70. González, B.; Baños-Sanz, J. I.; Villate, M.; Brearley, C. A.; Sanz-Aparicio, J., Inositol 1,3,4,5,6-pentakisphosphate 2-kinase is a distant IPK member with a singular inositide binding site for axial 2-OH recognition. *Proc. Natl. Acad. Sci. U.S.A.* **2010,** *107* (21), 9608-9613.
71. Basu, M. K.; Selengut, J. D.; Haft, D. H., ProPhylo: partial phylogenetic profiling to guide protein family construction and assignment of biological process. *BMC Bioinformatics* **2011,** *12* (1).
72. Nayak, D. D.; Mahanta, N.; Mitchell, D. A.; Metcalf, W. W., Post-translational thioamidation of methyl-coenzyme M reductase, a key enzyme in methanogenic and methanotrophic Archaea. *eLife* **2017,** *6*.
73. Mahanta, N.; Liu, A.; Dong, S.; Nair, S. K.; Mitchell, D. A., Enzymatic reconstitution of ribosomal peptide backbone thioamidation. *Proceedings of the National Academy of Sciences* **2018,** *115* (12), 3030-3035.
74. Dunbar, K. L.; Melby, J. O.; Mitchell, D. A., YcaO domains use ATP to activate amide backbones during peptide cyclodehydrations. *Nat Chem Biol* **2012,** *8* (6), 569-75.

75. Xiang, Y.; Leiman, P. G.; Li, L.; Grimes, S.; Anderson, D. L.; Rossmann, M. G., Crystallographic Insights into the Autocatalytic Assembly Mechanism of a Bacteriophage Tail Spike. *Molecular Cell* **2009,** *34* (3), 375-386.
76. Kim, S. H.; Witte, C. P.; Rhee, S., Structural basis for the substrate specificity and catalytic features of pseudouridine kinase from Arabidopsis thaliana. *Nucleic Acids Res* **2021,** *49* (1), 491-503.
77. Shumilin, I. A.; Cymborowski, M.; Chertihin, O.; Jha, K. N.; Herr, J. C.; Lesley, S. A.; Joachimiak, A.; Minor, W., Identification of unknown protein function using metabolite cocktail screening. *Structure* **2012,** *20* (10), 1715-25.
78. Kang, P. A.; Oh, J.; Lee, H.; Witte, C. P.; Rhee, S., Crystal structure and mutational analyses of ribokinase from Arabidopsis thaliana. *J Struct Biol* **2019,** *206* (1), 110-118.
79. Sigrell, J. A.; Cameron, A. D.; Jones, T. A.; Mowbray, S. L., Structure of Escherichia coli ribokinase in complex with ribose and dinucleotide determined to 1.8 A resolution: insights into a new family of kinase structures. *Structure* **1998,** *6* (2), 183-93.
80. Siebold, C.; Arnold, I.; Garcia-Alles, L. F.; Baumann, U.; Erni, B., Crystal structure of the Citrobacter freundii dihydroxyacetone kinase reveals an eight-stranded alpha-helical barrel ATP-binding domain. *J Biol Chem* **2003,** *278* (48), 48236-44.
81. Bachler, C.; Schneider, P.; Bahler, P.; Lustig, A.; Erni, B., Escherichia coli dihydroxyacetone kinase controls gene expression by binding to transcription factor DhaR. *EMBO J* **2005,** *24* (2), 283-93.
82. Yong, S. C.; Roversi, P.; Lillington, J.; Rodriguez, F.; Krehenbrink, M.; Zeldin, O. B.; Garman, E. F.; Lea, S. M.; Berks, B. C., A complex iron-calcium cofactor catalyzing phosphotransfer chemistry. *Science* **2014,** *345* (6201), 1170-1173.
83. AN, B., Catalytic mechanisms for phosphotriesterases. *Biochim. Biophys. Acta* **2013,** *1834* (1), 443-453.
84. Knape, M. J.; Ahuja, L. G.; Bertinetti, D.; Burghardt, N. C.; Zimmermann, B.; Taylor, S. S.; Herberg, F. W., Divalent Metal Ions Mg(2)(+) and Ca(2)(+) Have Distinct Effects on Protein Kinase A Activity and Regulation. *ACS Chem Biol* **2015,** *10* (10), 2303-15.
85. H, G., Purification and characterization of glutathione synthetase from Escherichia coli B. *J. Biochem.* **1983,** *94* (5), 1347-1352.
86. SW, N., Magnesium-assisted catalysis by a D-ribulose-5-phosphate 3-epimerase homologue. *Biochemistry* **2004,** *43* (21), 6784-6794.
87. Walsh, J. P.; Bell, R. M., sn-1,2-Diacylglycerol kinase of Escherichia coli. Mixed micellar analysis of the phospholipid cofactor requirement and divalent cation dependence. *Journal of Biological Chemistry* **1986,** *261* (14), 6239-6247.
88. Huang, L. Y.; Wang, S. C.; Cheng, T. R.; Wong, C. H., Undecaprenyl Phosphate Phosphatase Activity of Undecaprenol Kinase Regulates the Lipid Pool in Gram-Positive Bacteria. *Biochemistry* **2017,** *56* (40), 5417-5427.
89. Keppetipola, N.; Shuman, S., A Phosphate-binding Histidine of Binuclear Metallophosphodiesterase Enzymes Is a Determinant of 2′,3′-Cyclic Nucleotide Phosphodiesterase Activity. *Journal of Biological Chemistry* **2008,** *283* (45), 30942-30949.
90. Li, H. C.; Chan, W. W., Activation of brain calcineurin towards proteins containing Thr(P) and Ser(P) by Ca2+, calmodulin, Mg2+ and transition metal ions. *Eur J Biochem* **1984,** *144* (3), 447-52.

91. Hvorecny, K. L.; Hargett, K.; Quispe, J. D.; Kollman, J. M., Human PRPS1 filaments stabilize allosteric sites to regulate activity. *Nature Structural & Molecular Biology* **2023,** *30* (3), 391-402.
92. Bhatia, M. B., The role of divalent magnesium in activating the reaction catalyzed by orotate phosphoribosyltransferase. *Archives of Biochemistry and Biophysics* **1993**.
93. Lu, L. D., Mycobacterial MazG is a novel NTP pyrophosphohydrolase involved in oxidative stress response. *Journal of Biological Chemistry* **2010**.
94. Schweikhard, E. S.; Kuhlmann, S. I.; Kunte, H.-J.; Grammann, K.; Ziegler, C. M., Structure and Function of the Universal Stress Protein TeaD and Its Role in Regulating the Ectoine Transporter TeaABC of Halomonas elongata DSM 2581T. *Biochemistry* **2010,** *49* (10), 2194-2204.
95. Bangera, M.; Panigrahi, R.; Sagurthi, S. R.; Savithri, H. S.; Murthy, M. R. N., Structural and functional analysis of two universal stress proteins YdaA and YnaF from Salmonella typhimurium: possible roles in microbial stress tolerance. *Journal of Structural Biology* **2015,** *189* (3), 238-250.
96. Tan, Y. W., Direct Mg2+ binding activates adenylate kinase from Escherichia coli. *Journal of Biological Chemistry* **2009**.
97. Turnquist, R. L., Uridine diphosphate glucose pyrophosphorylase. Crystallization and properties of the enzyme from rabbit liver and species comparisons. *Journal of Biological Chemistry* **1974**.
98. Tokgöz, Z., Pleiotropic effects of ATP.Mg2+ binding in the catalytic cycle of ubiquitin-activating enzyme. *Journal of Biological Chemistry* **2006**.
99. Kishimoto, A.; Kita, A.; Ishibashi, T.; Tomita, H.; Yokooji, Y.; Imanaka, T.; Atomi, H.; Miki, K., Crystal structure of phosphopantothenate synthetase from Thermococcus kodakarensis. *Proteins: Structure, Function, and Bioinformatics* **2014,** *82* (9), 1924-1936.
100. Yokooji, Y.; Tomita, H.; Atomi, H.; Imanaka, T., Pantoate Kinase and Phosphopantothenate Synthetase, Two Novel Enzymes Necessary for CoA Biosynthesis in the Archaea. *Journal of Biological Chemistry* **2009,** *284* (41), 28137-28145.
101. Zheng, R., Steady-state and pre-steady-state kinetic analysis of Mycobacterium tuberculosis pantothenate synthetase. *Biochemistry* **2001**.
102. Gleghorn, M. L.; Davydova, E. K.; Rothman-Denes, L. B.; Murakami, K. S., Structural Basis for DNA-Hairpin Promoter Recognition by the Bacteriophage N4 Virion RNA Polymerase. *Molecular Cell* **2008,** *32* (5), 707-717.
103. Lykke-Andersen, J., The C-terminal carboxy group of T7 RNA polymerase ensures efficient magnesium ion-dependent catalysis. *Nucleic Acids Research* **1998**.
104. Shi, G.; Gong, Y.; Savchenko, A.; Zeikus, J. G.; Xiao, B.; Ji, X.; Yan, H., Dissecting the nucleotide binding properties of Escherichia coli 6-hydroxymethyl-7,8-dihydropterin pyrophosphokinase with fluorescent 3'(2)'-o-anthraniloyladenosine 5'-triphosphate. *Biochim Biophys Acta* **2000,** *1478* (2), 289-99.
105. Ador, L.; Jaeger, S.; Geslain, R.; Martin, F.; Cavarelli, J.; Eriani, G., Mutation and evolution of the magnesium-binding site of a class II aminoacyl-tRNA synthetase. *Biochemistry* **2004,** *43* (22), 7028-37.
106. Gabelli, S. B.; Bianchet, M. A.; Ohnishi, Y.; Ichikawa, Y.; Bessman, M. J.; Amzel, L. M., Mechanism of the Escherichia coli ADP-ribose pyrophosphatase, a Nudix hydrolase. *Biochemistry* **2002,** *41* (30), 9279-85.

107. Zaychikov, E., Mapping of catalytic residues in the RNA polymerase active center. *Science* **1996**.
108. Jacewicz, A.; Dantuluri, S.; Shuman, S., Structures of RNA ligase RtcB in complexes with divalent cations and GTP. *Rna* **2022,** *28* (11), 1509-1518.
109. Mydy, L. S.; Bailey, D. C.; Patel, K. D.; Rice, M. R.; Gulick, A. M., The Siderophore Synthetase IucA of the Aerobactin Biosynthetic Pathway Uses an Ordered Mechanism. *Biochemistry* **2020,** *59* (23), 2143-2153.
110. Johansson, E.; Fanø, M.; Bynck, J. H.; Neuhard, J.; Larsen, S.; Sigurskjold, B. W.; Christensen, U.; Willemoës, M., Structures of dCTP Deaminase from Escherichia coli with Bound Substrate and Product. *Journal of Biological Chemistry* **2005,** *280* (4), 3051-3059.
111. Mustafi, D.; Bekesi, A.; Vertessy, B. G.; Makinen, M. W., Catalytic and structural role of the metal ion in dUTP pyrophosphatase. *Proc Natl Acad Sci U S A* **2003,** *100* (10), 5670-5.
112. Shuman, S.; Hurwitz, J., Mechanism of mRNA capping by vaccinia virus guanylyltransferase: characterization of an enzyme--guanylate intermediate. *Proc Natl Acad Sci U S A* **1981,** *78* (1), 187-91.
113. Lin, S.; McLennan, A. G.; Ying, K.; Wang, Z.; Gu, S.; Jin, H.; Wu, C.; Liu, W.; Yuan, Y.; Tang, R.; Xie, Y.; Mao, Y., Cloning, expression, and characterization of a human inosine triphosphate pyrophosphatase encoded by the itpa gene. *J Biol Chem* **2001,** *276* (22), 18695-701.
114. Mostert, K. J.; Sharma, N.; van der Zwaag, M.; Staats, R.; Koekemoer, L.; Anand, R.; Sibon, O. C. M.; Strauss, E., The Coenzyme A Level Modulator Hopantenate (HoPan) Inhibits Phosphopantotenoylcysteine Synthetase Activity. *ACS Chemical Biology* **2021,** *16* (11), 2401-2414.
115. Yao, J.; Patrone, J. D.; Dotson, G. D., Characterization and kinetics of phosphopantothenoylcysteine synthetase from Enterococcus faecalis. *Biochemistry* **2009,** *48* (12), 2799-806.
116. Chakravarty, A. K.; Smith, P.; Shuman, S., Structures of RNA 3′-phosphate cyclase bound to ATP reveal the mechanism of nucleotidyl transfer and metal-assisted catalysis. *Proceedings of the National Academy of Sciences* **2011,** *108* (52), 21034-21039.
117. Yang, B.; Yao, H.; Li, D.; Liu, Z., The phosphatidylglycerol phosphate synthase PgsA utilizes a trifurcated amphipathic cavity for catalysis at the membrane-cytosol interface. *Curr Res Struct Biol* **2021,** *3*, 312-323.
118. Chakravarty, A. K.; Shuman, S., RNA 3'-phosphate cyclase (RtcA) catalyzes ligase-like adenylylation of DNA and RNA 5'-monophosphate ends. *J Biol Chem* **2011,** *286* (6), 4117-22.
119. Harris, K. A.; Jones, V.; Bilbille, Y.; Swairjo, M. A.; Agris, P. F., YrdC exhibits properties expected of a subunit for a tRNA threonylcarbamoyl transferase. *RNA* **2011,** *17* (9), 1678-87.
120. Witte, G.; Hartung, S.; Buttner, K.; Hopfner, K. P., Structural biochemistry of a bacterial checkpoint protein reveals diadenylate cyclase activity regulated by DNA recombination intermediates. *Mol Cell* **2008,** *30* (2), 167-78.
121. Müller, M.; Deimling, T.; Hopfner, K.-P.; Witte, G., Structural analysis of the diadenylate cyclase reaction of DNA-integrity scanning protein A (DisA) and its inhibition by 3′-dATP. *Biochemical Journal* **2015,** *469* (3), 367-374.
122. Bai, Y.; Yang, J.; Zhou, X.; Ding, X.; Eisele, L. E.; Bai, G., Mycobacterium tuberculosis Rv3586 (DacA) is a diadenylate cyclase that converts ATP or ADP into c-di-AMP. *PLoS One* **2012,** *7* (4), e35206.

123. Li, C.; Li, H.; Zhou, S.; Sun, E.; Yoshizawa, J.; Poulos, T. L.; Gershon, P. D., Polymerase Translocation with Respect to Single-Stranded Nucleic Acid: Looping or Wrapping of Primer around a Poly(A) Polymerase. *Structure* **2009,** *17* (5), 680-689.
124. Balbo, P. B.; Meinke, G.; Bohm, A., Kinetic studies of yeast polyA polymerase indicate an induced fit mechanism for nucleotide specificity. *Biochemistry* **2005,** *44* (21), 7777-86.
125. Berta, D.; Buigues, P. J.; Badaoui, M.; Rosta, E., Cations in motion: QM/MM studies of the dynamic and electrostatic roles of H(+) and Mg(2+) ions in enzyme reactions. *Curr Opin Struct Biol* **2020,** *61*, 198-206.
126. Bermek, O.; Grindley, N. D.; Joyce, C. M., Distinct roles of the active-site Mg2+ ligands, Asp882 and Asp705, of DNA polymerase I (Klenow fragment) during the prechemistry conformational transitions. *J Biol Chem* **2011,** *286* (5), 3755-66.
127. Deibel, M. R.; Coleman, M. S., Biochemical properties of purified human terminal deoxynucleotidyltransferase. *Journal of Biological Chemistry* **1980,** *255* (9), 4206-4212.
128. Steegborn, C.; Litvin, T. N.; Levin, L. R.; Buck, J.; Wu, H., Bicarbonate activation of adenylyl cyclase via promotion of catalytic active site closure and metal recruitment. *Nat Struct Mol Biol* **2005,** *12* (1), 32-7.
129. Zimmermann, G., Mutations uncover a role for two magnesium ions in the catalytic mechanism of adenylyl cyclase. *Journal of Biological Chemistry* **1998**.
130. Tokarsky, E. J.; Wallenmeyer, P. C.; Phi, K. K.; Suo, Z., Significant impact of divalent metal ions on the fidelity, sugar selectivity, and drug incorporation efficiency of human PrimPol. *DNA Repair* **2017,** *49*, 51-59.
131. Calvo, P. A.; Sastre-Moreno, G.; Perpiñá, C.; Guerra, S.; Martínez-Jiménez, M. I.; Blanco, L., The invariant glutamate of human PrimPol DxE motif is critical for its Mn2+-dependent distinctive activities. *DNA Repair* **2019,** *77*, 65-75.
132. Gonzalez, S., Distinct regions of influenza virus PB1 polymerase subunit recognize vRNA and cRNA templates. *The EMBO Journal* **1999,** *18* (13), 3767-3775.
133. Crépin, T.; Dias, A.; Palencia, A. s.; Swale, C.; Cusack, S.; Ruigrok, R. W. H., Mutational and Metal Binding Analysis of the Endonuclease Domain of the Influenza Virus Polymerase PA Subunit. *Journal of Virology* **2010,** *84* (18), 9096-9104.
134. Iyer, L. M.; Koonin, E. V.; Aravind, L., *BMC Structural Biology* **2003,** *3* (1).
135. Arnold, J. J., Poliovirus RNA-dependent RNA polymerase (3Dpol): pre-steady-state kinetic analysis of ribonucleotide incorporation in the presence of Mg2+. *Biochemistry* **2004**.
136. Luong, P.; Kinch, L. N.; Brautigam, C. A.; Grishin, N. V.; Tomchick, D. R.; Orth, K., Kinetic and Structural Insights into the Mechanism of AMPylation by VopS Fic Domain. *Journal of Biological Chemistry* **2010,** *285* (26), 20155-20163.
137. Ren, S.; Caforio, A.; Yang, Q.; Sun, B.; Yu, F.; Zhu, X.; Wang, J.; Dou, C.; Fu, Q.; Huang, N.; Sun, Q.; Nie, C.; Qi, S.; Gong, X.; He, J.; Wei, Y.; Driessen, A. J. M.; Cheng, W., Structural and mechanistic insights into the biosynthesis of CDP-archaeol in membranes. *Cell Research* **2017,** *27* (11), 1378-1391.
138. Karuppiah, V.; Thistlethwaite, A.; Dajani, R.; Warwicker, J.; Derrick, J. P., Structure and Mechanism of the Bifunctional CinA Enzyme from Thermus thermophilus. *Journal of Biological Chemistry* **2014,** *289* (48), 33187-33197.
139. Nichols, J.; Rajagopalan, K. V., Escherichia coli MoeA and MogA. *Journal of Biological Chemistry* **2002,** *277* (28), 24995-25000.
140. Collier, R. J.; Young, J. A. T., Anthrax Toxin. *Annual Review of Cell and Developmental Biology* **2003,** *19* (1), 45-70.

141. Guo, Q.; Shen, Y.; Zhukovskaya, N. L.; Florián, J.; Tang, W.-J., Structural and Kinetic Analyses of the Interaction of Anthrax Adenylyl Cyclase Toxin with Reaction Products cAMP and Pyrophosphate. *Journal of Biological Chemistry* **2004,** *279* (28), 29427-29435.
142. Shen, Y., Physiological calcium concentrations regulate calmodulin binding and catalysis of adenylyl cyclase exotoxins. *The EMBO Journal* **2002**.
143. Godson, G. N.; Schoenich, J.; Sun, W.; Mustaev, A. A., Identification of the Magnesium Ion Binding Site in the Catalytic Center of Escherichia coli Primase by Iron Cleavage. *Biochemistry* **1999,** *39* (2), 332-339.
144. Webster, L. T., Studies of the Acetyl Coenzyme A Synthetase Reaction. *Journal of Biological Chemistry* **1965,** *240* (11), 4164-4169.
145. Webster, L. T., Studies of the acetyl coenzyme A synthetase reaction. III. Evidence of a double requirement for divalent cations. *J Biol Chem* **1967**.
146. Gorelik, A.; Randriamihaja, A.; Illes, K.; Nagar, B., Structural basis for nucleotide recognition by the ectoenzyme CD203c. *The FEBS Journal* **2018,** *285* (13), 2481-2494.
147. Gorelik, A.; Randriamihaja, A.; Illes, K.; Nagar, B., Structural basis for nucleotide recognition by the ectoenzyme CD203c. *FEBS J* **2018,** *285* (13), 2481-2494.
148. Gijsbers, R.; Ceulemans, H.; Stalmans, W.; Bollen, M., Structural and Catalytic Similarities between Nucleotide Pyrophosphatases/Phosphodiesterases and Alkaline Phosphatases. *Journal of Biological Chemistry* **2001,** *276* (2), 1361-1368.
149. Zalatan, J. G.; Fenn, T. D.; Brunger, A. T.; Herschlag, D., Structural and Functional Comparisons of Nucleotide Pyrophosphatase/Phosphodiesterase and Alkaline Phosphatase: Implications for Mechanism and Evolution. *Biochemistry* **2006,** *45* (32), 9788-9803.
150. Hausmann, J.; Keune, W.-J.; Hipgrave Ederveen, A. L.; van Zeijl, L.; Joosten, R. P.; Perrakis, A., Structural snapshots of the catalytic cycle of the phosphodiesterase Autotaxin. *Journal of Structural Biology* **2016,** *195* (2), 199-206.
151. Stec, B., A revised mechanism for the alkaline phosphatase reaction involving three metal ions. *Journal of Molecular Biology* **2000**.
152. Nosaka, K., Steady-state kinetics and mutational studies of recombinant human thiamin pyrophosphokinase. *Journal of Nutritional Science and Vitaminology* **2003**.
153. Deng, J.; Schnaufer, A.; Salavati, R.; Stuart, K. D.; Hol, W. G. J., High Resolution Crystal Structure of a Key Editosome Enzyme from Trypanosoma brucei: RNA Editing Ligase 1. *Journal of Molecular Biology* **2004,** *343* (3), 601-613.
154. Cherepanov, A. V.; de Vries, S., Kinetic Mechanism of the Mg2+-dependent Nucleotidyl Transfer Catalyzed by T4 DNA and RNA Ligases. *Journal of Biological Chemistry* **2002,** *277* (3), 1695-1704.
155. Shuman, S.; Goldgur, Y.; Unciuleac, M.-C., Caveat mutator: alanine substitutions for conserved amino acids in RNA ligase elicit unexpected rearrangements of the active site for lysine adenylylation. *Nucleic Acids Research* **2020,** *48* (10), 5603-5615.
156. Taylor, M. R.; Conrad, J. A.; Wahl, D.; O'Brien, P. J., Kinetic mechanism of human DNA ligase I reveals magnesium-dependent changes in the rate-limiting step that compromise ligation efficiency. *J Biol Chem* **2011,** *286* (26), 23054-62.
157. Ren, J., GTP cyclohydrolase II structure and mechanism. *Journal of Biological Chemistry* **2005**.
158. Estrada, P.; Manandhar, M.; Dong, S. H.; Deveryshetty, J.; Agarwal, V.; Cronan, J. E.; Nair, S. K., The pimeloyl-CoA synthetase BioW defines a new fold for adenylate-forming enzymes. *Nat Chem Biol* **2017,** *13* (6), 668-674.

159. Ploux, O.; Soularue, P.; Marquet, A.; Gloeckler, R.; Lemoine, Y., Investigation of the first step of biotin biosynthesis in Bacillus sphaericus. Purification and characterization of the pimeloyl-CoA synthase, and uptake of pimelate. *Biochem J* **1992,** *287 (Pt 3)* (Pt 3), 685-90.
160. Schwarzenbacher, R.; McMullan, D.; Krishna, S. S.; Xu, Q.; Miller, M. D.; Canaves, J. M.; Elsliger, M. A.; Floyd, R.; Grzechnik, S. K.; Jaroszewski, L.; Klock, H. E.; Koesema, E.; Kovarik, J. S.; Kreusch, A.; Kuhn, P.; McPhillips, T. M.; Morse, A. T.; Quijano, K.; Spraggon, G.; Stevens, R. C.; van den Bedem, H.; Wolf, G.; Hodgson, K. O.; Wooley, J.; Deacon, A. M.; Godzik, A.; Lesley, S. A.; Wilson, I. A., Crystal structure of a glycerate kinase (TM1585) from Thermotoga maritima at 2.70 Å resolution reveals a new fold. *Proteins: Structure, Function, and Bioinformatics* **2006,** *65* (1), 243-248.
161. Kehrer, D.; Ahmed, H.; Brinkmann, H.; Siebers, B., Glycerate kinase of the hyperthermophilic archaeon Thermoproteus tenax: new insights into the phylogenetic distribution and physiological role of members of the three different glycerate kinase classes. *BMC Genomics* **2007,** *8* (1).
162. McCoy, J. G.; Arabshahi, A.; Bitto, E.; Bingman, C. A.; Ruzicka, F. J.; Frey, P. A.; Phillips, G. N., Structure and Mechanism of an ADP-Glucose Phosphorylase from Arabidopsis thaliana. *Biochemistry* **2006,** *45* (10), 3154-3162.
163. Hou, W.-T.; Li, W.-Z.; Chen, Y.; Jiang, Y.-L.; Zhou, C.-Z., Structures of Yeast Apa2 Reveal Catalytic Insights into a Canonical Ap4A Phosphorylase of the Histidine Triad Superfamily. *Journal of Molecular Biology* **2013,** *425* (15), 2687-2698.
164. Barnes, L. D.; Garrison, P. N.; Siprashvili, Z.; Guranowski, A.; Robinson, A. K.; Ingram, S. W.; Croce, C. M.; Ohta, M.; Huebner, K., Fhit, a Putative Tumor Suppressor in Humans, Is a Dinucleoside 5‘,5‘ “-P1,P3-Triphosphate Hydrolase. *Biochemistry* **1996,** *35* (36), 11529-11535.
165. Terasaka, N.; Kimura, S.; Osawa, T.; Numata, T.; Suzuki, T., Biogenesis of 2-agmatinylcytidine catalyzed by the dual protein and RNA kinase TiaS. *Nature Structural & Molecular Biology* **2011,** *18* (11), 1268-1274.
166. Wang, Z.; Shen, H.; He, B.; Teng, M.; Guo, Q.; Li, X., The structural mechanism for the nucleoside tri- and diphosphate hydrolysis activity of Ntdp from Staphylococcus aureus. *The FEBS Journal* **2021,** *288* (20), 6019-6034.
167. Sato, Biochemical and Structural Analysis of FomD That Catalyzes the Hydrolysis of Cytidylyl (S)-2-Hydroxypropylphosphonate in Fosfomycin Biosynthesis. *Biochemistry* **2018**.
168. Simon, M. A.; Ongpipattanakul, C.; Nair, S. K.; van der Donk, W. A., Biosynthesis of fosfomycin in pseudomonads reveals an unexpected enzymatic activity in the metallohydrolase superfamily. *Proceedings of the National Academy of Sciences* **2021,** *118* (23).
169. Gottlin, E. B.; Rudolph, A. E.; Zhao, Y.; Matthews, H. R.; Dixon, J. E., Catalytic mechanism of the phospholipase D superfamily proceeds via a covalent phosphohistidine intermediate. *Proceedings of the National Academy of Sciences* **1998,** *95* (16), 9202-9207.
170. Vinitsky, A.; Grubmeyer, C., A new paradigm for biochemical energy coupling. Salmonella typhimurium nicotinate phosphoribosyltransferase. *Journal of Biological Chemistry* **1993,** *268* (34), 26004-26010.
171. Cao, Quinolinate Phosphoribosyltransferase: Kinetic Mechanism for a Type II PRTase. *Biochemistry* **2002**.
172. Wang, T.-F.; Guidotti, G., CD39 Is an Ecto-(Ca2+,Mg2+)-apyrase. *Journal of Biological Chemistry* **1996,** *271* (17), 9898-9901.
173. Ashihara, H.; Stasolla, C.; Fujimura, T.; Crozier, A., Purine salvage in plants. *Phytochemistry* **2018,** *147*, 89-124.

174. Laliberté, Kinetic effects of Ca2+ and Mg2+ on ATP hydrolysis by the purified ATP diphosphohydrolase. *J Biol Chem* **1982**.
175. Permyakov, E. A.; Ha, H. J.; Kwon, S.; Jeong, E. M.; Kim, C. M.; Lee, K. B.; Kim, I.-G.; Park, H. H., Structure of natural variant transglutaminase 2 reveals molecular basis of gaining stability and higher activity. *Plos One* **2018,** *13* (10).
176. Han, B.-G.; Cho, J.-W.; Cho, Y. D.; Jeong, K.-C.; Kim, S.-Y.; Lee, B. I., Crystal structure of human transglutaminase 2 in complex with adenosine triphosphate. *International Journal of Biological Macromolecules* **2010,** *47* (2), 190-195.
177. Benson, R. B. J.; Frigot, R. A.; Goswami, A.; Andres, B.; Butler, R. J., Competition and constraint drove Cope's rule in the evolution of giant flying reptiles. *Nature Communications* **2014,** *5* (1).
178. Issur, M.; Geiss, B. J.; Bougie, I.; Picard-Jean, F.; Despins, S.; Mayette, J.; Hobdey, S. E.; Bisaillon, M., The flavivirus NS5 protein is a true RNA guanylyltransferase that catalyzes a two-step reaction to form the RNA cap structure. *Rna* **2009,** *15* (12), 2340-2350.
179. Zhang, K.; Law, M. C. Y.; Nguyen, T. M.; Tan, Y. B.; Wirawan, M.; Law, Y.-S.; Jeong, L. S.; Luo, D., Molecular basis of specific viral RNA recognition and 5′-end capping by the Chikungunya virus nsP1. *Cell Reports* **2022,** *40* (4).
180. Jones, R.; Bragagnolo, G.; Arranz, R.; Reguera, J., Capping pores of alphavirus nsP1 gate membranous viral replication factories. *Nature* **2020,** *589* (7843), 615-619.
181. Rey, F. A.; Zhao, Y.; Soh, T. S.; Zheng, J.; Chan, K. W. K.; Phoo, W. W.; Lee, C. C.; Tay, M. Y. F.; Swaminathan, K.; Cornvik, T. C.; Lim, S. P.; Shi, P.-Y.; Lescar, J.; Vasudevan, S. G.; Luo, D., A Crystal Structure of the Dengue Virus NS5 Protein Reveals a Novel Inter-domain Interface Essential for Protein Flexibility and Virus Replication. *PLOS Pathogens* **2015,** *11* (3).
182. Zhao, Y.; Soh, T. S.; Lim, S. P.; Chung, K. Y.; Swaminathan, K.; Vasudevan, S. G.; Shi, P.-Y.; Lescar, J.; Luo, D., Molecular basis for specific viral RNA recognition and 2′-O-ribose methylation by the dengue virus nonstructural protein 5 (NS5). *Proceedings of the National Academy of Sciences* **2015,** *112* (48), 14834-14839.
183. Osawa, T.; Aoki, M.; Ehara, H.; Sekine, S.-i., Structures of dengue virus RNA replicase complexes. *Molecular Cell* **2023,** *83* (15), 2781-2791.e4.
184. Jia, H.; Zhong, Y.; Peng, C.; Gong, P.; Parrish, C. R., Crystal Structures of Flavivirus NS5 Guanylyltransferase Reveal a GMP-Arginine Adduct. *Journal of Virology* **2022,** *96* (14).
185. Todone, F.; Weinzierl, R. O. J.; Brick, P.; Onesti, S., Crystal structure of RPB5, a universal eukaryotic RNA polymerase subunit and transcription factor interaction target. *Proceedings of the National Academy of Sciences* **2000,** *97* (12), 6306-6310.
186. Tamura, Y.; Harada, Y.; Nishikawa, S.-i.; Yamano, K.; Kamiya, M.; Shiota, T.; Kuroda, T.; Kuge, O.; Sesaki, H.; Imai, K.; Tomii, K.; Endo, T., Tam41 Is a CDP-Diacylglycerol Synthase Required for Cardiolipin Biosynthesis in Mitochondria. *Cell Metabolism* **2013,** *17* (5), 709-718.
187. Hover, B. M.; Tonthat, N. K.; Schumacher, M. A.; Yokoyama, K., Mechanism of pyranopterin ring formation in molybdenum cofactor biosynthesis. *Proceedings of the National Academy of Sciences* **2015,** *112* (20), 6347-6352.
188. Grochowski, L. L.; Xu, H.; Leung, K.; White, R. H., Characterization of an Fe2+-Dependent Archaeal-Specific GTP Cyclohydrolase, MptA, from Methanocaldococcus jannaschii. *Biochemistry* **2007,** *46* (22), 6658-6667.
189. Horikawa, S.; Sasuga, J.; Shimizu, K.; Ozasa, H.; Tsukada, K., Molecular cloning and nucleotide sequence of cDNA encoding the rat kidney S-adenosylmethionine synthetase. *Journal of Biological Chemistry* **1990,** *265* (23), 13683-13686.

190. Sekula, B.; Ruszkowski, M.; Dauter, Z., S-adenosylmethionine synthases in plants: Structural characterization of type I and II isoenzymes from Arabidopsis thaliana and Medicago truncatula. *International Journal of Biological Macromolecules* **2020,** *151*, 554-565.
191. Sheppard, D. E.; Penrod, J. T.; Bobik, T.; Kofoid, E.; Roth, J. R., Evidence that a B12-adenosyl transferase is encoded within the ethanolamine operon of Salmonella enterica. *Journal of Bacteriology* **2004,** *186* (22), 7635-7644.
192. Nar, H.; Huber, R.; Heizmann, C. W.; Thöny, B.; Bürgisser, D., Three-dimensional structure of 6-pyruvoyl tetrahydropterin synthase, an enzyme involved in tetrahydrobiopterin biosynthesis. *The EMBO Journal* **1994,** *13* (6), 1255-1262.
193. Bürgisser, D. M.; Thöny, B.; Redweik, U.; Hess, D.; Heizmann, C. W.; Huber, R.; Nar, H., 6-Pyruvoyl Tetrahydropterin Synthase, An Enzyme With a Novel Type of Active Site Involving Both Zinc Binding and an Intersubunit Catalytic Triad Motif; Site-directed Mutagenesis of the Proposed Active Center, Characterization of the Metal Binding Site and Modelling of substrate Binding. *Journal of Molecular Biology* **1995,** *253* (2), 358-369.
194. Morris, E. R.; Caswell, S. J.; Kunzelmann, S.; Arnold, L. H.; Purkiss, A. G.; Kelly, G.; Taylor, I. A., Crystal structures of SAMHD1 inhibitor complexes reveal the mechanism of water-mediated dNTP hydrolysis. *Nature Communications* **2020,** *11* (1).
195. Deng, W. H.; Lewin, H.; Liao, R. Z.; Rosta, E., Reaction Mechanism and Metal Selectivity of Human SAMHD1 Elucidated by QM/MM Calculations. *ACS Catal* **2025,** *15* (12), 10176-10187.
